# An RNase domain-dependent microRNA detection with DNA-spiked nanocage for accurate cancer diagnosis

**DOI:** 10.1038/s41467-026-76169-0

**Published:** 2026-07-29

**Authors:** Hye Hyun Kim, Yong Hwan Seol, Dae Kwon Park, Young Eun Jang, Jeewon Lee

**Affiliations:** Department of Chemical and Biological Engineering, Korea University, Seoul, Republic of Korea

**Keywords:** Assay systems, Diagnosis, Biosensors

## Abstract

Cancer-associated microRNAs are promising biomarkers for early cancer diagnosis. However, current microRNA detections – which are based on polymerase chain reaction, microarray analysis, Raman scattering, or various electrochemical assays – rely on multi-step analysis including amplification of microRNA and/or read-out signals, often causing inaccuracy despite using elaborate instruments, which remains a major challenge. Here, we describe an RNaseH1-dependent, amplification-free detection of microRNAs, which is based on Förster resonance energy transfer (FRET) between donor (single-stranded DNA probe (ssDNA)-spiked, green fluorescent nanocage) and acceptor (RNaseH1-linked, orange fluorescent protein) and enables reliable cancer diagnosis with high accuracy for human lung and gastric cancer patients and healthy donors by directly quantifying serum microRNA targets. Notably, unlike traditional ensemble FRET, this FRET platform senses molecular interactions between ssDNA and miRNAs and conformational changes of their hybrid helices and thus differentiates even a single-base mismatch, which holds significant promise for reliable, clinical cancer diagnosis.

## Abstract (web summary)

Direct measurement of circulating microRNAs in serum without prior amplification remains challenging. Here, the authors develop a fluorescent nanocage platform that directly measures serum microRNAs, which can distinguish samples from patients with lung or gastric cancer from those of healthy donors.

## Introduction

MicroRNAs (miRNAs) are non‑coding single-stranded RNAs (18 to 22 nucleotides) that regulate post-transcriptional gene expression. Because they influence cell proliferation, differentiation, and death, dysregulated miRNAs are closely linked to the pathogenesis of many cancers^1–3^. Measuring cancer-associated, circulating miRNAs – which exist in the range of 10 fM to 10 pM in the blood of cancer patients^4–6^ – has thus emerged as an attractive approach for minimally invasive cancer diagnosis and prognosis. Beyond conventional clinical workflows, detection of serum miRNAs could make cancer diagnosis accessible to at-home patients as well as outpatients and lead to significantly earlier diagnosis of various cancers. However, current miRNA detection methods – which are mostly based on reverse transcription-quantitative polymerase chain reaction (RT-qPCR)^7^, microarray analysis^8^, Raman scattering^9^, and electrochemical assays^10^ – have significant drawbacks. The RT-qPCR is a most widely used technique that offers attomolar sensitivity but requires multiple analytical steps including RNA extraction, reverse transcription, and subsequent DNA amplification, which increase turnaround time and cost and potentially compromise accuracy due to sample loss, enzymatic inhibition, or amplification bias. Similarly, although microarray analysis enables broad multiplexing, it also permits base mismatches through cross-hybridization and thus causes false signals because many cancer-associated miRNAs differ by only a single nucleotide^11–13^. To address these limitations, researchers have explored various label-free biosensors (e.g., Raman scattering and various electrochemical assays) for miRNA detection. While these methods avoid some steps including enzymatic miRNA amplification, they still rely on complicated instruments and multi-step analysis such as RNA extraction and/or read-out signal amplification to achieve sufficient sensitivity, which prolongs analysis time, generates assay variability and false signals, and thus often causes inaccuracy problems. Thus, there remains an essential need for simple and accurate detection of cancer-associated miRNAs, which operates directly in serum with avoiding amplification of miRNA and/or read-out signals and gives diagnostic signals with discriminating a single-nucleotide difference among a target and closely related analogues to prevent false positives.

In this context, early-stage lung cancer is frequently asymptomatic, and many patients with non-small-cell lung cancer (NSCLC) are diagnosed after progression to regionally advanced or metastatic stage (stage 3 or 4), which markedly lowers survival rate and thus highlights the need for early and accurate diagnosis of NSCLC^14^. Among circulating miRNAs, miR-196a^15–17^, miR-1290^18,19^, and miR-182-5p^20–22^ have been reported to be oncogenic and upregulated in NSCLC.

To address these needs, we construct a FRET^23^-based assay platform, which is comprised of (1) ssDNA probe-spiked, green fluorescent nanocage (FRET donor), which sensitively captures miRNA target in serum and (2) human RNaseH1-linked, orange fluorescent protein (FRET acceptor), which recognizes only perfect, or mismatch-free miRNA-ssDNA hybrid helix. Notably, beyond conventional ensemble FRET, which makes the FRET acceptor emit orange fluorescence at the excitation wavelength of green fluorescence as long as the two fluorophores are located closely within Förster distance, this RNaseH1-dependent FRET platform senses molecular interactions between ssDNA probe and miRNAs and conformational changes of their hybrid helices and thus gives strong FRET signals only for a perfectly matched hybrid helix. In the design of the FRET donor, we first engineer hepatitis B virus capsid (HBVC comprised of 240 coat proteins^24–29^, a self-assembled nanocage with ~35 nm in diameter) through genetically fusing an enhanced green fluorescent protein (EGFP)^28–30^ to each coat protein, resulting in the green fluorescent nanocage, where 240 EGFPs are localized at the outer surface of each nanocage. Then, we chemically link ssDNA probes – which capture a particular cancer-specific miRNA target – to surface-exposed loops of EGFP on the green fluorescent nanocage, resulting in a three-dimensional (3D) array of both ssDNA probes and green fluorophores around the nanocage. In the design of FRET acceptor, we genetically fuse the hybrid-binding domain (HBD) of human RNaseH1 to orange fluorescent protein (mOrange)^31^. A native role of RNaseH1 removes RNA from DNA-RNA hybrids (R-loops) to stall transcription after its N-terminal HBD specifically recognizes and binds to minor groove of perfectly matched A-form of R-loops with over 25-fold stronger affinity than for double-stranded RNAs or DNAs^32–35^. At the molecular level, the key interactions in the engagement of HBD with R-loops include hydrophobic and electrostatic interactions and hydrogen bonds to 2′-hydroxyl (2′-OH) groups of ribose rings of RNA strands. Even a single-nucleotide mismatch in R-loop distorts helix geometry, dramatically lowers HBD binding affinity, and thus significantly hinders the engagement of HBD with R-loop^32,35,36^. This RNA-DNA hybrid geometry/conformation-sensitive binding of HBD could ensure a differentiation of single-nucleotide mismatch between ssDNA probe and miRNAs upon the interaction between the FRET donor and acceptor.

Here, we further evaluate the applicability of this approach to clinical cancer diagnosis using cohort samples (sera) from 80 NSCLC patients and 80 healthy donors as well as 40 gastric cancer (GC) patients and 40 healthy donors. With ssDNA probes that capture these three NSCLC-specific miRNA markers (miR-196a, miR-1290, and miR-182-5p), we obtain AUC values above 0.93 for all three miRNA markers using a simple mix-and-read protocol in solution that requires neither probe immobilization and washing steps, nor prior extraction and amplification of serum miRNAs, demonstrating that this RNaseH1-dependent FRET platform holds a promising potential for opening opportunities for clinically reliable diagnosis of various cancers.

## Results

### Construction of ssDNA probe-spiked FRET donor and RNaseH1-linked FRET acceptor

#### Genetic variation of HBVC coat protein

**Fig. 1:**
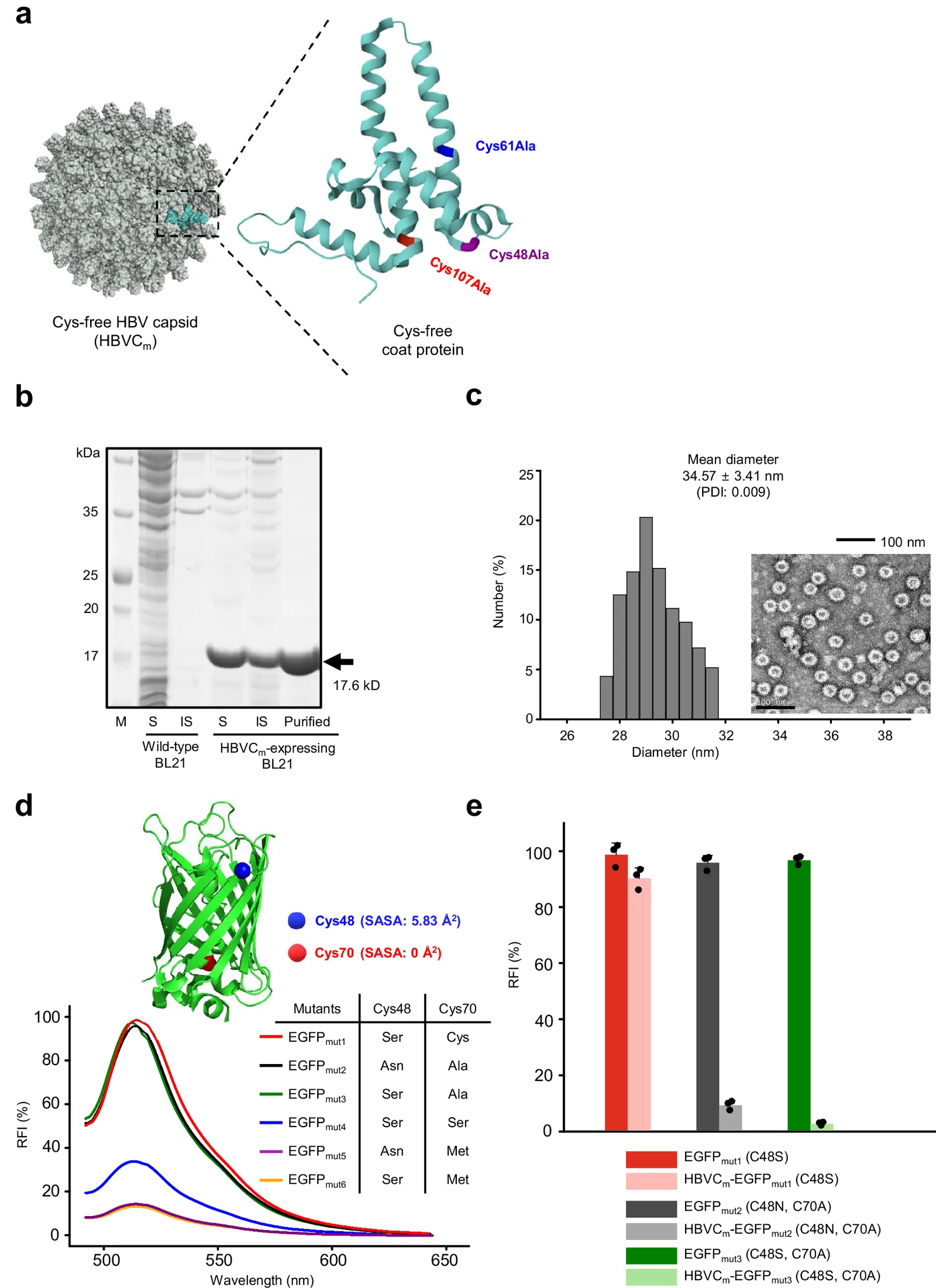
Genetic variation of HBVC coat protein and EGFP. **a** Schematic structure of HBVC mutant (HBVC_m_) comprised of cysteine-free coat proteins (Cys48Ala, Cys61Ala, and Cys107Ala), based on the hepatitis B virus capsid structure (PDB 1QGT). **b** SDS-PAGE analysis of soluble (S) and insoluble (IS) fractions from wild-type and HBVCm-expressing *E. coli* cells and Ni^2+^-affinity purified HBVCm. (M: protein marker) **c** DLS (left) and TEM (right, scale bar: 100 nm) analyses of purified HBVCm. (PDI represents polydispersity index.) **d** EGFP structure (PDB 2Y0G) indicating the position of Cys48 (solvent-accessible surface area (SASA) = 5.83 Å^2^) and Cys70 (SASA = 0 Å^2^) and normalized emission spectra (*λ*_ex_: 488 nm, *λ*_em, max_: 514 nm) of six EGFP mutants (RFI, relative fluorescence intensity with regarding the fluorescence intensity of native EGFP as 100%). **e** Relative fluorescence intensity of three EGFP mutants (EGFP_mut1_ to EGFP_mut3_) before and after fused to HBVC_m_. (RFI of (**d**, **e**) represents relative fluorescence intensity with regarding the fluorescence intensity of native EGFP as 100%.) Data in (**e**) represent mean ± s.d. of three technical replicates. The SDS-PAGE analysis in (**b**) and the DLS and TEM analyses in (**c**) are representative of three independent experiments with similar results. HBVC hepatitis B virus capsid, EGFP enhanced green fluorescent protein, SDS-PAGE sodium dodecyl sulfate–polyacrylamide gel electrophoresis, DLS dynamic light scattering, TEM transmission electron microscopy, PDB Protein Data Bank. Source data are provided as a Source Data file.

We eliminated all cysteine residues (Cys48, Cys61, and Cys107) from native HBVC coat protein through replacing the three Cys residues with Ala^30^, yielding a formation of cysteine-free HBVC mutant (HBVC_m_) (Fig. 1a). When expressed in *Escherichia coli*, the mutated coat proteins are found mostly in soluble fraction (Fig. 1b), and transmission electron microscopy (TEM) and dynamic light scattering (DLS) analyses show that they form *T* = 4 capsid particles (~ 35 nm in diameter) in the cytoplasm of *E. coli* (Fig. 1c), demonstrating that the Cys to Ala mutations do not hamper the native self-assembly function of coat protein to form native HBVC architecture.

#### Genetic variation of EGFP

For conjugating ssDNA probes – which are labeled by 3′-maleimide and capture miRNA target – to a particular Cys residue of EGFP, we first removed undesired thiol reactivity from native EGFP, which contains two cysteines (solvent-accessible Cys48 and buried internal Cys70). Given that the solvent‑accessible surface area of Cys48 and Cys70 is ~5.83 and ~0 Å², respectively, it seems that Cys48 is much more likely to undergo a maleimide-coupling reaction than Cys70. We prepared six EGFP mutants through replacing Cys48 and/or Cys70 with other residues^37–39^ as presented in Fig. 1d, among which EGFP_mut1_ (Cys48Ser), EGFP_mut2_ (Cys48Asn, Cys70Ala), and EGFP_mut3_ (Cys48Ser, Cys70Ala) exhibited nearly the same fluorescence emission spectra and retained more than 95% of native fluorescence intensity of EGFP (Fig. 1d, Suppl. Fig. 1a). Further, Cy5-labeled maleimide was never conjugated to the three mutants above (Suppl. Fig. 2), indicating the total elimination of reactive thiols from native EGFP.

Next, we genetically fused each of EGFP_mut1_, EGFP_mut2_, and EGFP_mut3_ to the N-terminus of HBVC_m_ coat protein and expressed the fusion protein in *E. coli*. Among the three fusion constructs, only HBVC_m_-EGFP_mut1_ was expressed as a soluble form in the bacterial cytoplasm (Suppl. Fig. 1b), and the purified HBVC_m_-EGFP_mut1_ has a uniform spherical shape (~ 40 nm) like *T* = 4 capsid particle (Suppl. Fig. 3) and retains nearly 90% of fluorescence intensity of EGFP_mut1_ alone (Fig. 1e, Suppl. Fig. 4). That is, HBVC_m_-EGFP_mut1_ emits native-like green fluorescence but has no reactive thiols.

**Fig. 2:**
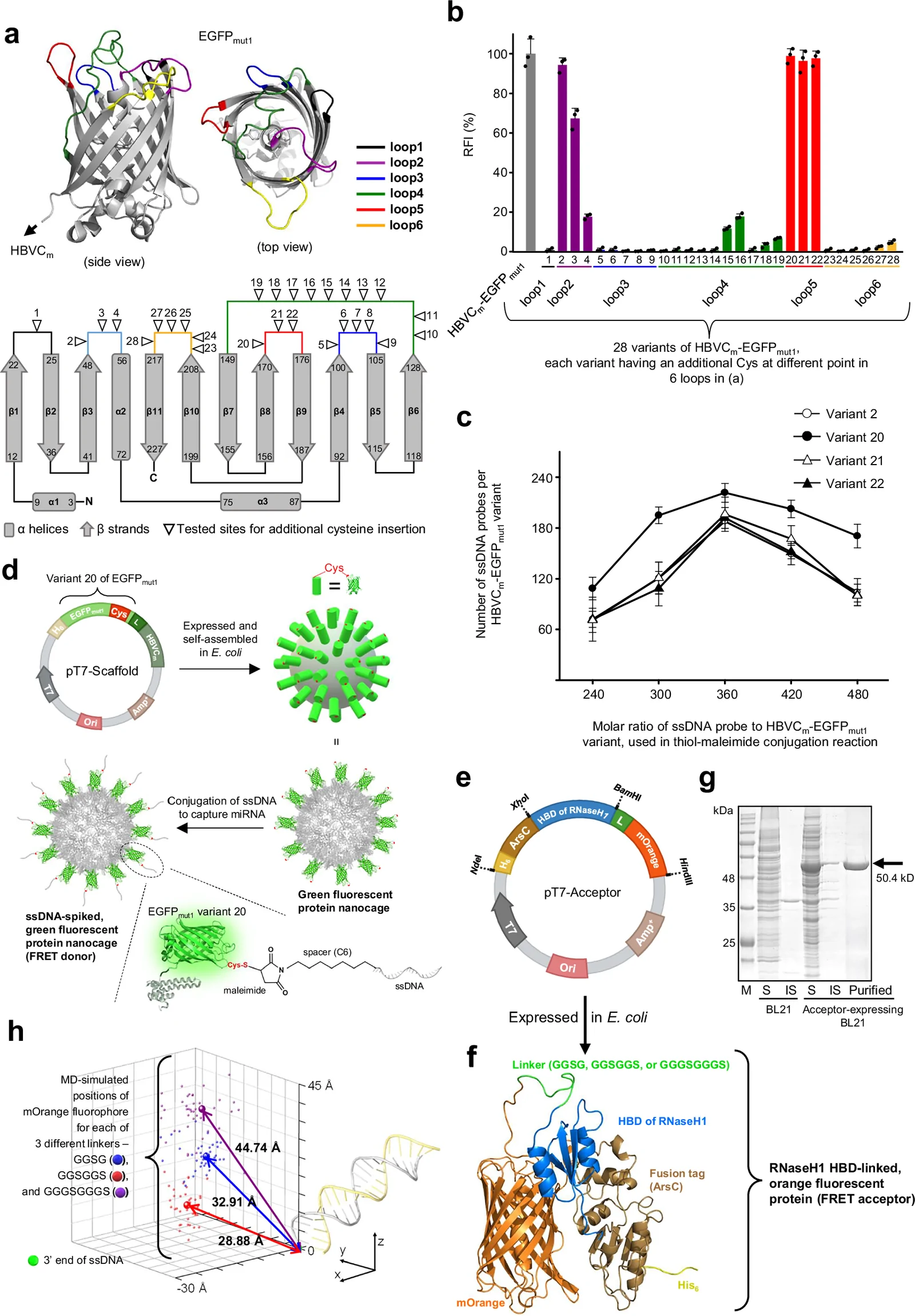
Construction of FRET donor and acceptor. **a** Structure of EGFP_mut1_, which is presumed to be identical to that of native EGFP and thus highlights six solvent-exposed loops (loop 1 to loop 6) and 28 provisional points (triangles) for inserting a single cysteine. **b** Relative fluorescence intensity of HBVC_m_-EGFP_mut1_ and its 28 variants (RFI: relative fluorescence intensity with regarding the fluorescence intensity of HBVC_m_-EGFP_mut1_ as 100%.) **c** The number of ssDNA probes conjugated to each of four HBVC_m_-EGFP_mut1_ variants (variant 2, 20, 21, or 22) via thiol-maleimide reaction, with varying the molar ratio of ssDNA probe to each variant from 240 to 480. **d** Schematic presentation of ssDNA probe-spiked, green fluorescent protein nanocage (FRET donor), which is constructed through conjugating ssDNA probes to the variant 20 of HBVC_m_-EGFP_mut1_ that is self-assembled immediately after the expression of plasmid vector, pT7-Scaffold in *E. coli*. A flexible C6 spacer was introduced between the 3′ end of ssDNA and maleimide to minimize steric hindrance in the binding of miRNA to ssDNA probe. **e** Plasmid expression vector, pT7-Acceptor, which codes for the synthesis of FRET acceptor (NH_2_-H_6_-ArsC-RNaseH1 HBD-L-mOrange-COOH) in *E. coli*, where H_6_ and L are hexa-histidine tag for Ni^2+^-affinity purification and a flexible Gly/Ser linker, respectively. **f** AlphaFold2 structure model of the FRET acceptor obtained through an explicit-solvent MD simulation. **g** SDS-PAGE analysis of soluble (S) and insoluble (IS) fractions of FRET acceptor-expressing *E. coli* cells and Ni^2+^-affinity purified FRET acceptor (M: protein marker). **h** MD-simulated distance between the 3′ end of ssDNA probe and mOrange fluorophore. The big sphere symbols represent the average coordinate positions of mOrange fluorophore when each of three linkers (GGSG, GGSGGS, and GGGSGGGS, respectively) was inserted between RNaseH1 and mOrange, whereas the small sphere symbols around each big symbol represent all MD-simulated positions of mOrange fluorophore for each linker above. Data in (**b**) represent mean ± s.d. of three technical replicates. Values in (**c**) represent mean ± s.d. of three independent conjugation reactions. The SDS-PAGE analysis in (**g**) is representative of three independent experiments with similar results. FRET Förster resonance energy transfer, ssDNA single-stranded DNA, EGFP enhanced green fluorescent protein, HBVCm cysteine-free hepatitis B virus capsid mutant, HBD hybrid-binding domain, ArsC arsenate reductase, MD molecular dynamics, SDS-PAGE sodium dodecyl sulfate–polyacrylamide gel electrophoresis. Source data are provided as a Source Data file.

Then, we attempted to genetically insert a Cys residue to a particular position of EGFP_mut1_ for the site-specific thiol conjugation of 3′-maleimide-labeled ssDNA probes. It is presumed that EGFP_mut1_ has a native EGFP-like structure, comprised of canonical 11-stranded β‑barrel architecture capped by short α‑helices, because it exhibits nearly the same fluorescence emission spectra as native EGFP. In particular, it is worthy of noting that six loops (loop 1 to loop 6 of Fig. 2a) are located at a fully solvent‑accessible region on the outer surface of HBVC_m_-EGFP_mut1_, suggesting that the loops are appropriate site for introducing a single Cys residue. Based on the previous reports on conformational and local electrostatic properties of native EGFP^40–42^, we chose 28 provisional points in the six outward‑facing loops and genetically inserted a Cys into each provisional point, resulting in the 28 variants of HBVC_m_-EGFP_mut1_ (Fig. 2a, b, Suppl. Fig. 5a). Among them, only four variants – variant 2, 20, 21, and 22 – retained more than 94% of the fluorescence intensity of HBVC_m_-EGFP_mut1_ and were synthesized as a soluble nanocage with spherical shape (~40 nm) in the cytoplasm of *E. coli*, as confirmed by TEM and DLS analyses (Fig. 2b, Suppl. Figs. 5b–g).

#### Construction of ssDNA-spiked, green fluorescent protein nanocage (FRET donor)

A Cy5-labeled ssDNA probe with 3′-maleimide – which is complementary to a well-characterized miRNA (let-7a: 3′-UUG AUA UGU UGG AUG AUG GAG U-5′)^43^ – was chemically attached to the newly introduced and reduced Cys residue of each of the variant 2, 20, 21, and 22 of HBVC_m_-EGFP_mut1_ via thiol-maleimide conjugation^44^, leading to the construction of ssDNA probe-spiked, HBVC_m_-EGFP_mut1_ variants. Even though theoretical moles of ssDNA probe that saturates all available thiols of a mole of HBVC_m_-EGFP_mut1_ variant is 240, the molar ratio of ssDNA probe to each variant was varied from 240 to 480 in the conjugation reaction, because the access of ssDNA probes to thiols might be sterically limited or maleimide groups might be hydrolyzed^45,46^. After the unconjugated, free ssDNA probes were removed through sequential Ni-NTA affinity purification followed by 100 kDa spin filtration, the amount of ssDNA probes conjugated to each variant of HBVC_m_-EGFP_mut1_ was quantified by measuring Cy5 fluorescence (Suppl. Fig. 6), showing that among the four variants, the largest amount of ssDNA probes was measured with the variant 20, which is approximately 222 ( ~ 92% of theoretical maximum, 240) per variant 20 (Fig. 2c). Consequently, the ssDNA probe-spiked variant 20 of HBVC_m_-EGFP_mut1_ was used as a green fluorescent FRET donor which captures cancer-specific miRNA target. A flexible C6 spacer was introduced between 3′-end of ssDNA probe and maleimide to minimize steric hindrance in the binding of miRNA target to the ssDNA probes (Fig. 2d). Since the high stability of the variant 20 of HBVC_m_-EGFP_mut1_ (nanocage) is essential to constructing the stable FRET donor, it was further investigated as follows: the synthesized nanocage was stored at −80 °C, and the frozen nanocage was thawed at 2-week intervals and analyzed through DLS and TEM for 6 weeks. As a result, the spherical shape of nanocage with a mean diameter of 38 to 40 nm (PDI of 0.002 ~ 0.003) is constantly observed for 6 weeks with neither inter-nanocage aggregation nor structural collapse (Suppl. Fig. 7), indicating the stable nature of the variant 20 of HBVC_m_-EGFP_mut1_.

Given that recent advances in AI-assisted de novo protein design are rapidly expanding the repertoire of programmable nanocages with diverse architectures^47^, a more optimal protein nanocage with different geometry (e.g., octahedral, icosahedral, etc.) could be designed for enhancing the function of FRET donor in the future, although here we rationally designed a particular protein nanocage from native HBVC.

#### Construction of RNaseH1-linked, orange fluorescent protein (FRET acceptor)

The FRET acceptor was constructed through genetically linking the C-terminus of HBD of human RNaseH1 to the N-terminus of orange fluorescent protein (mOrange)^31^ after the genes were codon-optimized for *E. coli* expression. A flexible Gly/Ser linker (GGSG, GGSGGS, or GGGSGGGS, to be discussed later) was also introduced between the HBD and mOrange. Hexa-histidine (H_6_) tag and ArsC (arsenate reductase)^48^ were genetically fused to the N-terminus of HBD for Ni^2+^ affinity purification and solubility-enhancing expression of FRET acceptor, respectively (Fig. 2e–g, Suppl. Fig. 8). As seen in the AlphaFold2 structural model of FRET acceptor (Fig. 2f), which was obtained through an explicit-solvent molecular dynamics (MD) simulation (10 ns), it looks likely that the HBD surface is unobstructed by the adjacent ArsC and mOrange. This suggests that without undergoing a significant steric hindrance, the HBD of FRET acceptor freely recognizes A-form minor-groove geometry of ssDNA probe-miRNA (hybrid helix), followed by fluorescence energy transfer from the FRET donor to acceptor and subsequent ratio-metric optical read-out from mOrange, which is enabled because the EGFP in FRET donor and the mOrange in FRET acceptor fill a requirement of Förster spectral overlap (Suppl. Fig. 9).

We evaluated how each of the three flexible Gly/Ser linkers above (GGSG, GGSGGS, and GGGSGGGS, Fig. 2f) influences steric separation between the ssDNA probes in FRET donor and the mOrange in FRET acceptor through all-atom MD- (100 ns) and docking simulations, demonstrating that the distance between mOrange fluorophore and the 3′ end of ssDNA probe is 28.9, 32.9, and 44.7 Å with GGSGGS, GGSG, and GGGSGGGS linkers, respectively and thus that the distance was minimized with the six-residue linker (GGSGGS) (Fig. 2h, Suppl. Fig. 10). Further, we estimated FRET ratio – ratio of acceptor to donor fluorescence emission upon donor excitation – in the detection of target miRNA (let-7a spiked in healthy serum) at various concentrations (10 to 10^9^ fM) using both of the aforementioned FRET donor (i.e. ssDNA probe-spiked, variant 20 of HBVC_m_-EGFP_mut1_) and each of the three FRET acceptors with the different linker above, showing that the GGSGGS linker resulted in the highest FRET ratio over the entire range of tested concentrations of let-7a (Suppl. Fig. 11). Consequently, the FRET acceptor with the GGSGGS linker was selected and used for all further experiments.

### RNaseH1-dependent, amplification-free detection of serum miRNAs

**Fig. 3:**
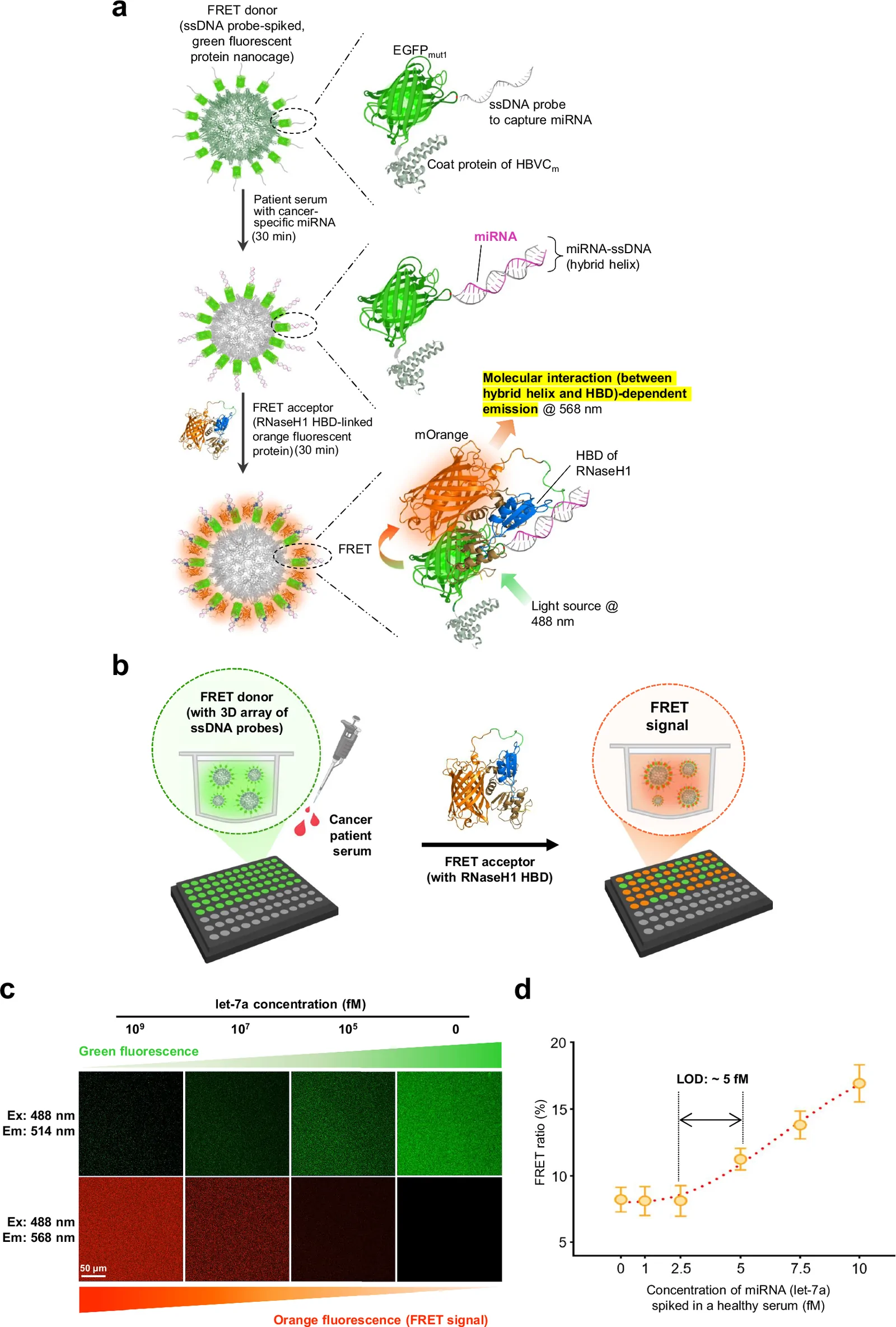
RNaseH1-dependent, amplification-free detection of serum miRNA. **a** Schematic description of serum miRNA detection using RNaseH1-dependent FRET platform: FRET donor captures miRNA target to form a hybrid helix, and the helical conformation based on the molecular interaction between miRNA and ssDNA probe is recognized by RNaseH1 HBD of FRET acceptor, followed by the emission of mOrange fluorescence signal at 568 nm upon excitation of EGFP at 488 nm. **b** Schematic illustration of direct, or amplification-free detection of serum miRNA of patients with well-established protocol using off-the-shelf instruments such as microplate reader. **c** Confocal images of EGFP and mOrange fluorescence signals (excitation: 488 nm; emission: 514 nm for EGFP and 568 nm for mOrange) from the detection of serum let-7a (spiked in a healthy serum at the concentrations from 0 to 10^9^ fM) by RNaseH1-dependent FRET platform (scale bar, 50 µm). The confocal images in (**c**) are representative of three independent experiments with similar results. **d** Estimation of FRET ratios in the detection of serum let-7a at 0 (blank) to 10 fM to find the limit of detection (LOD) of RNaseH1-dependent FRET platform. The LOD (= blank mean + 3 × s.d.) was calculated to be around 5 fM. Error bars represent s.d. of three technical replicates. FRET Förster resonance energy transfer, ssDNA single-stranded DNA, HBD hybrid-binding domain, EGFP enhanced green fluorescent protein. Source data are provided as a Source Data file.

Figure 3a, b schematically describe how serum miRNA target is detected through following a simple mix-and-read protocol in solution with off-the-shelf instruments: (1) miRNA target in patient serum – which is never purified and amplified prior to being detected – is captured by complementary ssDNA probes of the FRET donor (Fig. 2d); (2) the resulting hybrid helix is then recognized by HBD of the FRET acceptor (Fig. 2f); and (3) excitation of EGFP at 488 nm leads to emission of mOrange at 568 nm, which is a ratio-metric, miRNA concentration-proportional read-out signal (Fig. 3c). When estimated with a target miRNA (let-7a) spiked in healthy serum, the limit of detection (LOD) was ~5 fM (Fig. 3d), which is below the range of circulating miRNA concentration in the blood of cancer patients (10 fM to 10 pM)^4–6^, indicating that this FRET platform is sensitive enough for cancer diagnosis even without pretreatment steps such as extraction, purification, and/or amplification of miRNA or read-out signals.

Further, batch-to-batch reproducibility and inter-assay variability in miRNA detection by FRET platform were evaluated using the HBVC_m_-EGFP_mut1_ nanocage and the RNaseH1 HBD-linked mOrange (FRET acceptor), which were produced from three independent batches of *E. coli* culture, followed by storage at −80 °C for 6 weeks. At 2-week intervals, the three FRET platforms prepared after thawing each of three batch products were used for detecting let-7a spiked into healthy serum at 0 (blank), 10, 10², and 10³ fM. As shown in Suppl. Fig. 12a–d, the detection signals (FRET ratios) from the three FRET platforms were not significantly distinguished from each other at each concentration of let-7a, which was consistently observed every 2 week for 6 weeks, thus confirming the negligible batch-to-batch- and inter-assay variabilities.

#### Differentiation of a single nucleotide mismatch

**Fig. 4:**
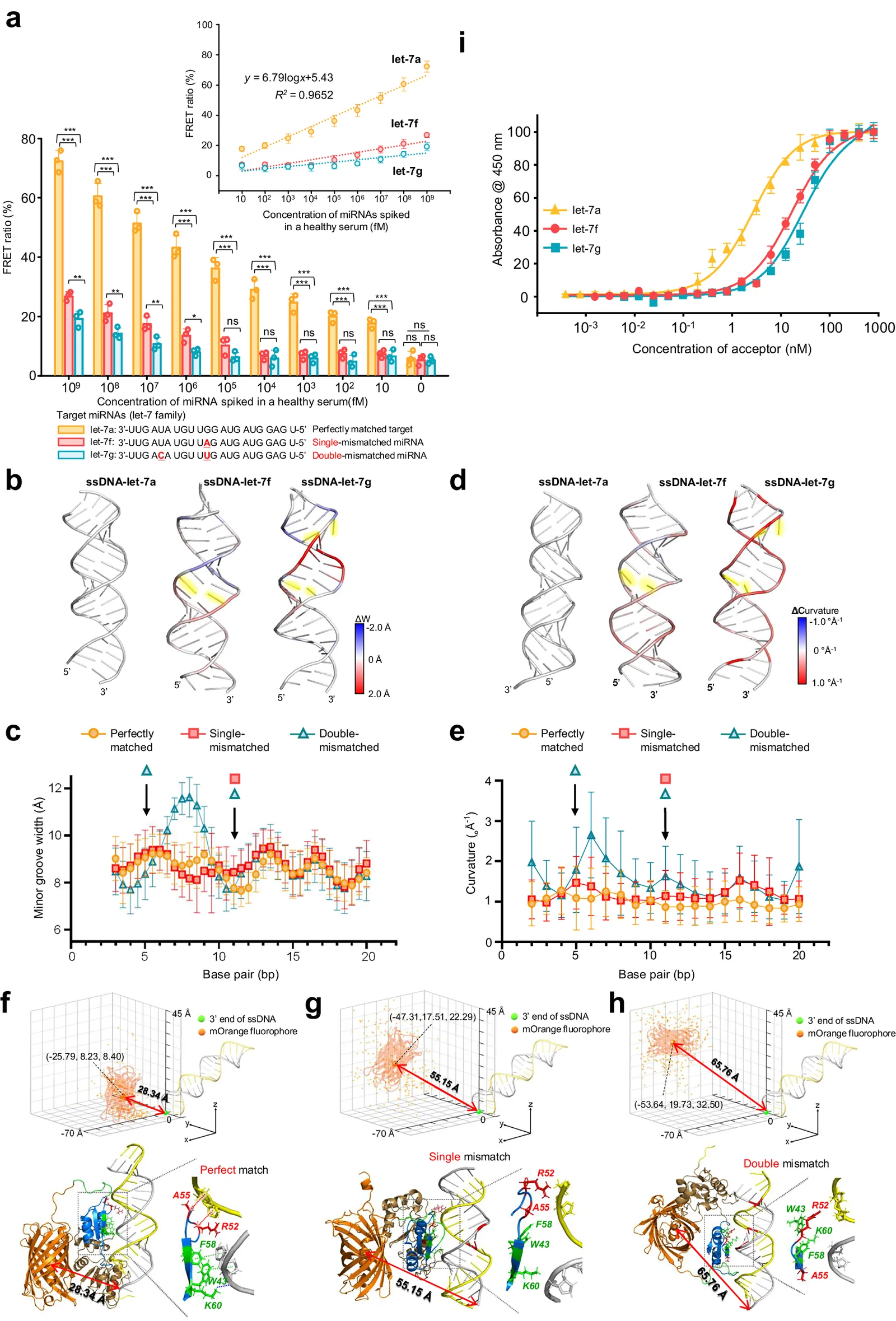
Conformational difference between and differentiation of perfect base match and mismatch in the detection of serum miRNA by RNaseH1-dependent FRET platform. **a** FRET ratios estimated in the detection of let-7 family miRNAs (let-7a (perfectly matched target) and let-7f and 7g (single- and double-mismatched miRNAs, respectively)), spiked into a healthy serum. In the aligned sequences, mismatched bases are shown in bold red. Statistical significance was assessed by ordinary two-way ANOVA (miRNA identity × concentration, with interaction) followed by Tukey’s multiple comparisons test comparing miRNAs within each concentration (two-tailed; multiplicity-adjusted *P*-values); *P < 0.05, **P < 0.01, ***P < 0.001; ns, not significant. Multiplicity-adjusted *P*-values, Tukey *q* statistics, degrees of freedom, and 95% CIs are provided in the Source Data file; values below 0.001 are reported as *P* < 0.001. **b**–**e** All-atom MD-simulated structures (**b**, **d**) and structural element profiles (**c**, **e**) of three hybrid helices (ssDNA-let-7a, ssDNA-let-7f, and ssDNA-let-7g). The simulated structures show the deviations of minor groove width (ΔW), (**b**) and helix-axis curvature (ΔCurvature), (**d**) from perfectly matched hybrid helix with highlighting mismatched base pairs (yellow highlights), and the simulated profiles are about minor groove width, (**c**) and helix-axis curvature, (**e**). The simulations of (**b**–**e**) were performed using GROMACS and CURVES+ . **f**–**h** All-atom MD- and docking simulations of the molecular interactions between each hybrid helix above (ssDNA-let-7a, (**f**), ssDNA-let-7f, (**g**) or ssDNA-let-7g, (**h**)) and RNaseH1 HBD and the distance between mOrange fluorophore (indicating average coordinate position) and 3′ end of the ssDNA probe using AutoDock Vina. The small orange spheres represent all MD-simulated positions of fluorophore, and the yellow and grey strands of hybrid helix represent miRNA and ssDNA probe, respectively. W43, F58, K60, R52, and A55 denote the HBD residues that contact the hybrid helix. **i** ELISA-like plate assay to estimate the b**i**nding affinity of RNaseH1 HBD of FRET acceptor for each hybrid helix above immobilized on well plate at various concentrations. Data in (**a**, **i**) represent mean ± s.d. of three technical replicates; in (**c**, **e**), data are presented as the mean with error bars representing the s.d. across 50 frames of the MD trajectory. FRET Förster resonance energy transfer, ssDNA single-stranded DNA, ANOVA analysis of variance, CI confidence interval, MD molecular dynamics, HBD hybrid-binding domain, ELISA enzyme-linked immunosorbent assay. Source data are provided as a Source Data file.

Given that native function of RNaseH1 HBD is to recognize a perfectly matched A-form of R-loops, the HBD-engaged FRET signaling is subjected to crucial dependence on the conformation of ssDNA probe-miRNA hybrid helix. That is, it is expected that more perfect match between miRNA and ssDNA probe leads to more native-like, tight binding of HBD and thus more proximate interaction between the two fluorophores of donor and acceptor, resulting in higher FRET ratio. To prove this, we attempted to detect three miRNAs of let-7 family (let-7a, 7f, and 7g) (Fig. 4a, showing that let-7f and 7g are one- and two-nucleotide different, respectively, from let-7a) using ssDNA probe that is complementary to let-7a, after the let-7 miRNAs were spiked in human healthy serum. From Fig. 4a and Suppl. Fig. 13, FRET ratio linearly increases as miRNA concentration increases from 10 to 10^9^ fM in serum, and in particular, as compared to let-7a, the FRET ratio for let-7f and 7g are significantly far lower in the entire range of serum miRNA concentration, indicating that both of the single- and double-nucleotide mismatch were successfully discriminated. Further, we examined whether the location of mismatch(es) within the hybrid helix (ssDNA-miRNA) affects the assay performance of FRET platform. We mutated the sequence of ssDNA probe (5′-AAC TAT ACA ACC TAC TAC CTC A-3′) that is complementary to let-7a miRNA (3′-UUG AUA UGU UGG AUG AUG GAG U-5′), resulting in the preparation of 4 different ssDNA probes as follows: 5′-AAC CAT ACA ACC TAC TAC CTC A-3′ (for a single mismatch proximal to 5′ end), 5′-AAC TAT ACA ACC TAC TAC CCC A-3′ (for a single mismatch proximal to 3′ end), 5′-AAC TAT ACA ACC CAC TAC CTC A-3′ (for a single mismatch in middle region), and 5′-AAC CAT ACA ACC TAC TAC CCC A-3′ (for double mismatch). Then, we spiked let-7a into human healthy serum at 10, 10², 10³, and 10⁴ fM and measured readout signals (FRET ratios) from the detection of let-7a by FRET platform using each of let-7a-complementary- and 4 mutated ssDNA probes above. As shown in Suppl. Fig. 14, the let-7a-complementary ssDNA probe emits apparently the highest, concentration-dependent signals compared to the other mutated ssDNA probes, which capture let-7a with single- or double-base mismatch at various positions and emit notably lower, negligible signals. This indicates that the discrimination of mismatched hybrids by HBD of FRET acceptor is not influenced by mismatch position and thus that FRET platform is useful for accurate cancer diagnosis with avoiding false positive signals. This result is explainable with the mechanism of recognizing DNA-RNA hybrids by native HBD, which is that HBD senses overall A-form minor-groove geometry of hybrid helix regardless of mismatch positions. Consequently, the above results suggest that HBD of the FRET acceptor can recognize the conformational difference between perfectly matched and mismatched hybrid helices regardless of number and position of mismatch and thereby significantly influences the molecular proximity between the two fluorophores, which is to be discussed next.

#### Molecular interaction between RNaseH1 HBD and hybrid helix

Perfectly matched A-form of RNA-DNA duplex – which presents a regular array of 2′-OH groups in the minor groove and maintains a uniform helix conformation – is recognized and bound by RNaseH1 HBD^35,36^. Reportedly^35,49,50^, magnitude and pattern of helix distortions that are caused by nucleotide mismatch(es) depend on sequence context and duplex length, and even a single-nucleotide mismatch significantly widens the minor groove of helix and causes local bending, suggesting that sequence mismatch(es) between miRNA target and ssDNA probe could also distort helix geometry, weaken HBD binding, and ultimately increase Förster distance. Through all-atom MD simulations (50 ns) with three canonical A-form models generated by CURVES+ ^51^ for ssDNA-let-7a, ssDNA-let-7f, and ssDNA-let-7g (i.e., perfectly matched, single-mismatched, and double-mismatched hybrid helix, respectively), we identified the most dominant MD clusters through GROMACS simulation using AMBER99SB-ILDN force field^52^ and then analyzed local deviations, relative to the perfectly matched hybrid helix, by mapping minor-groove width (ΔW) and helix curvature (ΔCurvature) under the simulated conditions of solvation and equilibration using CURVES+ (Fig. 4b–e). The ssDNA-let-7a maintains a nearly uniform minor‑groove width of 8 ~ 9 Å across the helix, while the groove of ssDNA-let-7f is widened locally by 1 ~ 2 Å relative to ssDNA-let‑7a around the single mismatch point (Fig. 4b, c). Notably, ssDNA-let‑7g exhibits two distinct groove‑widening hotspots: ΔW of ~2 Å around the first mismatch point (6~8th bp) and ΔW of 1 ~ 2 Å around the second mismatch point (12~13th bp) (Fig. 4b, c). Further, we simulated curvature profile and ΔCurvature-mapped structure along the hybrid helix (Fig. 4d, e), demonstrating that ssDNA-let‑7a has a nearly straight helical axis throughout the entire helix with a relatively uniform curvature (~ 1° Å⁻¹). The single mismatch in ssDNA-let-7f causes a moderate local increase in curvature around the mismatch point, while the double mismatch in ssDNA-let-7g more strongly increases helix‑axis curvature over the broader region including the mismatch points.

Then, we attempted to disclose the more detailed, molecular interaction between HBD and each of ssDNA-let-7a, ssDNA-let-7f, and ssDNA-let-7g in explicit solvent using CHARMM36 force field^53,54^ through all-atom MD- (100 ns) and docking simulations using AutoDock Vina^55^ (Suppl. Fig. 15, Fig. 4f–h). The interaction between HBD and ssDNA-let-7a forms an extensive interface, where groove-sensing residues W43 and F58 closely interact with deoxyribose rings of ssDNA strand; K60 electrostatically contacts with phosphate backbone; and main-chain amides of R52 and A55 form hydrogen bonds with 2′-OH groups of miRNA, corresponding exactly to the previous results^32–34^. Notably, mOrange fluorophore was positioned 28.34 Å apart from the 3′ end of ssDNA (Fig. 4f). Figure 4g also reveals that HBD binds to the ssDNA-let-7f with a slightly tilted angle, showing that W43, F58, and K60 residues are localized at more distant positions from the single-mismatched hybrid helix and that due to incomplete stacking or interaction of HBD with the hybrid helix, the distance between mOrange fluorophore and ssDNA probe (3′ end) increased to 55.15 Å, which is nearly 2-fold increase compared to the HBD binding to ssDNA-let-7a (Fig. 4g). The mOrange fluorophore becomes more far away (65.76 Å) from ssDNA probe (3′ end) upon the binding of HBD to the ssDNA-let-7g due to more loose engagement of HBD with the double-mismatched hybrid helix (Fig. 4h). For the interaction between HBD and each of ssDNA-let-7a, ssDNA-let-7f, and ssDNA-let-7g, HBD-docking scores were also calculated using AutoDock Vina, to be approximately −8.0, −5.7, and −2.2 kcal·mol^−^^1^, respectively (Suppl. Fig. 16). Further, through ELISA-like plate assay^56^ (Methods), dissociation constants (K_d_) representing the binding affinities of HBD for the three hybrid helices above were measured to be 2.6, 16.8, and 27.0 nM, respectively (Fig. 4i), indicating that the binding strength between HBD and hybrid helix decreases progressively as the number of mismatched nucleotides increases. Consequently, the results above (Fig. 4a–i) demonstrate that HBD senses even a slight conformational difference among the hybrid helices – which is caused by one or two nucleotide mismatch – and thus sensitively discriminate even a single nucleotide mismatch, which in turn influences Förster distance and FRET ratio.

### Clinical diagnosis of human lung and gastric cancer

Prior to the clinical diagnosis of two different types of human cancer (NSCLC and GC), we examined if serum RNase activity substantially affects the diagnostic performance under the assay conditions (30 min-capture of serum miRNA by FRET donor + 30 min-detection of ssDNA-miRNA hybrid by FRET acceptor, Fig. 3a). Synthetic let-7a miRNA was spiked into healthy serum at various concentrations (0, 1.0, 2.5, 5.0, 7.5, and 10.0 fM), followed by the let-7a detection by FRET platform in the absence or presence of Protector RNase Inhibitor (0.5 U/µL serum; Roche). As shown in Suppl. Fig. 17, RNase inhibition does not significantly change the assay performance, that is, LOD still remains at femtomolar level although slightly decreased to ~2.5 fM, indicating that serum RNase does not largely hamper the performance of FRET platform, which is presumably because once miRNA-ssDNA hybrids are quickly formed and captured by HBD of FRET acceptor, the complex is recalcitrant to RNase degradation. We also checked out if ssDNA probes of FRET donor selectively capture their sequence-complementary target miRNAs without non-specific cross-reactivity for other non-target serum miRNAs. As shown in Suppl. Fig. 18, each of six different ssDNA probes (three for NSCLC- and three for GC diagnosis) emits the highest readout signal (FRET ratio) for its complementary target miRNA, while the readout signals for other non-target miRNAs as well as for negative control (healthy serum only) are negligible, indicating that highly selective, target-specific detection of miRNA can be performed by FRET platform.

Then, we diagnosed NSCLC with 80 patients (25, 25, 15, and 15 patients at stage I, II, III, and IV, respectively) (Suppl. Table 1) and 80 healthy donors (Suppl. Table 2) through the detection of three NSCLC-associated miRNA markers – miR-196a, miR-1290, and miR-182-5p – using FRET platform. Reportedly, miR-196a and miR-1290 are significantly overexpressed in NSCLC patients^15–19^, and miR-182-5p is also upregulated in NSCLC tissues and sera, because it functions as an oncogene that promotes cancer cell proliferation, migration, and invasion^20–22^. The full sequences of the three miRNA markers and their complementary ssDNA probes are summarized in Suppl. Table 3. Based on the linear correlation between readout signals (FRET ratio (%)) and concentrations (fM) of each of three miRNA markers (Suppl. Fig. 19, Methods), the limit of detection (LOD) for each miRNA marker was estimated to be ~5 fM for all three markers (Suppl. Fig. 20), which is below the concentration range of circulating miRNAs in the blood of cancer patients^57–59^.

**Fig. 5:**
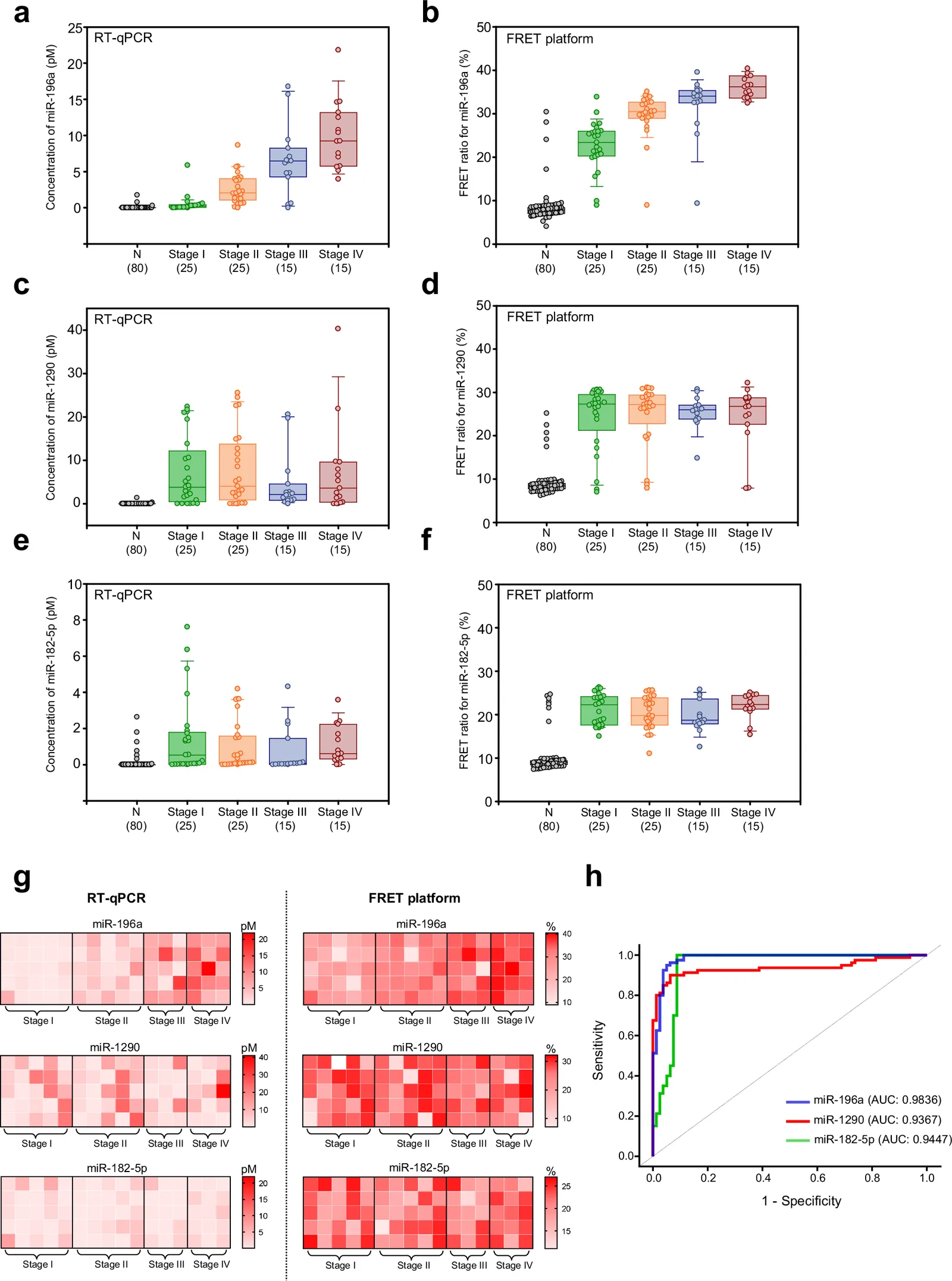
Diagnosis of NSCLC using clinical samples of patient- and healthy group. **a**–**f** Box plots of miRNA levels in the sera of 80 NSCLC patients (stage I–IV) and 80 healthy donors, measured by RT-qPCR (**a**, **c**, **e**) and FRET platform (**b**, **d**, **f**) for miR-196a (**a**, **b**), miR-1290 (**c**, **d**), and miR-182-5p (**e**, **f**). N denotes healthy donors. **g** Heat maps of readout signals from RT-qPCR (color scale, miRNA concentration in pM) and FRET platform (color scale, FRET ratio in %) for the three miRNA markers. **h** ROC curves for the NSCLC diagnosis by FRET platform to detect each of the three miRNA markers (**b**, **d**, **f**). Box plots in (**a**–**f**) show the median (center line), the 25th–75th percentiles (box bounds), whiskers extending to the most extreme values within 1.5× the interquartile range, and all individual data points; the minima and maxima are the smallest and largest plotted values. Each data point represents one biological replicate (an individual patient or donor), shown as the mean of three technical replicates. NSCLC non-small-cell lung cancer, RT-qPCR reverse transcription–quantitative polymerase chain reaction, FRET Förster resonance energy transfer, ROC receiver operating characteristic, AUC area under the curve. Source data are provided as a Source Data file.

The readout signals from 80 patient- and 80 healthy sera, measured by both RT-qPCR and FRET platform, are presented in the box plots (Fig. 5a–f), demonstrating that FRET platform more clearly distinguished patients from healthy group. Compared to RT-qPCR, the difference in the readout signals between from stage-I patients and healthy group is notably higher in the diagnosis by FRET platform. Further, as seen in the heat maps (Fig. 5g) that were plotted using the same diagnostic results of the box plots above, the readout signal patterns of FRET platform and RT-qPCR look very similar, which was also confirmed through the assessment of Spearman’s rank correlation coefficients (*ρ*) (Suppl. Fig. 21). That is, the high *ρ* values – 0.8675, 0.8408, and 0.9212 for miR-196a, miR-1290, and miR-182-5p, respectively (*P* < 0.0001) – indicate strong, monotonic correlations in the readout signals^60,61^ between FRET platform and RT-qPCR, suggesting that FRET platform is a quantitatively reliable diagnostic tool like established RT-qPCR. Finally, we evaluated clinical sensitivity and specificity of FRET platform through ROC (Receiver Operating Characteristic) curve analysis for each miRNA marker (Fig. 5h). The AUC (Area Under the Curve) values – 0.9836 (95% confidence interval (CI): 0.9669 ~ 1.000), 0.9367 (95% CI: 0.8931 ~ 0.9804), and 0.9447 (95% CI: 0.9033 ~ 0.9860) for miR-196a, miR-1290, and miR-182-5p, respectively – show that the FRET platform reliably distinguishes between the patient- and healthy groups. Also, using Youden index (*J* = sensitivity + specificity − 1), which is a widely used criterion for selecting a cutoff point that yields maximum *J* in ROC analysis^62,63^, we estimated the optimal cutoff of FRET ratio to be 13.05%, 12.35%, and 11.06% for miR-196a, miR-1290, and miR-182-5p, respectively. Based on the cutoff values above, clinical sensitivity and specificity were estimated: 95.0 and 95.0%, respectively, for miR-196a; 90.0 and 93.8%, respectively, for miR-1290; and 98.8 and 91.3%, respectively, for miR-182-5p, indicating that FRET platform brings about improved diagnostic performance as compared to the other recent reports on cancer diagnosis^58,64,65^.

We further attempted to diagnose another type of cancer, GC (40 patients and 40 healthy donors; Suppl. Tables 2, 4) using FRET platform through detecting three GC-associated miRNA markers (miR-223-3p, miR-222-3p, and miR-106a-5p)^66–69^. LOD was estimated to be ~5 fM for all three markers (Suppl. Figs. 22, 23), and FRET platform also clearly distinguished GC patients from healthy group (Suppl. Fig. 24). The heat maps for 40 GC patients (10 at each stage of I to IV) (Suppl. Fig. 25a) and *ρ* values between FRET platform and RT-qPCR – 0.9131, 0.8989, and 0.8678 (*P* < 0.0001) for each marker (Suppl. Fig. 26) – demonstrate that FRET platform and RT-qPCR have a strong, monotonic correlations in the readout signals^60,61^ like in the diagnosis of NSCLC. Further, the quantitative performance of GC diagnosis was analyzed with each of three GC-associated markers through ROC analysis as follows: AUC of 0.9494, 0.9188, and 0.8838, sensitivity of 95.0% (for all three markers), and specificity of 92.5, 87.5, and 85.0% (Suppl. Fig. 25b), indicating that FRET platform also enables a reliable diagnosis and is quantitatively better than or comparable to other recent results of cancer diagnosis^58,64,65^.

## Discussion

Circulating miRNAs are attractive cancer biomarkers that can be detected through minimally invasive manner; however, their femto-to-picomolar abundance in the blood and high sequence similarity among target and closely related analogues make it difficult to detect a particular cancer-specific miRNA target in serum with filling both low LOD and high accuracy. Current technologies of miRNA detection, including RT-qPCR, microarray analysis, Raman scattering, and various electrochemical assays, typically rely on multi-step analysis for the amplification of miRNAs extracted from serum and/or readout signals, which involves sample loss, polymerase inhibition, amplification bias, or cross-hybridization and thereby prolongs turnaround time, increases cost, and often suffers from false positives due to inaccurate discrimination between a correct target and target-like analogues. To address this challenge, we built up here an RNaseH1 domain (HBD)-dependent FRET platform, which operates directly in serum without miRNA amplification and quantifies serum miRNAs with high detection accuracy that allows a single-nucleotide resolution. The traditional ensemble FRET cannot or rarely probes conformational changes and molecular interactions although it is a powerful spectroscopic method for measuring Förster distances in 2 to 8 nm range; however, this FRET platform senses conformational change of ssDNA-miRNA hybrid helix and bimolecular interaction between the hybrid helix in FRET donor and RNaseH1 HBD in the FRET acceptor and thus makes the conformation change-dependent molecular interaction detectable as fluorescence signals. That is, we extend here the capability of ensemble FRET beyond bimolecular distance measurements through integrating the HBD function of human RNaseH1 and a 3D architecture of ssDNA-spiked nanocage into a FRET platform. A particular noteworthy of this platform is that HBD of human RNaseH1 – which specifically binds to minor groove of perfectly matched A-form of R-loops and weakens its binding strength to mismatched DNA-RNA hybrid – here makes it possible to sensitively discriminate nucleotide mismatch between ssDNA probe and miRNAs regardless of mismatch position. This was evidenced by the simulated data about variation in minor-groove width, helix curvature, molecular distance between acceptor fluorophore and ssDNA probe, and HBD-docking scores of perfectly matched and mismatched hybrid helices and also by the experimental data about FRET ratio and molecular affinity of HBD for the hybrid helices. Another key component of this FRET platform is a 3D architecture of ssDNA-spiked, fluorescent nanocage (FRET donor), which allows a 3D array of ssDNA probes and donor fluorophores through their localization on the surface of viral capsid nanocage and thus significantly enhances diffusional access of both miRNA target and acceptor fluorophores to ssDNA probes and donor fluorophores, respectively. Although native HBVC was rationally engineered here and used as a nanocage scaffold, recent advances in AI-assisted de novo protein design are rapidly expanding the repertoire of programmable nanocages with diverse architectures, and a more optimal protein nanocage with different geometry could be designed to enhance the capability of FRET donor through the de novo protein design in the future.

Using clinical cohort samples (80 NSCLC patient- and 80 healthy sera; 40 GC patient- and 40 healthy sera), we applied this FRET platform to the diagnosis of human NSCLC and GC through detecting three NSCLC-associated miRNA markers (miR-196a, miR-1290, and miR-182-5p) and three GC-associated miRNA markers (miR-223-3p, miR-222-3p, and miR-106a-5p), which were selected based on the previous findings that they are significantly upregulated in each type of cancer tissues^15–22,66–69^, although testing additional closely related miRNAs would strengthen diagnostic specificity. Prior to the clinical diagnosis, we confirmed that serum matrix effects by hemolysis and serum RNase are negligible (Suppl. Figs. 17, 27), but matrix heterogeneity and assay variability across clinical serum specimens remain to be characterized with other independent clinical cohorts. The FRET platform resulted in high sensitivity (90.0 ~ 98.8% for NSCLC; 95.0% for GC) and specificity (91.3 ~ 95.0% for NSCLC; 85.0 ~ 92.5% for GC), which were all estimated through systematic ROC analysis (AUC 0.88 ~ 0.98) for each miRNA marker (Fig. 5h and Suppl. Fig. 25b). Notably, a significantly large difference in readout signals (FRET ratios) between stage I- and II patients and healthy group was observed in the diagnosis by FRET platform for both NSCLC and GC, and thus FRET platform is likely effective for early cancer diagnosis. As compared to the recent reports on direct serum/plasma-detection technologies for detecting miRNAs, such as single-molecule electro-optical nanopore^70^, nanophotonic BiMW biosensor^57^, nanoplasmonic LSPR assay^71^, and CRISPR-based systems^58,59^, FRET platform demonstrates better diagnostic performance in terms of LOD and clinical sensitivity and specificity, although it tested far larger-size cohort (totally 120 patients and 80 healthy donors) through following a simple mix-and-read protocol in solution without additional steps for miRNA extraction and amplification and used off-the-shelf instruments such as well-plates and microplate reader instead of elaborately designed, sophisticated instruments (Suppl. Table 5). Although it is inevitable to prepare ssDNA probe-spiked FRET donor and RNaseH1 HBD-linked FRET acceptor through recombinant expression and purification, it seems that this platform holds a promising potential for clinically reliable diagnosis of various types of cancer through detecting cancer-associated, multiple miRNA markers with different sequences and/or length with high selectivity (Suppl. Table 6), which is readily implemented by simply switching the ssDNA probes while keeping the other components unchanged.

## Methods

### Ethical statement

All procedures were performed in accordance with the Guidelines for the Care and Use of Human Biological Materials of Korea University and were approved as exempt by the Institutional Review Board of Korea University (IRB Nos. KUIRB-2024-0439-04 and KUIRB-2026-0152-02); informed consent was not required for these previously collected, de-identified banked specimens. No participants were recruited or compensated for this study. Sex was obtained from the KIRAMS Radiation Biobank records and was not self-reported; gender was not collected. No sex- or gender-based analysis was performed, because the study was designed to validate the analytical performance of the platform. To prevent identification of individuals, participant ages in Suppl. Tables 1, 2, and 4 are reported in 10-year age ranges, while sex is retained. No information that could identify individual participants is reported; therefore, separate consent for publication of identifiable information was not required.

### Synthesis of ssDNA probe-spiked fluorescent nanocage (FRET donor)

An expression plasmid encoding the N-terminal green fluorescent protein nanocage (HBVC_m_-EGFP_mut1_ variant 20) was constructed. Two plasmid templates were mutated using the Q5® Site-Directed Mutagenesis Kit (Cat. No. E0554S; New England Biolabs, Ipswich, MA, USA) to generate (i) HBVC_m_Ag(1–149), in which Cys48, Cys61 and Cys107 were replaced with alanine, and (ii) EGFP_mut1_ variant 20, in which Cys48 was replaced with serine and an additional cysteine codon was inserted between Ile171 and Glu172 (I171-E172insCys). The resulting open reading frames were fused by overlap-extension PCR to generate the cassette NH_2_-*Nde*I-H_6_-EGFP_mut1_ variant 20-*Bam*HI-G_3_SG_3_T-HBVC_m_Ag(1–149)-*Hin*dIII-COOH. The cassette was digested with *Nde*I and *Hin*dIII and ligated into the pT7-7 expression vector digested with the same enzymes to generate pT7-Scaffold. The plasmid sequence was verified before transformation into *Escherichia coli* BL21 (DE3), and ampicillin-resistant colonies were selected.

The recombinant *E. coli* harboring pT7-Scaffold was cultured in LB medium at 37 °C until the optical density at 600 nm reached 0.5. Protein expression was induced with 1 mM isopropyl *β*-D-1-thiogalactopyranoside (IPTG), and cultures were incubated overnight at room temperature with shaking. Cells were harvested by centrifugation at 5311 × *g* for 5 min, and the resulting pellet was resuspended in lysis buffer (50 mM Tris-HCl, 10 mM imidazole, pH 8.5). Cells were disrupted using a Branson sonifier (Branson Ultrasonics Corp., Danbury, USA), and lysates were clarified by centrifugation at 24,562 × *g* for 10 min. The recombinant scaffold was purified from the soluble fraction by Ni^2+^-affinity chromatography using Ni-NTA agarose (Qiagen, Hilden, Germany) as follows: (1) prior to sample loading, the column resin was washed with 10 mL of buffer A (50 mM Tris-HCl, 10 mM imidazole, pH 8.5); (2) following sample loading and binding under batch mode conditions at 4 °C for 1 h, the resin was washed again with 10 mL of buffer B (50 mM Tris-HCl, 50 mM imidazole, pH 8.5); and (3) the recombinant protein was subsequently eluted with 2 mL of elution buffer (50 mM Tris-HCl, 250 mM imidazole, pH 8.5), followed by buffer exchange to Tris buffer (50 mM Tris-HCl, pH 8.5) using ultrafiltration (Amicon Ultra 100 K, Millipore)^72^.

For ssDNA probe conjugation, purified scaffold (20 nM) was diluted in Tris buffer 1 (50 mM Tris-HCl, pH 8.5), supplemented with 1.2 mM dithiothreitol (DTT) and 2 mM ethylenediamine-tetraacetic acid (EDTA), and incubated for 3 h at room temperature with gentle shaking. The reduced scaffold was desalted into Tris buffer 2 (50 mM Tris-HCl, 500 mM NaCl, pH 7.0) using a desalting column and then mixed with a maleimide-functionalized ssDNA probe (Bioneer Inc., Daejeon, Republic of Korea) at a 360-fold molar excess relative to nanocage particles (i.e., 1.5-fold relative to the 240 conjugation sites per particle) and incubated overnight at 25 °C. The full sequences of the target miRNAs and their complementary ssDNA probes used in this work are listed in Suppl. Table 3. The reaction mixture was applied to Ni-NTA agarose, washed extensively, and the His-tagged scaffold-DNA conjugate was eluted with 250 mM imidazole. Free oligonucleotides and imidazole were removed by ultrafiltration using a 100 kDa MWCO Amicon® Ultra centrifugal filter, yielding the ssDNA probe-spiked donor nanocage (FRET donor). For quantification of ssDNA loading per nanocage, a 5′-Cy5-labeled version of the ssDNA probe (same sequence as above) was conjugated in parallel under identical conditions (e.g., 5′-Cy5-AAC TAT ACA ACC TAC TAC CTC A-C6 Spacer-Maleimide-3′ for the let-7a probe, or 5′-Cy5-CCC AAC AAC ATG AAA CTA CCT A-C6 Spacer-Maleimide-3′ for the miR-196a probe). After removal of free probes, Cy5 fluorescence (Ex/Em = 646/662 nm) was measured using a microplate reader and compared with a calibration curve generated from known concentrations of the Cy5-labeled ssDNA probe processed in parallel. The number of ssDNA molecules per particle was calculated from the molar amount of conjugated ssDNA and the molar concentration of donor nanocage particles. Unless otherwise stated, unlabeled ssDNA probes were used for FRET assays.

### Synthesis of RNaseH1 HBD-linked mOrange protein (FRET acceptor)

An expression plasmid encoding an N-terminal hexa-histidine-tagged hybrid-binding domain (HBD) of human RNaseH1 (residues 27–76) fused to mOrange was constructed by overlap-extension PCR to generate the fragment *Xho*I-D_4_K-RNaseH1 HBD(27–76)-*Bam*HI-linker[G_2_SG_2_S]-mOrange-*Hin*dIII, where D_4_K denotes the Asp-Asp-Asp-Asp-Lys sequence (DDDDK). This PCR product replaced the hG-CSF region in the parental vector *Nde*I-H_6_-ArsC-*Xho*I-hG-CSF-*Hin*dIII^48^ via *Xho*I/*Hin*dIII digestion and ligation to yield the intermediate construct (NH₂-*Nde*I-H_6_-ArsC-*Xho*I-D_4_K-RNaseH1 HBD(27–76)-*Bam*HI-G_2_SG_2_S-mOrange-*Hind*III-COOH). The insert was excised by *Nde*I/*Hin*dIII digestion and ligated into the pT7-7 expression vector to generate pT7-Acceptor. The plasmid sequence was verified before transformation into *E. coli* BL21 (DE3), and ampicillin-resistant colonies were selected.

The recombinant *E. coli* harboring pT7-Acceptor was cultured in LB medium at 37 °C until the optical density at 600 nm reached 0.5. Expression was induced with 1 mM IPTG and cultures were incubated overnight at room temperature with shaking. Cells were harvested by centrifugation at 5311 × *g* for 5 min, and resuspended in lysis buffer (50 mM Tris-HCl, 10 mM imidazole, pH 8.5). Cells were disrupted using a Branson sonifier (Branson Ultrasonics Corp., Danbury, USA), and lysates were clarified by centrifugation at 24,562 × *g* for 10 min. The recombinant acceptor was purified from the soluble fraction by Ni^2+^-affinity chromatography using Ni-NTA agarose (Qiagen, Hilden, Germany) as follows: (1) prior to sample loading, the column resin was washed with 10 mL of buffer A (50 mM Tris-HCl, 10 mM imidazole, pH 8.5); (2) following sample loading and binding under batch mode conditions at 4 °C for 1 h, the resin was washed again with 10 mL of buffer B (50 mM Tris-HCl, 50 mM imidazole, pH 8.5); and (3) the recombinant protein was subsequently eluted with 2 mL of elution buffer (50 mM Tris-HCl, 250 mM imidazole, pH 8.5), followed by buffer exchange to Tris buffer (20 mM Tris-HCl, pH 7.4) using ultrafiltration (Amicon Ultra 30 K, Millipore)^72^. The plasmids pT7-Scaffold and pT7-Acceptor generated in this study are available from the corresponding author (leejw@korea.ac.kr) under a standard material transfer agreement.

### TEM and DLS analyses

Transmission electron microscopy (TEM) of HBVC protein nanocages was performed using a Tecnai 20 (FEI, Hillsboro, Oregon, U.S.A.) operated at 200 kV. For sample preparation, a droplet of the colloidal solution was deposited onto a 200-mesh carbon-coated copper grid (TED PELLA, Inc., Redding, CA, USA) and allowed to adsorb for 5 min. Grids were stained with 2% (w/v) uranyl acetate solution for 10 min and air-dried for 1 h. TEM images were acquired using a CCD (charge-coupled device) camera and processed using Gatan DigitalMicrograph (v 3.9.4) installed on the Tecnai 20. The hydrodynamic diameters of the nanocages were measured by dynamic light scattering (DLS) using an ELSZ-1000 zeta potential and particle size analyzer (Otsuka Electronics, Osaka, Japan). Data represent the mean ± s.d. of 60 measurements and are presented as a number distribution.

### Preparation of serum samples

Serum specimens from 80 NSCLC patients, 40 GC patients, and 80 healthy donors were obtained from the Korea Institute of Radiological and Medical Sciences (KIRAMS) Radiation Biobank in the Republic of Korea. All serum specimens of patients were collected before receiving anticancer treatment. Although several independent external cohorts are necessary for complete clinical validation, here we performed a clinical diagnosis using the cohort samples from a single biobank (the KIRAMS Radiation Biobank). The demographic, clinical, and histopathological characteristics and smoking status of the NSCLC- and GC patients and the demographic information of healthy donors are summarized in Suppl. Tables 1, 4, and 2, respectively. All procedures were performed in accordance with the Guidelines for the Care and Use of Human Biological Materials of Korea University and were approved as exempt by the Institutional Review Board of Korea University (IRB Nos. KUIRB-2024-0439-04 and KUIRB-2026-0152-02). All serum specimens were aliquoted into a single-use volume upon receipt from the KIRAMS Radiation Biobank and stored at −80 °C; each aliquot was thawed only once immediately before the assay, and no specimen underwent repeated freeze-thaw cycles. The serum specimens were consumed during the analyses and were not retained for further study. Hemolysis of sera was assessed by measuring the absorbance ratio at 414- and 375 nm (hemolysis index, A₄₁₄/A₃₇₅) using a microplate reader; all specimens showed a hemolysis index below 2.0 (Suppl. Fig. 27). All miRNAs (let-7a, let-7f, let-7g, miR-196a, miR-1290, miR-182-5p, miR-223-3p, miR-222-3p, and miR-106a-5p; GenePharma, Shanghai, China) were serially diluted in normal human serum (Cat. No. S1; Sigma-Aldrich, St. Louis, MO, USA) to final concentrations ranging from 1 µM to 10 fM. The sequences of all ssDNA probes and miRNA targets used in this study are provided in Suppl. Table 3.

### Fluorescence signal measurements and confocal imaging

For microplate assays, FRET donor (20 nM; 50 µL) was mixed with miRNA-containing serum (50 µL) in a black 96-well microplate (Nunc F96 Micro Well Black Polystyrene plate, Cat. No. 137101; Thermo Fisher Scientific) and incubated for 30 min at 37 °C. FRET acceptor (4 µM; 100 µL) was then added, followed by incubation for 30 min at room temperature with gentle shaking. Fluorescence was recorded using a microplate reader (Infinite M200 PRO; TECAN, Zürich, Switzerland) with excitation/emission (Ex/Em) settings of 488/514 nm (donor), 548/568 nm (acceptor), and 488/568 nm (FRET signal). The ratiometric FRET signal was calculated as the acceptor-to-donor emission ratio upon 488 nm excitation. For confocal imaging, samples were prepared in 5-mm glass-bottom dishes by incubating FRET donor (20 nM; 50 µL) with serum containing miRNA (let-7a, 50 µL) at a concentration of 1 × 10⁹, 1 × 10⁷ or 1 × 10⁵ fM for 30 min at 37 °C. FRET acceptor (4 µM; 100 µL) was then added, followed by incubation for 30 min at room temperature. Imaging was performed using a confocal laser-scanning microscope (LSM 700, Carl-Zeiss, Jena, Germany) with 488 nm excitation; emission was collected at 514 nm (EGFP) and 568 nm (mOrange/FRET). For RNase-inhibition assay, synthetic let-7a was spiked into healthy serum at 0, 1.0, 2.5, 5.0, 7.5, and 10.0 fM. Protector RNase Inhibitor (Roche, Basel, Switzerland) was added to serum at 0.5 U/µL before incubation with the donor. FRET donor (20 nM, 50 µL) was mixed with serum (50 µL) for 30 min at 37 °C, followed by addition of FRET acceptor (4 µM, 100 µL) and incubation for 30 min at room temperature before fluorescence measurement. For estimating the effect of mismatch position on the binding between hybrid helix and FRET acceptor, we mutated the sequence of ssDNA probe (5′-AAC TAT ACA ACC TAC TAC CTC A-3′) complementary to let-7a miRNA (a cancer-associated miRNA marker, 3′-UUG AUA UGU UGG AUG AUG GAG U-5′), resulting in the preparation of 4 different ssDNA probes as follows: 5′-AAC CAT ACA ACC TAC TAC CTC A-3′ (for a single mismatch proximal to 5′ end), 5′-AAC TAT ACA ACC TAC TAC CCC A-3′ (for a single mismatch proximal to 3′ end), 5′-AAC TAT ACA ACC CAC TAC CTC A-3′ (for a single mismatch in middle region), and 5′-AAC CAT ACA ACC TAC TAC CCC A-3′ (for double mismatch). Then, we spiked let-7a into human healthy serum at 10, 10^2^, 10^3^, and 10^4^ fM and measured readout signals (FRET ratios) from the detection of let-7a by FRET platform using each of let-7a-complementary- and 4 mutated ssDNA probes above.

We also tested the cross-reactivity between ssDNA probes of FRET donor and various miRNAs with different sequences. That is, FRET donor with each of six ssDNA probes (complementary to miR-196a, miR-1290, miR-182-5p, miR-223-3p, miR-222-3p, or miR-106a-5p) was incubated with each of the six miRNA markers above (10⁴ fM in healthy serum) and a negative control (miRNA marker-free healthy serum) at a time, following the same microplate assay protocol described above.

### RT-qPCR analysis of serum miRNAs in patients and healthy donors

Serum miRNA concentrations were determined by RT-qPCR using the Mir-X miRNA First-Strand Synthesis and TB Green® qRT-PCR kit (Cat. No. 638314; Takara Bio, USA) with standard-curve-based absolute quantification. miRNA was isolated from 200 µL of serum using the XENOPURE™ Plasma Small RNA Purification Kit (Cat. No. 93667873-SP; XENO HELIX, Republic of Korea) and eluted in 20 µL of RNase-free water. Reverse transcription was performed according to the manufacturer′s protocol: RNA samples and synthetic miRNA standards were subjected in parallel to polyadenylation and first-strand cDNA synthesis using the supplied mRQ Buffer and mRQ Enzyme Mix. The reaction mixture (10 µL total) was incubated at 37 °C for 1 h and then at 85 °C for 5 min, followed by dilution to 100 µL with RNase-free water. qPCR was carried out on a CFX96 Touch Real-Time PCR Detection System (Bio-Rad, USA) in 25 µL total volume containing TB Green Advantage Premix, a target-specific forward primer corresponding to the full mature miRNA sequence, the supplied mRQ 3′ primer, and 2 µL of cDNA. Cycling conditions were 95 °C for 10 s, followed by 40 cycles of 95 °C for 5 s and 60 °C for 20 s, with a dissociation-curve step. For absolute quantification, synthetic miRNA standards were reverse-transcribed and amplified in parallel with the serum samples, and sample concentrations were interpolated from the Ct–log[miRNA] calibration curve (Suppl. Fig. 28). No endogenous reference-based normalization was applied. All reactions were performed in technical triplicate and concentrations are reported as mean ± s.d. No-template controls (NTCs) were included for each primer set. To evaluate matrix recovery and assay variability, synthetic miR-196a was spiked into healthy human serum at 0, 1.0, 2.5, 5.0, 7.5, and 10.0 fM and processed through the same workflow; recovery and variability data are summarized in Suppl. Table 7.

### Batch-to-batch reproducibility and stability

We produced three independent batches of both EGFP_mut1_-linked nanocage (variant 20) and HBD-linked FRET acceptor from three separate *E. coli* BL21(DE3) cultures (400 mL each). Then immediately, FRET platform was prepared through constructing FRET donor by attaching ssDNA probes onto the EGFP_mut1_-linked nanocage produced from each batch and subsequently was used for detecting miRNA (let-7a) spiked into healthy serum at 0 (blank), 10, 10², and 10³ fM, while the remaining FRET platform were aliquoted into a single-use volume and stored at −80 °C for 6 weeks. Afterwards, the frozen FRET platform was thawed at 2-week intervals and used to detect miRNA (let-7a), through following the standard microplate assay protocol.

To evaluate whether the stability of EGFP_mut1_-linked nanocage (variant 20) – which is critical to stable construction of FRET donor – lasts during 6-week period of storage at −80 °C, the frozen nanocage was thawed at 2-week intervals and analyzed through DLS and TEM.

### Estimation of dissociation constants (K_d_) in the binding of HBD to hybrid helices

Streptavidin-coated 96-well plates (Pierce™ Streptavidin Coated Plates; Cat. No. 15118; Thermo Fisher Scientific) were loaded with 100 µL of a pre-annealed duplex formed from 5′-biotinylated DNA (20 µM; complementary to let-7a) and equimolar miRNA (let-7a, let-7f or let-7g) in hybridization buffer (20 mM Tris-HCl, 150 mM NaCl, 10 mM MgCl_2_, 5 mM KCl, pH 7.4). Plates were incubated at 4 °C for 24 h and washed three times with PBS containing 0.05% (v/v) Tween-20 (PBS-T). The FRET acceptor was serially diluted (0–1000 nM) in binding buffer (20 mM HEPES-HCl, 100 mM NaCl, 5% glycerol, 10 mM DTT, 0.5 mM EDTA, pH 7.0), added to wells (100 µL per well), and incubated for 4 h at 25 °C with gentle shaking. After washing with PBS-T, bound protein was detected using an HRP-conjugated anti-His-Tag antibody (His-probe Antibody (H-3) HRP, Cat. No. sc-8036 HRP; Santa Cruz Biotechnology; 1:200 dilution in PBS-T) and developed with TMB substrate (3,3′,5,5′-tetramethylbenzidine; Cat. No. T0440; Sigma-Aldrich, St. Louis, MO, USA). After 10 min, the reaction was stopped with 50 µL of 1 M H₂SO₄, and absorbance at 450 nm was measured using an Infinite M200 PRO plate reader (TECAN). Apparent dissociation constants (K_d_) were obtained by fitting binding curves to a one-site model in GraphPad Prism.

### MD simulations for the geometry of hybrid helices

ssDNA-miRNA hybrid helix models (22 bp; ssDNA-let-7a, ssDNA-let-7f, and ssDNA-let-7g) were generated in CURVES+ (v3.0) using a canonical A-form geometry. Each helix was placed in a periodic cubic box (1.2 nm padding) and solvated with TIP3P water. The systems were neutralized and adjusted to 0.15 M NaCl and 20 mM Tris. All simulations were performed in GROMACS 2021.4 using the AMBER99SB-ILDN force field. Energy minimization (steepest descent, 50,000 steps) was performed until the maximum force was below 1000 kJ/mol/nm. Equilibration consisted of NVT (300 K; V-rescale thermostat; 250,000 steps) followed by NPT (1 bar; Parrinello–Rahman barostat; 250,000 steps). All bonds involving hydrogen were constrained by LINCS, enabling a 2 fs timestep. Production simulations were run for 50 ns with coordinates saved every 10 ps. A 1.0 nm cutoff was used for short-range nonbonded interactions and particle-mesh Ewald (PME) was used for long-range electrostatics. Trajectories were clustered (GROMOS; 0.2 nm RMSD cutoff), and the representative structure was taken from the most populated cluster. Representative structures were analyzed in CURVES+ to quantify local minor groove width and helix curvature.

### All-atom MD and docking simulations for the interaction between HBD and hybrid helices

Molecular docking and molecular dynamics (MD) simulations were performed to characterize the interaction between the RNaseH1 hybrid-binding domain (HBD) (in the context of the FRET acceptor, RNaseH1 HBD-linked mOrange protein) and three RNA–DNA hybrid helices (ssDNA-let-7a, ssDNA-let-7f, and ssDNA-let-7g). Representative hybrid-helix conformations were obtained from the pre-equilibrated trajectories described above. The full RNaseH1 HBD-linked mOrange fusion structure was predicted using AlphaFold2 (ColabFold implementation, via the Neurosnap web server; job submitted 2025-04-17) and subsequently refined by an explicit-solvent MD simulation (10 ns; RNaseH1 HBD-linked mOrange protein-only refinement) in GROMACS 2021.4 using the CHARMM36-jul2022 force field and the TIP3P water model to obtain a relaxed fusion-protein structure for subsequent complex construction. For docking, the HBD domain alone was docked to each hybrid helix using AutoDock Vina (v1.2.3) with a cubic search box of 30 × 30 × 30 Å positioned to cover the hybrid-helix minor-groove region; Vina was run with default parameters unless otherwise stated. For each system, the top-scoring docking pose (lowest predicted ΔG) was selected, and the resulting HBD–helix complex was merged with the MD-refined full RNaseH1 HBD-linked mOrange fusion structure by structural alignment of the HBD domain, yielding the initial coordinates of the full fusion–hybrid helix complex. Each full fusion–hybrid helix complex was solvated in a cubic box (1.2 nm padding), neutralized, and adjusted to 0.15 M NaCl. Following energy minimization, the system was equilibrated in the NVT ensemble at 300 K for 250 ps (dt = 1 fs; 250,000 steps) using the V-rescale thermostat, and subsequently equilibrated in the NPT ensemble at 300 K and 1 bar for 50 ps (dt = 1 fs; 50,000 steps) using the Parrinello–Rahman barostat (*τ*_p_ = 2.0 ps). Production simulations of the full RNaseH1 HBD-linked mOrange protein-hybrid helix complexes were performed for 100 ns in explicit solvent using the CHARMM36-jul2022 force field and TIP3P water model, with PME electrostatics and a 1.2 nm cutoff for short-range interactions. A 2 fs timestep was used during production with all bonds involving hydrogen constrained using LINCS. Coordinates were saved every 10 ps, and trajectories were used to assess differences in HBD positioning and minor-groove engagement across mismatch conditions.

### Statistics & Reproducibility

No data were excluded from the analyses, that is, no data points were excluded as outliers and all measured values were used for data analysis. No statistical method was used to predetermine sample size; sample sizes were determined by the availability of specimens from the KIRAMS Radiation Biobank. The experiments were not randomized, and the investigators were not blinded to allocation during experiments and outcome assessment. ROC curves were plotted, and AUC (Area Under the Curve) values with 95% asymptotic confidence intervals were calculated using GraphPad Prism (version 10.6.1; GraphPad Software, San Diego, CA, USA) to evaluate the diagnostic performance of FRET platform for each miRNA marker. Also, using the Youden index (*J* = sensitivity + specificity − 1), which is a widely used criterion for selecting a cutoff point that maximizes overall classification performance in ROC analysis^62,63^, we estimated the optimal cutoff of FRET ratio for each of six miRNA markers for NSCLC and GC through calculating *J* at every possible threshold along the ROC curve and determined an optimal cutoff that yields maximum *J*. The above statistical analyses for ROC, AUC, and optimal cutoff values were performed using the same cohort samples from a single biobank (the KIRAMS Radiation Biobank).

Spearman’s rank correlation coefficients (*ρ*) were calculated using GraphPad Prism to assess the monotonic agreement between the readout signals from FRET platform and RT-qPCR, resulting in a nonlinear (logarithmic) relationship between the two readout signals.

Through the residual normality test upon using two-way ANOVA to statistically compare two different data groups (Fig. 4a, Suppl. Fig. 12), we confirmed that the residuals were not significantly deviated from a normal distribution, which was validated through multiple protocols including D’Agostino–Pearson, Anderson–Darling, Shapiro–Wilk, and Kolmogorov–Smirnov. That is, FRET ratios for perfectly matched and mismatched miRNA targets (Fig. 4a) were analyzed by ordinary two-way ANOVA with miRNA identity (let-7a, let-7f, and let-7g) and miRNA concentration as fixed factors using GraphPad Prism. miRNA concentration was treated as a categorical factor, and the full model including the miRNA identity × concentration interaction was fitted. For post hoc analysis, pairwise comparisons among miRNAs were performed at each concentration by comparing column means within each row using Tukey’s multiple comparisons test. Each concentration was treated as a separate family of comparisons, and multiplicity-adjusted *P* values were reported. *P* < 0.05 was considered statistically significant and is denoted as *P < 0.05, **P < 0.01, ***P < 0.001; ns, not significant.

In this study, in vitro analyses or clinical diagnoses were performed properly through the measurement of both technical and biological replicates. Technical-replicate measurement denotes that the measurement was repeated three times for every single specimen, and the results were presented as mean ± s.d. For biological-replicate measurements in clinical diagnoses, we used multiple serum samples for a single type of cancer (80 sera for NSCLC and 40 sera for GC) as well as for healthy control (80 sera) to minimize inter-patient variability. (For example, in Fig. 5 and Suppl. Fig. 24, multiple data points at each clinical stage as well as at healthy control represent biological replicates, and each data point is the mean of measured values from three technical replicates.) Box plots were generated using SigmaPlot (version 10.0; Systat Software, San Jose, CA, USA) and show median (center line), interquartile range (box), 1.5× interquartile range (whiskers), and individual data points. Data are presented as mean ± s.d. unless otherwise stated. All statistical tests were two-tailed unless otherwise stated.

### Software

Data were acquired using the instrument manufacturers’ software: i-control (version 1.7; TECAN) for the Infinite M200 PRO microplate reader, ZEN 2012 black edition (Carl Zeiss) for the LSM 700 confocal microscope, Photal ELSZ-1000 software (version 5.01; Otsuka Electronics) for DLS, CFX Manager (version 3.0; Bio-Rad) for RT-qPCR, and Gatan DigitalMicrograph (version 3.9.4) for TEM. Three-dimensional scatter plots were generated using Origin (version 8.5; OriginLab). Molecular structures obtained from the MD and docking simulations, the AlphaFold2-predicted FRET acceptor model, and the EGFP structure retrieved from PDB entry 2Y0G were visualized using PyMOL (version 3.1.4; Schrödinger). The HBVC structure retrieved from PDB entry 1QGT was rendered using the RCSB PDB 3D View (web-based; no versioned release). No custom code was used in this study.

### Reporting summary

Further information on research design is available in the Nature Portfolio Reporting Summary linked to this article.

## Supplementary information


Suppl. Information
Reporting Summary
Transparent Peer Review file


## Source data


Source Data


## Data Availability

All data generated in this study that support the findings are available within the paper, its Suppl. Information, and the accompanying Source Data file. Specifically, source data underlying the graphs and statistical analyses are provided as a Source Data file, and the demographic, clinical, and histopathological characteristics of the serum specimens are provided in Suppl. Tables 1, 2, and 4. The protein structures used in this study are available in the Protein Data Bank under accession codes 2Y0G (EGFP) and 1QGT (hepatitis B virus capsid). Source data are provided with this paper.
