# Supplementary material for: An RNase domain-dependent microRNA detection with DNA-spiked nanocage for accurate cancer diagnosis
Source: Nat Commun. 2026 Jul 29;17:9198. doi: 10.1038/s41467-026-76169-0 (PMC13522518; doi:10.1038/s41467-026-76169-0)
Supplement: Supplementary file 1 [file 41467_2026_76169_MOESM1_ESM.pdf]

Supplementary Information

An RNase domain-dependent microRNA detection with DNA-spiked nanocage for accurate cancer diagnosis

Hye Hyun Kim[1], Yong Hwan Seol[1], Dae Kwon Park[1], Young Eun Jang[1], Jeewon Lee[1]*

[1]Department of Chemical and Biological Engineering, Korea University, Anam-Dong 5-1, Seongbuk-Gu, Seoul 02841, Republic of Korea.

* Corresponding author. Email: leejw@korea.ac.kr.

Supplementary Figures

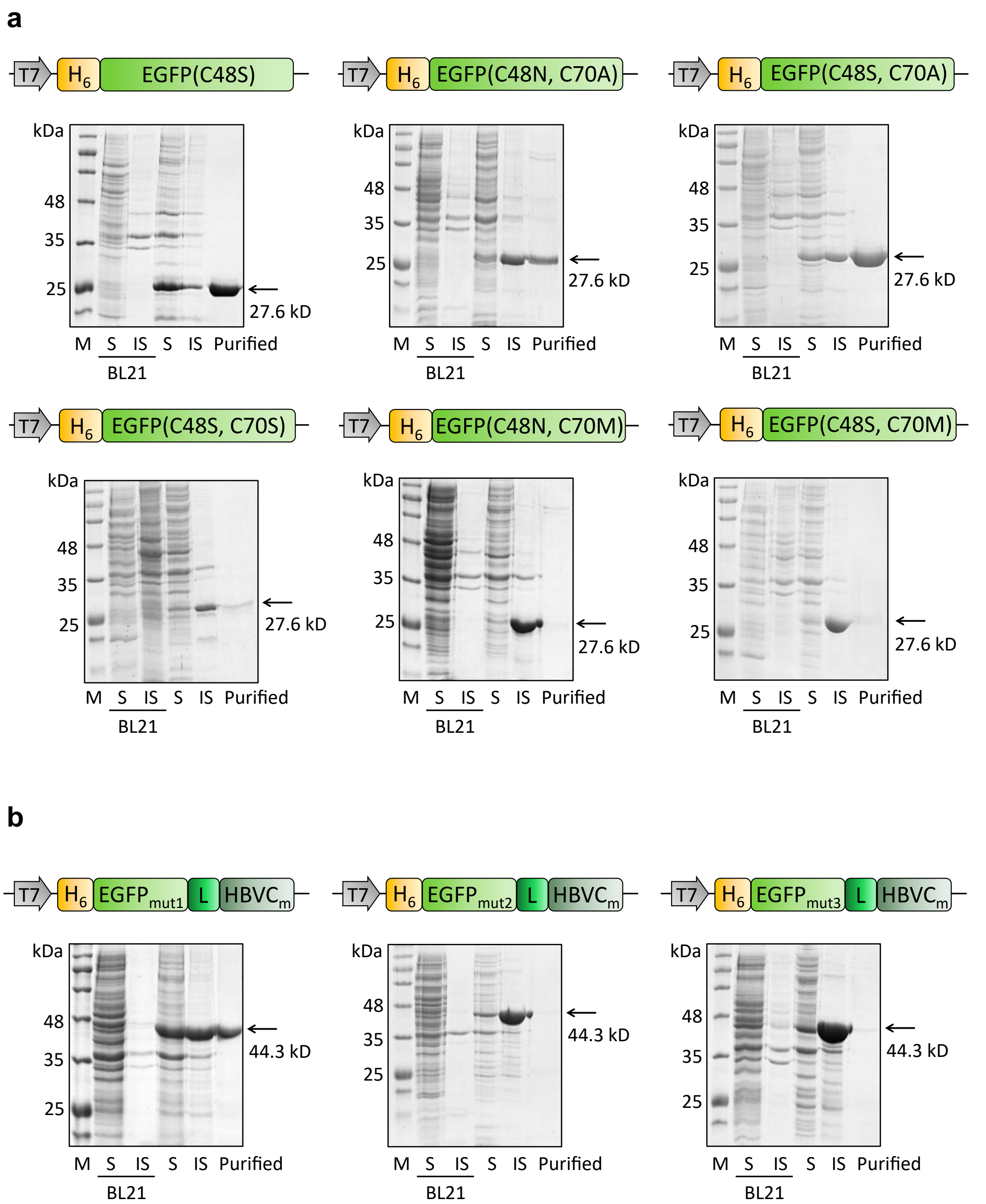

Supplementary Figure 1. Plasmid expression vectors and SDS-PAGE analysis for recombinant expression in *E. coli*. (a,b) Plasmid expression vectors encoding $EGFP_{mut}$ variants (a) and $HBVC_m$-$EGFP_{mut}$ variants (b); all constructs carry a T7 promoter. For SDS-PAGE, soluble (S) and insoluble (IS) fractions of induced *E. coli* BL21(DE3) lysates and Ni^{2+}-affinity purified protein are shown; arrows indicate the calculated molecular weight of each target protein (M: protein marker). The SDS-PAGE analysis is representative of three independent experiments with similar results. Uncropped gel images are provided in the Source Data file.

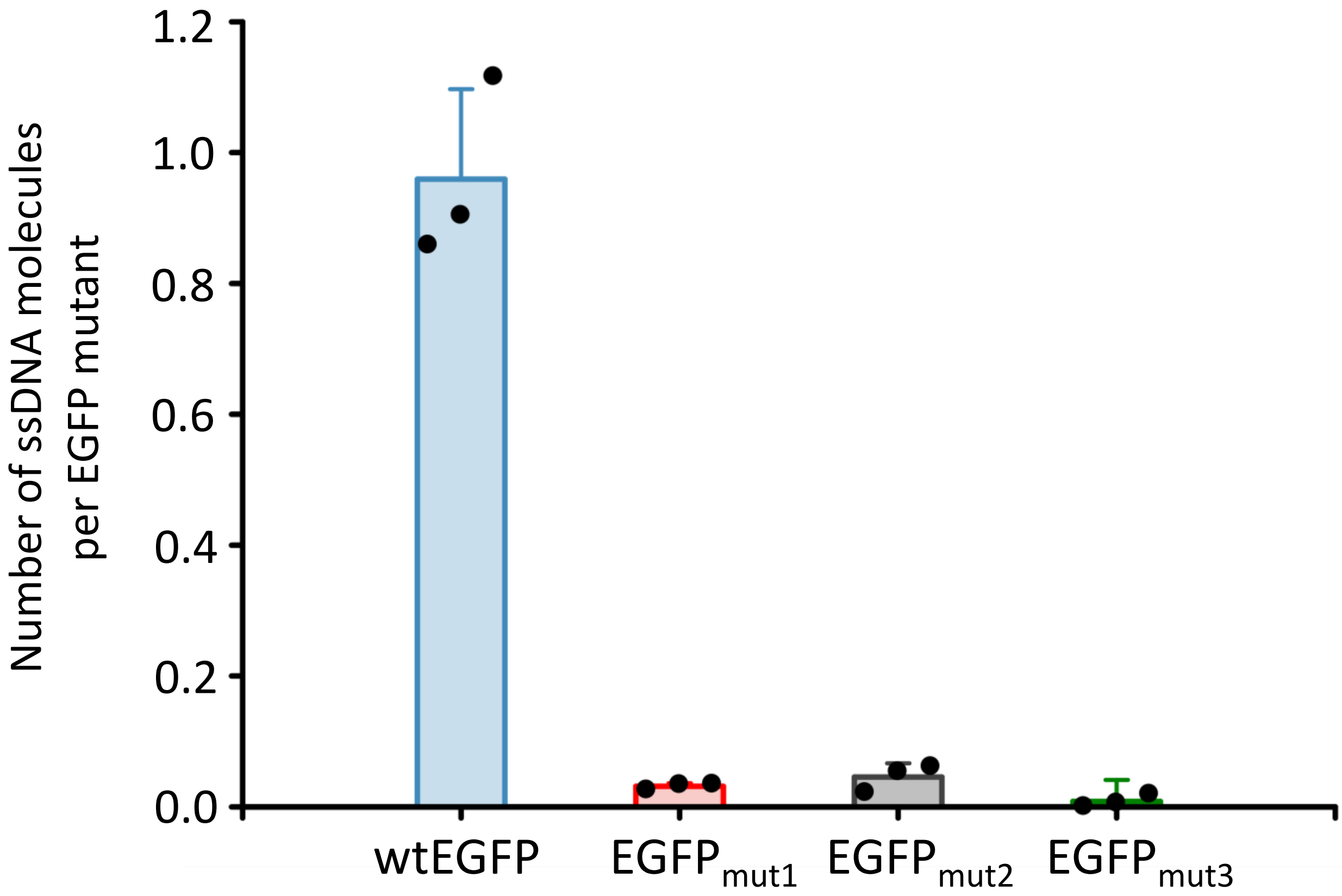

Supplementary Figure 2. Thiol reactivity of EGFP variants. Purified proteins were reduced to expose free thiols and reacted with Cy5-maleimide under identical conditions. Wild-type EGFP was labeled ~1 Cy5 molecule per protein, whereas three EGFP mutants ($EGFP_{mut1}$ to $EGFP_{mut3}$) showed < 3% of the wild-type labeling level, confirming effective removal of reactive cysteines. Bars show mean and error bars represent s.d. (n = 3 technical replicates) (wtEGFP; wild-type EGFP). Source data are provided as a Source Data file.

a

b

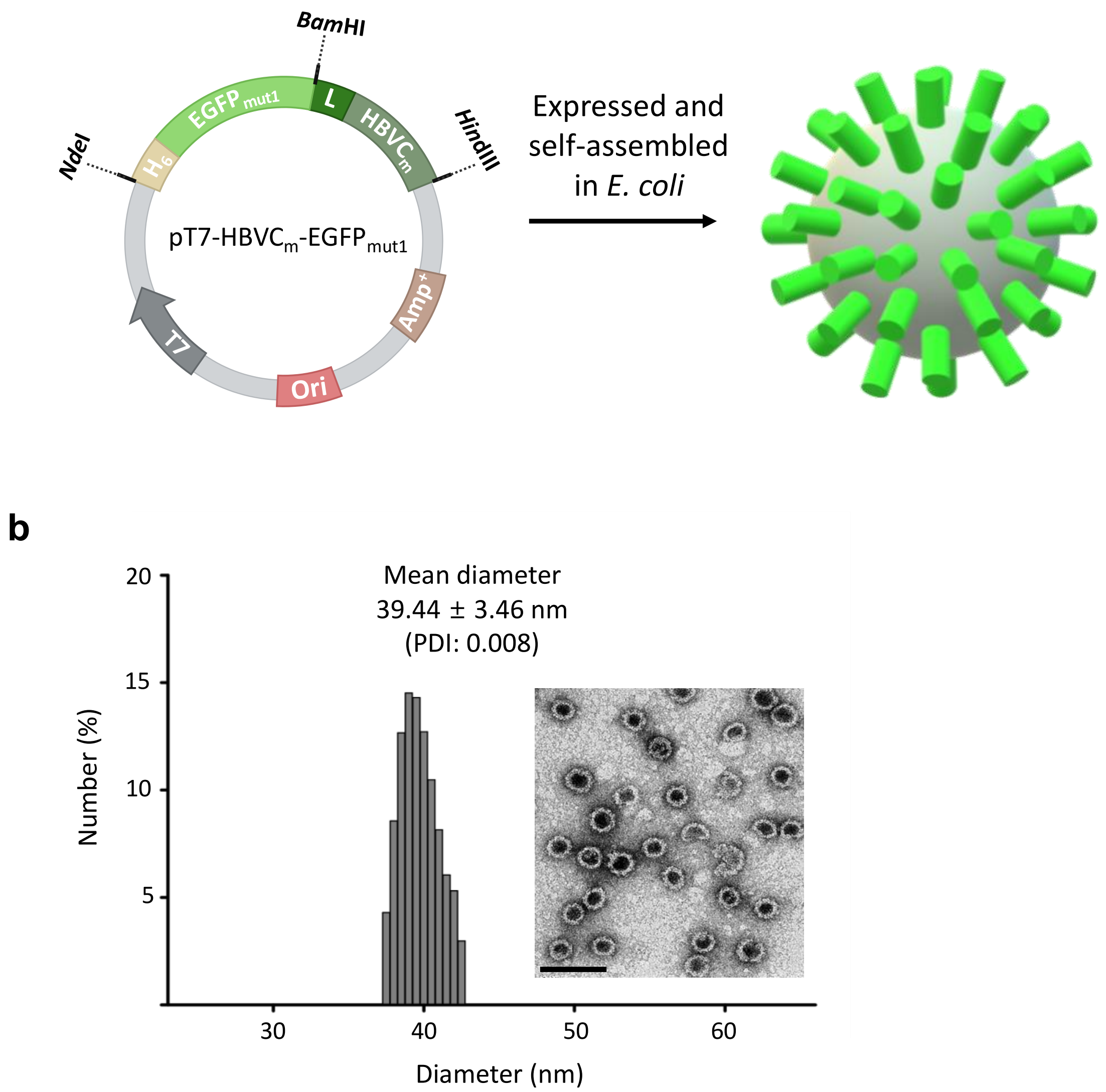

Supplementary Figure 3. Characterization of HBVC$_m$-EGFP$_{mut1}$. (a) Plasmid expression vector encoding NH$_2$-H$_6$-EGFP$_{mut1}$-G$_3$SG$_3$T-HBVC$_m$-COOH and schematic of self-assembly in *E. coli* to yield HBVC$_m$-EGFP$_{mut1}$, a protein nanocage displaying 240 EGFPs on the capsid surface. (H$_6$, hexa-histidine tag for Ni^{2+}-affinity purification; L, flexible linker peptide (G$_3$SG$_3$T)). **(b)** TEM image (scale bar, 100 nm) and DLS size distribution of purified HBVC$_m$-EGFP$_{mut1}$. The TEM and DLS analyses are representative of three independent experiments with similar results. Source data are provided as a Source Data file. (PDI, polydispersity index)

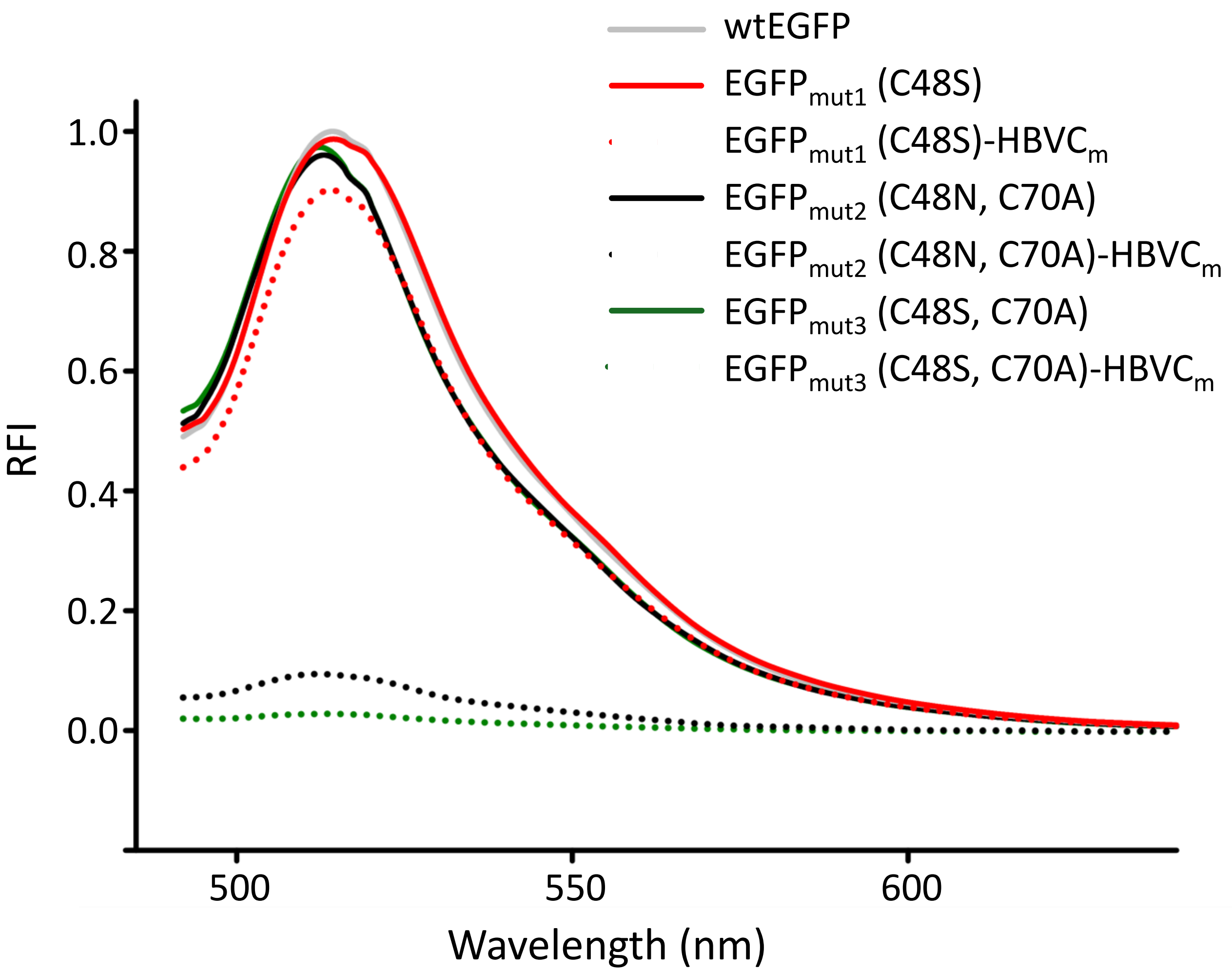

Supplementary Figure 4. Emission spectra of EGFP variants ($EGFP_{mut1}$, $EGFP_{mut2}$, and $EGFP_{mut3}$) and their $HBVC_m$ fusions. Normalized emission spectra (λ_{ex} = 488 nm) of wild-type EGFP and three EGFP mutants ($EGFP_{mut1}$ to $EGFP_{mut3}$) (solid lines) and the corresponding $HBVC_m$-$EGFP_{mut}$ fusions (dotted lines), normalized to the peak intensity of wild-type EGFP. Relative brightness at 514 nm (wild-type = 100%) is: $EGFP_{mut1}$ 98.7% and $HBVC_m$-$EGFP_{mut1}$ 90.2%, $EGFP_{mut2}$ 95.8% and $HBVC_m$-$EGFP_{mut2}$ 9.3%, $EGFP_{mut3}$ 96.7% and $HBVC_m$-$EGFP_{mut3}$ 2.8%. Spectra are representative of three independent experiments. Source data are provided as a Source Data file.

a

```
                                   1
1                                                                  40
VSKGEELFTG VVPILVELDG DVNGHKFSVS GEGEGDATYG

            2  3 4
41                                                                 80
KLTLKFISTT GKLPVPWPTL VTTLTYGVQC FSRYPDHMKQ

                                5 6 7 8 9
81                                                                120
HDFFKSAMPE GYVQERTIFF KDDGNYKTRA EVKFEGDTLV

            10  11  12 13 14 15 16 17  18  19
121                                                               160
NRIELKGIDF KEDGNILGHK LEYNYNSHNV YIMADKQKNG

               20  21 22
161                                                               200
IKVNFKIRHN IEDGSVQLAD HYQQNTPIGD GPVLLPDNHY

          23  24  25 26 27 28
201                                                       238
LSTQSALSKD PNEKRDHMVL LEFVTAAGIT LGMDELYK
```

b

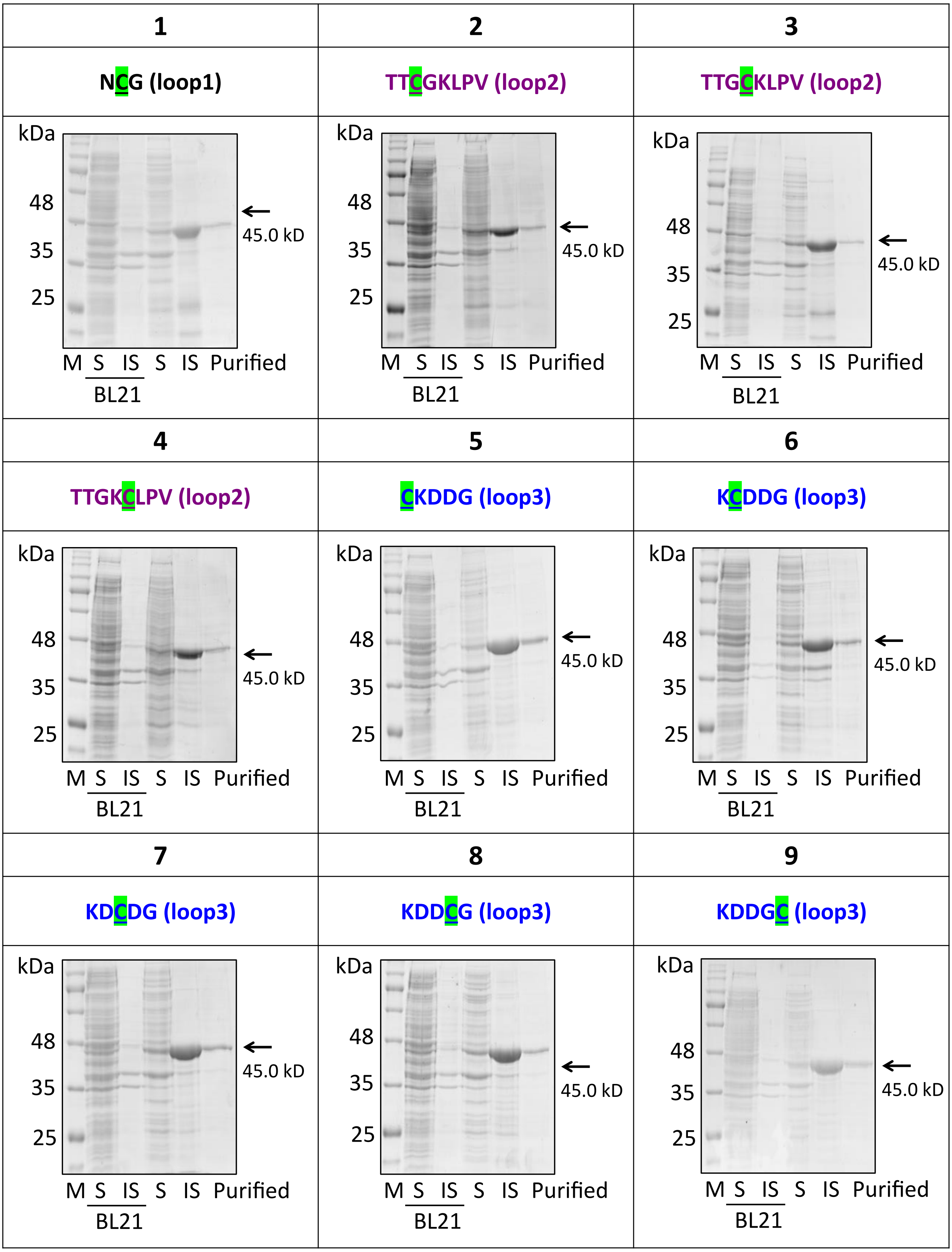
1
NCG (loop1)
2
TTCGKLPV (loop2)
3
TTGCKLPV (loop2)
4
TTGKCLPV (loop2)
5
CKDDG (loop3)
6
KCDDG (loop3)
7
KDCDG (loop3)
8
KDDCG (loop3)
9
KDDGC (loop3)
kDa
48
35
25
45.0 kD
M S IS S IS Purified
BL21

10

DFCKEDGNILGHKLEYNYNSH (loop4)

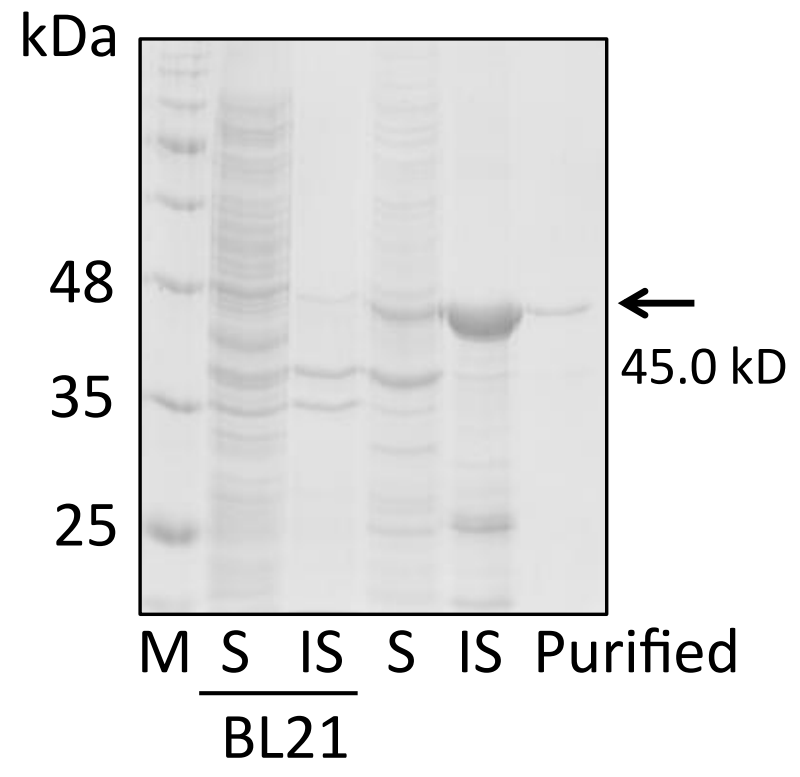

11

DFKCEDGNILGHKLEYNYNSH (loop4)

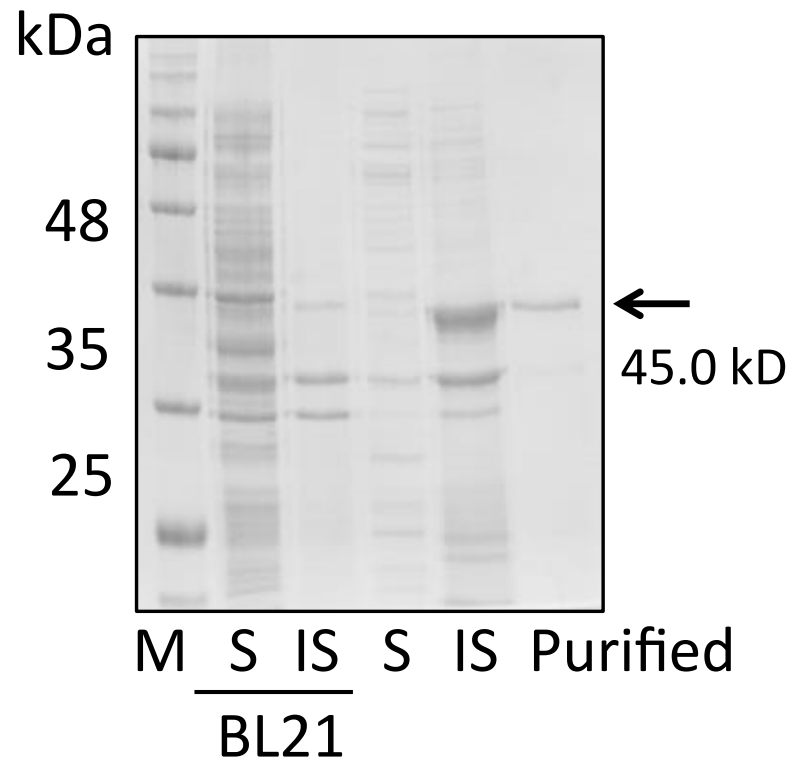

12

DFKECDGNILGHKLEYNYNSH (loop4)

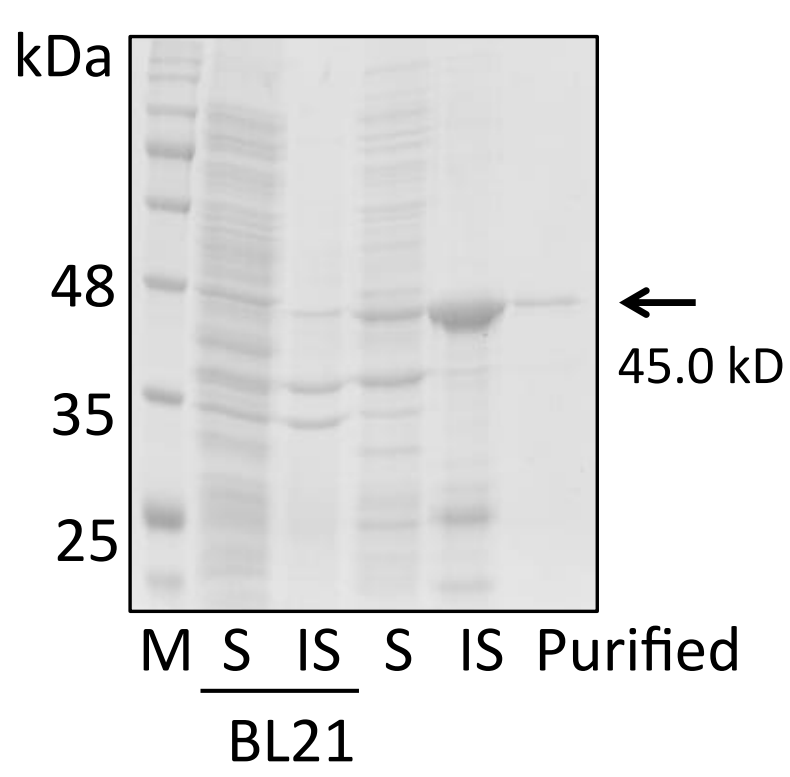

13

DFKEDGCNILGHKLEYNYNSH (loop4)

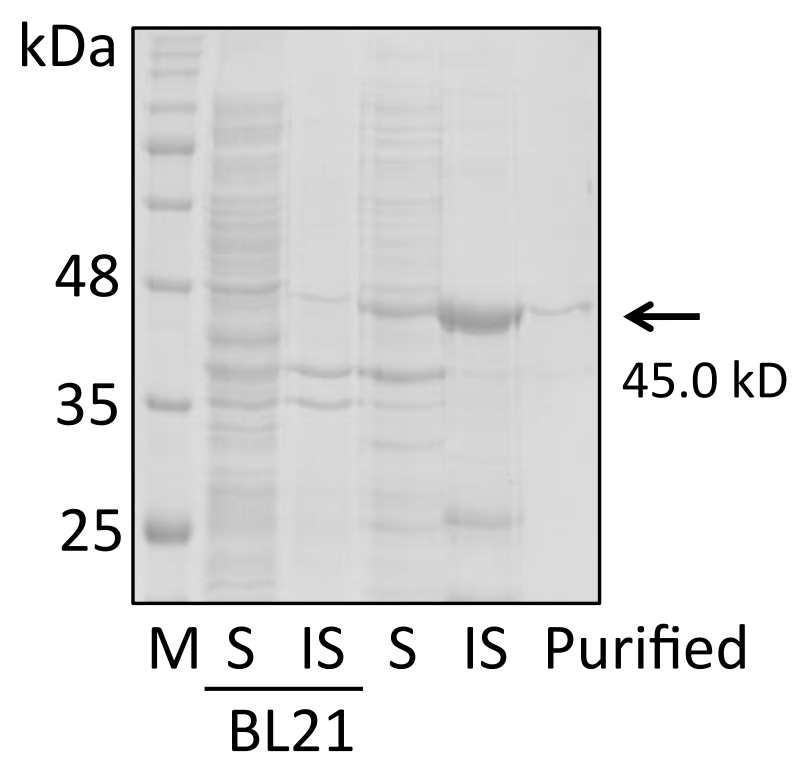

14

DFKEDGNCILGHKLEYNYNSH (loop4)

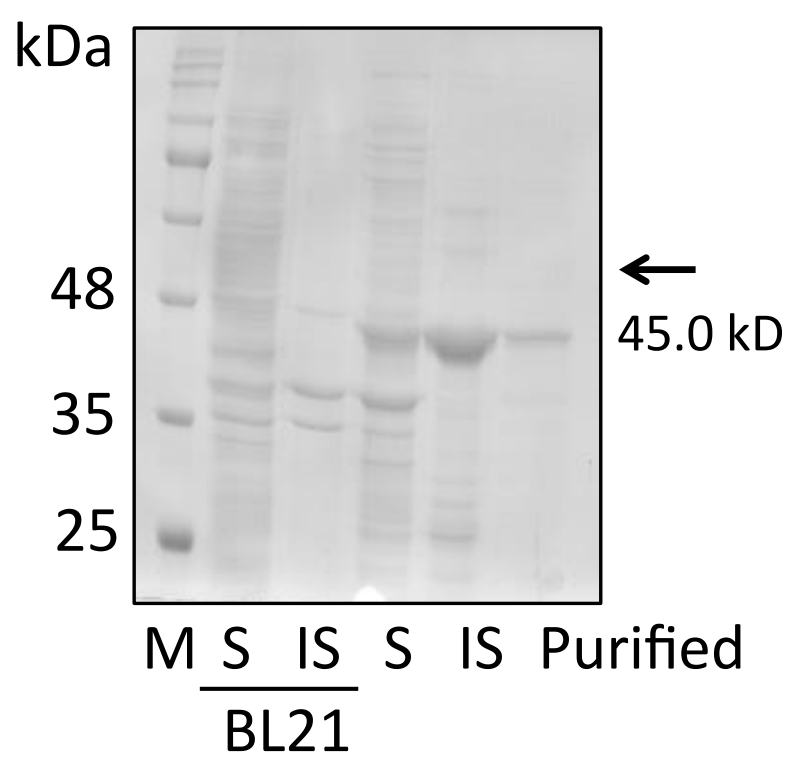

15

DFKEDGNILCGHKLEYNYNSH (loop4)

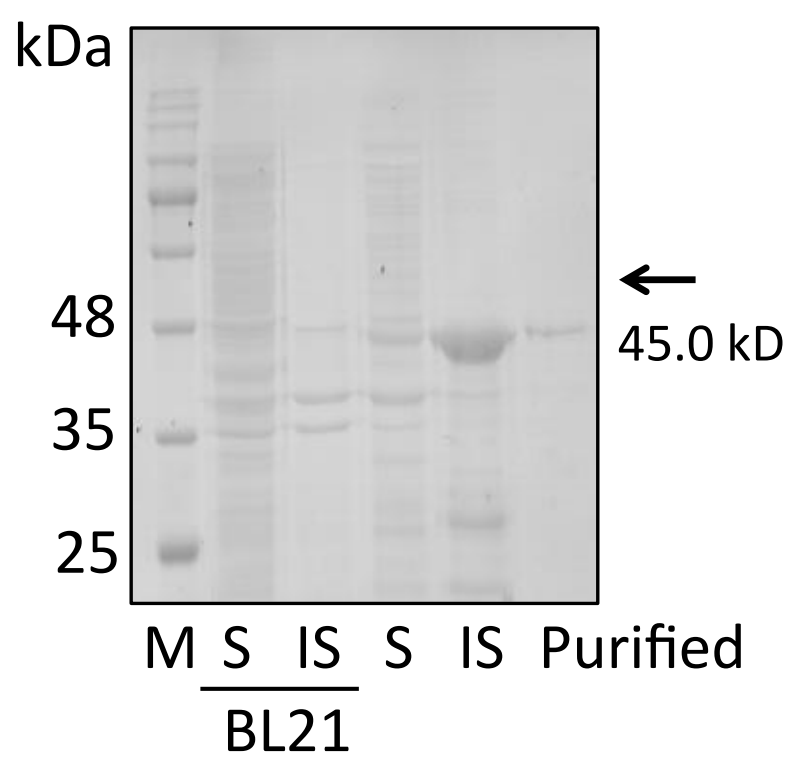

16

DFKEDGNILGCHKLEYNYNSH (loop4)

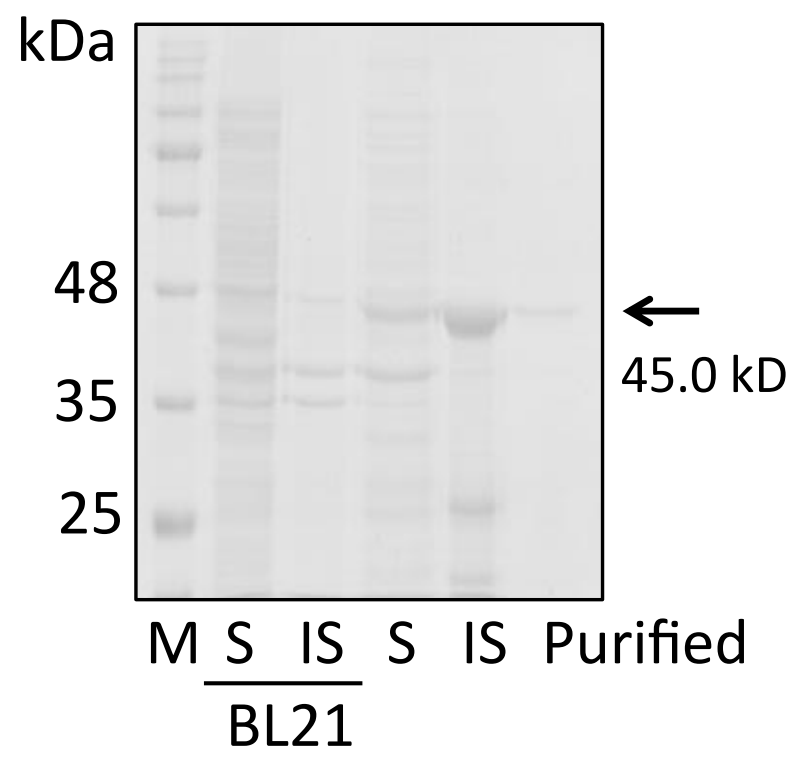

17

DFKEDGNILGHCKLEYNYNSH (loop4)

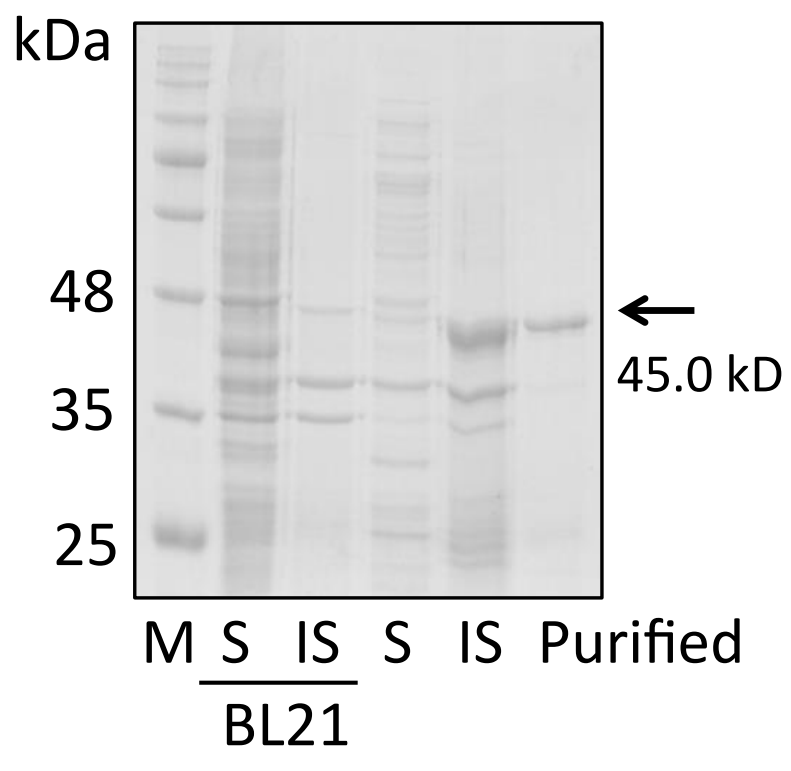

18

DFKEDGNILGHKCLEYNYNSH (loop4)

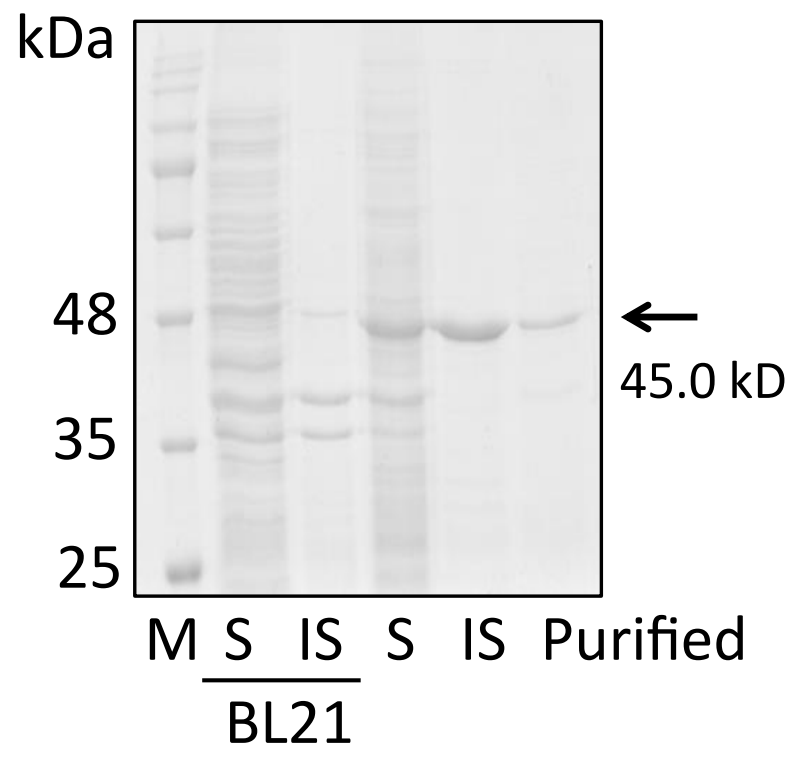

19

DFKEDGNILGHKLECYNYNSH (loop4)

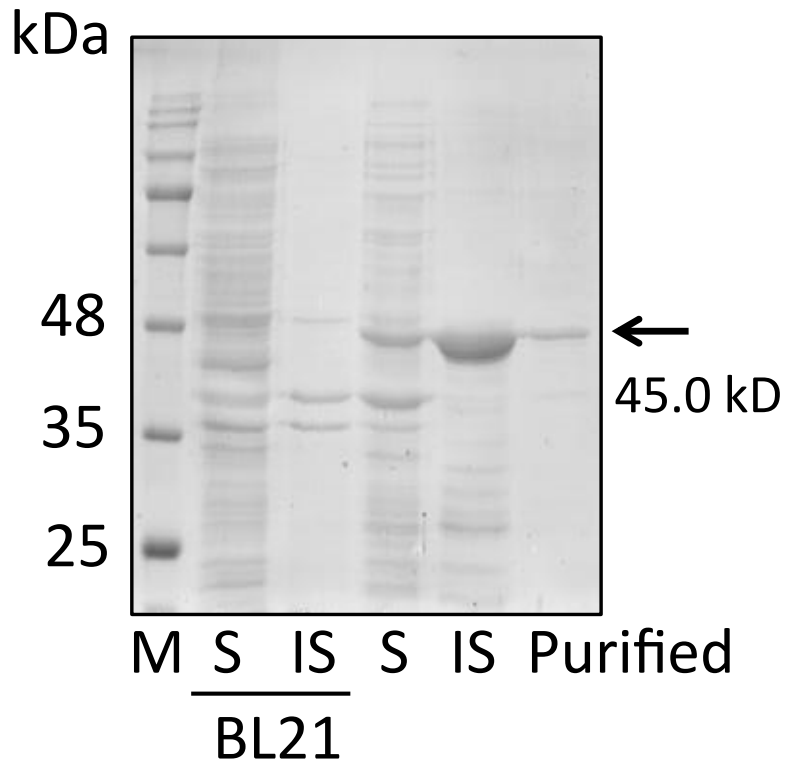

20

ICEDGS (loop5)

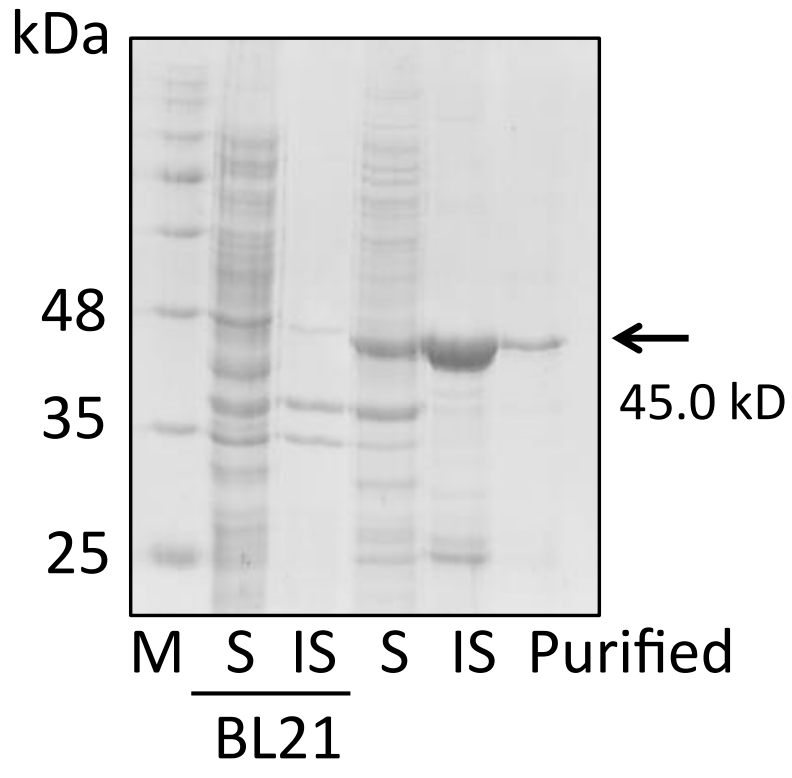

21

IEDCGS (loop5)

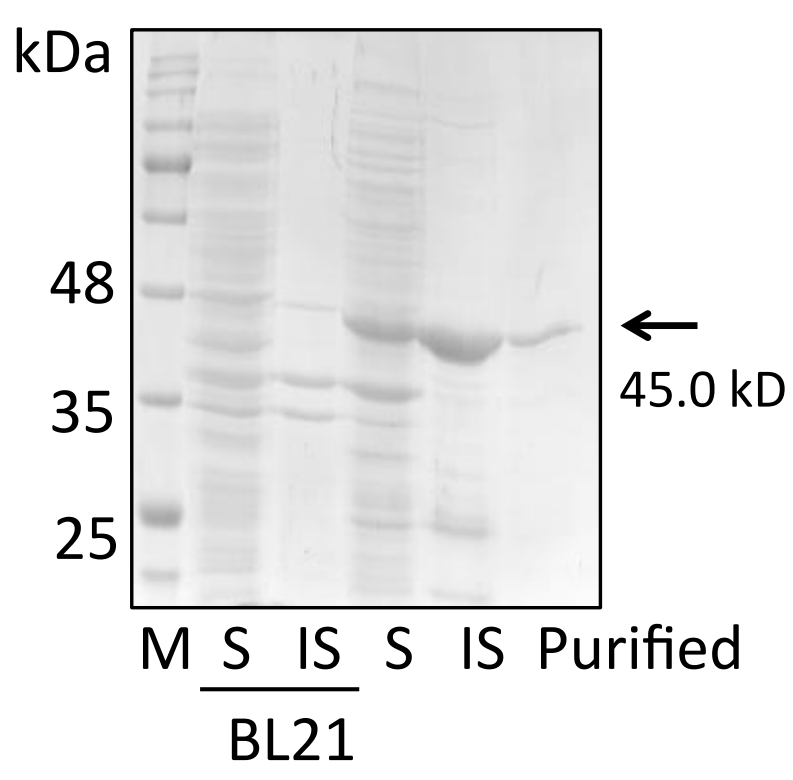

22

IEDGCS (loop5)

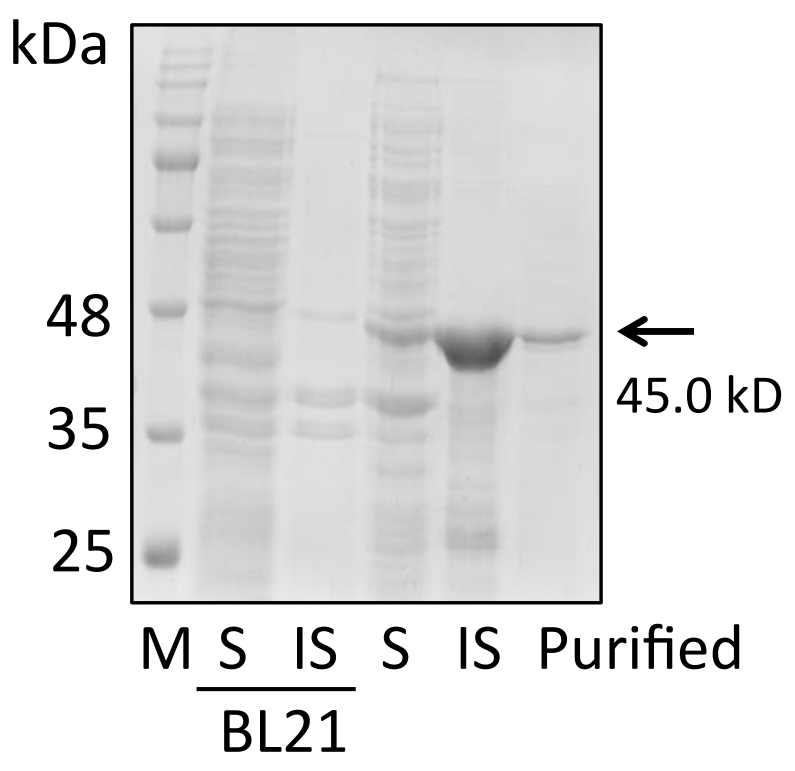

23

CKDPNEKRD (loop6)

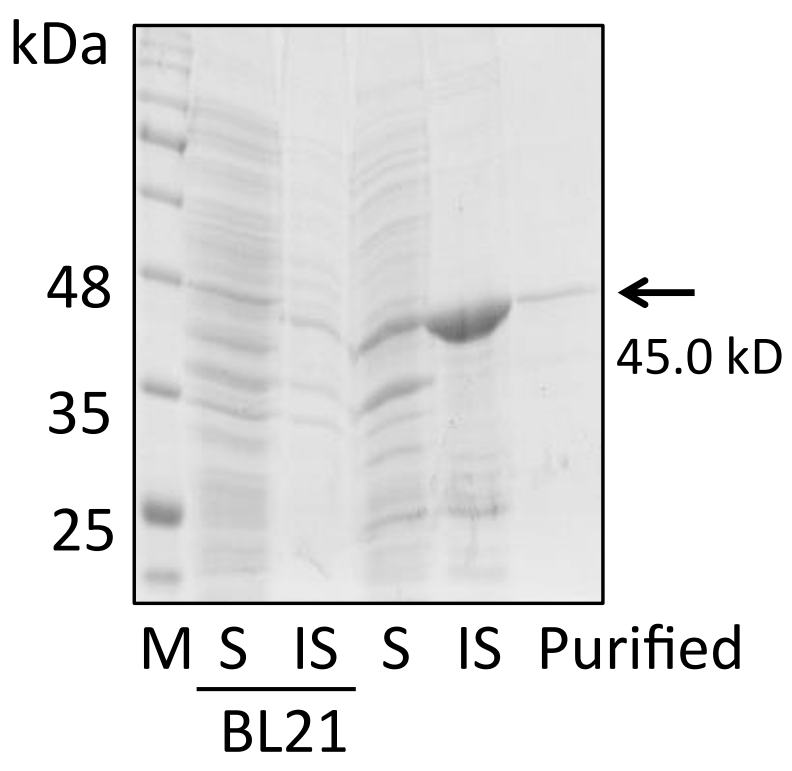

24

KDCPNEKRD (loop6)

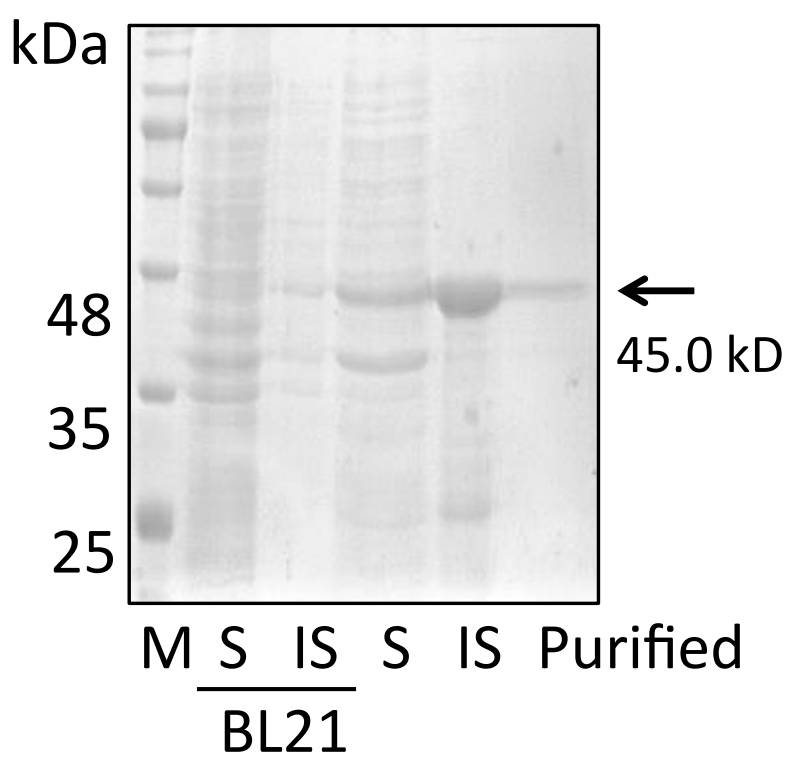

25

KDPCNEKRD (loop6)

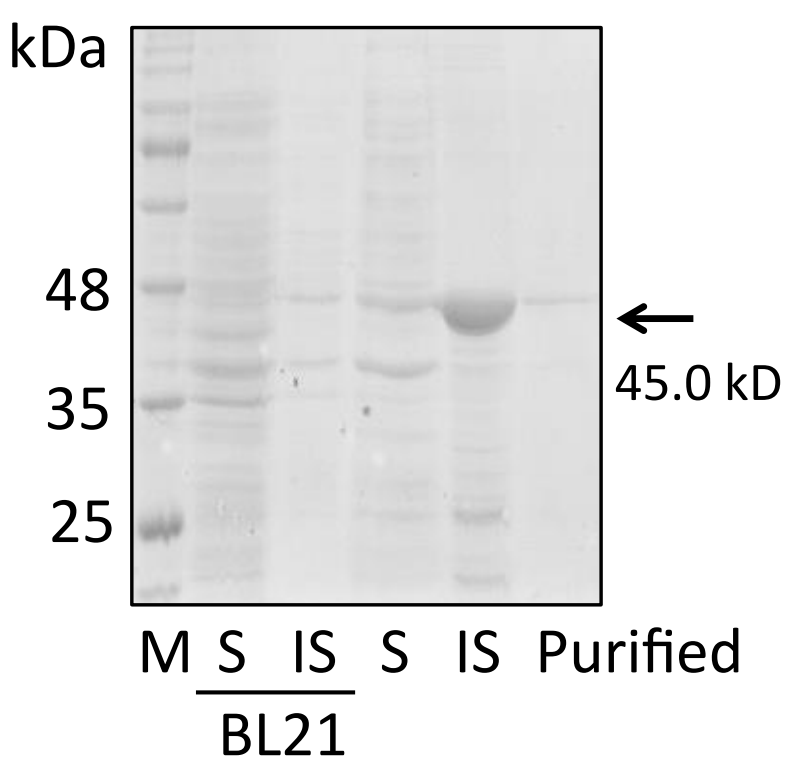

26

KDPNCEKRD (loop6)

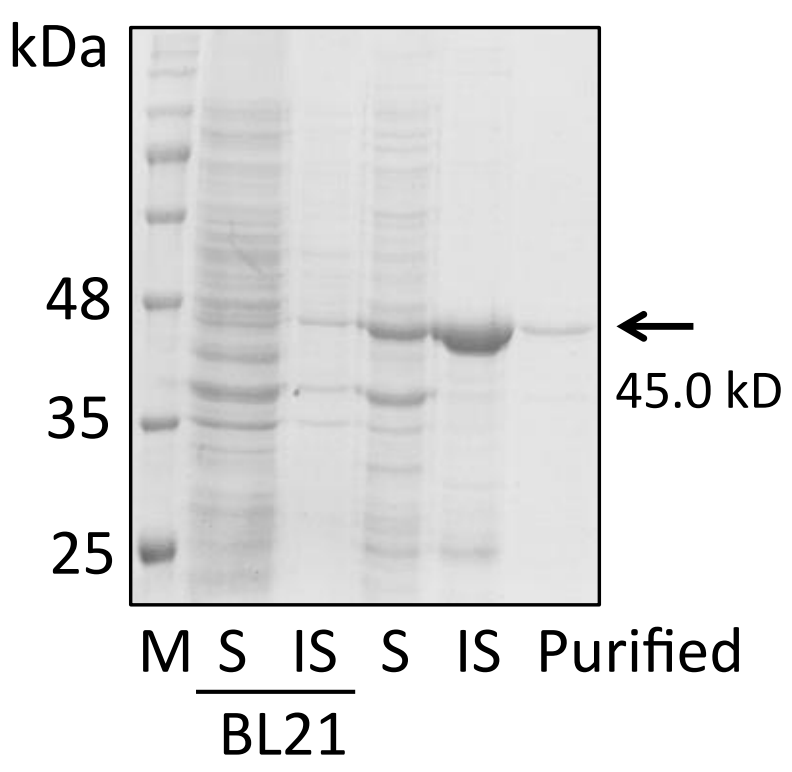

27

KDPNECKRD (loop6)

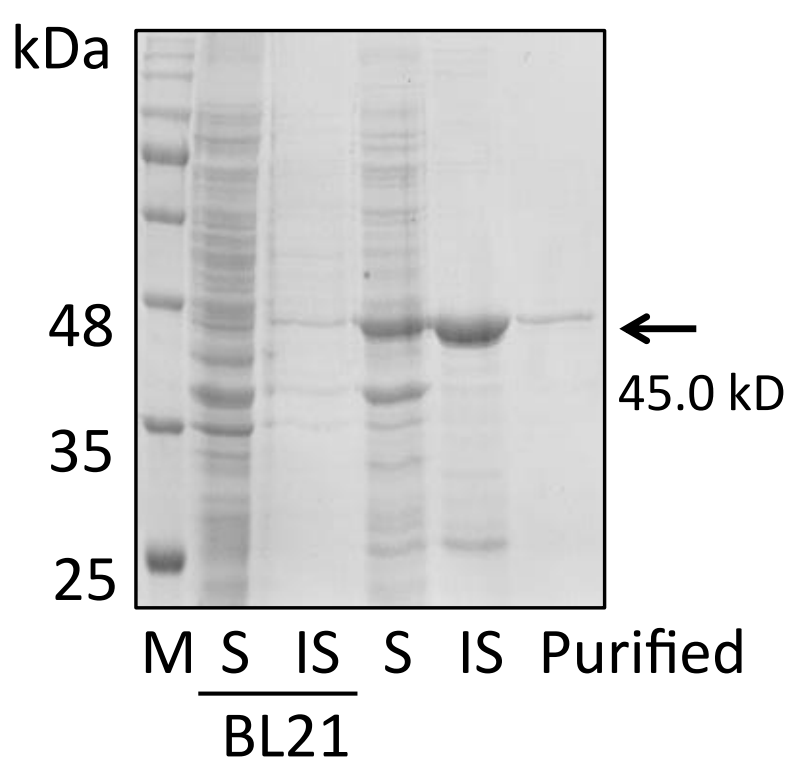

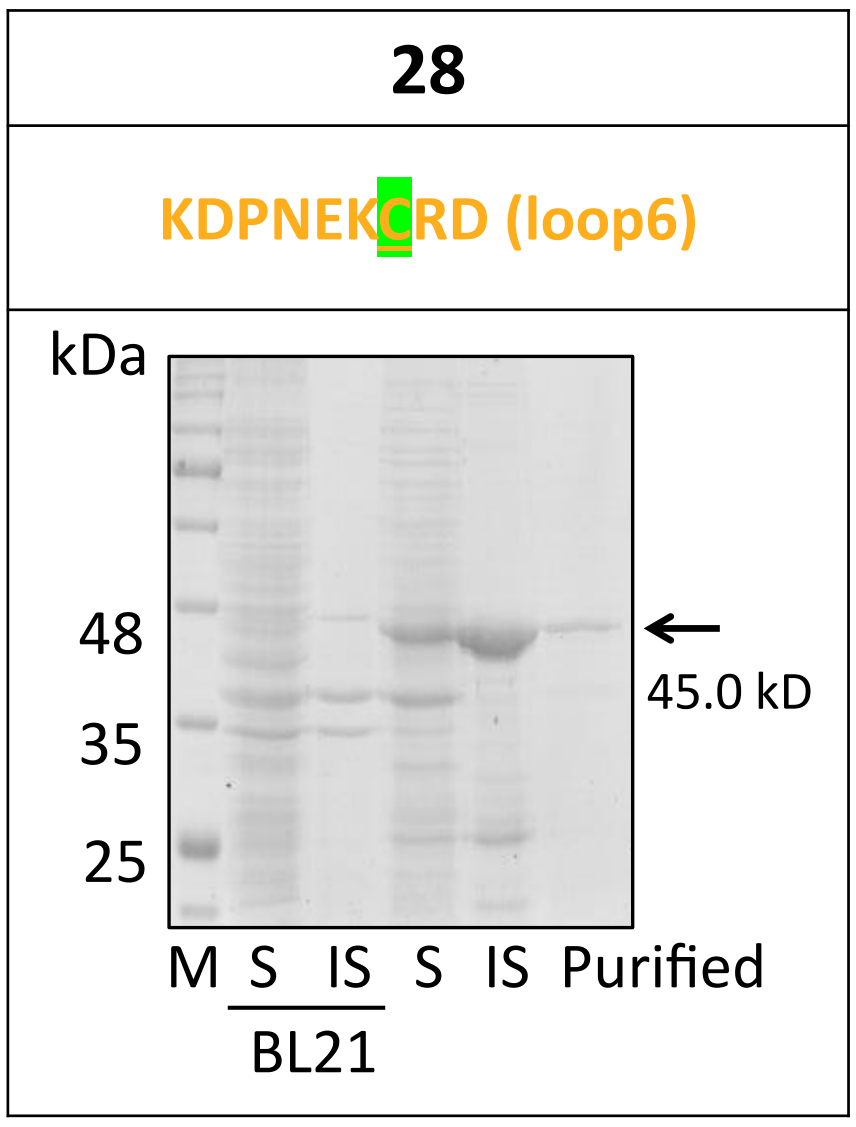
28
KDPNEKCRD (loop6)
kDa
48
35
25
45.0 kD
M S IS S IS Purified
BL21

c

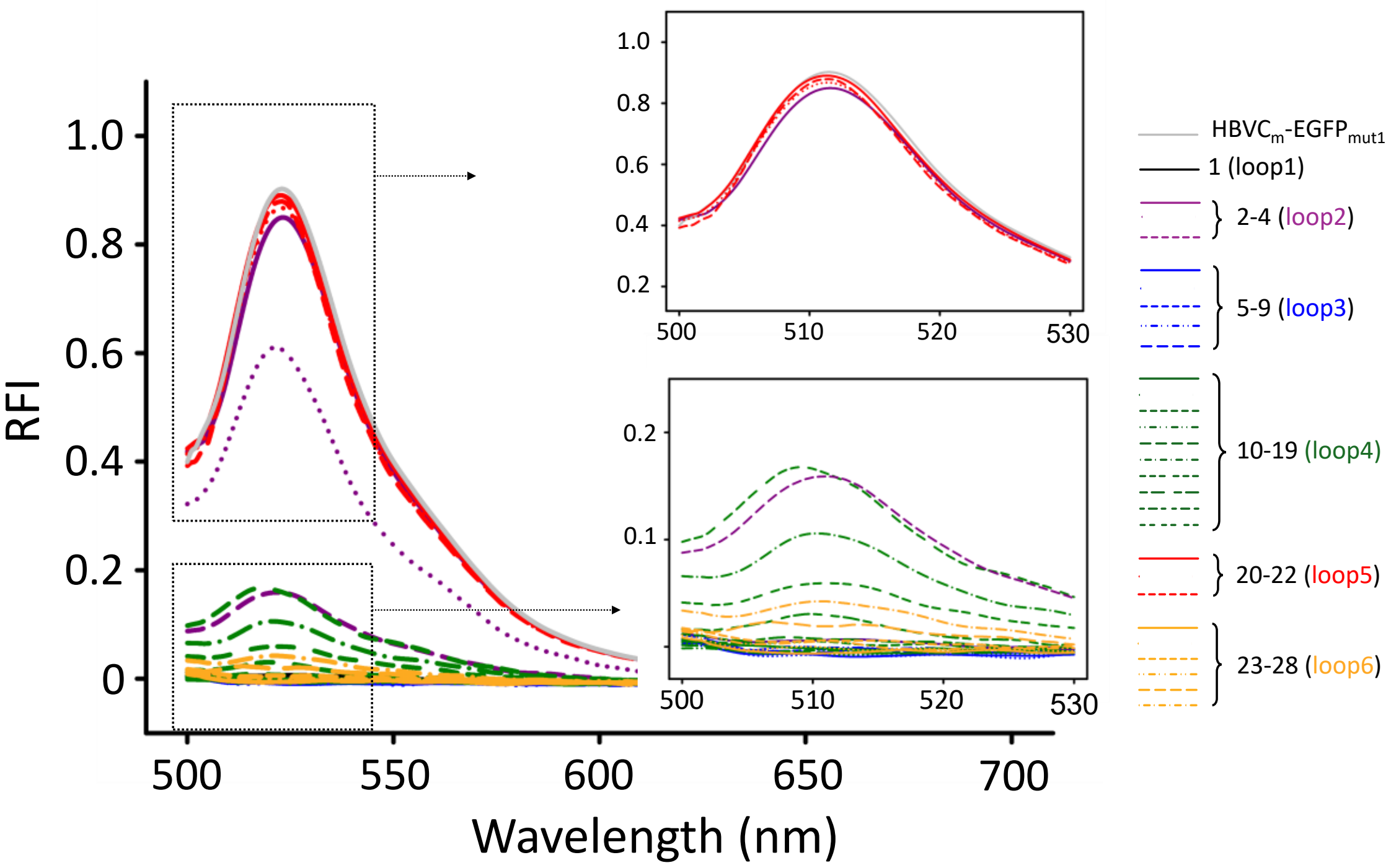
RFI
1.0
0.8
0.6
0.4
0.2
0
500
550
600
650
700
Wavelength (nm)
510
520
530
0.1
HBVCm-EGFPmut1
1 (loop1)
2-4 (loop2)
5-9 (loop3)
10-19 (loop4)
20-22 (loop5)
23-28 (loop6)

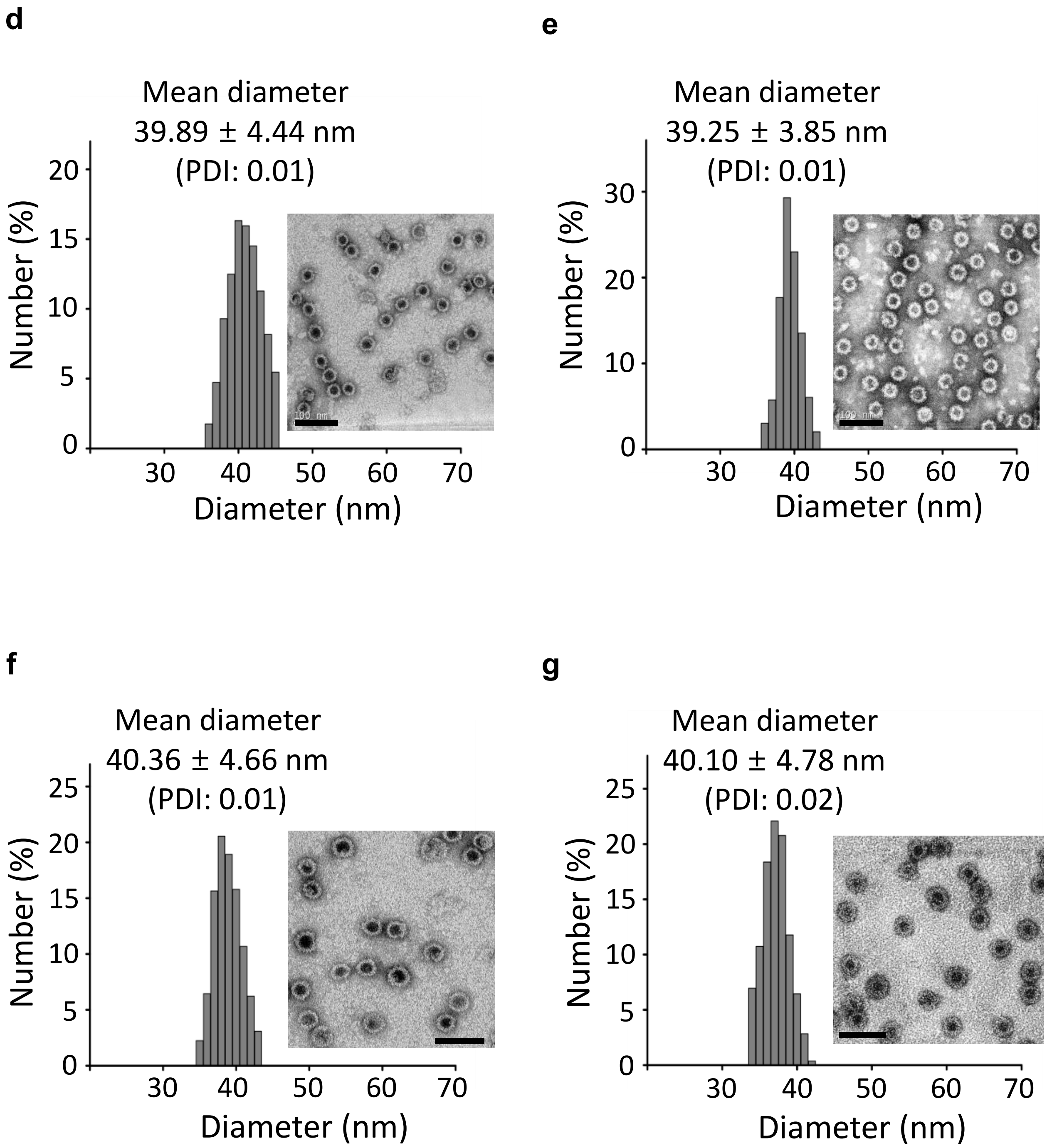
d
Mean diameter
39.89 ± 4.44 nm
(PDI: 0.01)
Number (%)
Diameter (nm)
e
Mean diameter
39.25 ± 3.85 nm
(PDI: 0.01)
Number (%)
Diameter (nm)
f
Mean diameter
40.36 ± 4.66 nm
(PDI: 0.01)
Number (%)
Diameter (nm)
g
Mean diameter
40.10 ± 4.78 nm
(PDI: 0.02)
Number (%)
Diameter (nm)

Supplementary Figure 5. Mapping and screening of single-cysteine insertion in HBVC$_m$-EGFP$_{mut1}$. (a) The full 238-residue EGFP$_{mut1}$ sequence is shown in grey. Six surface loops selected for cysteine insertion are color-coded and underlined: loop 1 (black), loop 2 (purple), loop 3 (blue), loop 4 (green), loop 5 (red) and loop 6 (orange). Arrowheads indicate the 28 individual insertion sites (1–28). **(b)** SDS-PAGE of soluble (S) and insoluble (IS) fractions of HBVC$_m$-EGFP$_{mut1}$ variant-expressing *E. coli* cells and Ni^{2+}-affinity purified HBVC$_m$ (M: protein marker). Panels are grouped by loop; arrows indicate the expected ~45 kDa band. Only variant 2, variant 20, variant 21, and variant 22 show soluble expressions comparable to HBVCm-EGFP$_{mut1}$. The SDS-PAGE analysis is representative of three independent experiments with similar results. Uncropped gel images are provided in the Source Data file. **(c)** Emission spectra (λ_{ex} = 488 nm; $\lambda_{em,\ max}$ = 514 nm). The upper inset highlights variants 2, 20, 21, and 22 (≥ 80% of the wild-type integrated emission), whereas the remaining variants show ≤ 20% emission (lower inset). **(d-g)** DLS and TEM images for the HBVC$_m$-EGFP$_{mut1}$ variant 2 (d), variant 20 (e), variant 21 (f), and variant 22 (g). All variants are monodisperse (mean diameter = 39–40 nm; PDI ≤ 0.02) and form intact spherical particles (TEM scale bar, 100 nm). The TEM and DLS analyses are representative of three independent experiments with similar results. Source data are provided as a Source Data file.

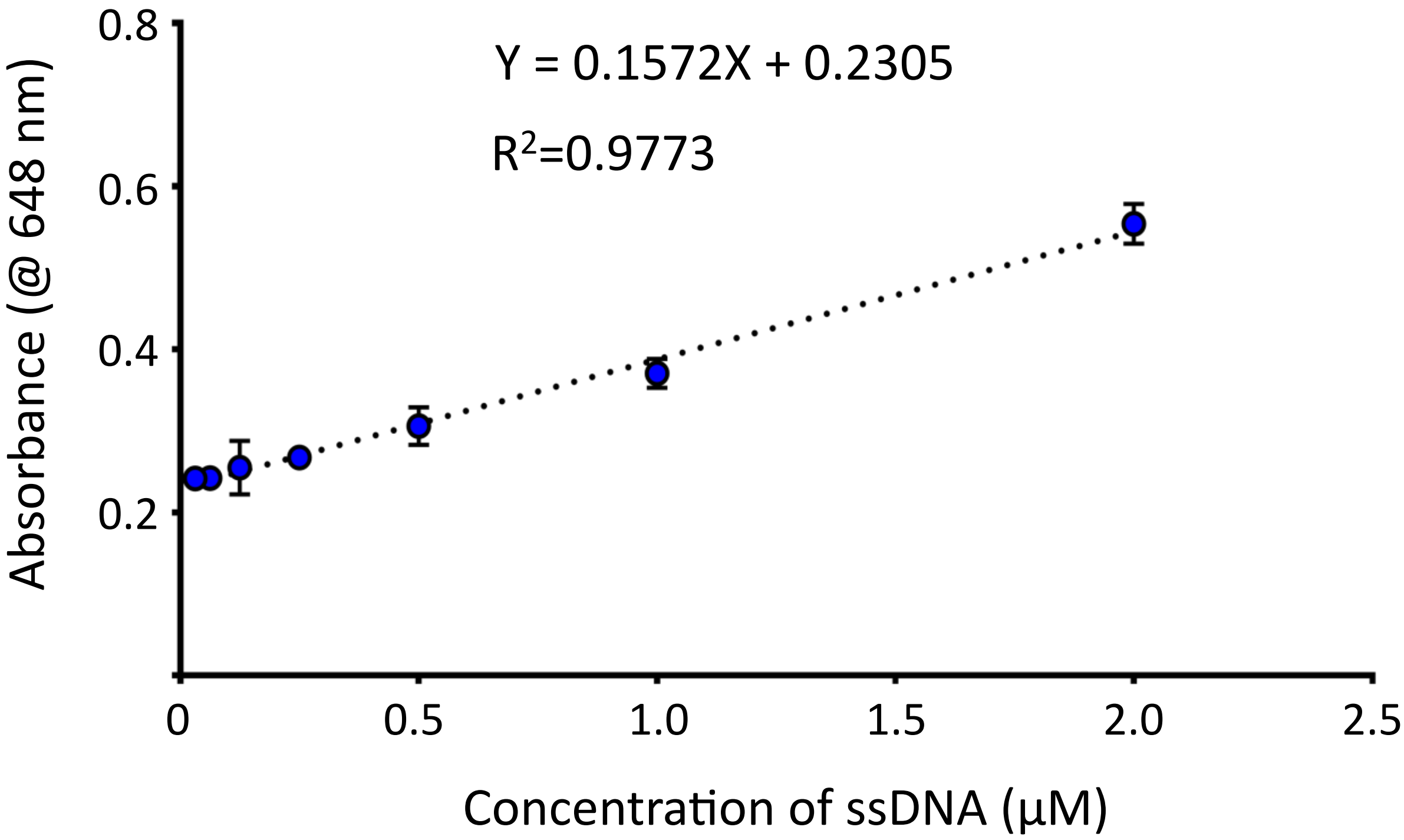

Supplementary Figure 6. Calibration curve for Cy5-labeled ssDNA. Serial dilutions of 3′-maleimide-ssDNA–Cy5 were prepared in capsid purification buffer and absorbance at 649 nm was measured. Data (blue circles) were fit by linear regression ($R^2 = 0.9773$); this calibration was used to quantify the number of ssDNA probes conjugated to $EGFP_{mut1}$ and $HBVC_m$-$EGFP_{mut1}$ variants. Error bars represent s.d. (n = 3 technical replicates). Source data are provided as a Source Data file.

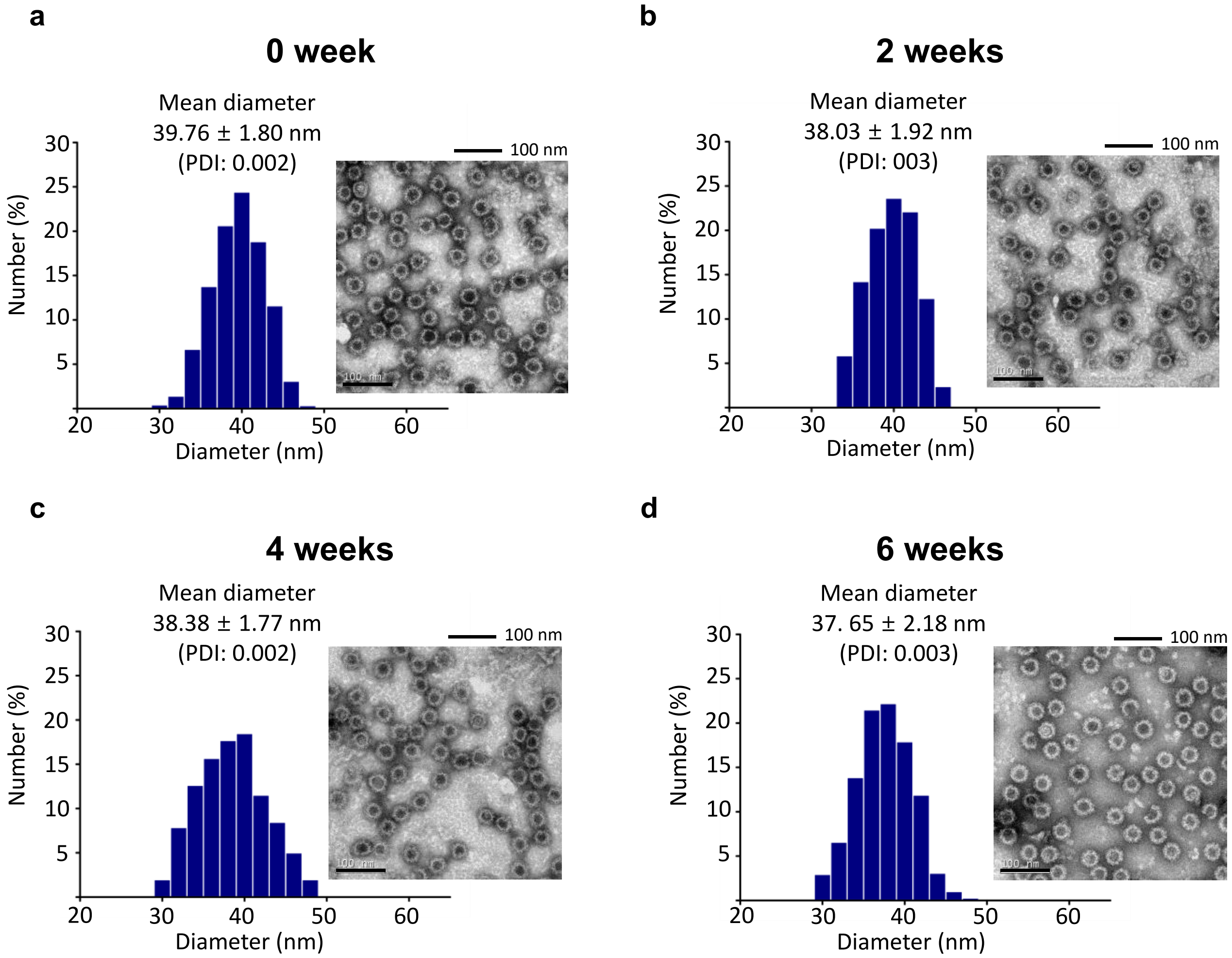

Supplementary Figure 7. Structural stability of the nanocage during frozen storage. DLS size distribution (left) and TEM image (right) of the assay-ready nanocage stored at −80 °C for **(a)** 0, **(b)** 2, **(c)** 4, and **(d)** 6 weeks. DLS showed mean diameters of 39.76 ± 1.80, 38.03 ± 1.92, 38.38 ± 1.77, and 37.65 ± 2.18 nm at 0, 2, 4, and 6 weeks, respectively, with consistently low PDI values (0.002–0.003). TEM images confirmed intact spherical morphology without detectable aggregation (scale bar, 100 nm). The TEM and DLS analyses are representative of three independent experiments with similar results. Source data are provided as a Source Data file.

a

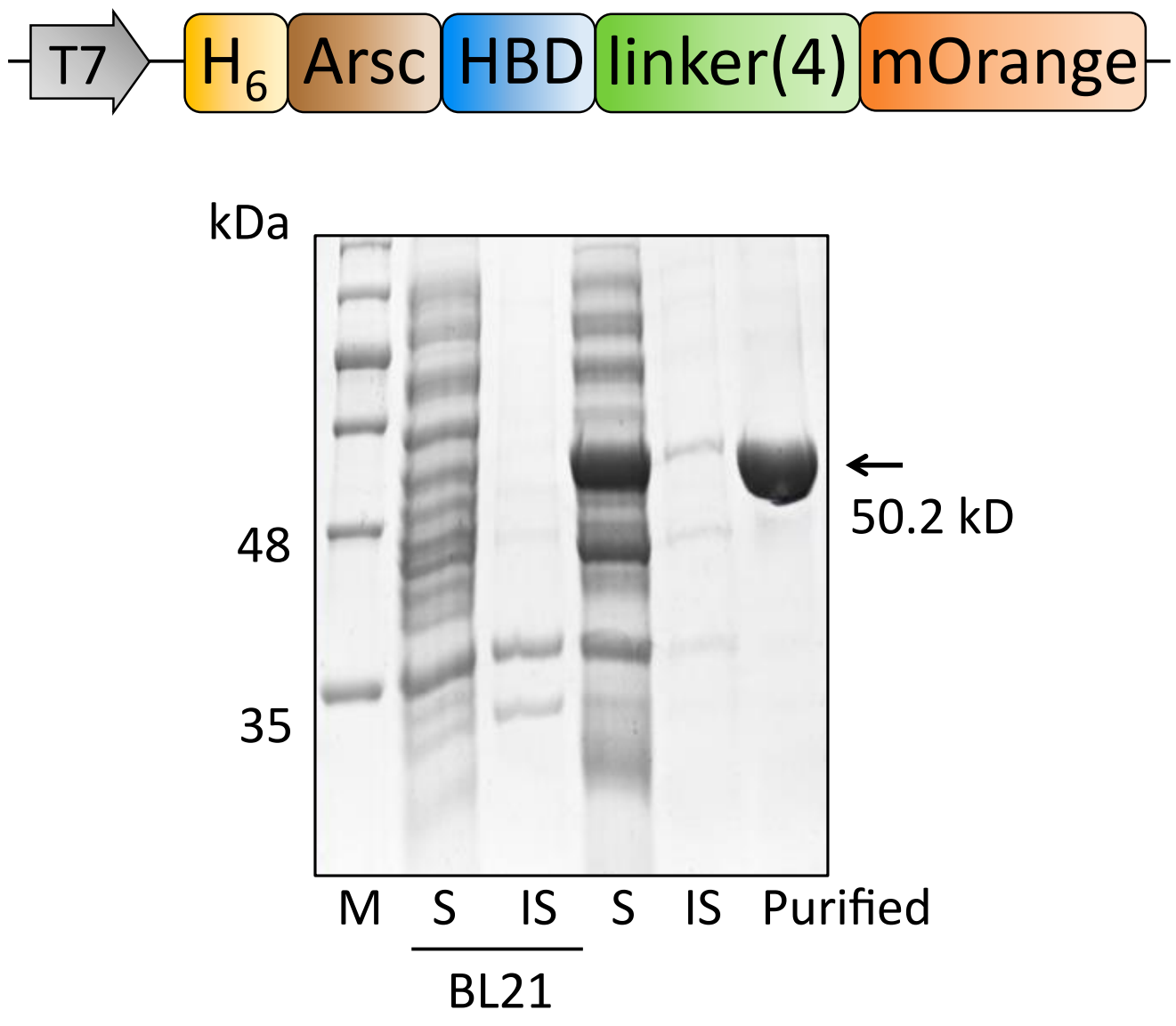

b

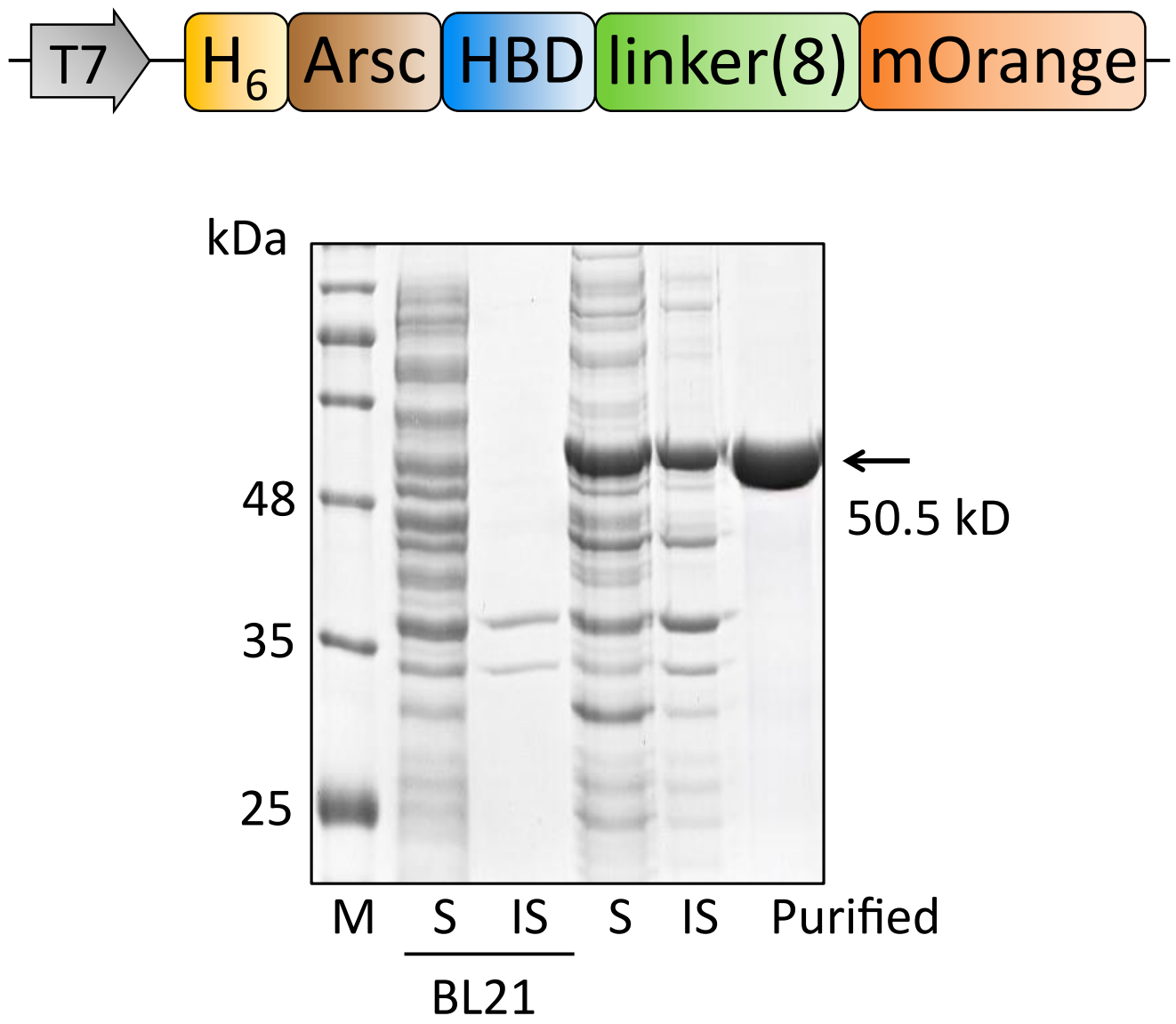

Supplementary Figure 8. Expression and purification of RNaseH1-linked acceptors with different linker lengths. (a,b) Plasmid expression vectors encoding acceptors bearing a four-residue linker (GGSG) (a), or an eight-residue linker (GGGSGGGS) (b) between RNaseH1 HBD and mOrange; all constructs carry a T7 promoter. SDS-PAGE shows soluble (S) and insoluble (IS) fractions of induced *E. coli* BL21(DE3) lysates and Ni^{2+}-affinity purified proteins; arrows indicate the calculated molecular weight of each target protein (M, protein marker). The SDS-PAGE analysis is representative of three independent experiments with similar results. Uncropped gel images are provided in the Source Data file.

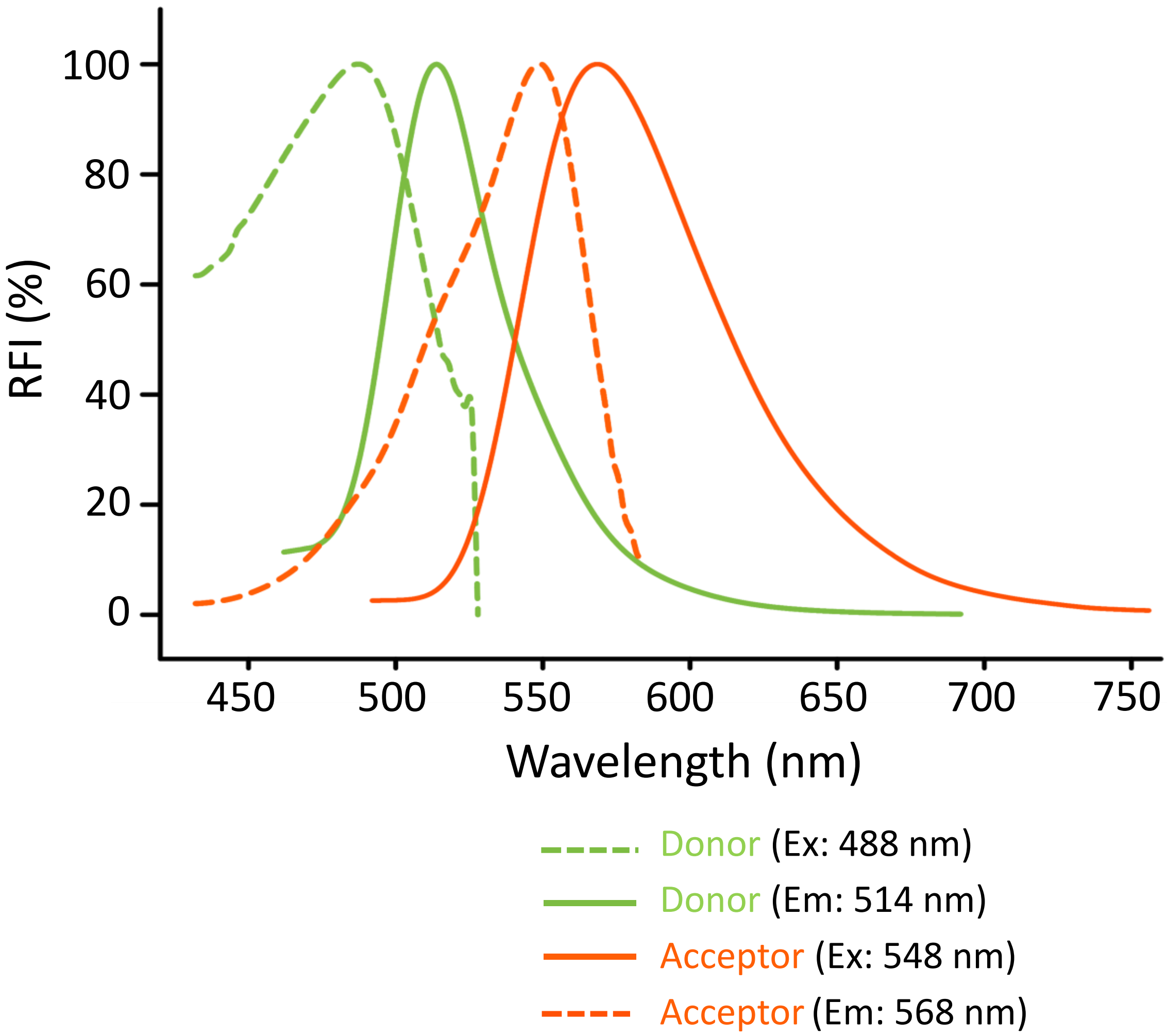

Supplementary Figure 9. Fluorescence spectra of the FRET donor and acceptor. Normalized spectra show that donor emission (λ_{ex} = 488 nm, $\lambda_{em,max}$ = 514 nm; green) overlaps the acceptor excitation band (λ_{ex} = 548 nm, $\lambda_{em,max}$ = 568 nm; orange), satisfying the Förster criterion for efficient energy transfer. Spectra are representative of three independent experiments. Source data are provided as a Source Data file.

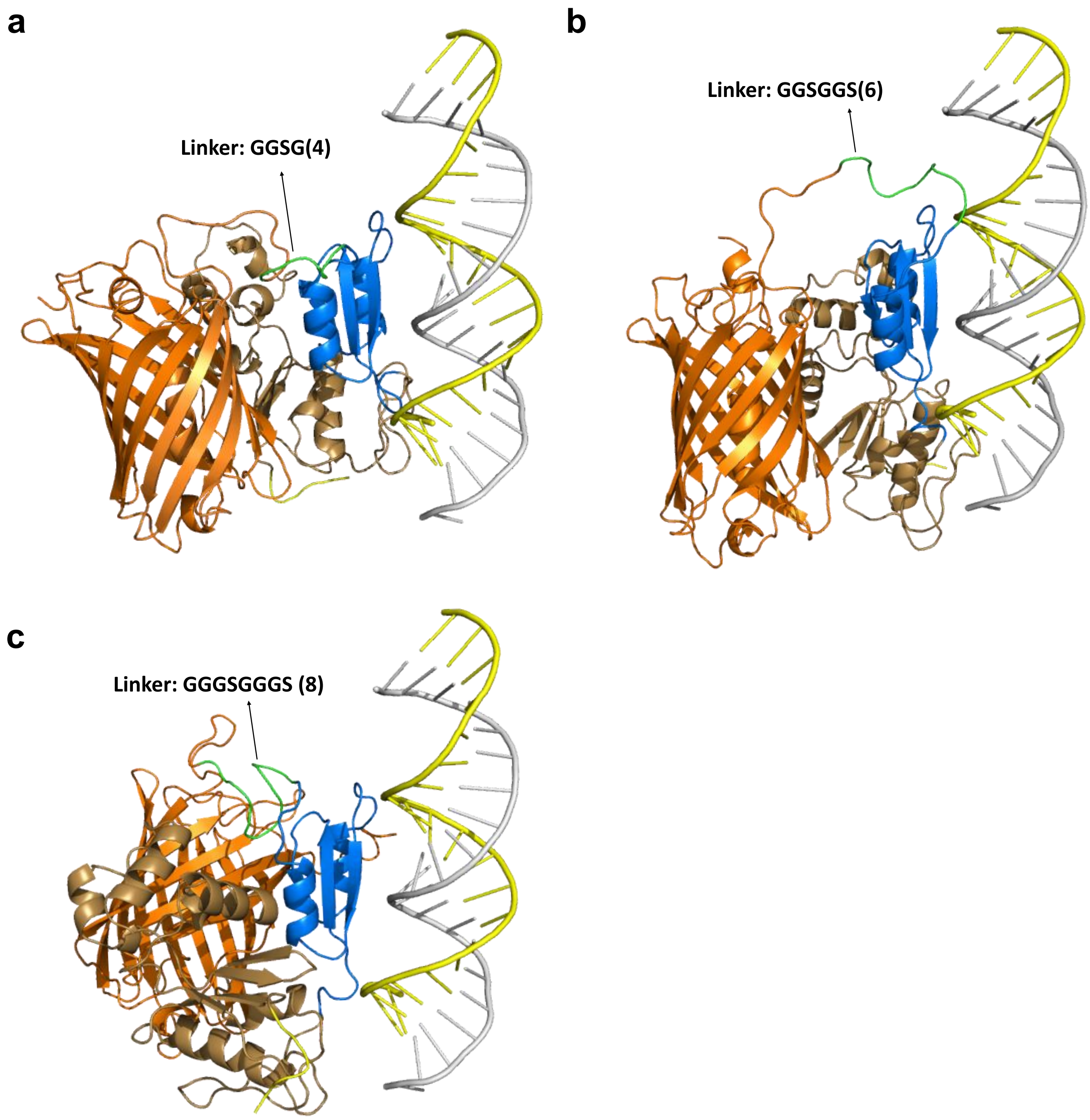

Supplementary Figure 10. MD simulations of the RNaseH1-linked acceptor binding to a miRNA-ssDNA hybrid with different linker lengths. Representative structures after 100 ns explicit-solvent MD simulations show the acceptor bound to the let-7a-ssDNA hybrid for linkers of **(a)** a four-residue linker (GGSG), **(b)** a six-residue linker (GGSGGS), and **(c)** an eight-residue linker (GGGSGGGS) inserted between RNaseH1 HBD and mOrange.

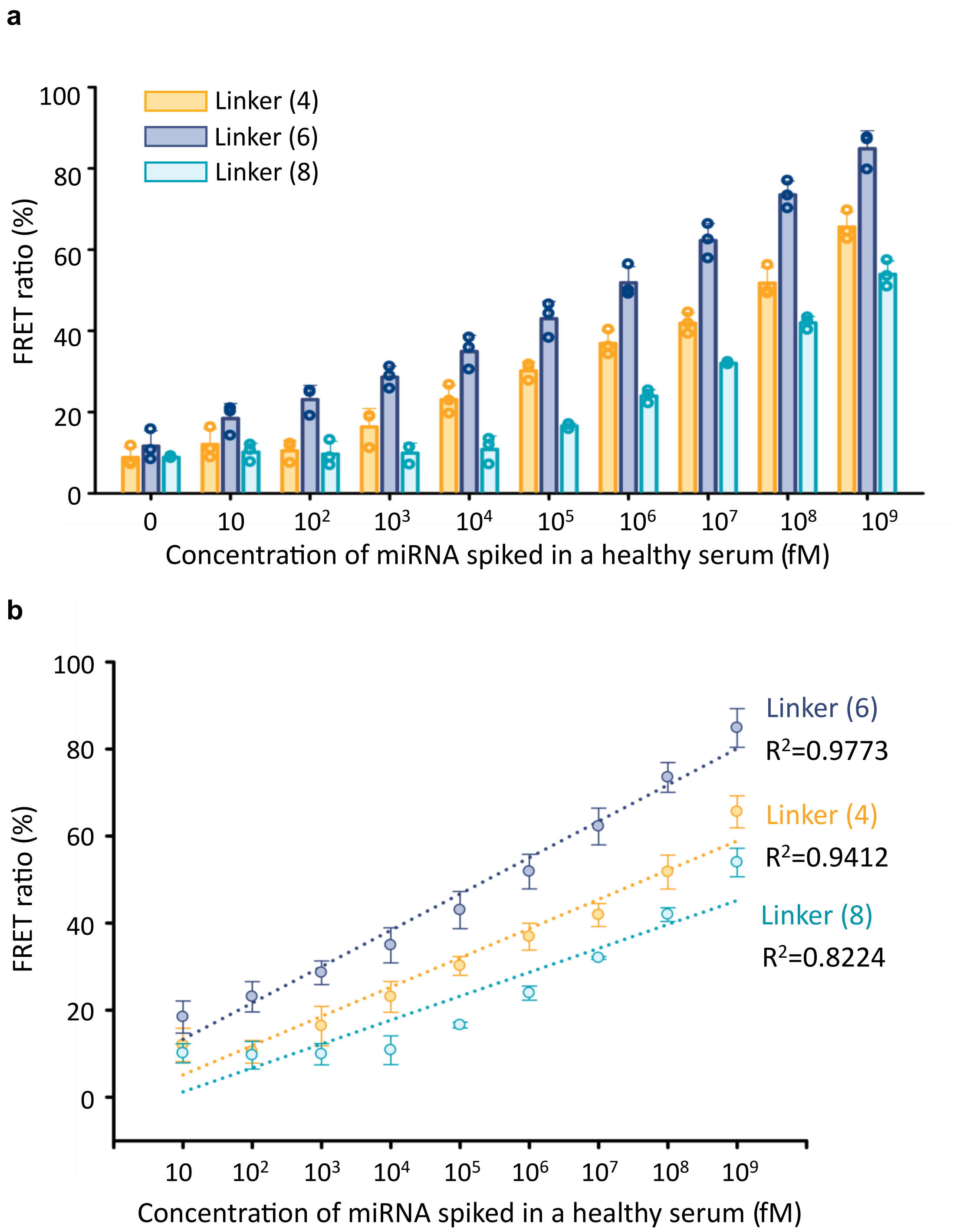

Supplementary Figure 11. Linker-length dependence of RNaseH1-dependent FRET platform signals in serum. (a) FRET ratios measured using the FRET donor incubated with synthetic let-7a spiked into healthy serum, followed by incubation with FRET acceptors containing a four-residue linker, a six-residue linker, or an eight-residue linker. **(b)** Calibration plots of FRET ratio versus log[let-7a] for each acceptor variant. Data represent mean ± s.d. (*n* = 3 technical replicates). Source data are provided as a Source Data file.

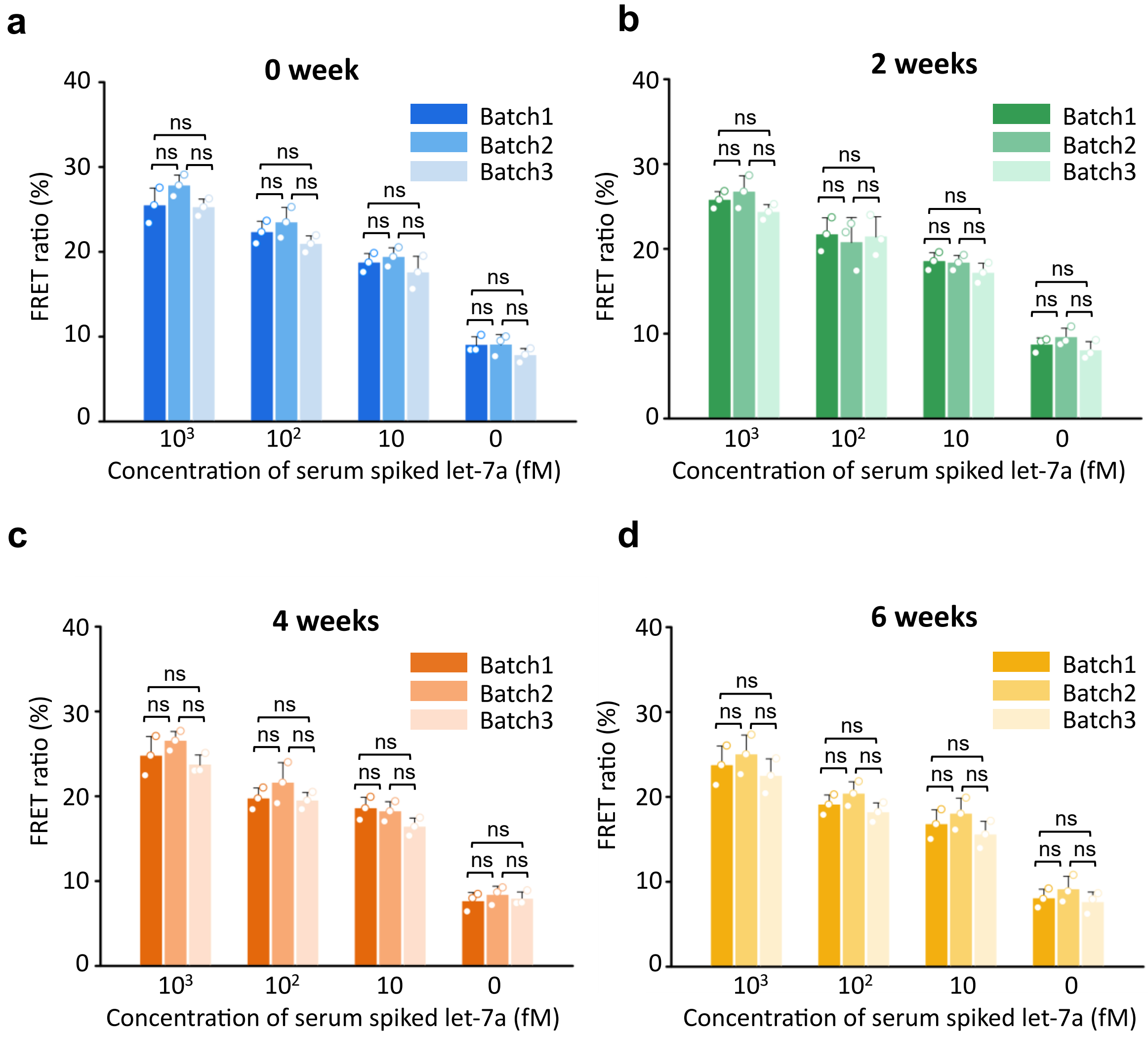

Supplementary Figure 12. Batch-to-batch reproducibility and functional stability of the RNaseH1-dependent FRET platform during frozen storage. (a–d) Batch-resolved FRET responses measured at (a) 0, (b) 2, (c) 4, and (d) 6 weeks using three independently produced donor/acceptor batches (biological replicates), each measured in technical triplicate, for synthetic let-7a spiked into healthy serum at 10^3, 10^2, 10, and 0 fM. Bars indicate mean and error bars indicate s.d. Statistical comparisons between batches at each concentration were performed by two-way ANOVA with Tukey's multiple comparisons test (two-tailed; multiplicity-adjusted P values); ns, not significant. Multiplicity-adjusted P values are provided in the Source Data file; values greater than 0.99 are reported as $P > 0.99$. After preparation of assay-ready materials, all components were divided into single-use aliquots, stored at −80 °C, and thawed only once at the designated time point. Source data are provided as a Source Data file.

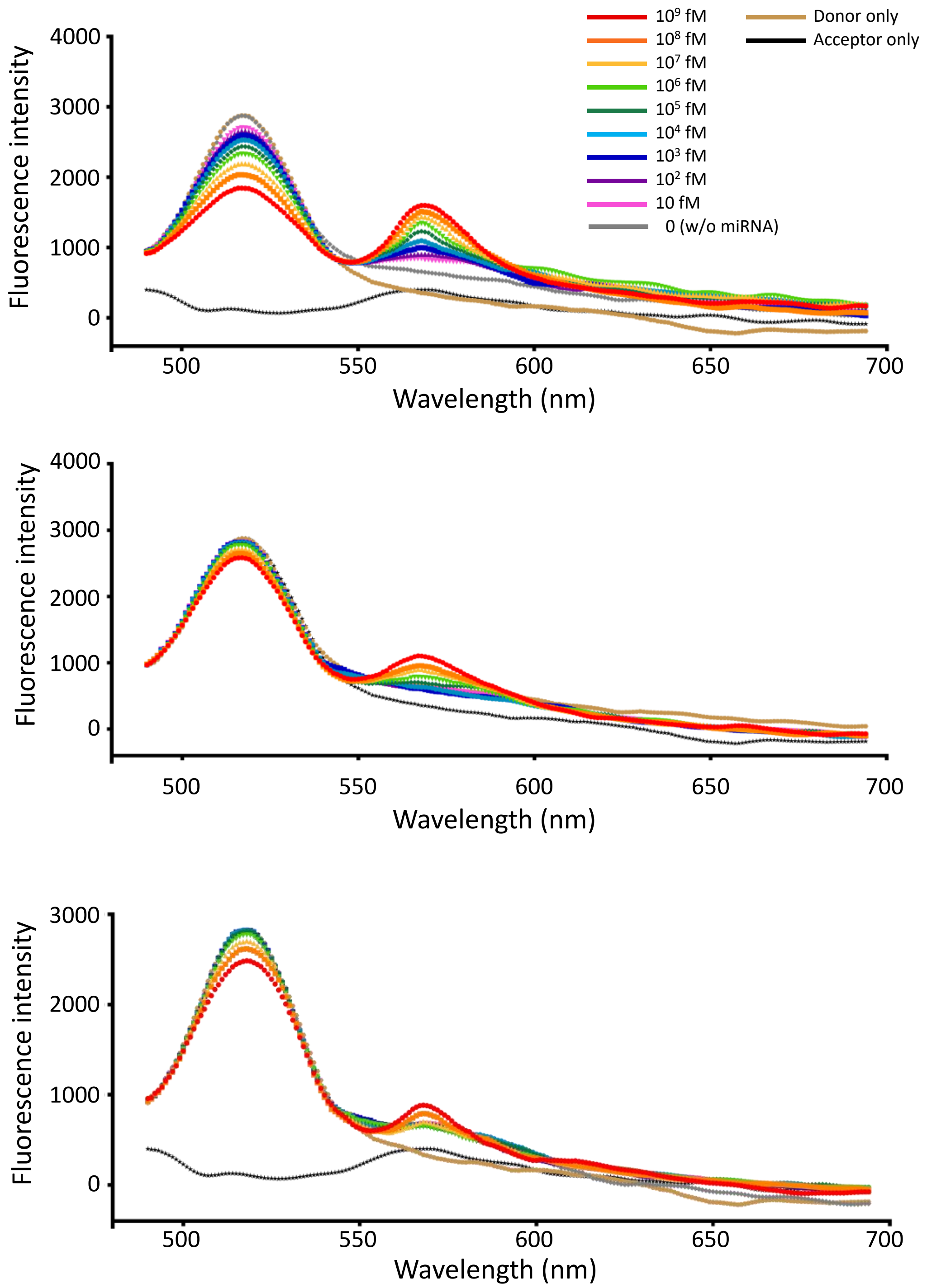

Supplementary Figure 13. Mismatch-dependent emission spectra during let-7 family miRNA detection in serum. Donor-acceptor mixtures were titrated with synthetic miRNA (let-7a, let-7f, and let-7g) spiked into healthy serum over 10 fM to 10^9 fM. Emission spectra were recorded at $\lambda_{ex} = 488$ nm, and each trace was normalized to the donor maximum (Top, fully complementary let-7a; middle, single-mismatch let-7f; bottom, double-mismatch let-7g). Spectra are representative of three independent experiments. Source data are provided as a Source Data file.

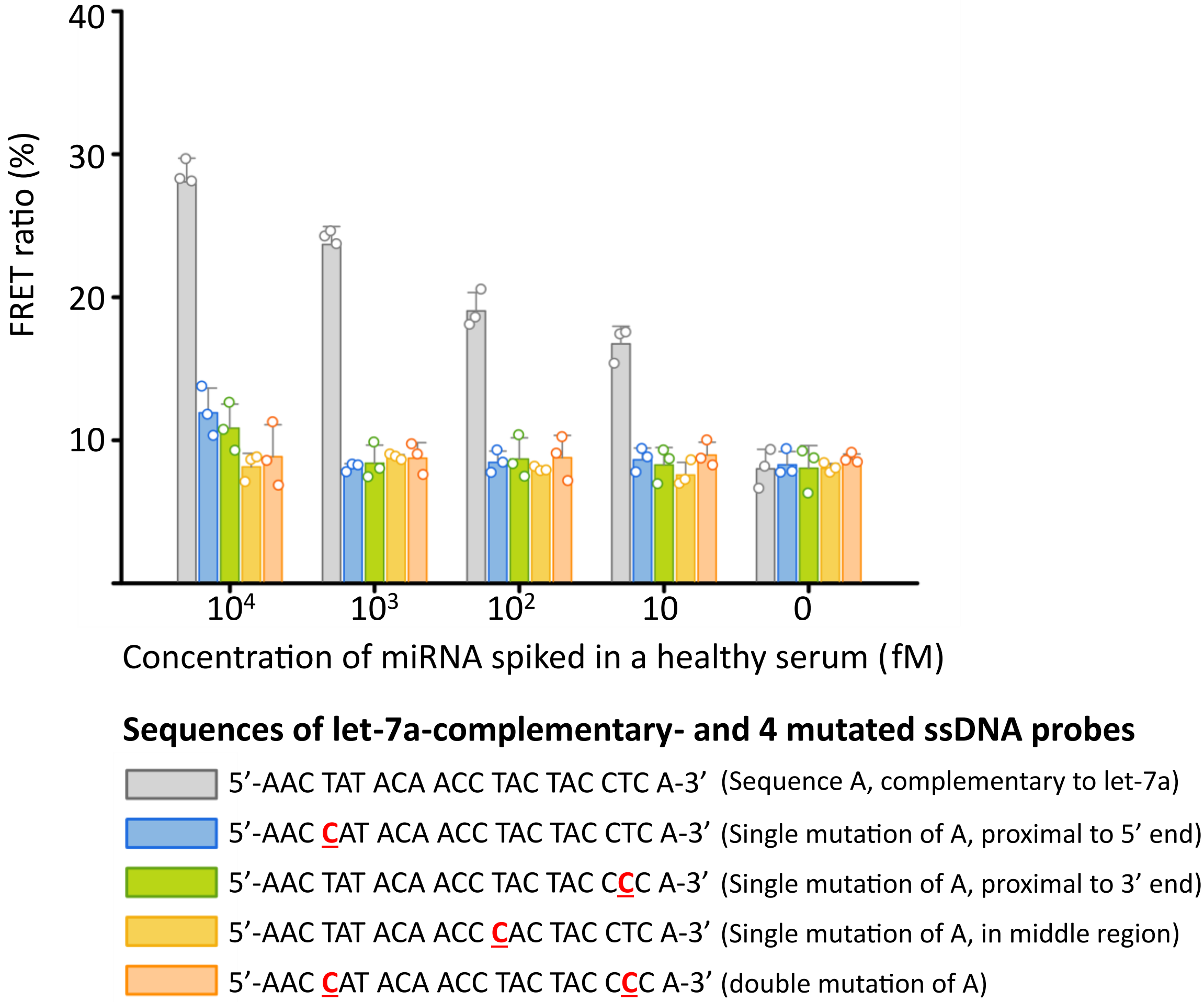

Supplementary Figure 14. Positional-mismatch control for the RNaseH1-dependent FRET platform. FRET ratios measured using donor nanocages bearing either the perfectly matched let-7a-complementary ssDNA probe (grey) or four mutated ssDNA probe variants containing a single mismatch proximal to the 5′ end (blue), proximal to the 3′ end (green), in the middle region (yellow), or a double mismatch at both ends (orange). Let-7a was spiked into healthy serum at 10^4, 10^3, 10^2, 10 fM, and 0 (blank). Only the perfectly matched let-7a-complementary ssDNA probe showed the highest, concentration-dependent signals, whereas all mutated probes yielded notably lower, negligible signals regardless of mismatch position, indicating that the discrimination of mismatched hybrids by the HBD of the FRET acceptor is not influenced by mismatch position. The sequences of the probe variants are shown below the graph; underlined bold red letters indicate the introduced mismatch positions. Data represent mean ± s.d. (*n* = 3 technical replicates). Source data are provided as a Source Data file.

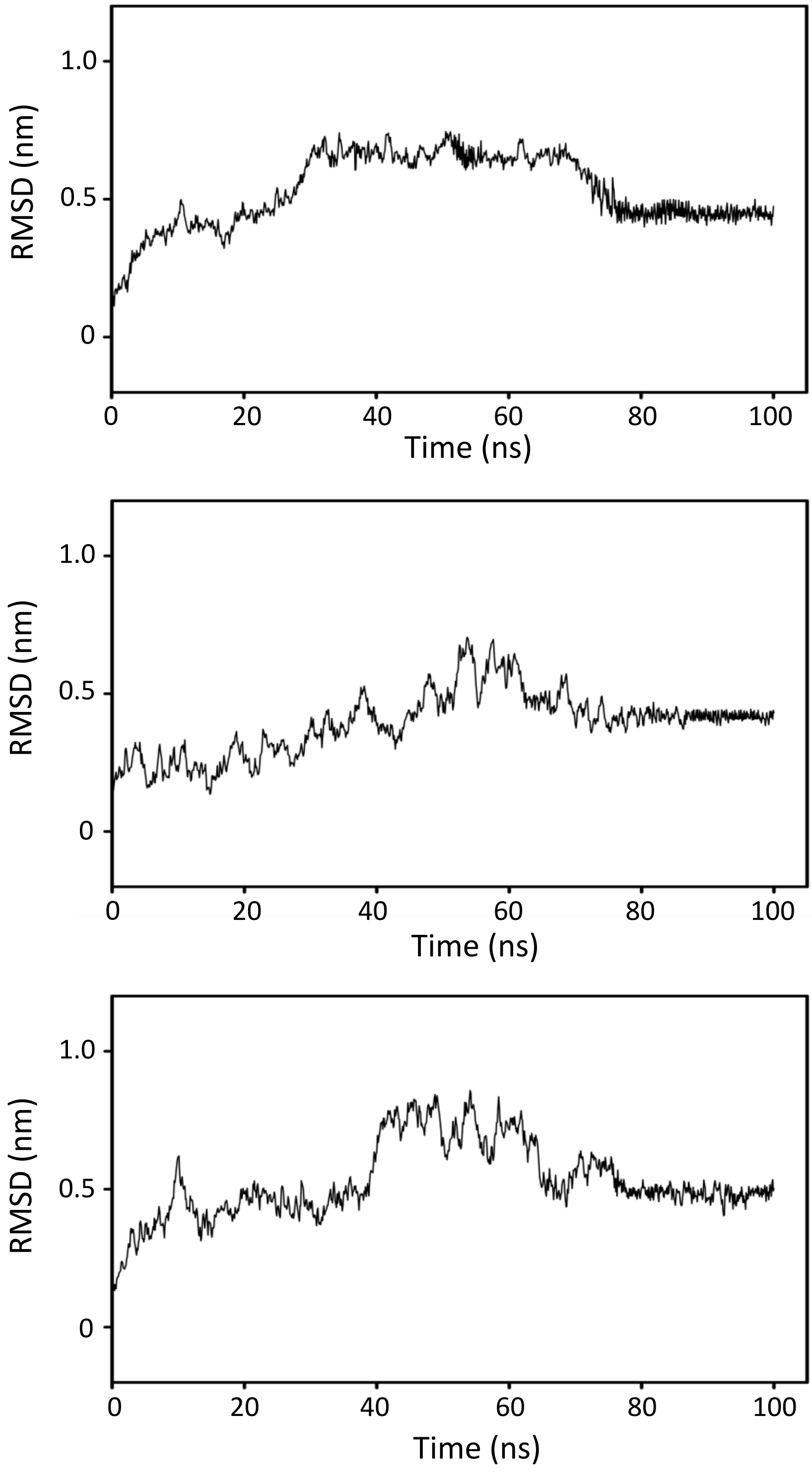

Supplementary Figure 15. Time-dependent RMSD for acceptor bound to miRNA–ssDNA hybrid helices. RMSD versus time over 100 ns production runs at 300 K and 1 bar for complexes of the acceptor bound to perfectly matched (let-7a-ssDNA; top), single-mismatched (let-7f-ssDNA; middle), and double-mismatched (let-7g-ssDNA; bottom) miRNA-ssDNA hybrids. Source data are provided as a Source Data file. (single MD trajectory, 100 ns)

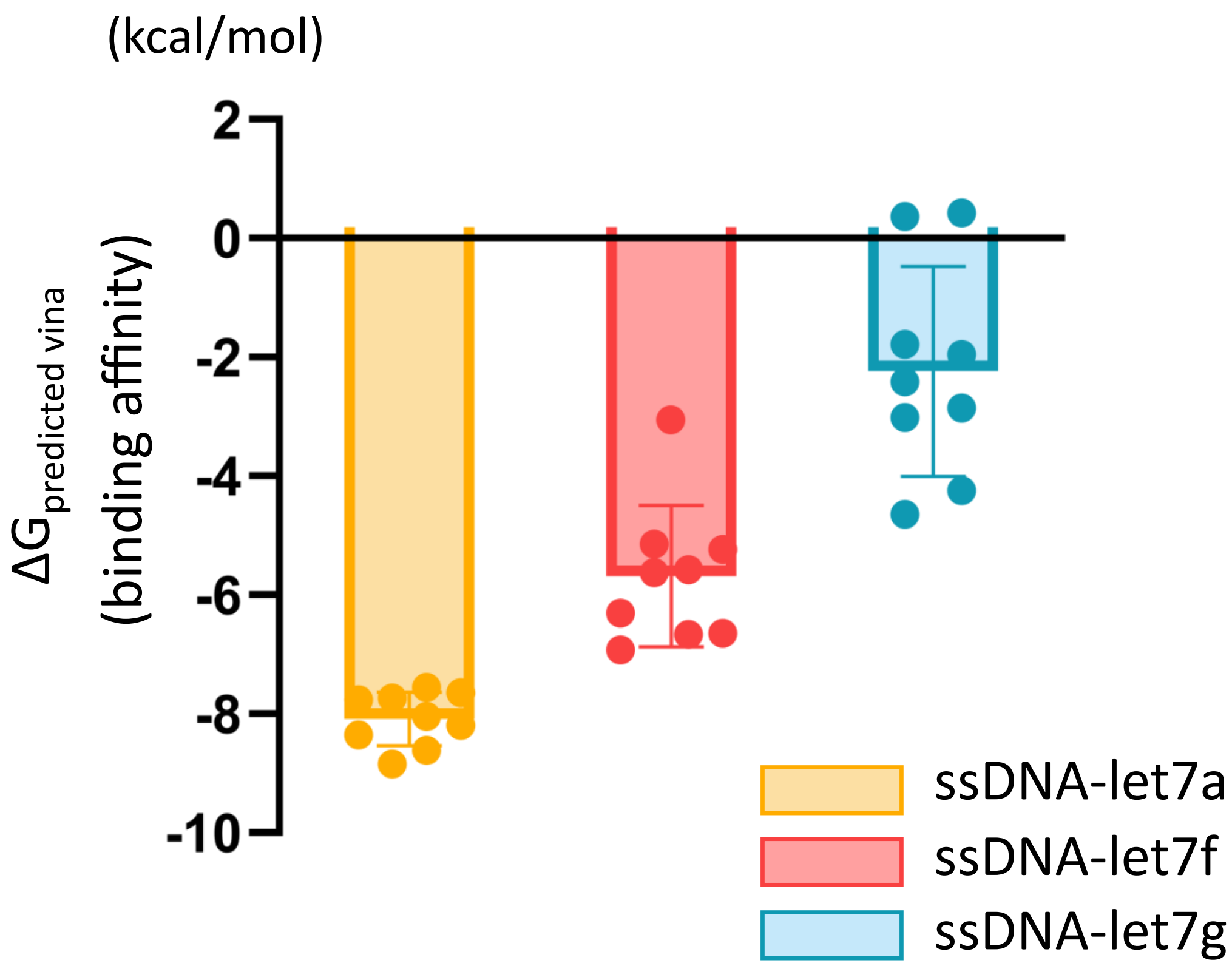

Supplementary Figure 16. AutoDock Vina binding-energy predictions for acceptor bound to miRNA-ssDNA hybrid helices. AutoDock Vina predicted binding energies for docking between the HBD and three hybrid helices (let-7a-ssDNA, let-7f-ssDNA, and let-7g-ssDNA). Bars indicate the mean and error bars represent s.d. ($n = 9$ independent docking poses); points indicate individual scores (kcal mol^{-1}; more negative values indicate stronger predicted affinity). Source data are provided as a Source Data file.

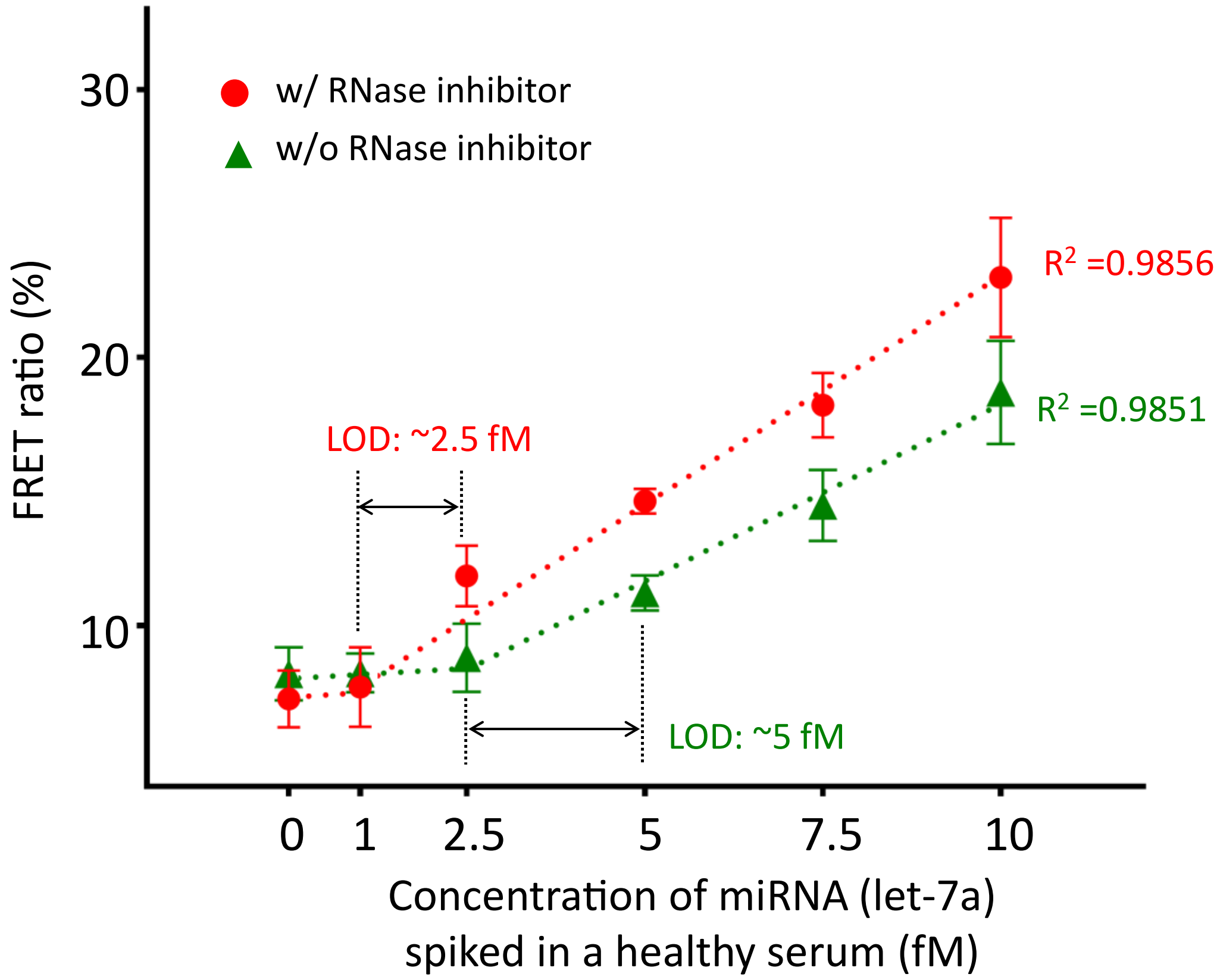

Supplementary Figure 17. Effect of serum RNase activity on assay performance. FRET ratio versus concentration of synthetic let-7a spiked in healthy serum at 0–10 fM, measured in the presence (red circles; 0.5 U/μL Protector RNase Inhibitor, Roche) or absence (green triangles) of an RNase inhibitor. The LOD was estimated to be ~2.5 fM with the RNase inhibitor and ~5 fM without the inhibitor, indicating that endogenous serum RNase activity modestly affects signal sensitivity but does not critically compromise femtomolar detection under the standard assay conditions. Error bars represent s.d. (*n* = 3 technical replicates). Source data are provided as a Source Data file.

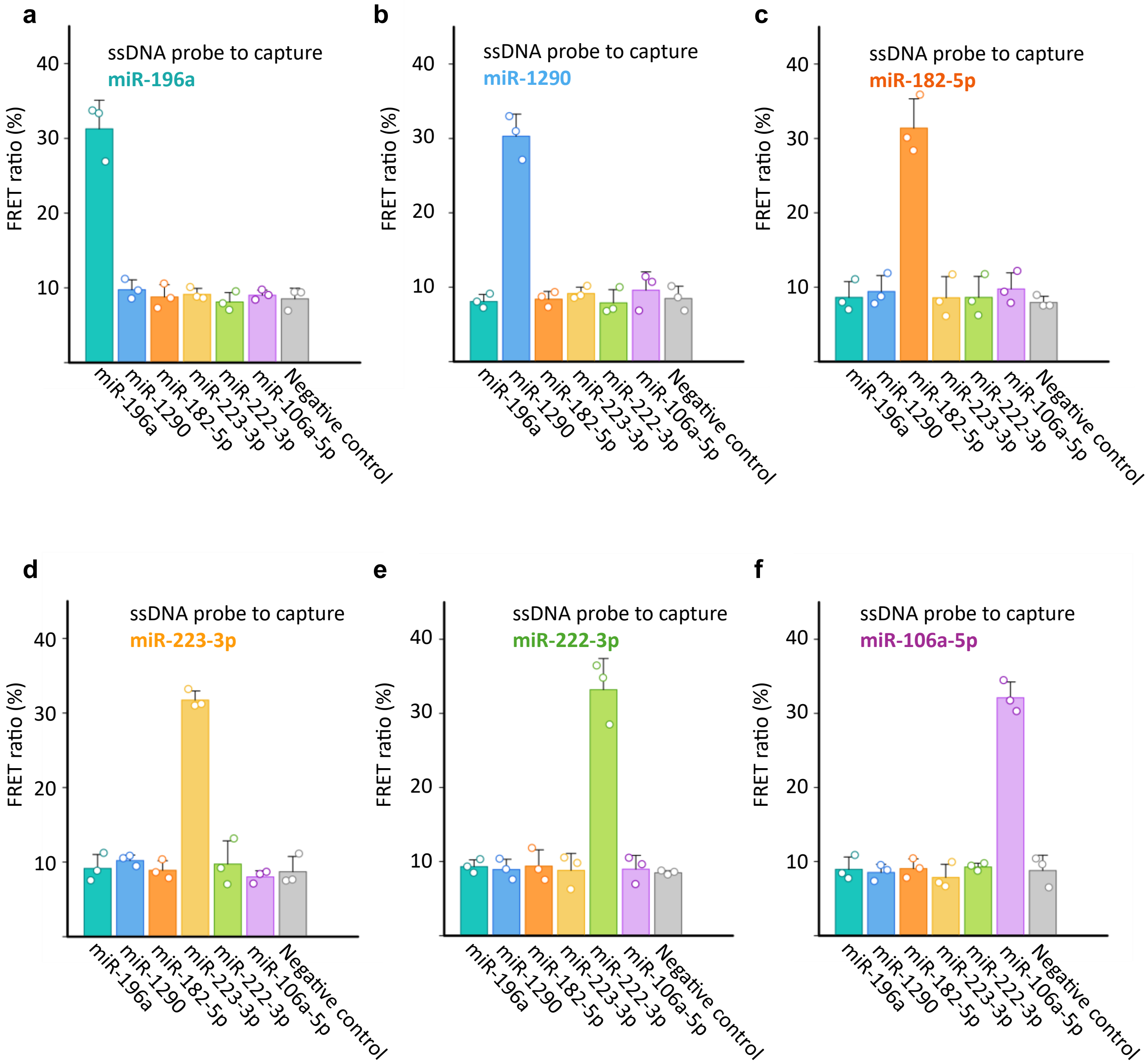

Supplementary Figure 18. Cross-reactivity test of ssDNA probes. FRET ratios measured when each of six ssDNA probes — designed to capture **(a)** miR-196a, **(b)** miR-1290, **(c)** miR-182-5p, **(d)** miR-223-3p, **(e)** miR-222-3p, and **(f)** miR-106a-5p — was incubated with each of the six miRNAs individually and a negative control (no miRNA) at 10^4 fM in healthy serum. Each probe yielded a high FRET ratio exclusively for its complementary target, while all non-target miRNAs and the negative control produced baseline-level signals, confirming probe specificity across diverse miRNA sequences. Data represent mean ± s.d. (n = 3 technical replicates). Source data are provided as a Source Data file.

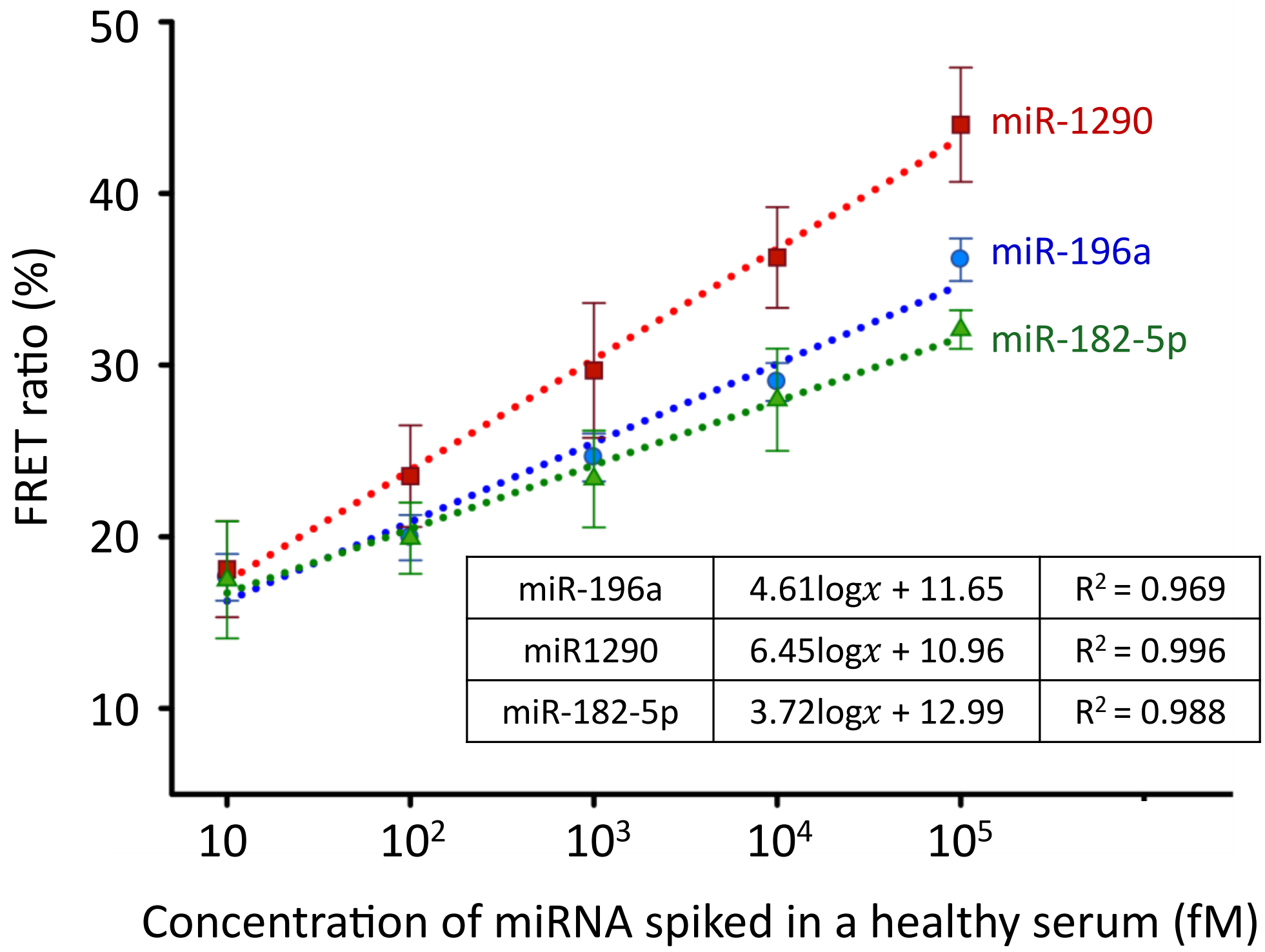

Supplementary Figure 19. Calibration of miRNA detection by the RNaseH1-dependent FRET platform. Calibration plots of FRET ratio (%) versus log concentration (fM) for synthetic miR-196a (blue circles), miR-1290 (red squares), and miR-182-5p (green triangles) spiked in healthy serum. Dotted lines represent log-linear regression fits; equations and R^2 values are shown in the inset table. Error bars represent s.d. (n = 3 technical replicates). Source data are provided as a Source Data file.

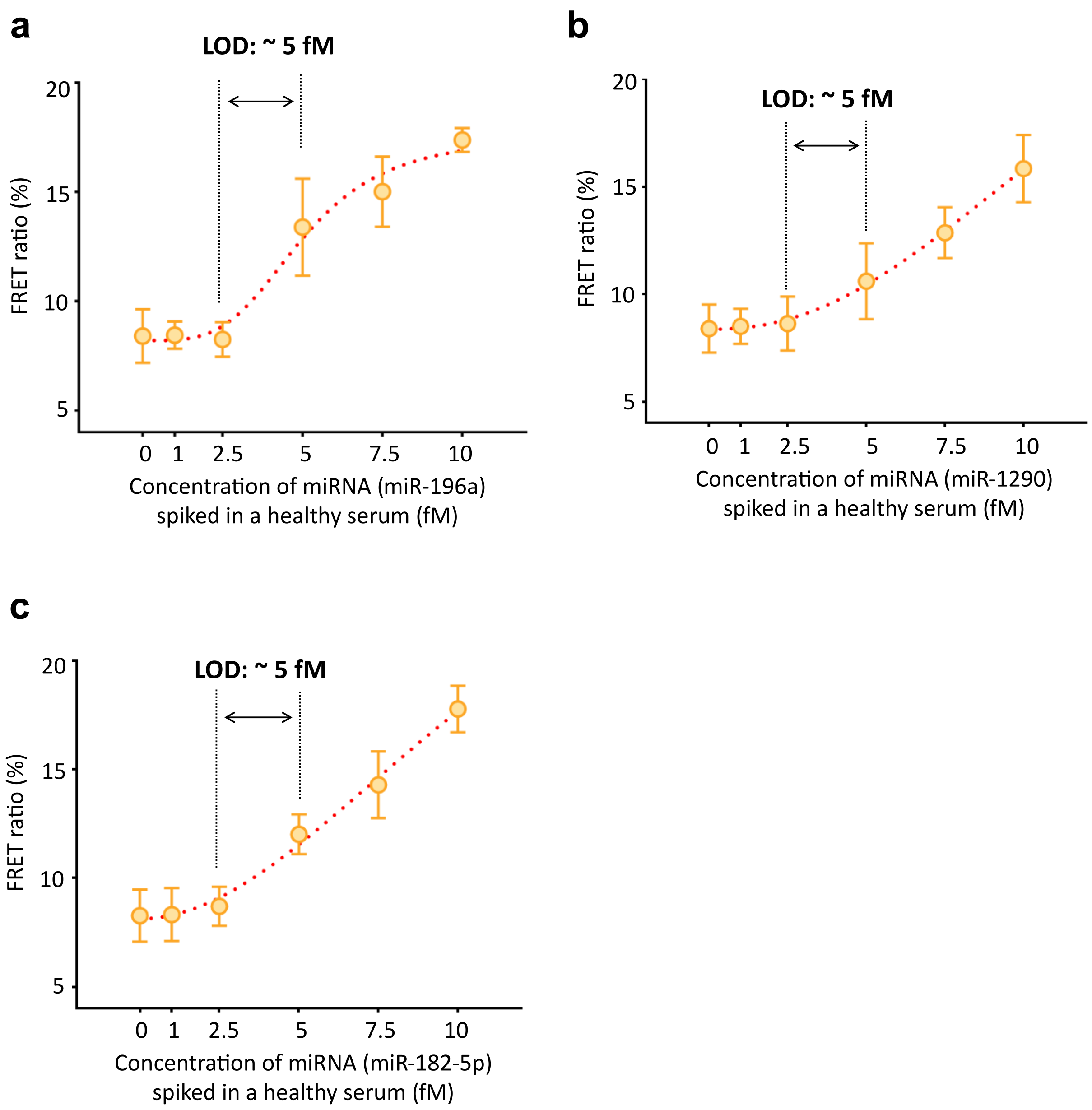

Supplementary Figure 20. Limit of detection (LOD) for three miRNA markers. FRET ratio versus concentration of synthetic **(a)** miR-196a, **(b)** miR-1290, and **(c)** miR-182-5p spiked in healthy serum at 0–10 fM. The LOD was estimated to be ~5 fM for all three markers, defined as the concentration corresponding to three times the standard deviation of the blank signal above the mean blank signal. Error bars represent s.d. (*n* = 3 technical replicates). Source data are provided as a Source Data file.

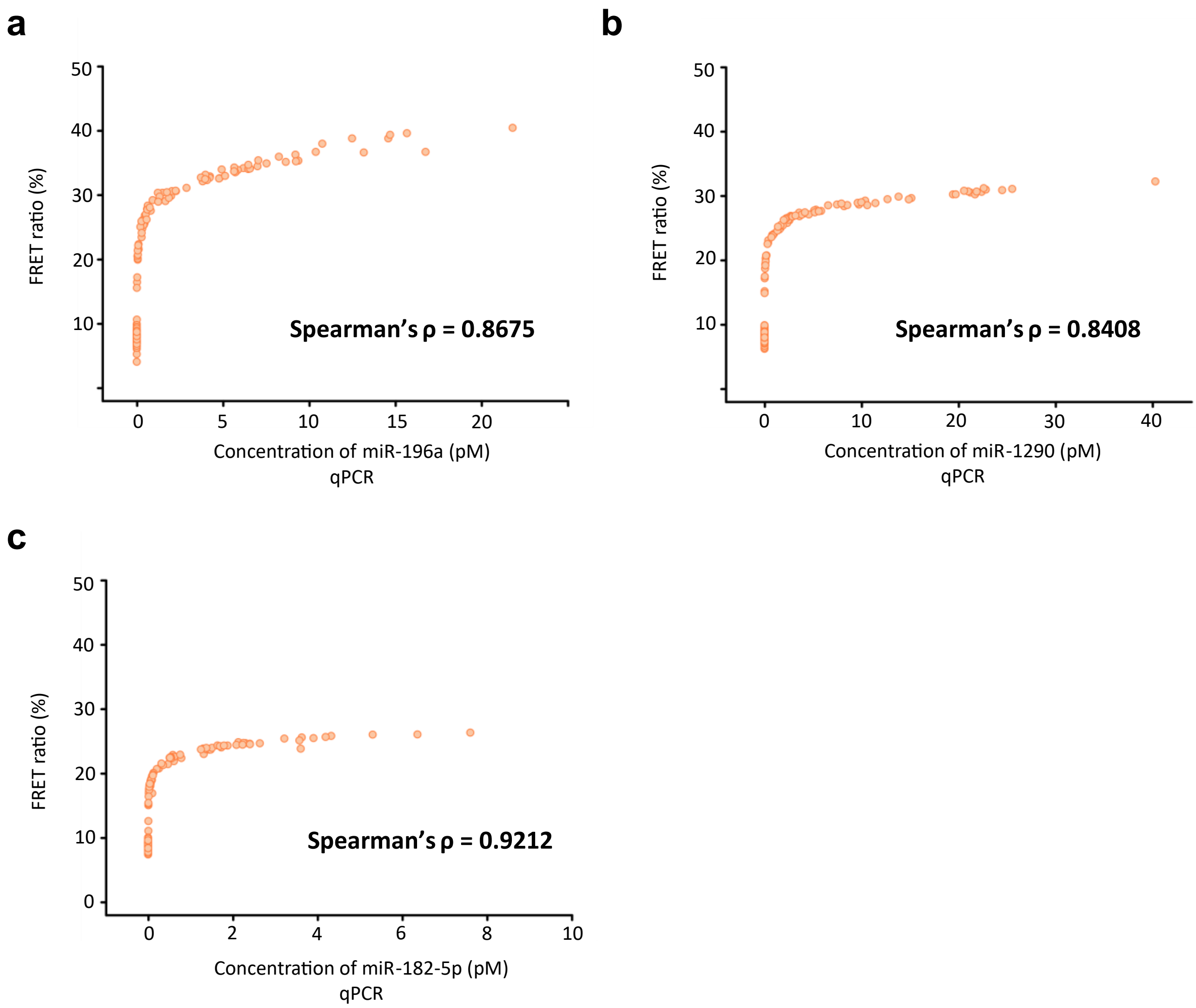

Supplementary Figure 21. Correlation between miRNA quantification by RT-qPCR and the FRET platform for NSCLC-associated markers. Correlation between miRNA concentrations (pM) estimated by RT-qPCR (x-axis) and FRET ratios (%) measured by the RNaseH1-dependent FRET platform (y-axis) for (a) miR-196a (Spearman's $\rho = 0.8675$), (b) miR-1290 (Spearman's $\rho = 0.8408$), and (c) miR-182-5p (Spearman's $\rho = 0.9212$). All $P < 0.0001$. Data include both NSCLC patient and healthy donor sera ($n = 160$). Source data are provided as a Source Data file.

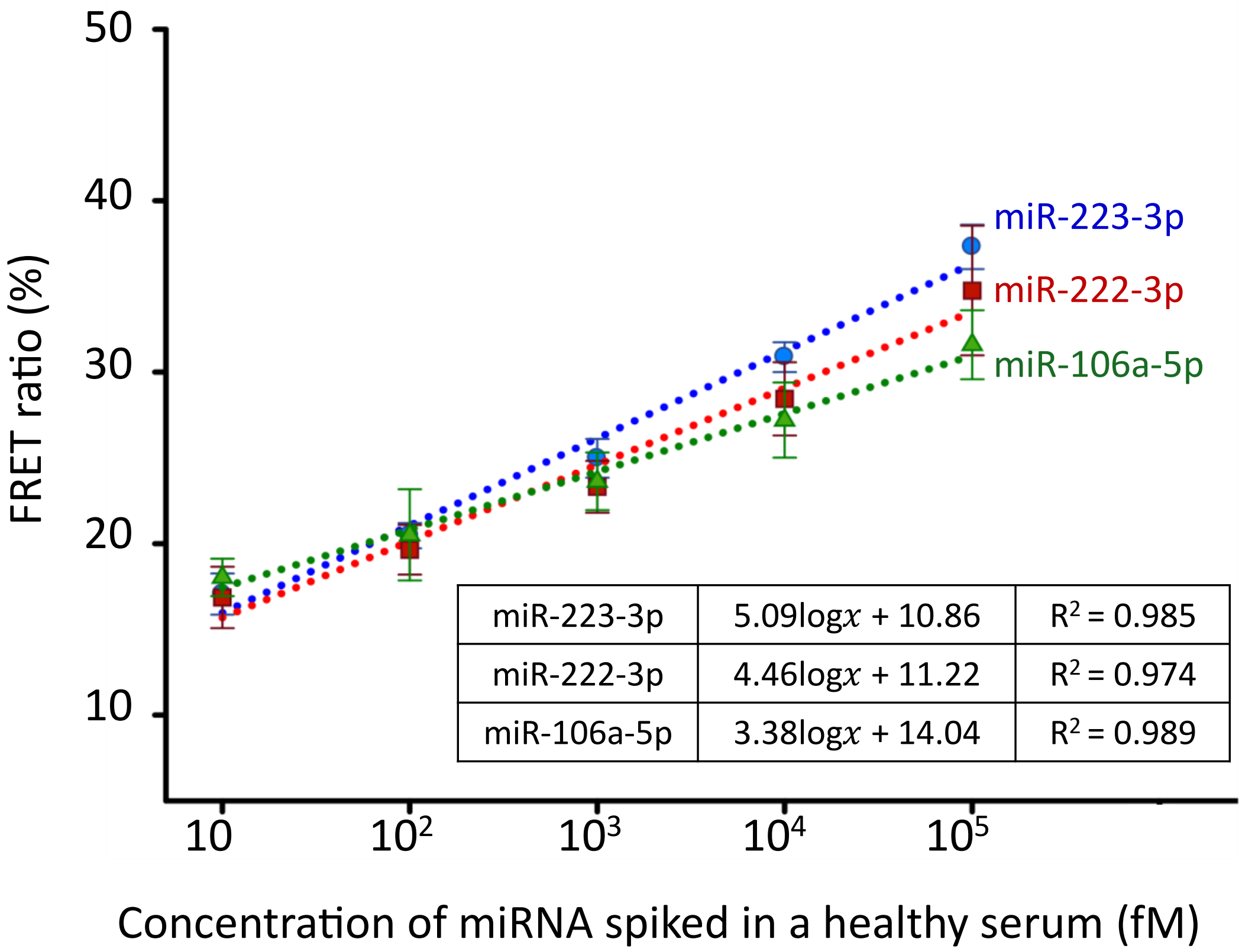

Supplementary Figure 22. Calibration of gastric cancer-associated miRNA detection by the RNaseH1-dependent FRET platform. Calibration plots of FRET ratio (%) versus log concentration (fM) for synthetic miR-223-3p (blue circles), miR-222-3p (red squares), and miR-106a-5p (green triangles) spiked in healthy serum. Dotted lines represent log-linear regression fits; equations and R^2 values are shown in the inset table. Error bars represent s.d. (n = 3 technical replicates). Source data are provided as a Source Data file.

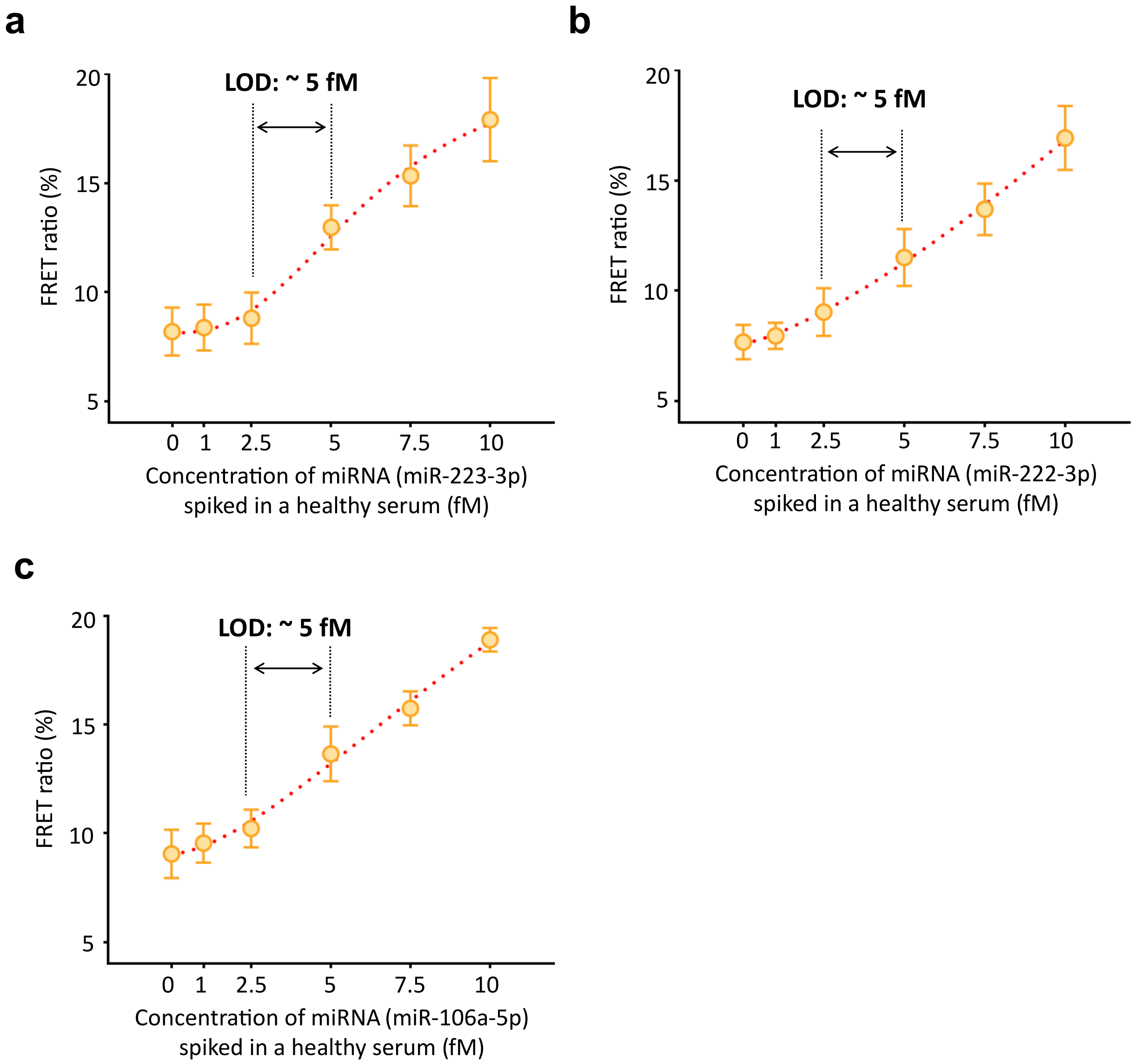

Supplementary Figure 23. Limit of detection (LOD) for three gastric cancer-associated miRNA markers. FRET ratio versus concentration of synthetic **(a)** miR-223-3p, **(b)** miR-222-3p, and **(c)** miR-106a-5p spiked in healthy serum at 0–10 fM. The LOD was estimated to be ~5 fM for all three markers, defined as the concentration corresponding to three times the standard deviation of the blank signal above the mean blank signal. Error bars represent s.d. (n = 3 technical replicates). Source data are provided as a Source Data file.

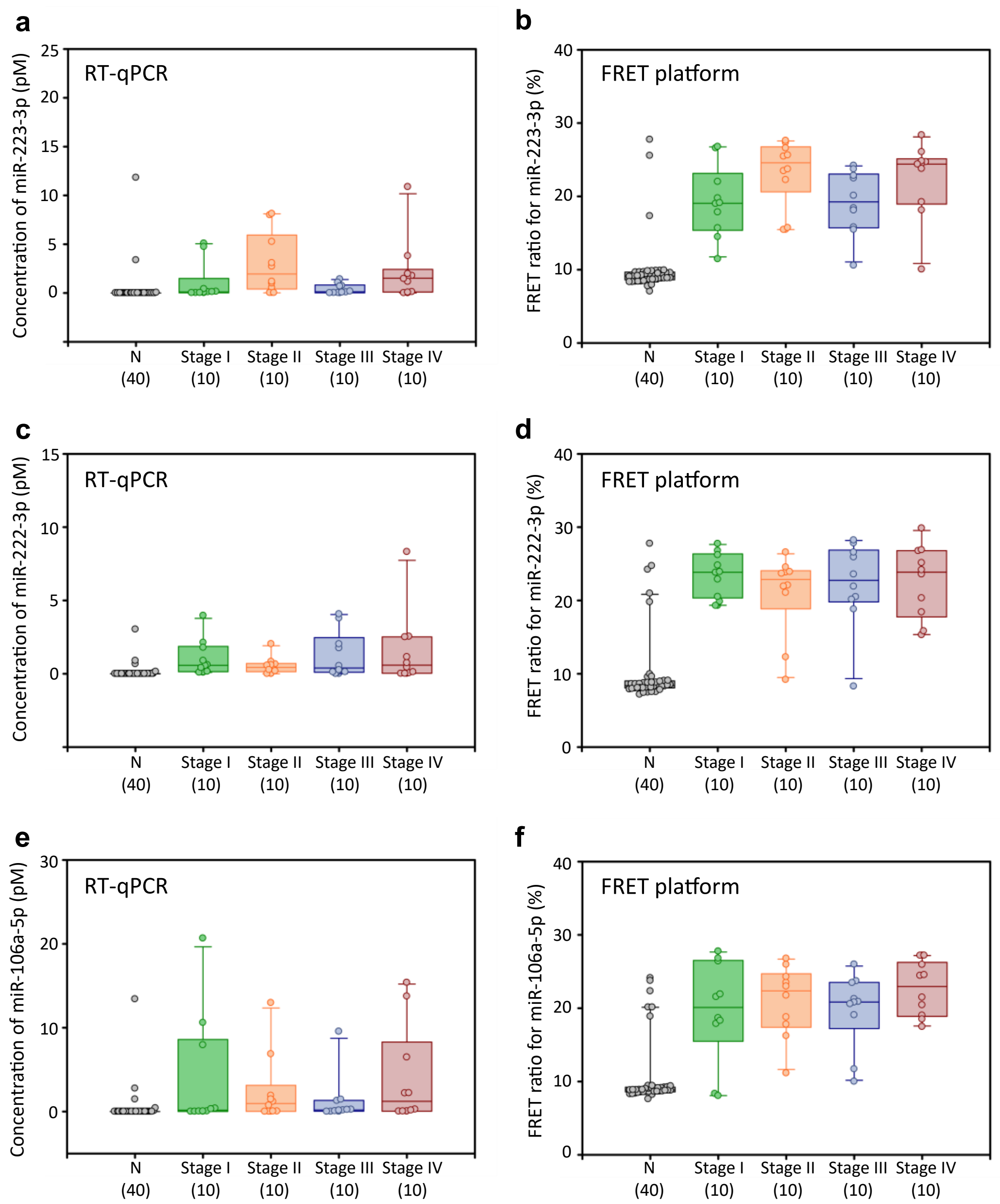

Supplementary Figure 24. Clinical detection of gastric cancer-associated miRNAs. (a,c,e) Serum concentrations of miR-223-3p (a), miR-222-3p (c), and miR-106a-5p (e), measured by RT-qPCR, for healthy donors (N; $n = 40$) and gastric cancer patients at clinical stage I ($n = 10$), stage II ($n = 10$), stage III ($n = 10$), and stage IV ($n = 10$). **(b,d,f)** FRET ratios (%) measured by the RNaseH1-dependent FRET platform for the same serum samples using ssDNA probes complementary to miR-223-3p (b), miR-222-3p (d), and miR-106a-5p (f). Box plots show median (center line), interquartile range (25th–75th percentiles, box), 1.5× IQR (whiskers), and individual data points (circles); the minima and maxima are the smallest and largest plotted values. Each data point represents one biological replicate (an individual patient or donor), shown as the mean of $n = 3$ technical replicates. Source data are provided as a Source Data file.

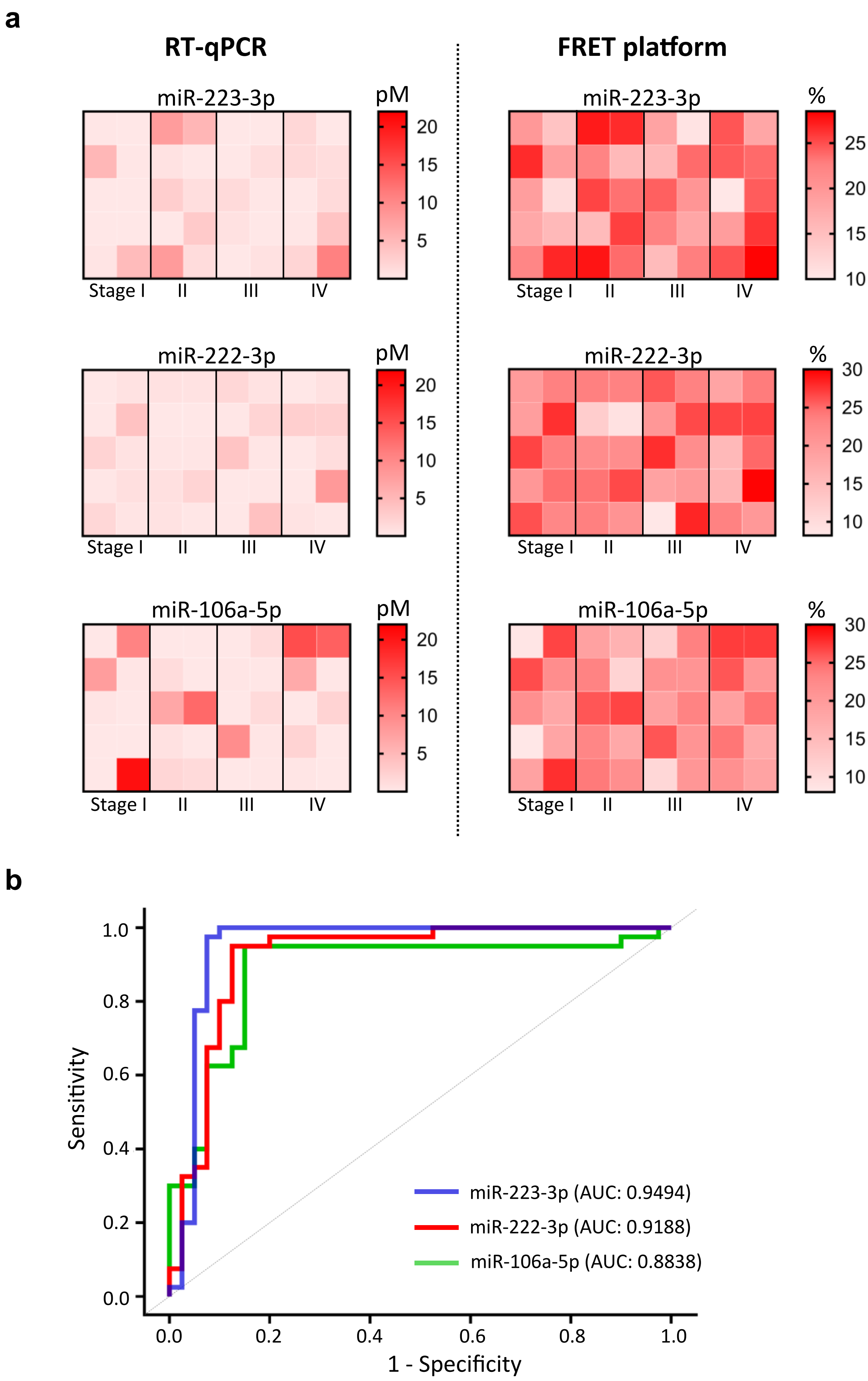

a
RT-qPCR
FRET platform
miR-223-3p
miR-222-3p
miR-106a-5p
pM
%
Stage I
II
III
IV
b
Sensitivity
1 - Specificity
miR-223-3p (AUC: 0.9494)
miR-222-3p (AUC: 0.9188)
miR-106a-5p (AUC: 0.8838)

Supplementary Figure 25. Heat maps and ROC curves for gastric cancer diagnosis. (a) Heat maps of miRNA quantification in gastric cancer patient sera (n = 40) determined by RT-qPCR (left) and by the RNaseH1-dependent FRET platform (right) for miR-223-3p (top), miR-222-3p (middle), and miR-106a-5p (bottom). Each cell represents a single serum sample; gastric cancer patient samples are grouped by clinical stage (stage I to IV) on the right. The color scale indicates the miRNA concentration (pM) for RT-qPCR and the FRET ratio (%) for the FRET platform. Each cell represents one biological replicate (one patient), shown as the mean of n = 3 technical replicates. **(b)** ROC curves for the FRET platform-based detection of three gastric cancer-associated miRNA markers; AUC values are 0.9494 (miR-223-3p; sensitivity 95.0%, specificity 92.5%), 0.9188 (miR-222-3p; sensitivity 95.0%, specificity 87.5%), and 0.8838 (miR-106a-5p; sensitivity 95.0%, specificity 85.0%). Source data are provided as a Source Data file.

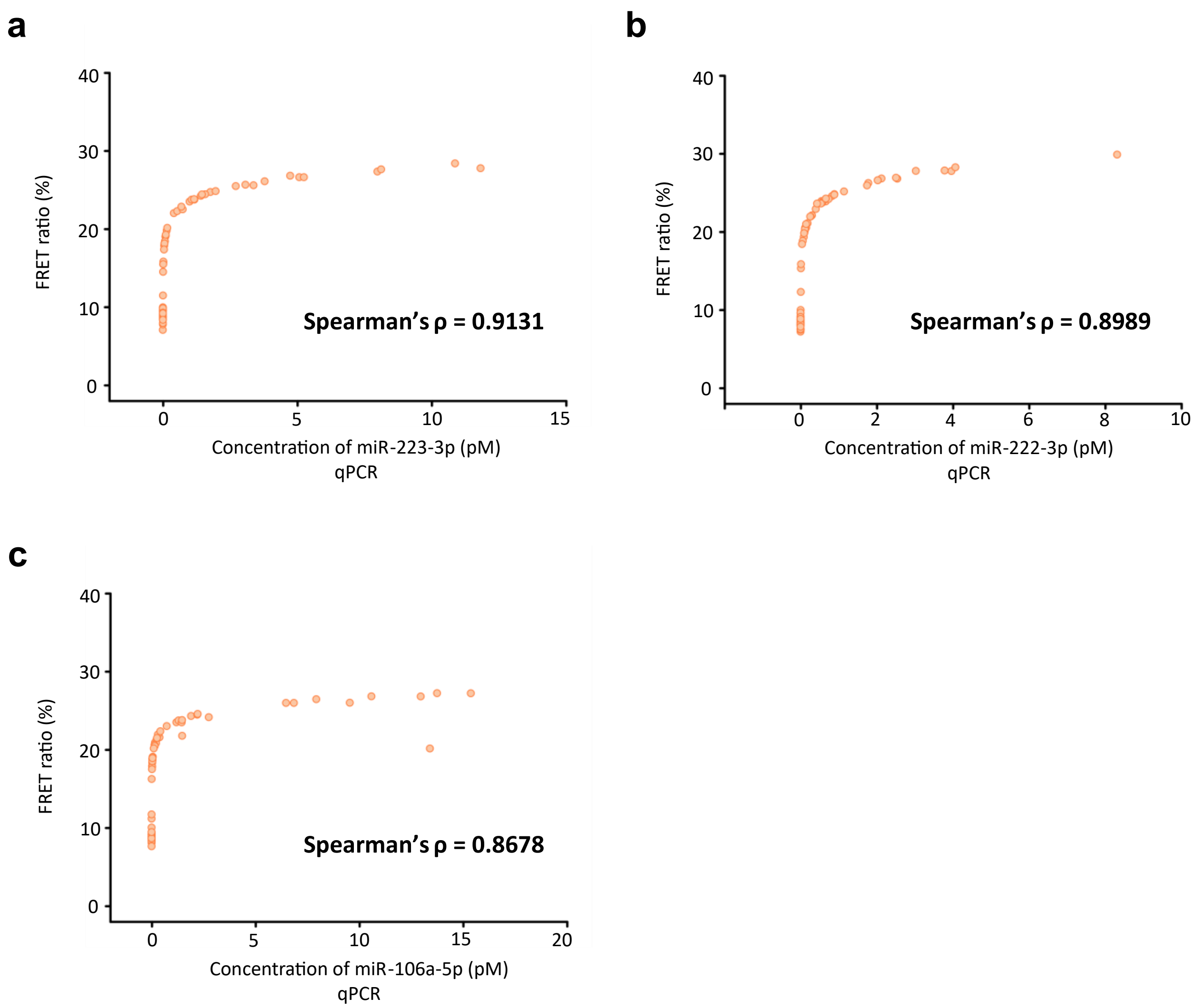

Supplementary Figure 26. Correlation between miRNA quantification by RT-qPCR and the FRET platform for gastric cancer-associated markers. Correlation between miRNA concentrations (pM) obtained by RT-qPCR (x-axis) and FRET ratios (%) measured by the RNaseH1-dependent FRET platform (y-axis) for **(a)** miR-223-3p (Spearman's ρ = 0.9131), **(b)** miR-222-3p (Spearman's ρ = 0.8989), and **(c)** miR-106a-5p (Spearman's ρ = 0.8678). All $P < 0.0001$. Data include both gastric cancer patient and healthy donor sera (n = 80). Source data are provided as a Source Data file.

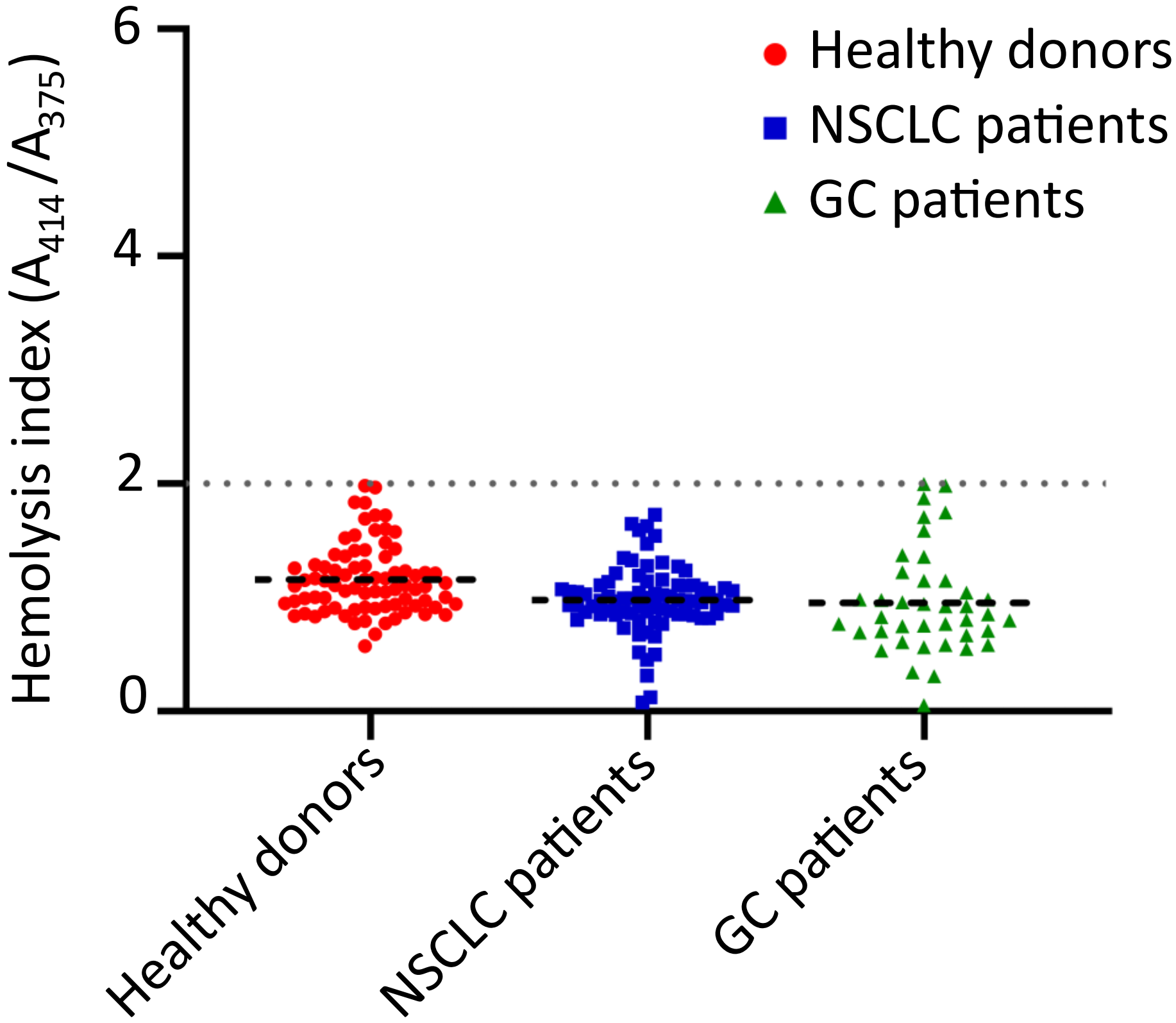

Supplementary Figure 27. Hemolysis assessment of serum specimens. Hemolysis index (A_{414}/A_{375}) for all serum samples used in this study: healthy donors (*n* = 80, red circles), NSCLC patients (*n* = 80, blue squares), and gastric cancer patients (*n* = 40, green triangles). The dotted line indicates the hemolysis threshold of 2.0. All samples exhibited hemolysis indices below the threshold, confirming that hemolysis did not confound the assay results. Dashed lines indicate mean values for each group. Source data are provided as a Source Data file.

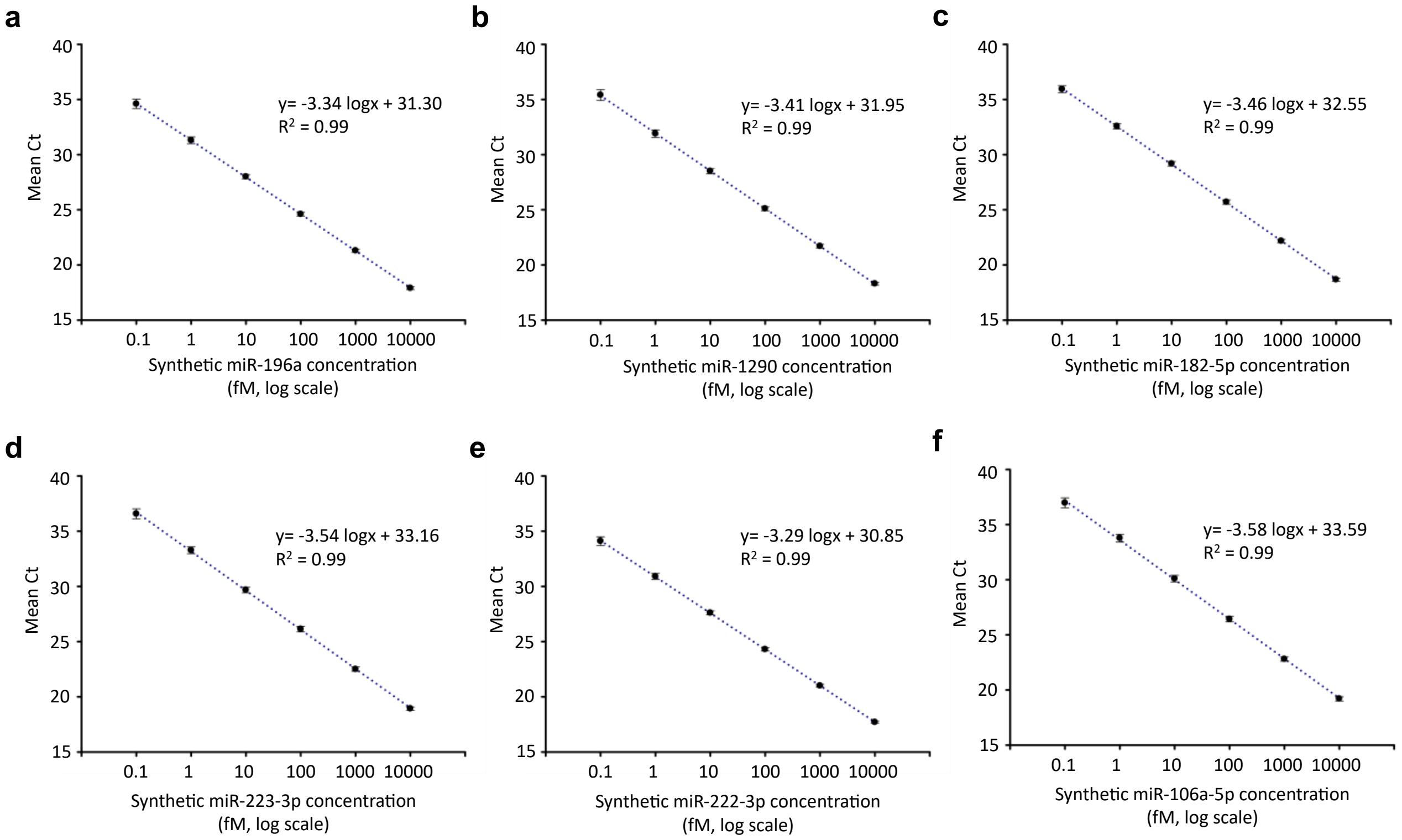

Supplementary Figure 28. RT-qPCR standard curves for the six miRNA targets used in this study. Calibration plots of mean Ct versus log concentration of synthetic miRNA standards for **(a)** miR-196a ($y = -3.34\ \log x + 31.30$, $R^2 = 0.99$), **(b)** miR-1290 ($y = -3.41\ \log x + 31.95$, $R^2 = 0.99$), **(c)** miR-182-5p ($y = -3.46\ \log x + 32.55$, $R^2 = 0.99$), **(d)** miR-223-3p ($y = -3.54\ \log x + 33.16$, $R^2 = 0.99$), **(e)** miR-222-3p ($y = -3.29\ \log x + 30.85$, $R^2 = 0.99$), and **(f)** miR-106a-5p ($y = -3.58\ \log x + 33.59$, $R^2 = 0.99$). Synthetic standards were reverse-transcribed and analyzed in parallel with the serum samples. Dotted lines indicate linear regression fits. Error bars represent s.d. ($n = 3$ technical replicates). Source data are provided as a Source Data file.

Supplementary Tables

Supplementary Table 1 | Demographic, clinical, and histopathological characteristics and smoking status of NSCLC patients (*n* = 80). (a) Sex, age, and clinical stage. **(b)** Histologic type classified according to the WHO Classification. **(c)** Smoking status (Y, current smoker; N, non-smoker).

(a)

	Sex	Age	Stage		Sex	Age	Stage		Sex	Age	Stage		Sex	Age	Stage
**P1**	F	40s	4	**P21**	F	80s	2	**P41**	M	60s	1	**P61**	M	60s	2
**P2**	M	60s	4	**P22**	F	70s	3	**P42**	M	40s	1	**P62**	F	60s	2
**P3**	M	50s	3	**P23**	M	70s	2	**P43**	F	70s	1	**P63**	M	80s	2
**P4**	M	70s	1	**P24**	M	80s	2	**P44**	F	30s	1	**P64**	M	60s	2
**P5**	F	80s	4	**P25**	M	70s	2	**P45**	F	70s	1	**P65**	F	70s	2
**P6**	F	60s	1	**P26**	M	70s	2	**P46**	M	60s	1	**P66**	M	70s	2
**P7**	M	60s	4	**P27**	M	60s	3	**P47**	M	50s	1	**P67**	M	50s	2
**P8**	M	70s	3	**P28**	M	60s	4	**P48**	M	60s	1	**P68**	M	60s	2
**P9**	M	60s	4	**P29**	M	50s	3	**P49**	F	60s	1	**P69**	F	70s	2
**P10**	F	60s	1	**P30**	M	50s	1	**P50**	F	50s	1	**P70**	M	40s	2
**P11**	F	70s	4	**P31**	M	70s	1	**P51**	M	50s	1	**P71**	M	60s	3
**P12**	M	80s	1	**P32**	F	70s	3	**P52**	M	60s	1	**P72**	M	60s	3
**P13**	F	50s	4	**P33**	M	60s	1	**P53**	F	70s	1	**P73**	F	70s	3
**P14**	M	70s	3	**P34**	M	40s	3	**P54**	F	60s	1	**P74**	F	60s	3
**P15**	F	70s	1	**P35**	F	60s	2	**P55**	M	50s	1	**P75**	M	80s	3
**P16**	F	70s	3	**P36**	M	60s	4	**P56**	M	60s	2	**P76**	M	80s	4
**P17**	M	50s	4	**P37**	F	50s	1	**P57**	M	80s	2	**P77**	F	50s	4
**P18**	F	60s	1	**P38**	M	60s	2	**P58**	M	60s	2	**P78**	F	50s	4
**P19**	M	60s	2	**P39**	F	70s	2	**P59**	M	80s	2	**P79**	M	50s	4
**P20**	F	70s	3	**P40**	F	40s	2	**P60**	M	60s	2	**P80**	M	70s	4

(b)

Patient	Histologic type	Patient	Histologic type
**P1**	Adenocarcinoma	**P41**	Adenocarcinoma
**P2**	Adenocarcinoma	**P42**	Adenocarcinoma
**P3**	Squamous cell carcinoma	**P43**	Adenocarcinoma
**P4**	Adenocarcinoma	**P44**	Adenocarcinoma
**P5**	Adenocarcinoma	**P45**	Adenocarcinoma
**P6**	Adenocarcinoma	**P46**	Adenocarcinoma
**P7**	Adenocarcinoma	**P47**	Adenocarcinoma
**P8**	Squamous cell carcinoma	**P48**	Adenocarcinoma
**P9**	Adenocarcinoma	**P49**	Adenocarcinoma
**P10**	Adenocarcinoma	**P50**	Adenocarcinoma
**P11**	Adenocarcinoma	**P51**	Adenocarcinoma
**P12**	Adenocarcinoma	**P52**	Squamous cell carcinoma
**P13**	Adenocarcinoma	**P53**	Squamous cell carcinoma
**P14**	Squamous cell carcinoma	**P54**	Adenocarcinoma
**P15**	Adenocarcinoma	**P55**	Adenocarcinoma
**P16**	Adenocarcinoma	**P56**	Adenocarcinoma
**P17**	Squamous cell carcinoma	**P57**	Squamous cell carcinoma
**P18**	Adenocarcinoma	**P58**	Squamous cell carcinoma
**P19**	Squamous cell carcinoma	**P59**	Squamous cell carcinoma
**P20**	Adenocarcinoma	**P60**	Adenocarcinoma
**P21**	Adenocarcinoma	**P61**	Adenocarcinoma
**P22**	Adenocarcinoma	**P62**	Adenocarcinoma
**P23**	Adenocarcinoma	**P63**	Adenocarcinoma
**P24**	Squamous cell carcinoma	**P64**	Squamous cell carcinoma
**P25**	Squamous cell carcinoma	**P65**	Adenocarcinoma
**P26**	Adenocarcinoma	**P66**	Adenocarcinoma
**P27**	Squamous cell carcinoma	**P67**	Adenocarcinoma
**P28**	Adenocarcinoma	**P68**	Squamous cell carcinoma
**P29**	Squamous cell carcinoma	**P69**	Adenocarcinoma
**P30**	Adenocarcinoma	**P70**	Adenocarcinoma
**P31**	Adenocarcinoma	**P71**	Adenocarcinoma
**P32**	Adenocarcinoma	**P72**	Squamous cell carcinoma
**P33**	Adenocarcinoma	**P73**	Squamous cell carcinoma
**P34**	Adenocarcinoma	**P74**	Squamous cell carcinoma
**P35**	Adenocarcinoma	**P75**	Adenocarcinoma
**P36**	Adenocarcinoma	**P76**	Squamous cell carcinoma
**P37**	Adenocarcinoma	**P77**	Adenocarcinoma
**P38**	Squamous cell carcinoma	**P78**	Adenocarcinoma
**P39**	Adenocarcinoma	**P79**	Adenocarcinoma
**P40**	Adenocarcinoma	**P80**	Squamous cell carcinoma

(c)

Patient	Smoking status	Patient	Smoking status
**P1**	N	**P41**	Y
**P2**	Y	**P42**	N
**P3**	Y	**P43**	N
**P4**	Y	**P44**	Y
**P5**	N	**P45**	N
**P6**	N	**P46**	Y
**P7**	Y	**P47**	Y
**P8**	Y	**P48**	N
**P9**	N	**P49**	N
**P10**	N	**P50**	N
**P11**	N	**P51**	Y
**P12**	Y	**P52**	Y
**P13**	N	**P53**	Y
**P14**	Y	**P54**	N
**P15**	N	**P55**	Y
**P16**	Y	**P56**	Y
**P17**	Y	**P57**	Y
**P18**	N	**P58**	Y
**P19**	Y	**P59**	Y
**P20**	N	**P60**	Y
**P21**	N	**P61**	Y
**P22**	N	**P62**	N
**P23**	Y	**P63**	Y
**P24**	N	**P64**	Y
**P25**	Y	**P65**	Y
**P26**	Y	**P66**	Y
**P27**	Y	**P67**	Y
**P28**	Y	**P68**	Y
**P29**	Y	**P69**	N
**P30**	Y	**P70**	N
**P31**	N	**P71**	Y
**P32**	N	**P72**	Y
**P33**	Y	**P73**	N
**P34**	Y	**P74**	N
**P35**	Y	**P75**	Y
**P36**	Y	**P76**	Y
**P37**	N	**P77**	Y
**P38**	Y	**P78**	Y
**P39**	Y	**P79**	Y
**P40**	Y	**P80**	Y

Supplementary Table 2 | Demographic information of healthy donor sera used in this study (*n* = 80).

	Sex	Age		Sex	Age		Sex	Age		Sex	Age
**N1**	M	70s	**N21**	F	80s	**N41**	M	30s	**N61**	M	30s
**N2**	M	60s	**N22**	F	40s	**N42**	M	20s	**N62**	M	30s
**N3**	M	70s	**N23**	F	60s	**N43**	M	20s	**N63**	F	20s
**N4**	F	60s	**N24**	F	40s	**N44**	M	40s	**N64**	F	50s
**N5**	F	60s	**N25**	F	70s	**N45**	F	30s	**N65**	F	30s
**N6**	F	60s	**N26**	M	80s	**N46**	M	40s	**N66**	F	30s
**N7**	M	70s	**N27**	F	70s	**N47**	M	50s	**N67**	F	30s
**N8**	M	70s	**N28**	F	70s	**N48**	F	40s	**N68**	M	30s
**N9**	M	60s	**N29**	M	60s	**N49**	M	40s	**N69**	M	30s
**N10**	F	50s	**N30**	M	70s	**N50**	F	40s	**N70**	F	20s
**N11**	F	50s	**N31**	M	70s	**N51**	F	50s	**N71**	M	30s
**N12**	M	60s	**N32**	M	70s	**N52**	F	30s	**N72**	M	30s
**N13**	M	60s	**N33**	M	60s	**N53**	F	40s	**N73**	M	40s
**N14**	M	50s	**N34**	F	70s	**N54**	M	30s	**N74**	F	40s
**N15**	M	70s	**N35**	M	60s	**N55**	M	30s	**N75**	F	50s
**N16**	M	80s	**N36**	F	60s	**N56**	F	30s	**N76**	F	20s
**N17**	M	40s	**N37**	F	50s	**N57**	F	50s	**N77**	F	20s
**N18**	M	70s	**N38**	M	60s	**N58**	M	30s	**N78**	M	40s
**N19**	F	70s	**N39**	M	50s	**N59**	F	30s	**N79**	M	30s
**N20**	F	70s	**N40**	F	50s	**N60**	M	40s	**N80**	M	30s

Supplementary Table 3 | Sequences of the target miRNAs and their complementary ssDNA probes used in this study.

Target miRNA	Length (nt)	Sequence (5′ → 3′)	ssDNA probe (5′ → 3′)
let-7a-5p	22	UGA GGU AGU AGG UUG UAU AGU U	AAC TAT ACA ACC TAC TAC CTC A-spacer-mal
let-7f-5p	22	UGA GGU AGU AGA UUG UAU AGU U	-
let-7g-5p	22	UGA GGU AGU AGU UUG UAC AGU U	-
miR-196a	22	UAG GUA GUU UCA UGU UGU UGG G	CCC AAC AAC ATG AAA CTA CCT A-spacer-mal
miR-1290	19	UGG AUU UUU GGA UCA GGG A	TCC CTG ATC CAA AAA TCC A-spacer-mal
miR-182-5p	24	UUU GGC AAU GGU AGA ACU CAC ACU	AGT GTG AGT TCT ACC ATT GCC AAA-spacer-mal
miR-223-3p	21	AGC UAC AUC UGG CUA CUG GGU	ACC CAG TAG CCA GAT GTA GCT-spacer-mal
miR-222-3p	22	UGU CAG UUU GUC AAA UAC CCC A	TGG GGT ATT TGA CAA ACT GAC A-spacer-mal
miR-106a-5p	23	AAA AGU GCU UAC AGU GCA GGU AG	CTA CCT GCA CTG TAA GCA CTT TT-spacer-mal

Supplementary Table 4 | Demographic, clinical, and histopathological characteristics and smoking status of GC patients (n = 40). (a) Sex, age, and clinical stage. **(b)** Histologic type classified according to the WHO Classification. **(c)** Smoking status (Y, current smoker; N, non-smoker).

(a)

	Sex	Age	Stage		Sex	Age	Stage
**P1**	M	50s	1	**P21**	M	50s	3
**P2**	M	50s	1	**P22**	M	60s	3
**P3**	F	60s	1	**P23**	M	60s	3
**P4**	M	50s	1	**P24**	F	80s	3
**P5**	M	70s	1	**P25**	M	50s	3
**P6**	M	40s	1	**P26**	F	70s	3
**P7**	F	60s	1	**P27**	M	70s	3
**P8**	M	60s	1	**P28**	F	70s	3
**P9**	M	60s	1	**P29**	M	80s	3
**P10**	M	50s	1	**P30**	F	50s	3
**P11**	F	60s	2	**P31**	F	60s	4
**P12**	M	80s	2	**P32**	F	50s	4
**P13**	M	50s	2	**P33**	M	60s	4
**P14**	M	60s	2	**P34**	M	70s	4
**P15**	M	60s	2	**P35**	M	80s	4
**P16**	F	40s	2	**P36**	M	50s	4
**P17**	F	80s	2	**P37**	M	50s	4
**P18**	F	60s	2	**P38**	F	50s	4
**P19**	M	80s	2	**P39**	M	60s	4
**P20**	M	60s	2	**P40**	M	70s	4

(b)

Patient	Histologic type	Patient	Histologic type
P1	Adenocarcinoma, poorly differentiated, with signet-ring cell component	P21	Poorly cohesive carcinoma, signet-ring cell type
P2	Poorly cohesive carcinoma, signet-ring cell type	P22	Adenocarcinoma, poorly differentiated
P3	Adenocarcinoma, poorly differentiated, with signet-ring cell component	P23	Adenocarcinoma, poorly differentiated
P4	Mucinous adenocarcinoma	P24	Adenocarcinoma, poorly differentiated
P5	Tubular adenocarcinoma, moderately differentiated	P25	Poorly cohesive carcinoma, signet-ring cell type
P6	Tubular adenocarcinoma, poorly differentiated	P26	Adenocarcinoma, poorly differentiated
P7	Adenocarcinoma, poorly differentiated	P27	Adenocarcinoma, mixed moderately and poorly differentiated
P8	Adenocarcinoma, poorly differentiated, with mucinous and focal signet-ring cell features	P28	Adenocarcinoma, poorly differentiated
P9	Adenocarcinoma, poorly differentiated	P29	Adenocarcinoma, poorly differentiated
P10	Tubular adenocarcinoma, moderately differentiated	P30	Adenocarcinoma, poorly differentiated
P11	Tubular adenocarcinoma, moderately differentiated	P31	Tubular adenocarcinoma, moderately differentiated
P12	Tubular adenocarcinoma, moderately differentiated	P32	Adenocarcinoma, poorly differentiated
P13	Tubular adenocarcinoma, moderately differentiated	P33	Tubular adenocarcinoma, moderately differentiated
P14	Tubular adenocarcinoma, moderately differentiated	P34	Tubular adenocarcinoma, moderately differentiated
P15	Tubular adenocarcinoma, moderately differentiated	P35	Adenocarcinoma, poorly differentiated
P16	Adenocarcinoma, poorly differentiated	P36	Adenocarcinoma, poorly differentiated
P17	Adenocarcinoma, poorly differentiated	P37	Adenocarcinoma, moderately differentiated
P18	Adenocarcinoma, poorly differentiated	P38	Poorly cohesive carcinoma, signet-ring cell type
P19	Tubular adenocarcinoma, moderately differentiated	P39	Tubular adenocarcinoma, moderately differentiated
P20	Adenocarcinoma, poorly differentiated	P40	Tubular adenocarcinoma, moderately differentiated

(c)

Patient	Smoking status	Patient	Smoking status
**P1**	Y	**P21**	Y
**P2**	Y	**P22**	Y
**P3**	N	**P23**	Y
**P4**	Y	**P24**	N
**P5**	Y	**P25**	Y
**P6**	Y	**P26**	N
**P7**	N	**P27**	Y
**P8**	Y	**P28**	N
**P9**	Y	**P29**	Y
**P10**	Y	**P30**	N
**P11**	N	**P31**	Y
**P12**	N	**P32**	Y
**P13**	Y	**P33**	Y
**P14**	N	**P34**	Y
**P15**	Y	**P35**	Y
**P16**	N	**P36**	Y
**P17**	N	**P37**	Y
**P18**	N	**P38**	N
**P19**	Y	**P39**	Y
**P20**	Y	**P40**	N

Supplementary Table 5 | Comparison of the present RNaseH1-dependent FRET platform with recent representative direct-detection platforms for circulating miRNAs in serum/plasma.

Reference	Workflow	Platform technology / Analytical instrument	LOD & Assay conditions	Assay selectivity	Clinical cohort
Nat. Commun. **12**, 3515 (2021)	- Unprocessed human serum<br>- Direct serum analysis	Single-molecule electro-optical nanopore	- LOD: 5–8 fM<br>(Test range of 0.1 to 10 pM for ~80 min)	- Single-/double-nucleotide mismatch discrimination	- PCa serum (n = 10) with 2-miRNA markers<br>- Localized (n = 3) metastatic (n = 3) remission (n = 7) with 3 miRNA markers
Anal. Chem. **94**, 14659 (2022)	- Human plasma<br>- Direct one-step<br>- Real-time, no extraction or amplification	Nanophotonic BiMW biosensor	- LOD: 25 pM<br>(0.5 to 100 nM tested for < 45 min)	- Non-complementary miR-210-3p shows no detectable response	- NSCLC plasma (n = 20) Healthy (n = 20) with 1 miRNA marker
ACS Sens. **8**, 1085 (2023)	- Unprocessed plasma<br>- Microsamples	High-throughput nanoplasmonic LSPR assay	- LOD: ~130 aM<br>(10 to 10^{11} aM tested for ~17 h)	- High specificity against non-complementary miRNA in plasma<br>- No single-base mismatch metric reported	- PDAC plasma (n = 75) Chronic Pancreatitis (n = 35) Healthy (n = 60) with 2 miRNA markers
Nat. Commun. **15**, 2901 (2024)	- Serum<br>- RNA extraction<br>- No target amplification	CRISPR-Cas13a Gemini System (fluorescence + lateral flow)	- LOD: 733 fM<br>(1 to 100 pM tested for ~60 min)	- Dual-target simultaneous detection	- Breast cancer serum (n = 30) healthy (n = 15) with 2 miRNA markers<br>- EBV detection (n = 75 positive, n = 52 negative) with 2 small RNA markers
Nat. Commun. **15**, 8342 (2024)	- Plasma/cells<br>- RNA extraction<br>- No reverse transcription or amplification	Split crRNA CRISPR/Cas12a (SCas12a) fluorescence + lateral flow	- LOD: ~100 fM<br>(10 to 10^6 fM tested for ~50 min)	- Single-point mutation discrimination in both DNA and miRNA<br>- Distinguishes mature miRNA from pre-miRNA	- Cervical cancer plasma (n = 7) Healthy (n = 3) with 1 miRNA marker<br>- HPV16 detection in vaginal secretion (n = 25)
**Present study**	- Serum<br>- No extraction/ purification/ amplification	RNaseH1-dependent FRET platform using off-the-shelf instruments (well plate, microplate reader)	- LOD: 5 fM<br>(10 to 10^9 fM tested for ~60 min, for 7 miRNA markers)	- Single- and double-mismatch discrimination<br>- Mismatch position-independent discrimination<br>- No cross-reactivity for non-target miRNAs	- NSCLC serum (n = 80) Healthy (n = 80) with 3 miRNA markers<br>- GC serum (n = 40) Healthy (n = 40) with 3 miRNA markers

Supplementary Table 6 | Analytical performance of the FRET platform for six clinical miRNA markers with varying lengths and sequences.

Cancer type	Target miRNA	Length	Calibration fit	R^2	LOD
NSCLC	miR-196a	22 nt	$y = 4.61\log x + 11.65$	0.969	~5 fM
NSCLC	miR-1290	19 nt	$y = 6.45\log x + 10.96$	0.996	~5 fM
NSCLC	miR-182-5p	24 nt	$y = 3.72\log x + 12.99$	0.988	~5 fM
GC	miR-223-3p	21 nt	$y = 5.09\log x + 10.86$	0.985	~5 fM
GC	miR-222-3p	22 nt	$y = 4.46\log x + 11.22$	0.974	~5 fM
GC	miR-106a-5p	23 nt	$y = 3.38\log x + 14.04$	0.989	~5 fM

Supplementary Table 7 | Raw Ct values, mean Ct, measured concentrations, background-corrected concentrations, recovery, and qPCR variability for synthetic miR-196a spiked into healthy human serum.

Added miR-196a (fM)	Ct1	Ct2	Ct3	Mean Ct ± s.d.	qPCR measured concentration (fM, mean ± s.d.)	Background-corrected concentration (fM)	Recovery (%)	Concentration CV (%)
0	33.49	33.86	33.57	33.64±0.19	0.20±0.03	NA	NA	9.88
1.0	31.36	31.1	31.26	31.24±0.13	1.05±0.10	0.85	84.45	9.84
2.5	30.02	30.21	29.98	30.07±0.12	2.34±0.19	2.14	85.60	11.70
5.0	29.41	29.07	29.18	29.22±0.17	4.22±0.49	4.02	80.29	8.28
7.5	28.58	28.84	28.59	28.67±0.15	6.15±0.61	5.96	79.33	9.12
10.0	28.11	28.33	28.37	28.27±0.14	8.10±0.80	7.9	79.01	12.97

Standard curve: Ct = -3.42 log10[C] + 22.35; R^2 = 0.998.
