# Supplementary material for: An RNase domain-dependent microRNA detection with DNA-spiked nanocage for accurate cancer diagnosis
Source: Nat Commun. 2026 Jul 29;17:9198. doi: 10.1038/s41467-026-76169-0 (PMC13522518; doi:10.1038/s41467-026-76169-0)
Supplement: Supplementary file 2 [file 41467_2026_76169_MOESM2_ESM.pdf]

nature portfolio

Corresponding author(s): Jeewon Lee

Last updated by author(s): July 15, 2026

Reporting Summary

Nature Portfolio wishes to improve the reproducibility of the work that we publish. This form provides structure for consistency and transparency in reporting. For further information on Nature Portfolio policies, see our Editorial Policies and the Editorial Policy Checklist.

Statistics

For all statistical analyses, confirm that the following items are present in the figure legend, table legend, main text, or Methods section.

n/a	Confirmed	
☐	☒	The exact sample size (*n*) for each experimental group/condition, given as a discrete number and unit of measurement
☐	☒	A statement on whether measurements were taken from distinct samples or whether the same sample was measured repeatedly
☐	☒	The statistical test(s) used AND whether they are one- or two-sided<br>*Only common tests should be described solely by name; describe more complex techniques in the Methods section.*
☒	☐	A description of all covariates tested
☐	☒	A description of any assumptions or corrections, such as tests of normality and adjustment for multiple comparisons
☐	☒	A full description of the statistical parameters including central tendency (e.g. means) or other basic estimates (e.g. regression coefficient) AND variation (e.g. standard deviation) or associated estimates of uncertainty (e.g. confidence intervals)
☐	☒	For null hypothesis testing, the test statistic (e.g. F, t, r) with confidence intervals, effect sizes, degrees of freedom and P value noted<br>*Give P values as exact values whenever suitable.*
☒	☐	For Bayesian analysis, information on the choice of priors and Markov chain Monte Carlo settings
☒	☐	For hierarchical and complex designs, identification of the appropriate level for tests and full reporting of outcomes
☐	☒	Estimates of effect sizes (e.g. Cohen's d, Pearson's r), indicating how they were calculated

Our web collection on statistics for biologists contains articles on many of the points above.

Software and code

Policy information about availability of computer code

Data collection	No custom code was used. Data were collected using the instrument manufacturers' software: i-control v1.7 (TECAN) for the Infinite M200 PRO microplate reader; ZEN 2012 black edition (Carl Zeiss) for the LSM 700 confocal microscope; Photal ELSZ-1000 software v5.01 (Otsuka Electronics) for the ELSZ-1000 DLS analyzer; CFX Manager v3.0 (Bio-Rad) for the CFX96 Touch Real-Time PCR Detection System; and Gatan DigitalMicrograph v3.9.4 for the Tecnai 20 TEM.
Data analysis	No custom code was used. Graphical data analysis and statistical analyses were performed using SigmaPlot 10.0, GraphPad Prism 10.6.1, and Origin 8.5. Protein structure prediction was performed using AlphaFold2 via ColabFold (Neurosnap web server; job submitted 2025-04-17). Structural analysis of DNA–RNA hybrids was performed using CURVES+ v3.0.<br>Molecular dynamics simulations were performed using GROMACS 2021.4 with the AMBER99SB-ILDN and CHARMM36-jul2022 force fields, and molecular docking was performed using AutoDock Vina 1.2.3. Molecular structures obtained from the simulations, the AlphaFold2-predicted FRET acceptor model, and the EGFP structure retrieved from PDB entry 2Y0G were visualized using PyMOL 3.1.4. The HBVC structure retrieved from PDB entry 1QGT was rendered using the RCSB PDB 3D View (web based; no versioned release).

For manuscripts utilizing custom algorithms or software that are central to the research but not yet described in published literature, software must be made available to editors and reviewers. We strongly encourage code deposition in a community repository (e.g. GitHub). See the Nature Portfolio guidelines for submitting code & software for further information.

Data

Policy information about availability of data

All manuscripts must include a data availability statement. This statement should provide the following information, where applicable:

- Accession codes, unique identifiers, or web links for publicly available datasets
- A description of any restrictions on data availability
- For clinical datasets or third party data, please ensure that the statement adheres to our policy

All data generated in this study that support the findings are available within the paper, its Supplementary Information, and the accompanying Source Data file. Specifically, source data underlying the graphs and statistical analyses are provided as a Source Data file, and the demographic, clinical, and histopathological characteristics of the serum specimens are provided in Supplementary Tables 1, 2, and 4. The protein structures used in this study are available in the Protein Data Bank under accession codes 2Y0G [https://doi.org/10.2210/pdb2y0g/pdb] (EGFP) and 1QGT [https://doi.org/10.2210/pdb1qgt/pdb] (hepatitis B virus capsid). Source data are provided with this paper.

Research involving human participants, their data, or biological material

Policy information about studies with human participants or human data. See also policy information about sex, gender (identity/presentation), and sexual orientation and race, ethnicity and racism.

Reporting on sex and gender	Reporting on sex and gender: Sex reported as male or female and age were provided by the KIRAMS Radiation Biobank at the time of sample distribution. These sex data were obtained from existing clinical/administrative records and were not self-reported for this study. Gender identity was not collected. Sex was not used as a stratification variable and no sex-based subgroup analysis was performed. This is because the study was designed to validate the analytical performance of the FRET-based diagnostic platform across clinical stages rather than to investigate sex-dependent differences in miRNA expression, and the subgroup sample sizes (e.g., per stage per sex) would have been insufficient to draw meaningful conclusions.
Reporting on race, ethnicity, or other socially relevant groupings	Race and ethnicity information was not collected or provided for the human serum samples used in this study, as these variables were not relevant to the study objectives.
Population characteristics	Serum specimens were obtained from 80 patients diagnosed with non-small-cell lung cancer (NSCLC), 40 patients diagnosed with gastric cancer, and 80 healthy donors through the Korea Institute of Radiological and Medical Sciences (KIRAMS) Radiation Biobank. For the gastric cancer analysis, sera from 40 of the 80 healthy donors were used as the control group. Limited demographic and clinical information, including age and sex for all participants and cancer stage, histopathological subtype, and smoking status for the patient cohorts, was provided by the KIRAMS Radiation Biobank. Full demographic and clinical, and histopathological characteristics are summarized in Supplementary Tables 1, 2, and 4.
Recruitment	Participants were not directly recruited for this study. Human serum samples were obtained through the Korea Institute of Radiological and Medical Sciences (KIRAMS) Radiation Biobank (KRB), and all samples were collected previously under biobank-approved protocols. Because participants were not recruited by the investigators, self-selection bias is unlikely. However, all specimens originated from a single biobank (KIRAMS Radiation Biobank), which may introduce selection bias with respect to the source population; validation in independent external cohorts therefore remains necessary, as noted in the Discussion.
Ethics oversight	The use of de-identified human serum specimens was reviewed and approved as exempt by the Institutional Review Board of Korea University (IRB Nos. KUIRB-2024-0439-04 and KUIRB-2026-0152-02). All specimens were obtained from the KIRAMS Radiation Biobank under biobank-approved protocols and were used in accordance with the Guidelines for the Care and Use of Human Biological Materials of Korea University.

Note that full information on the approval of the study protocol must also be provided in the manuscript.

Field-specific reporting

Please select the one below that is the best fit for your research. If you are not sure, read the appropriate sections before making your selection.

☒ Life sciences ☐ Behavioural & social sciences ☐ Ecological, evolutionary & environmental sciences

For a reference copy of the document with all sections, see nature.com/documents/nr-reporting-summary-flat.pdf

Life sciences study design

All studies must disclose on these points even when the disclosure is negative.

Sample size	No formal sample-size calculation was performed a priori. Sample sizes were determined by availability from the KIRAMS Radiation Biobank: 80 NSCLC patients, 40 gastric cancer patients, and 80 healthy donors. All FRET and RT-qPCR measurements were performed in technical triplicate. These sample sizes are considered sufficient because they are larger than the cohorts used in comparable amplification-free miRNA detection studies (Supplementary Table 5) and yielded narrow 95% confidence intervals for the AUC estimates of the three NSCLC markers (Fig. 5h).

Data exclusions	No data were excluded. All serum specimens passed the hemolysis assessment, with hemolysis indices below the predefined threshold of 2.0.
Replication	All FRET and RT-qPCR measurements were performed in technical triplicate. All attempts at replication were successful. Batch-to-batch reproducibility was confirmed using three independently produced donor/acceptor batches (Supplementary Fig. 12). In addition, the SDS-PAGE, TEM, and DLS analyses were each performed three times independently with similar results.
Randomization	Randomization was not relevant to this study. Samples were allocated into groups based on clinical diagnosis (NSCLC, gastric cancer, or healthy), not by random assignment.
Blinding	Blinding was not applicable to this study. The objective was to validate the analytical performance of a diagnostic platform using clinically characterized specimens with known diagnoses.

Reporting for specific materials, systems and methods

We require information from authors about some types of materials, experimental systems and methods used in many studies. Here, indicate whether each material, system or method listed is relevant to your study. If you are not sure if a list item applies to your research, read the appropriate section before selecting a response.

Materials & experimental systems

n/a	Involved in the study	
☐	☒	Antibodies
☒	☐	Eukaryotic cell lines
☒	☐	Palaeontology and archaeology
☒	☐	Animals and other organisms
☐	☒	Clinical data
☒	☐	Dual use research of concern
☒	☐	Plants

Methods

n/a	Involved in the study	
☒	☐	ChIP-seq
☒	☐	Flow cytometry
☒	☐	MRI-based neuroimaging

Antibodies

Antibodies used	HRP-conjugated anti-His tag antibody (His-probe Antibody (H-3) HRP; Santa Cruz Biotechnology, Cat. No. sc-8036 HRP) was used in this study at a dilution of 1:200
Validation	The antibody used in this study was validated by the manufacturer for the reported application, and validation information is available on the manufacturer's website.<br>https://www.scbt.com/p/his-probe-antibody-h-3

Clinical data

Policy information about clinical studies
All manuscripts should comply with the ICMJE guidelines for publication of clinical research and a completed CONSORT checklist must be included with all submissions.

Clinical trial registration	Not applicable. This is not a clinical trial.
Study protocol	Not applicable as this was not an interventional clinical trial. The use of de-identified banked serum specimens and the experimental assay protocol were reviewed by the Korea University IRB as described above.
Data collection	Serum specimens were collected and distributed by the KIRAMS Radiation Biobank. Upon receipt, specimens were aliquoted into single-use volumes, stored at −80 °C, thawed once immediately before assay, and assessed for hemolysis.
Outcomes	Diagnostic performance was evaluated by ROC curve analysis (AUC, sensitivity, specificity).

Plants

Seed stocks	Not applicable
Novel plant genotypes	Not applicable
Authentication	Not applicable
