# Supplementary material for: An RNase domain-dependent microRNA detection with DNA-spiked nanocage for accurate cancer diagnosis
Source: Nat Commun. 2026 Jul 29;17:9198. doi: 10.1038/s41467-026-76169-0 (PMC13522518; doi:10.1038/s41467-026-76169-0)
Supplement: Supplementary file 3 [file 41467_2026_76169_MOESM3_ESM.pdf]

nature portfolio

Peer Review File

An RNase domain-dependent microRNA detection with DNA-spiked nanocage for accurate cancer diagnosis

Corresponding Author: Professor Jeewon Lee

This file contains all reviewer reports in order by version, followed by all author rebuttals in order by version.

Version 0:

Reviewer comments:

Reviewer #1

(Remarks to the Author)
This manuscript "RNase-dependent microRNA detection with DNA-spiked nanocage for accurate cancer diagnosis" has described a RNaseH1-dependent amplification-free FRET based miRNA diagnosis method. The manuscript is focused on human lung cancer. By detecting serum miRNAs, the manuscript claims that the developed method enables accurate cancer diagnosis and discrimination down to single-base mismatch. The manuscript presents an interesting approach for a fine-tuned FRET platform for a sensitive detection of miRNAs. The data support some of the claims but not all. Using a stand-alone miRNA in clinical diagnosis is challenging due to overlapping roles of miRNAs in different diseases. Therefore, the manuscript does not address his fundamental challenge in the field. In other words, without being able to detect a panel of miRNAs associated with the cancer of interest, the reliability needed for disease diagnosis is hard to achieve. In addition, some of the claims made about other techniques should be revisited as detailed in the comments below:

· Reference 31 talks about the role of miR196a in various cancers and the miRNA. The point is that a miRNA can have overlapping roles with other cancers. Being able to detect miR196a in serum with 100% specificity does not necessarily mean it enables accurate detection of NSCLC as it can also be associated with other cancers. This challenge is not addressed in this manuscript.

· Similarly, Let-7a, although strongly associated with the non-small-cell lung cancer (NSCLC), is not exclusive to NSCLC. Again, same issue here as noted above.

· The paper mentions that the miRNA detection using qPCR is costly and potentially compromise accuracy. The method developed in this manuscript requires protein expression, protein purification, conjugation of a DNA probe to the protein, and other moving parts to be optimized such as establishment of a protein-based donor/acceptor system. Therefore, the complexity of the ssDNA-spiked green fluorescent protein nanocage (FRET donor) appears to be underemphasized in this manuscript.

· It is confusing but the claim around the LOD appears to be misleading. The LOD is estimated to be 5 fM (Fig. 3d) for let-7a but I was not able to locate any data showing detection of 5 fM miR196a. In addition, estimation of LOD using one miRNA does not necessarily mean that it applies to all miRNAs. Please see the point below.

· Not all miRNAs equally hybridize with the complementary DNA probes due to their own secondary structures, sequence composition etc. Without testing a good set of miRNAs covering diverse scenarios, the claims made here related to 'accurate detection of cancer' is premature.

Reviewer #2

(Remarks to the Author)
The manuscript describes the development of a novel RNase H1 hybrid binding domain (HBD)-based FRET platform utilizing DNA-spiked protein nanocages for the direct, amplification-free detection of microRNAs in serum. The authors detail the genetic engineering of viral capsids to display capture probes and the use of the HBD to specifically recognize DNA-RNA hybrids, allowing for the discrimination of single-nucleotide mismatches. The study applies this platform to the detection of miR-196a in clinical serum samples for the diagnosis of non-small cell lung cancer (NSCLC), reporting high

sensitivity and specificity. This work presents an innovative integration of protein nanotechnology and molecular recognition that could offer significant advantages over traditional PCR-based methods in terms of workflow simplicity. However, to fully support the claims of clinical utility and mechanistic robustness, several critical issues regarding sample size, experimental controls, and assay characterization need to be addressed.

Major Comments
1. The clinical validation currently includes only 40 patients with a distribution of 10 cases per clinical stage. For a study claiming significant "clinical diagnostic potential" and 100% sensitivity/specificity, this sample size is insufficient to draw statistically robust conclusions, particularly regarding the ability to accurately diagnose Stage I cancer against biological variance. The authors should either increase the sample size and provide a statistical power analysis.
2. The method relies on the direct incubation of probes with patient serum. Serum is a complex matrix containing active nucleases (RNases) and a high background of non-specific nucleic acids and proteins. The manuscript lacks sufficient data demonstrating how the assay prevents the degradation of the target miRNA or the DNA probe by serum RNases during the incubation, and how non-specific binding events are excluded to ensure the signal is derived solely from the specific target.
3. In Figure 3, the authors demonstrate discrimination of single and double base mismatches; however, the sensitivity of the HBD is known to be sensitive to the structural perturbation of the helix. The authors should investigate and discuss whether the position of the mismatch affects the HBD binding affinity and the resulting FRET signal, as this is critical for the general applicability of the assay.
4. The rationale for selecting miR-196a needs stronger justification; while upregulated in NSCLC, it is not a unique marker for this malignancy. To demonstrate the versatility and robustness of the platform, the authors should provide data showing the detection of other low-abundance miRNAs or targets relevant to other conditions, proving the platform is not limited to this specific sequence.
5. The current data relies on a single miRNA length and sequence model. To validate the methodological advantage and structural compatibility of the nanocage-HBD system, the authors should test miRNAs of varying lengths and sequences to ensure that the steric constraints of the nanocage do not hinder the detection of different targets.
6. While the study focuses on NSCLC, the discussion would benefit from a broader evaluation of the method's generalizability to other diseases or sample types to substantiate the claimed universality of the platform.
7. There is a visual discrepancy in Figures 5a and 5b between the described sample size (10 patients per group) and the plotted data, which appears to show a density of points exceeding the stated N number. The authors must clarify this inconsistency, ensure the plots accurately reflect the individual data points, or explain the data visualization method used.

Minor comment:
1. The title "RNase-dependent microRNA detection" is misleading because the assay utilizes the Hybrid Binding Domain (HBD) of RNase H1 for structural recognition, not the enzymatic cleavage activity of the RNase. The title and corresponding text should be revised to accurately reflect that this is an HBD-binding-based assay to avoid confusion regarding the mechanism.

Reviewer #3

(Remarks to the Author)
Summary
This manuscript reports an RNaseH1-dependent, amplification-free FRET platform for detecting circulating microRNAs directly in serum. The system combines a DNA probe–decorated fluorescent HBV capsid nanocage (donor) with an RNaseH1 HBD–mOrange fusion protein (acceptor), with the goal of enabling highly specific, mismatch-sensitive miRNA detection. The authors report a limit of detection of ~5 fM and claim 100% sensitivity and 100% specificity in distinguishing 40 NSCLC patients from 38 healthy controls based on serum miR-196a levels.
The conceptual framework is interesting and technically sophisticated. However, the clinical and diagnostic claims are overstated, and key validation steps are missing. In its current form, the manuscript does not provide sufficient evidence to support the strength of its conclusions.

Major Concerns
1. Diagnostic Performance Is Overstated
The claim of 100% sensitivity and 100% specificity is not adequately supported. The cohort size is modest (40 patients and 38 controls), yet no ROC curves, AUC values, confidence intervals, or validation procedures are provided. Without proper diagnostic statistics and independent validation, the reported performance cannot be considered robust. As presented, the data suggest separation between groups, but this is not equivalent to establishing clinical diagnostic accuracy.
2. Comparison to RT-qPCR Is Not Convincing
The manuscript suggests that this platform outperforms RT-qPCR, particularly in early-stage disease. However, the RT-qPCR methodology is insufficiently detailed. There is no clear description of normalization strategy, technical replicates, recovery controls, or assay variability. Without a rigorous and well-controlled comparison, the claim of improved sensitivity over RT-qPCR is not persuasive.
3. Lack of Interference and Matrix Controls
The assay is described as working directly in serum without extraction. However, no data are provided to address common confounders such as hemolysis, RNase activity, sample storage conditions, freeze-thaw cycles, or nonspecific serum effects. Given the complexity of serum, the absence of interference testing is a significant weakness, especially for a study that emphasizes clinical applicability.

The manuscript does not adequately acknowledge or discuss prior reports demonstrating direct detection of miRNAs in raw plasma or serum samples. There are multiple published platforms that claim amplification-free or minimal-processing

detection of circulating miRNAs. The current manuscript does not provide a meaningful comparison of performance metrics such as limit of detection, dynamic range, assay time, specificity, or clinical validation scale. Without a clear and balanced discussion positioning this platform relative to existing direct-detection technologies, it is difficult to assess the true novelty and advantage of the proposed system. The manuscript would benefit substantially from a critical discussion of how this approach differs mechanistically and practically from prior amplification-free miRNA detection strategies. At present, the claim of conceptual and clinical superiority is not sufficiently justified in the context of the existing literature.

4. Mechanistic Claims Rely Heavily on Simulations
The mismatch discrimination mechanism is supported primarily by molecular dynamics simulations and docking analyses. While these are well executed, computational modeling cannot replace experimental validation of binding geometry and kinetics. Direct biophysical measurements (e.g., SPR or ITC) would substantially strengthen the mechanistic claims.

5. Reproducibility and Practical Considerations
There is no information on batch-to-batch reproducibility, inter-assay variability, stability of the nanocage constructs, or scalability of production. These issues are critical for any diagnostic platform but are not addressed.

Minor Points
• Statistical methods are not described in sufficient detail.
• The language throughout the manuscript is overly definitive relative to the data presented.
• The discussion does not sufficiently position this work relative to other amplification-free miRNA detection technologies, including CRISPR-based systems.

Version 1:

Reviewer comments:

Reviewer #1

(Remarks to the Author)
The authors have tested three instead of one miRNA initially to validate the detection assay. The authors also addressed my other comments satisfactorily. The manuscript has been much improved after revision.

Reviewer #2

(Remarks to the Author)
All my concerns have been adequately addressed. I recommend publication of this work.

Reviewer #3

(Remarks to the Author)
The revised manuscript is improved and addresses many of the major concerns raised previously. The added mechanistic studies, mismatch analyses, reproducibility experiments, and expanded methodological details strengthen the work significantly. The amplification-free FRET platform for direct serum miRNA detection is innovative and potentially important for cancer diagnostics.
However, minor issues should still be addressed before publication:
1. More clinical information should be provided for the patient cohorts, including smoking status, histology, treatment status, and possible confounding factors.
2. The manuscript should acknowledge the lack of an independent external validation cohort as a limitation.
3. Claims that the platform outperforms RT-qPCR and other methods should be moderated unless directly validated under comparable conditions.
4. The distinction between technical and biological replicates should be clarified throughout the manuscript.
5. Additional details are needed for statistical analyses, including testing for normality, handling of outliers, and multiple-comparison corrections.
6. The Discussion should better address potential serum matrix effects and assay variability in real clinical samples.
7. Specificity testing against additional closely related miRNAs would strengthen the conclusions, or this limitation should be acknowledged.
8. Some statements regarding clinical applicability appear overstated and should be toned down.
9. The ROC cutoff analysis should clarify whether cutoff determination and testing were performed using the same cohort.
10. Minor language editing and simplification of several long sentences would improve readability.
Overall, the manuscript is much improved and suitable for publication after minor revision.

Open Access This Peer Review File is licensed under a Creative Commons Attribution 4.0 International License, which permits use, sharing, adaptation, distribution and reproduction in any medium or format, as long as you give appropriate credit to the original author(s) and the source, provide a link to the Creative Commons license, and indicate if changes were made.
In cases where reviewers are anonymous, credit should be given to 'Anonymous Referee' and the source.
The images or other third party material in this Peer Review File are included in the article's Creative Commons license, unless indicated otherwise in a credit line to the material. If material is not included in the article's Creative Commons license and your intended use is not permitted by statutory regulation or exceeds the permitted use, you will need to obtain permission directly from the copyright holder.
To view a copy of this license, visit https://creativecommons.org/licenses/by/4.0/

KOREA UNIVERSITY

Revision Details (NCOMMS-26-005397-T)

Reviewer #1:

This manuscript "RNase-dependent microRNA detection with DNA-spiked nanocage for accurate cancer diagnosis" has described a RNaseH1-dependent amplification-free FRET based miRNA diagnosis method. The manuscript is focused on human lung cancer. By detecting serum miRNAs, the manuscript claims that the developed method enables accurate cancer diagnosis and discrimination down to single-base mismatch. The manuscript presents an interesting approach for a fine-tuned FRET platform for a sensitive detection of miRNAs. The data support some of the claims but not all. Using a stand-alone miRNA in clinical diagnosis is challenging due to overlapping roles of miRNAs in different diseases. Therefore, the manuscript does not address his fundamental challenge in the field. In other words, without being able to detect a panel of miRNAs associated with the cancer of interest, the reliability needed for disease diagnosis is hard to achieve. In addition, some of the claims made about other techniques should be revisited as detailed in the comments below:

Major comments:

[Comment 1]
Reference 31 talks about the role of miR196a in various cancers and the miRNA. The point is that a miRNA can have overlapping roles with other cancers. Being able to detect miR196a in serum with 100% specificity does not necessarily mean it enables accurate detection of NSCLC as it can also be associated with other cancers. This challenge is not addressed in this manuscript.

Revision:

In this study, to evaluate the efficacy of FRET platform in clinical cancer diagnosis, we used NSCLC patient cohort and attempted to directly detect miR-196a in the patient sera. That is, miR-196a was used as a model marker representing the malignancy of NSCLC. We fully agree that reliability of FRET platform in accurate cancer diagnosis is not sufficiently supported by the diagnostic results presented in the original manuscript, because one miRNA has overlapping roles with multiple cancer types. Reportedly, it is well established that individual circulating miRNAs reflect shared oncogenic pathways rather than organ-specific signatures, and any single circulating miRNA is not exclusively

specific to a particular cancer type (*Clin. Chem.* **61**, 1138-1155 (2015); *Biomark. Res.* **9**, 18 (2021)). This is the reason why recent studies concerning miRNA detection for cancer diagnosis have employed multiple miRNA markers – miR-141-3p and miR-375-3p for prostate cancer diagnosis (*Nat. Commun.* **12**, 3515 (2021)) and miR-155 and miR-375 for breast cancer diagnosis (*Nat. Commun.* **15**, 2901 (2024)) – although the miRNA markers above are associated with multiple cancer malignancies.

According to the reviewer's comment, we tried to detect a panel of miRNAs in the diagnosis of NSCLC – including two additional markers, or miR-1290 and miR-182-5p alongside miR-196a – in the manuscript revision. The full sequences of the three miRNA markers and their complementary ssDNA probes are summarized below (Supplementary Table 3 of the revised manuscript).

Target miRNA	Length (nt)	Sequence (5' → 3')	ssDNA probe (5' → 3')
let-7a	22	UGA GGU AGU AGG UUG UAU AGU U	AAC TAT ACA ACC TAC TAC CTC A-spacer (C6)-mal
let-7f	22	UGA GGU AGU AGA UUG UAU AGU U	-
let-7g	22	UGA GGU AGU AGU UUG UAC AGU U	-
miR-196a	22	UAG GUA GUU UCA UGU UGU UGG G	CCC AAC AAC ATG AAA CTA CCT A-spacer (C6)-mal
miR-1290	19	UGG AUU UUU GGA UCA GGG A	TCC CTG ATC CAA AAA TCC A-spacer (C6)-mal
miR-182-5p	24	UUU GGC AAU GGU AGA ACU CAC ACU	AGT GTG AGT TCT ACC ATT GCC AAA-spacer (C6)-mal
miR-223-3p	21	AGC UAC AUC UGG CUA CUG GGU	ACC CAG TAG CCA GAT GTA GCT-spacer (C6)-mal
miR-222-3p	22	UGU CAG UUU GUC AAA UAC CCC A	TGG GGT ATT TGA CAA ACT GAC A-spacer (C6)-mal
miR-106a-5p	23	AAA AGU GCU UAC AGU GCA GGU AG	CTA CCT GCA CTG TAA GCA CTT TT-spacer (C6)-mal

Although these miRNAs are dysregulated in other malignancies, we selected these markers based on the robust clinical evidences reported in previous literature, as described in detail as follows. In a discovery cohort of NSCLC patients, miR-1290 was the most significantly upregulated marker among a panel of six exosomal miRNAs and subsequently demonstrated the highest diagnostic performance, outperforming conventional serum protein markers such as CEA and CYFRA21-1 (*Onco Targets Ther.* **13**, 7809–7818 (2020)). Its overexpression and association with advanced TNM (tumor, node, and metastasis) stage of NSCLC and poor prognosis were independently confirmed in a separate cohort (*J. Thorac. Dis.* **7**, 1570–1579 (2015)). miR-182-5p was identified as consistently upregulated in NSCLC patients through large-scale database analyses and validated using multiple independent, NSCLC tissue cohorts (*J. Cancer* **12**, 7120–7129 (2021); *BMC Med. Genomics* **13**, 3 (2020)). Notably, it was demonstrated that serum miR-182-5p levels in NSCLC patients were distinguishable not only from healthy controls but also from pneumonia patients, gastric cancer patients, and smokers (*PLoS*

ONE **11**, e0153046 (2016)). miR-196a was reported to be significantly overexpressed in NSCLC tissues and serum, correlating with advanced stage and lymph node metastasis (*Int. J. Clin. Exp. Pathol.* **11**, 773–780 (2018)).

The additional experiments performed in the manuscript revision are described in detail below. After each of the NSCLC-associated, three miRNA markers above was spiked in a healthy serum at 10 to 10^5 fM, the detection signals (FRET ratios) by FRET platform were measured at each concentration of each marker. The correlation between FRET ratio (%) and miRNA concentration was estimated for each miRNA marker, giving the calibration plots with excellent linearity as attached below (Supplementary Fig. 19 of the revised manuscript).

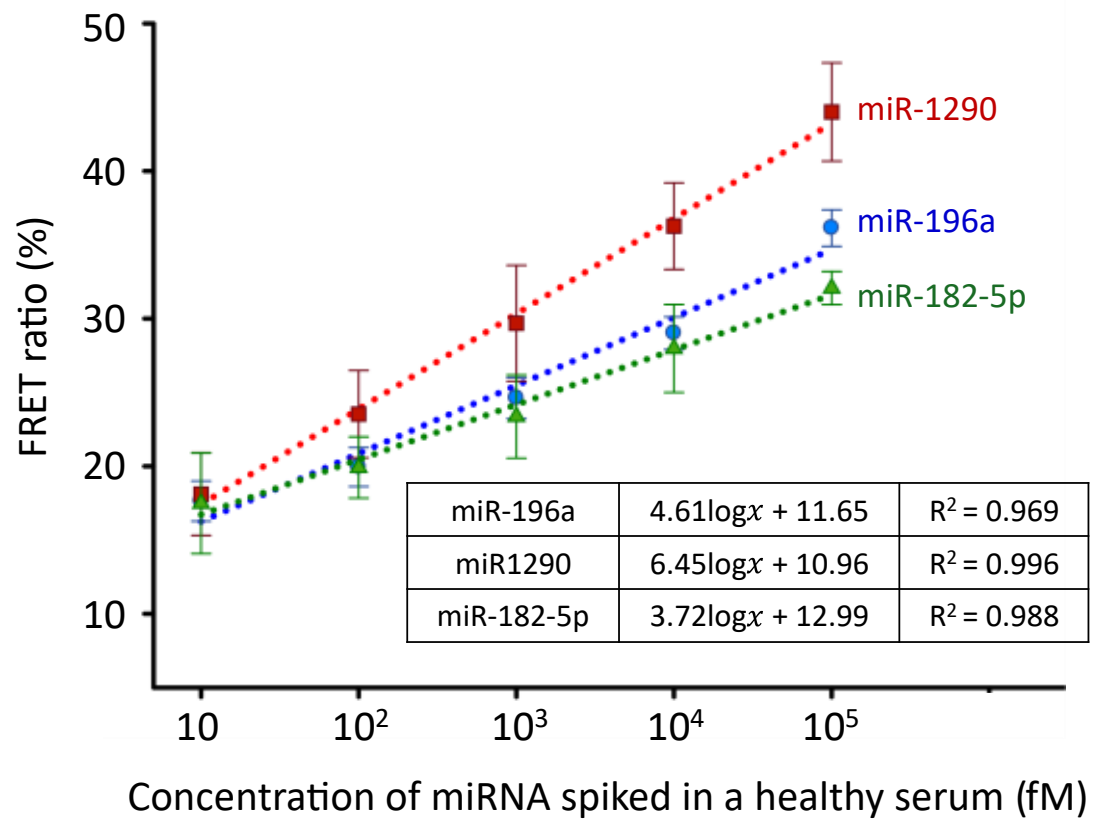

Based on the above correlations, we estimated limit of detection (LOD) in the detection of each miRNA marker by FRET platform, showing that LOD is ~ 5 fM for all three miRNA markers as seen in the plots below (Supplementary Fig. 20 of the revised manuscript), which is below the concentration range of circulating miRNAs in the blood of cancer patients (*Nat. Commun.* **15**, 2901 (2024), *Nat. Commun.* **15**, 8342 (2024), *Anal. Chem.* **94**, 14659 (2022)).

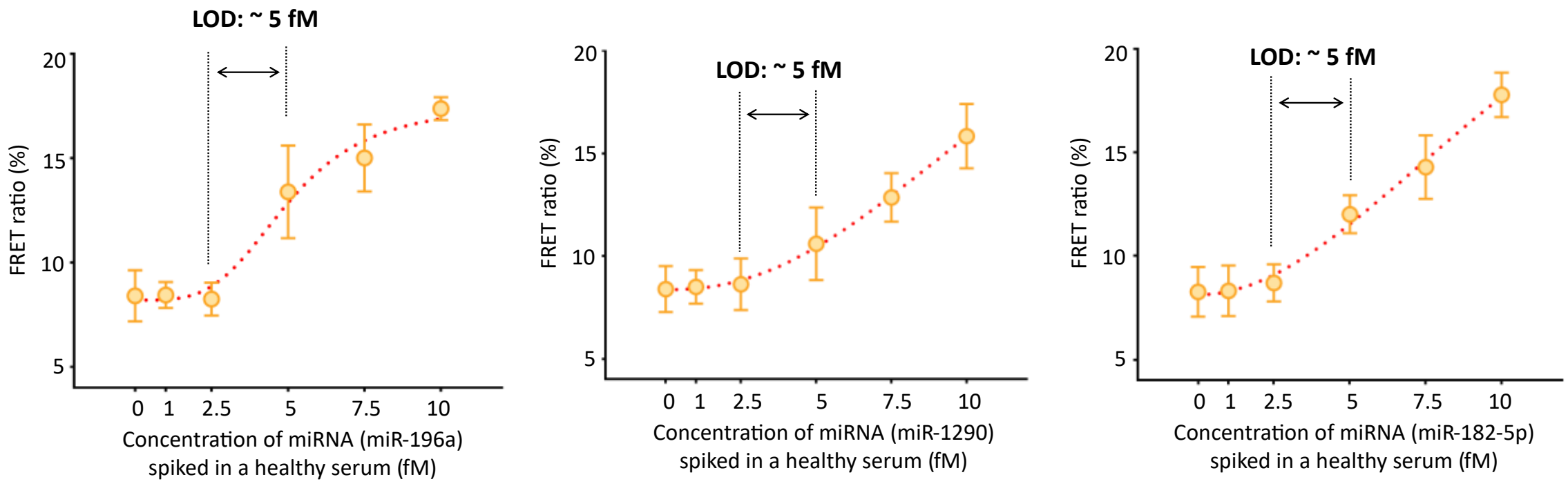

In addition, we expanded the cohort size by adding 40 more patients (15, 15, 5, and 5 patients at stage I, II, III, and IV, respectively) and 42 more healthy controls, that is, we tested totally 80 NSCLC patients (25, 25, 15, and 15 patients at stage I, II, III, and IV, respectively) and 80 healthy donors. The miRNA levels in the patient sera, measured by both RT-qPCR and FRET platform, are presented in the box plots below (Figs. 5a-f of the revised manuscript). Notably, in the detection of all three miRNA markers (miR-196a (Figs. 5a,b), miR-1290 (Figs. 5c,d), and miR-182-5p (Figs. 5e,f)), the readout signals (FRET ratios) from FRET platform for 80 patients are clearly distinguished from those for healthy controls. Notably, compared to RT-qPCR, the much larger difference in readout signals between the stage I- and II patients and healthy group was observed in the diagnosis by FRET platform, suggesting that FRET platform is likely more effective in early diagnosis of NSCLC.

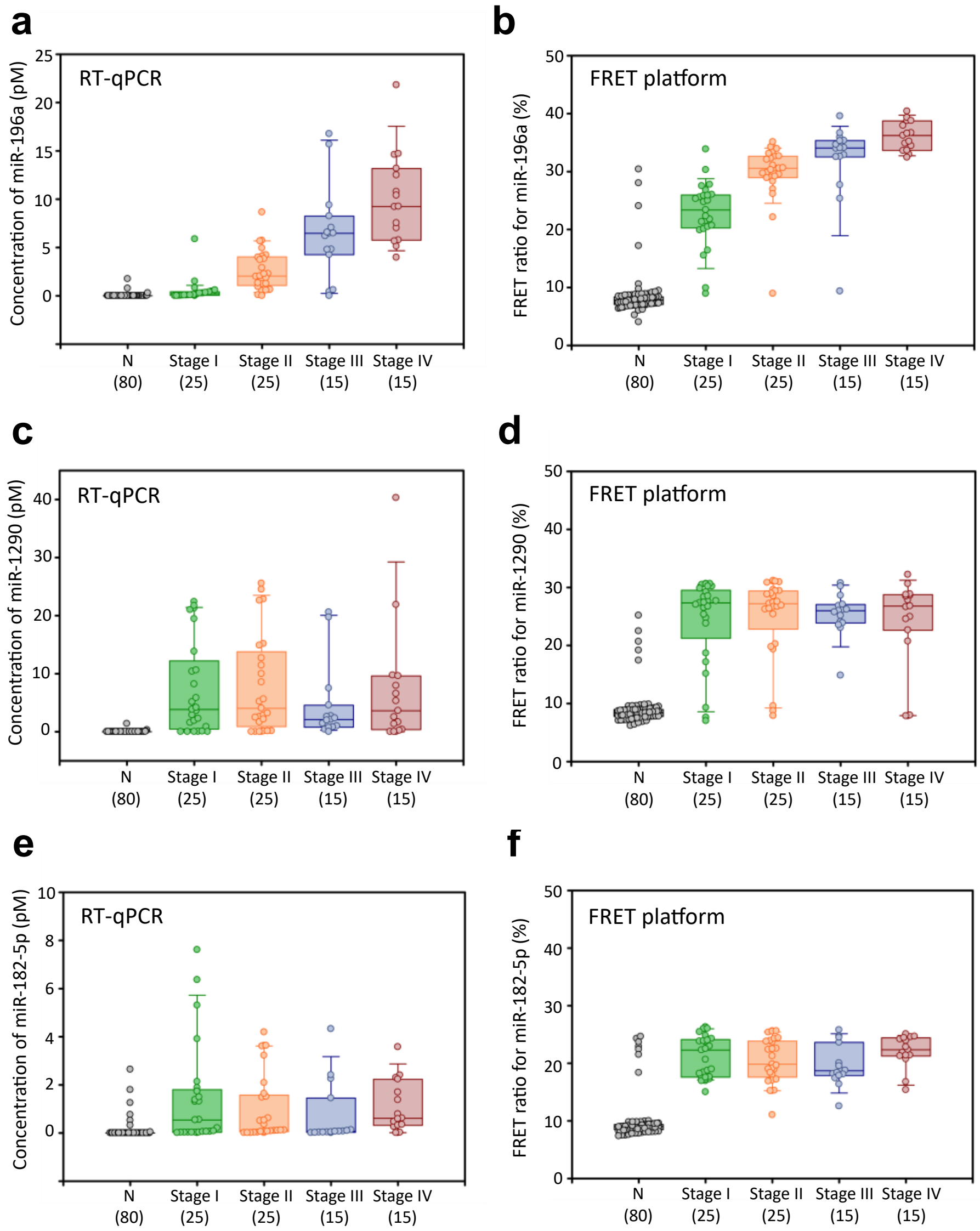

Further, as seen in the heat maps below (Fig. 5g of the revised manuscript), which are plotted using the same readout signals of the box plots above (Figs. 5a-f), the signal pattern from FRET platform and qRT-PCR looks very similar.

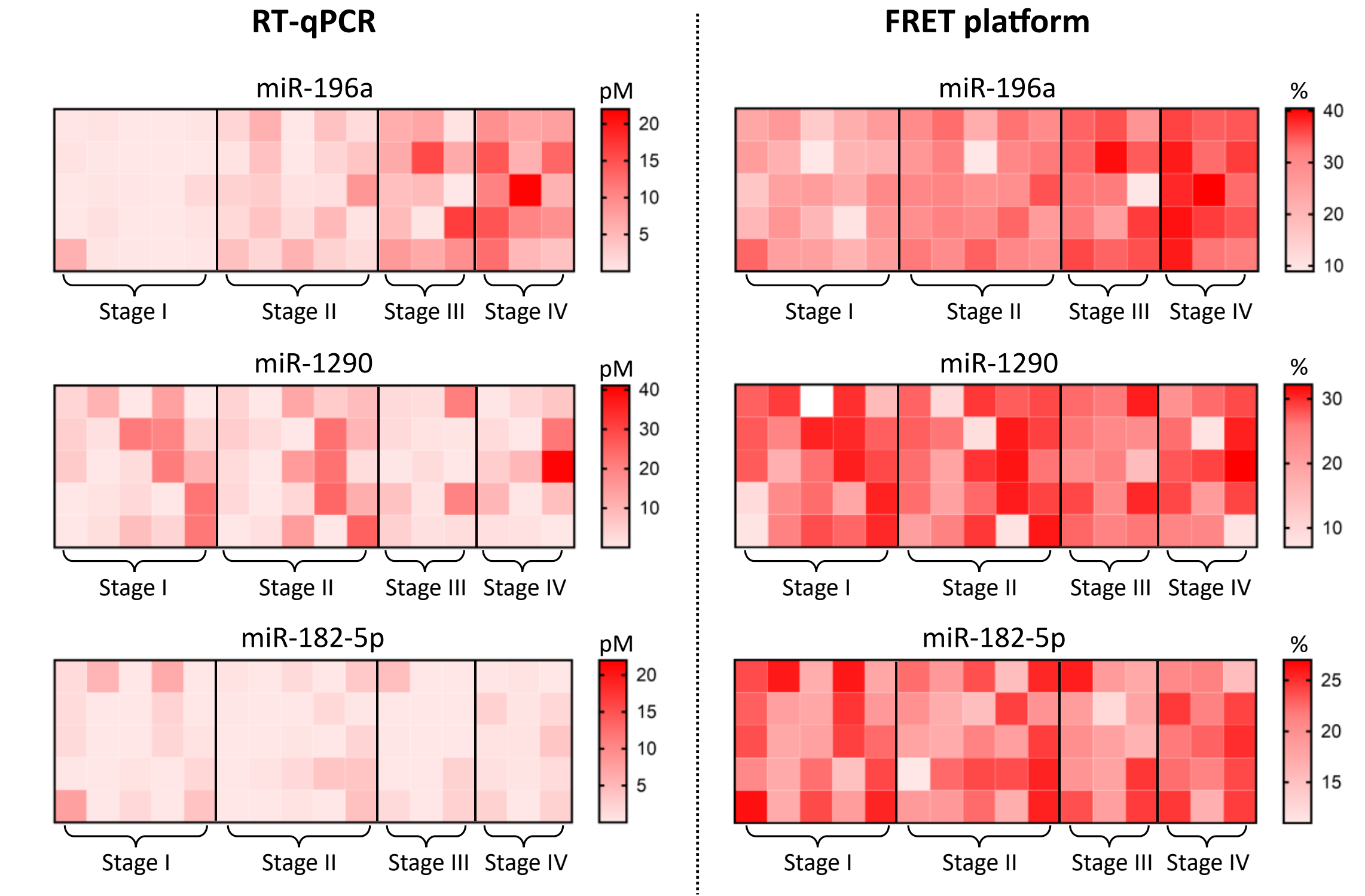

To assess the agreement in readout signal pattern between FRET platform and RT-qPCR, Spearman's rank correlation coefficients (ρ) were calculated for each miRNA marker. As presented below (Supplementary Fig. 21 of the revised manuscript), the ρ values were 0.8675, 0.8408, and 0.9212 for miR-196a, miR-1290, and miR-182-5p, respectively ($p < 0.0001$ for all calculations), all of which are sufficiently high values to indicate strong, monotonic correlations in the diagnostic signals between FRET platform and RT-qPCR (*Nat. Commun.* **16**, 2610 (2025); *Anesth. Analg.* **126**, 1763–1768 (2018)), suggesting that FRET platform is a quantitatively reliable diagnostic tool like established RT-qPCR.

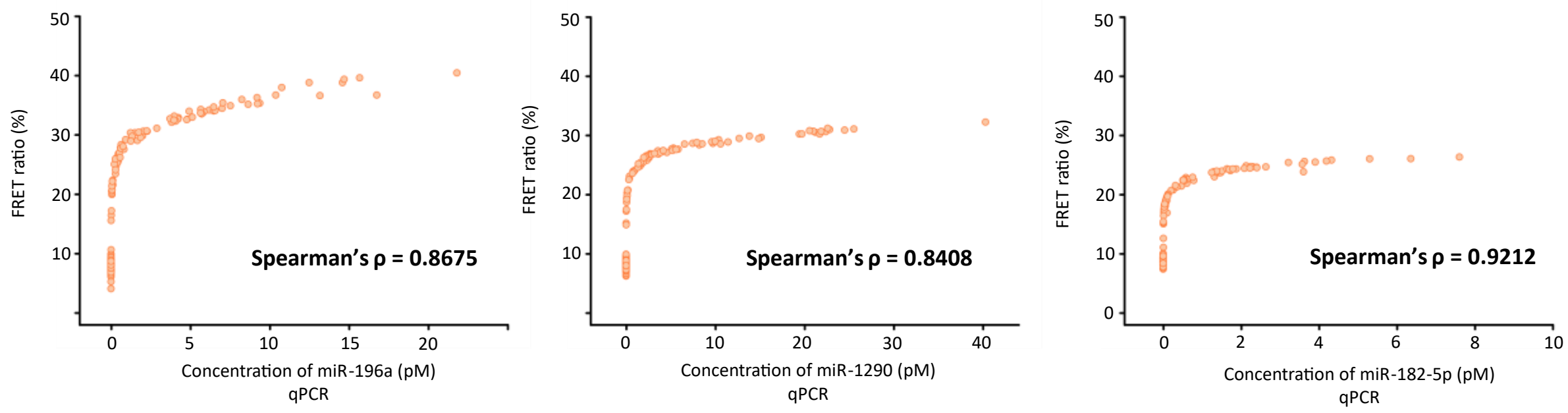

Further, to evaluate clinical sensitivity and specificity of FRET platform, ROC (Receiver Operating Characteristic) curve analysis was performed for each miRNA marker as presented in the plot below (Fig. 5h of the revised manuscript).

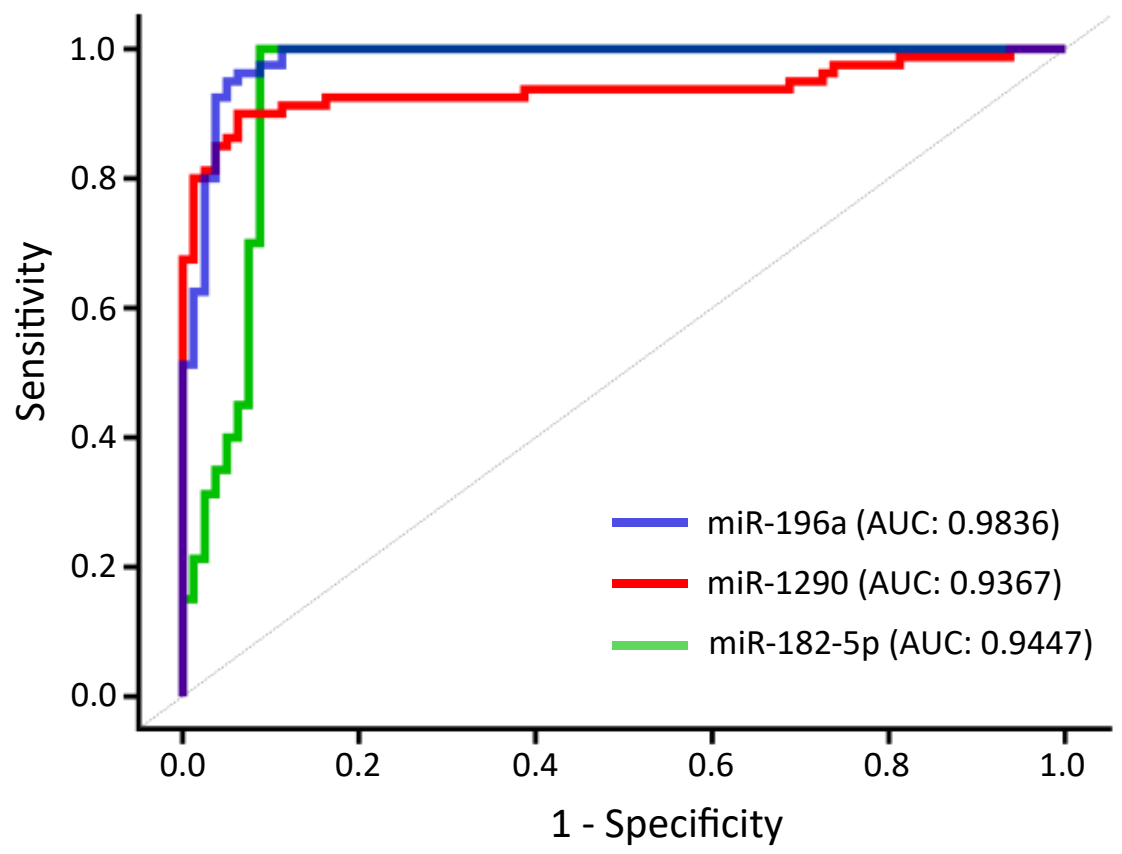

The AUC (Area Under the Curve) values were high enough – 0.9836 (95% confidence interval (CI): 0.9669~1.000), 0.9367 (95% CI: 0.8931~0.9804), and 0.9447 (95% CI: 0.9033~0.9860) for miR-196a, miR-1290, and miR-182-5p, respectively – to claim that FRET platform is such a reliable diagnostic method that well distinguishes between patient- and healthy groups. Also, using Youden index (J = sensitivity + specificity − 1), which is a widely used criterion for selecting a cutoff point that maximizes overall classification performance in ROC analysis (*Nat. Commun.* **15**, 10213 (2024); *BMC Med. Res. Methodol.* **24**, 84 (2024)), we estimated the optimal cutoff of FRET ratios to be 13.05%, 12.35%, and 11.06% for miR-196a, miR-1290, and miR-182-5p, respectively, through calculating J at every possible threshold along the ROC curve to find an optimal cutoff that yields maximum J. Based on the cutoff values above, clinical sensitivity and specificity in the diagnosis of 80 NSCLC patients and 80 healthy donors were estimated: 95.0 and 95.0 %, respectively, for miR-196a; 90.0 and 93.8%, respectively, for miR-1290; and 98.8 and 91.3 %, respectively, for miR-182-5p, demonstrating that FRET platform enables significantly better, improved diagnostic performance as compared to the other recent reports on cancer diagnosis [sensitivity and specificity: 86.7, 93.3 % for breast cancer using CRISPR-Cas13a Gemini System (*Nat. Commun.* **15**, 2901 (2024)); 81.3, 90.7 % for lung cancer using RT-qPCR (*Proc. Natl. Acad. Sci. U.S.A.* **117**, 25036 (2020)); 89.0, 91.0 % (PDAC/pancreatic ductal adenocarcinoma using RT-qPCR (*Gastroenterology* **163**, 1252 (2022))].

The above results were discussed in the section "Clinical diagnosis of human lung and gastric cancer" of Results (p. 12-15) and in Conclusion (p. 16) of the revised manuscript.

[Comment 2]

Similarly, Let-7a, although strongly associated with the non-small-cell lung cancer (NSCLC), is not exclusive to NSCLC. Again, same issue here as noted above.

Revision:

In this study, we used let-7a and its family miRNAs (let-7f and let-7g) as model miRNA markers for estimating LOD of FRET platform (Fig. 3) and investigating its capability of discriminating mismatched hybridization between ssDNA probes and miRNAs (Fig. 4), NOT for the clinical diagnosis of NSCLC. For NSCLC diagnosis (Fig. 5), we used different miRNA markers, i.e., miR-196a, miR-1290, and miR-182-5p that reportedly are closely associated with NSCLC as stated in the response to Comment 1 above.

[Comment 3]

The paper mentions that the miRNA detection using qPCR is costly and potentially compromise accuracy. The method developed in this manuscript requires protein expression, protein purification, conjugation of a DNA probe to the protein, and other moving parts to be optimized such as establishment of a protein-based donor/acceptor system. Therefore, the complexity of the ssDNA-spiked green fluorescent protein nanocage (FRET donor) appears to be underemphasized in this manuscript.

Revision:

Like other analytical methods such as RT-qPCR and ELISA that require prior preparation of assay components (reverse transcriptase, DNA polymerase, sequence-specific DNA primers, *etc*. for RT-qPCR and polyclonal- and monoclonal primary antibodies, enzyme-conjugated secondary antibodies, *etc*. for ELISA, all of which must be highly purified after recombinant expression or DNA synthesis), it is inevitable to prepare ssDNA probe-spiked FRET donor and RNaseH1 HBD-linked FRET acceptor prior to applying FRET platform to diagnosis. Of course, in general, various assay components for analytical methods including FRET platform are prepared in a large quantity at a time, stably stored as a long-term stock, and repeatedly used to manufacture assay kits until they are expired.

It should be noted that a notable, marked difference between FRET platform and RT-qPCR is that FRET platform enables direct serum analysis through following a simple mix-and-read protocol without additional steps for miRNA extraction, reverse transcription, and DNA amplification and nevertheless differentiates more clearly stage I- and II patients from healthy group as well as discriminates a single nucleotide mismatch.

According to the reviewer's comment, we stated that the recombinant expression and purification of FRET donor and acceptor are necessary to construct FRET platform in the text of revised manuscript (p. 17).

[Comment 4]

It is confusing but the claim around the LOD appears to be misleading. The LOD is estimated to be 5 fM (Fig. 3d) for let-7a but I was not able to locate any data showing detection of 5 fM miR196a. In addition, estimation of LOD using one miRNA does not necessarily mean that it applies to all miRNAs.

Revision:

To address this concern, we estimated LOD for all three miRNA markers (miR-196a, miR-1290, and miR-182-5p) used in the NSCLC diagnosis by FRET platform, showing that the LOD is ~ 5 fM for all markers (Supplementary Figs. 20, 23 of the revised manuscript). Please see the responses to Comments 1 and 5, where we discussed these results in detail.

[Comment 5]

Not all miRNAs equally hybridize with the complementary DNA probes due to their own secondary structures, sequence composition etc. Without testing a good set of miRNAs covering diverse scenarios, the claims made here related to 'accurate detection of cancer' is premature.

Revision:

Target miRNA	Length (nt)	Sequence (5' → 3')	ssDNA probe (5' → 3')
let-7a	22	UGA GGU AGU AGG UUG UAU AGU U	AAC TAT ACA ACC TAC TAC CTC A-spacer (C6)-mal
let-7f	22	UGA GGU AGU AGA UUG UAU AGU U	-
let-7g	22	UGA GGU AGU AGU UUG UAC AGU U	-
miR-196a	22	UAG GUA GUU UCA UGU UGU UGG G	CCC AAC AAC ATG AAA CTA CCT A-spacer (C6)-mal
miR-1290	19	UGG AUU UUU GGA UCA GGG A	TCC CTG ATC CAA AAA TCC A-spacer (C6)-mal
miR-182-5p	24	UUU GGC AAU GGU AGA ACU CAC ACU	AGT GTG AGT TCT ACC ATT GCC AAA-spacer (C6)-mal
miR-223-3p	21	AGC UAC AUC UGG CUA CUG GGU	ACC CAG TAG CCA GAT GTA GCT-spacer (C6)-mal
miR-222-3p	22	UGU CAG UUU GUC AAA UAC CCC A	TGG GGT ATT TGA CAA ACT GAC A-spacer (C6)-mal
miR-106a-5p	23	AAA AGU GCU UAC AGU GCA GGU AG	CTA CCT GCA CTG TAA GCA CTT TT-spacer (C6)-mal

In the revision of manuscript, we tested 5 more miRNA markers with different sequences and lengths (19 to 24 nt) in addition to let-7 family (let-7a, let-7f, and let-7g) and miR-

196a: miR-1290 and miR-182-5p (NSCLC-associated markers, Fig. 5) and miR-223-3p, miR-222-3p, and miR-106a-5p (gastric cancer (GC)-associated markers, Supplementary Figs. 24, 25). In total, we tested 9 miRNA markers to verify diagnostic performance of FRET platform. The sequences, lengths, and complementary ssDNA probes for the 9 miRNAs are listed in Supplementary Table 3 attached above.

First, we examined if any significant non-specific binding happens between ssDNA probes of FRET donor and serum miRNAs in the diagnosis of NSCLC and GC through cross-reactivity for non-target miRNAs. The figure below (Supplementary Figure 18 of the revised manuscript) shows that each ssDNA probe emits the highest readout signal (FRET ratio) for its complementary target miRNA, whereas the readout signals for the other non-target miRNAs are quantitatively negligible, indicating that FRET platform enables highly selective, target-specific detection of miRNA markers.

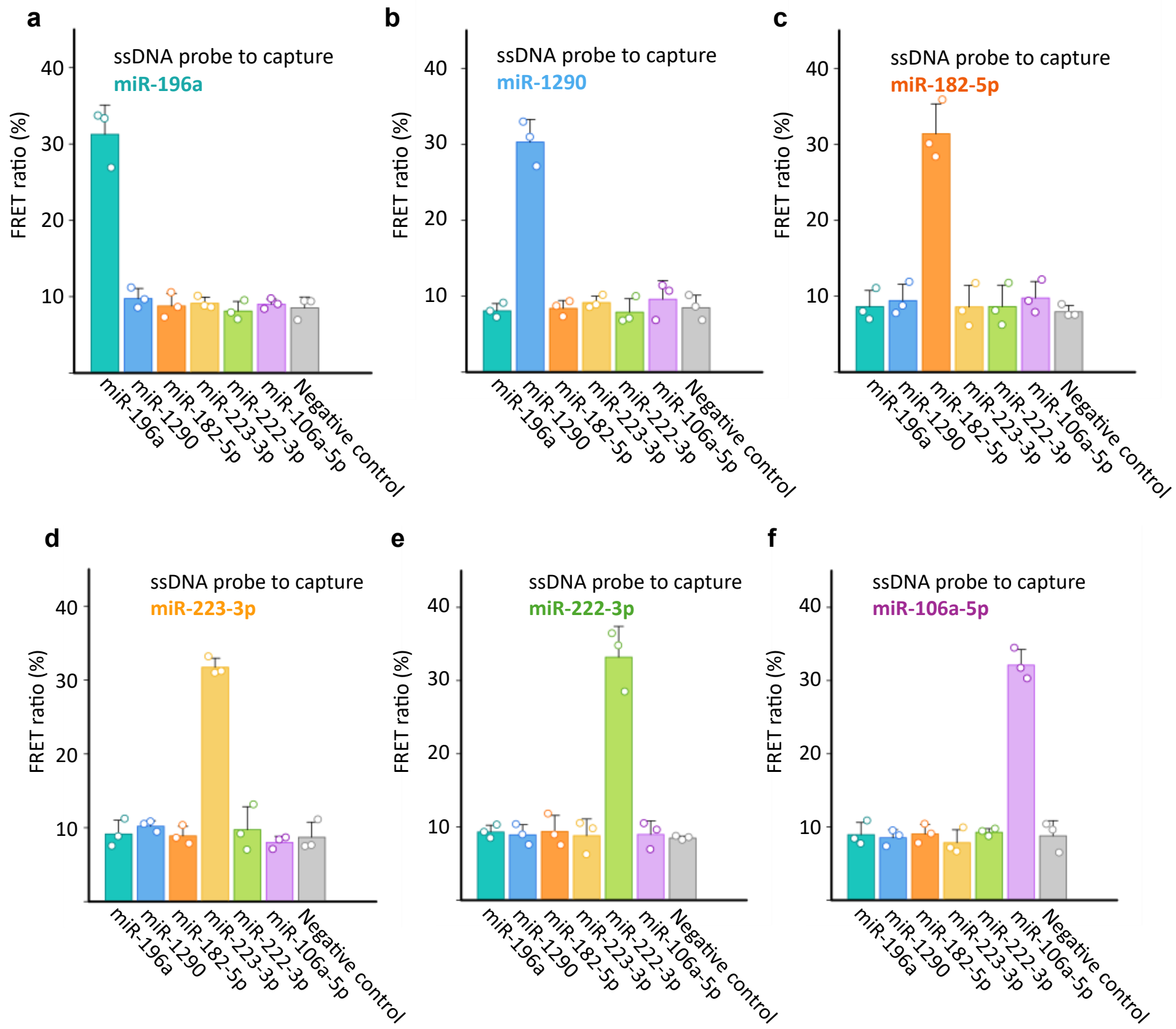

The highly linear correlation ($R^2 \geq 0.97$) between readout signals (FRET ratios) and miRNA concentrations was also confirmed in the detection of all six miRNA markers above (Supplementary Figs. 19 and 22 of the revised manuscript).

As mentioned earlier in the response to Comment 1, we estimated AUC, cut-off values,

and clinical sensitivity and specificity for each NSCLC-associated miRNA marker (miR-196a, miR-1290, or miR-182-5p) from the analysis of ROC curves in the diagnosis of 80 NSCLC patients and 80 healthy donors. In the manuscript revision, we additionally performed the diagnosis of GC using 40 patient- and 40 healthy sera through detecting three GC-associated miRNA markers (miR-223-3p, miR-222-3p, and miR-106a-5p) and estimated AUC, cut-off values, and clinical sensitivity and specificity from the ROC curve for each marker (see the figure below, Supplementary Fig. 25b) as follows (AUC, sensitivity, specificity): 0.9494, 95.0%, and 92.5% for miR-223-3p; 0.9188, 95.0%, and 87.5% for miR-222-3p; and 0.8838, 95.0%, and 85.0% for miR-106a-5p, which is likely comparable or better performance as compared to cancer diagnosis of recent literatures that were introduced in the response above to Comment 1.

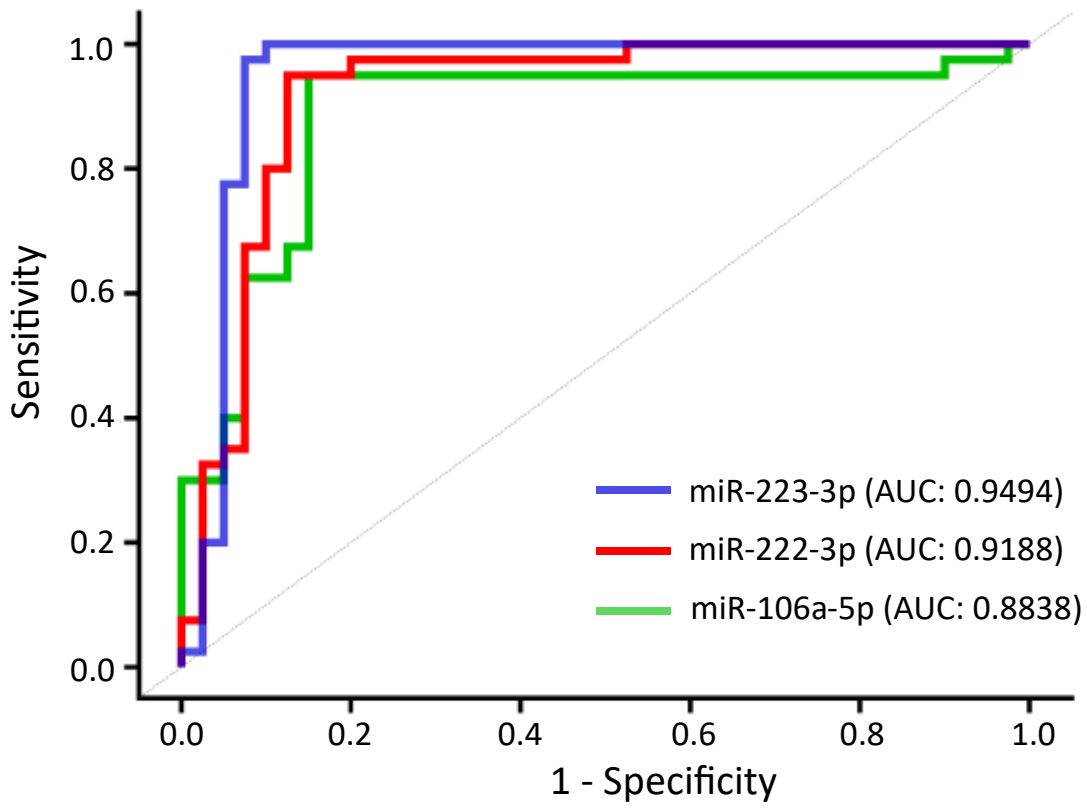

Spearman's rank correlation coefficients (ρ) between the diagnostic results by FRET platform and RT-qPCR were also estimated to be 0.9131, 0.8989, and 0.8678 ($p < 0.0001$) for miR-223-3p, miR-222-3p, and miR-106a-5p, respectively (Supplementary Figs. 26 of the revised manuscript), and the ρ values are high enough to remark that the readout signal patterns of FRET platform and RT-qPCR are in good agreement (*Nat. Commun.* **16**, 2610 (2025); *Anesth. Analg.* **126**, 1763–1768 (2018)).

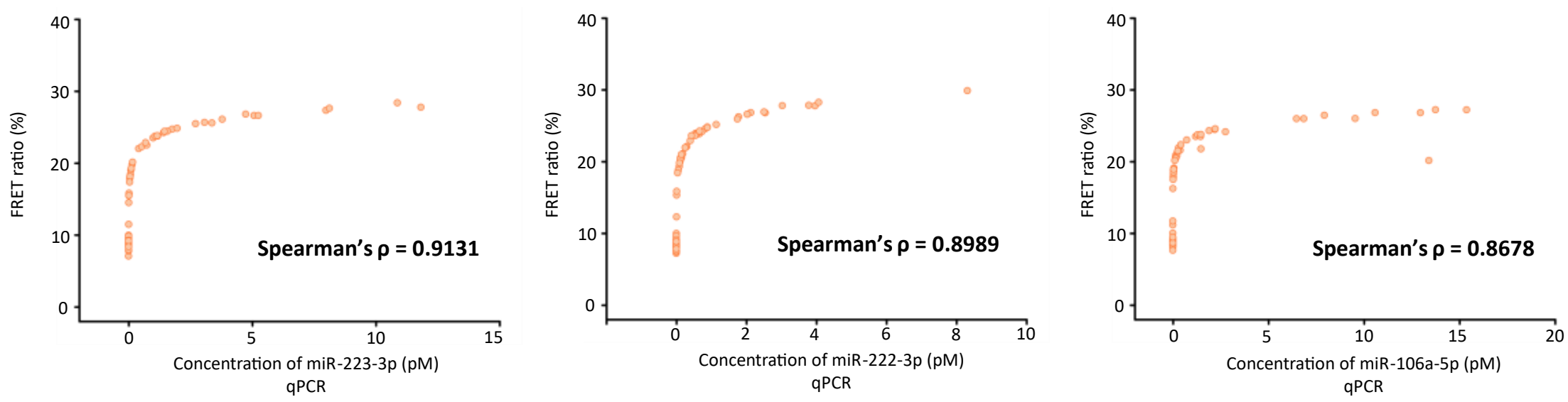

The above results collectively demonstrate that FRET platform is not confined to detecting a particular miRNA marker and is capable of detecting cancer-associated, various miRNA markers with different sequences and lengths with high selectivity, which was discussed in the text of revised manuscript (p. 12–15).

Reviewer #2:

The manuscript describes the development of a novel RNase H1 hybrid binding domain (HBD)-based FRET platform utilizing DNA-spiked protein nanocages for the direct, amplification-free detection of microRNAs in serum. The authors detail the genetic engineering of viral capsids to display capture probes and the use of the HBD to specifically recognize DNA-RNA hybrids, allowing for the discrimination of single-nucleotide mismatches. The study applies this platform to the detection of miR-196a in clinical serum samples for the diagnosis of non-small cell lung cancer (NSCLC), reporting high sensitivity and specificity. This work presents an innovative integration of protein nanotechnology and molecular recognition that could offer significant advantages over traditional PCR-based methods in terms of workflow simplicity. However, to fully support the claims of clinical utility and mechanistic robustness, several critical issues regarding sample size, experimental controls, and assay characterization need to be addressed.

Major comments:

[Comment 1]
The clinical validation currently includes only 40 patients with a distribution of 10 cases per clinical stage. For a study claiming significant "clinical diagnostic potential" and 100% sensitivity/specificity, this sample size is insufficient to draw statistically robust conclusions, particularly regarding the ability to accurately diagnose Stage I cancer against biological variance. The authors should either increase the sample size and provide a statistical power analysis.

Revision:
According to the reviewer's comment, we increased the cohort size from 40 NSCLC patients and 38 healthy donors to 80 NSCLC patients (25, 25, 15, and 15 patients at stage I, II, III, and IV, respectively) and 80 healthy donors (Supplementary Tables 1, 2 of the revised manuscript). In particular, the number of stage I- and II patients increased from 10 to 25 to verify the diagnostic performance for patients at earlier clinical stage. Further, as mentioned above, we increased NSCLC-associated miRNA marker to three (miR-196a, miR-1290, and miR-182-5p) and performed NSCLC diagnosis using FRET platform. ROC curves were plotted using the readout signals (FRET ratios) for 80 NSCLC patients and 80 healthy donors, followed by calculating AUC and cutoff values (Fig. 5h of the

revised manuscript, attached below), based on which we estimated clinical sensitivity and specificity as summarized as follows: 1) miR-196a: sensitivity 95.0% and specificity 95.0% (AUC 0.9836, cutoff value 13.05%); 2) miR-1290: sensitivity 90.0% and specificity 93.8% (AUC 0.9367, cutoff value 12.35%); and 3) miR-182-5p: sensitivity 98.8% and specificity 91.3% (AUC 0.9447, cutoff value 11.06%). This is significantly better, improved diagnostic performance, as compared to the other cancer diagnosis reported in recent literatures (sensitivity and specificity): 86.7, 93.3 % for breast cancer using CRISPR-Cas13a Gemini System (*Nat. Commun*. **15**, 2901 (2024)); 81.3, 90.7 % for lung cancer using RT-qPCR (*Proc Natl Acad Sci USA* **117**, 25036 (2020)); 89.0, 91.0 % (PDAC/pancreatic ductal adenocarcinoma using RT-qPCR (*Gastroenterology* **163**, 1252 (2022))].

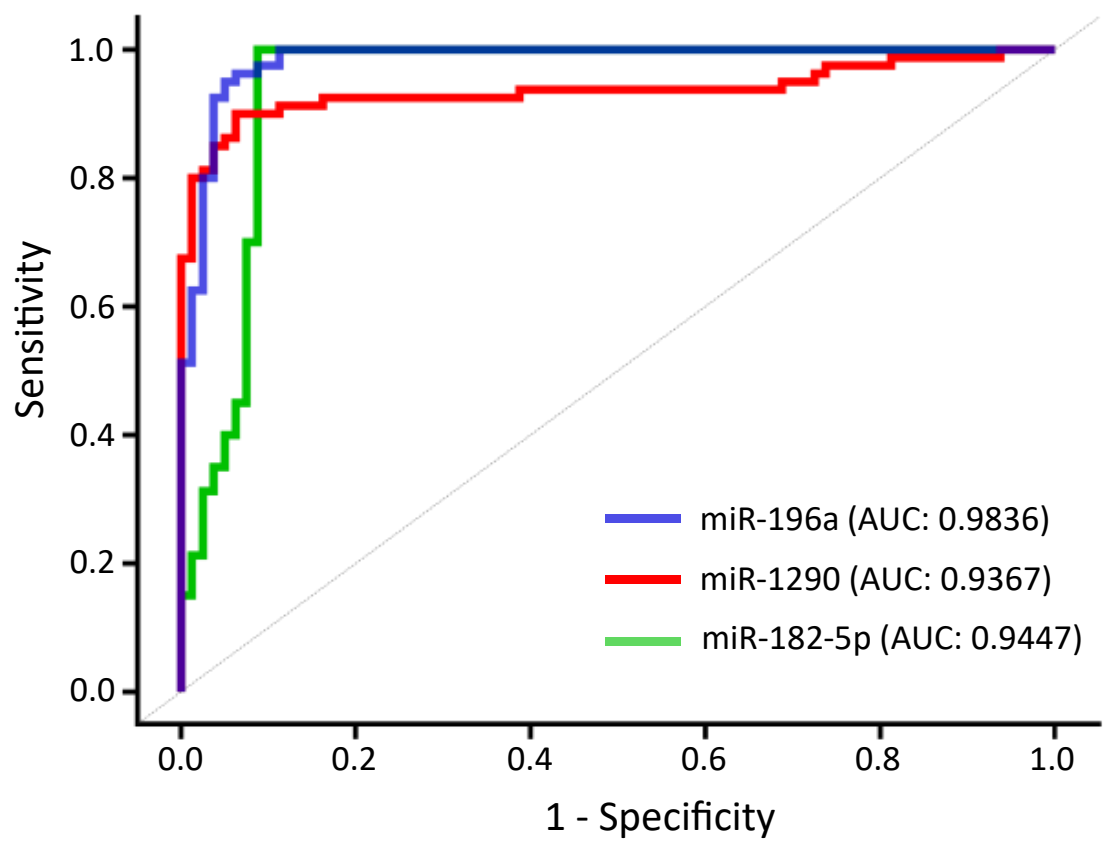

Notably, as shown in Figs. 5b, d, and f of the revised manuscript, the detection signals from stage I- and II patients are clearly distinguished from those of healthy group for all three miRNA markers, suggesting that FRET platform holds a high potential for early diagnosis of NSCLC.

The above results were discussed in the Results and Conclusion of the revised manuscript (p. 12-14) and (p. 16).

[Comment 2]

The method relies on the direct incubation of probes with patient serum. Serum is a complex matrix containing active nucleases (RNases) and a high background of non-specific nucleic acids and proteins. The manuscript lacks sufficient data demonstrating how the assay prevents the degradation of the target miRNA or the DNA probe by serum RNases during the incubation, and how non-specific binding events are excluded to ensure the signal is derived solely from the specific target.

Revision:

Although it seems obvious that in clinical detection of miRNA in patient serum, miRNA markers that survived against serum RNase are detected, we performed additional analyses according to the reviewer's comment.

We examined whether serum RNase activity substantially affects the results of FRET platform-based diagnosis, which is performed during 1-h incubation period (30 min for miRNA capture by FRET donor + 30 min for hybrid (ssDNA-miRNA) recognition by FRET acceptor). Synthetic let-7a miRNA was spiked into healthy serum at 0, 1.0, 2.5, 5.0, 7.5, and 10.0 fM, followed by let-7a detection by FRET platform in the absence or presence of Protector RNase Inhibitor (0.5 U/μL serum; Roche) (*Nat. Commun.* **11**, 6075 (2020)). As shown in Supplementary Fig. 17 of the revised manuscript, RNase inhibition does not significantly change the assay performance, that is, LOD still remains at femtomolar level although slightly decreased to ~ 2.5 fM from ~ 5 fM, indicating that serum RNase does not significantly affect the performance of FRET platform, which is presumably because the serum sample (50 μL) is mixed, or diluted with 150-μL assay solution and further because once miRNA-ssDNA hybrids are formed and subsequently bound by HBD of FRET acceptor, it is recalcitrant to RNase degradation. The above results were discussed in the Results (p. 12), and the experimental details were added to the Methods (p. 21) of revised manuscript.

Next, we investigated whether the ssDNA probes of FRET donor non-specifically capture non-target miRNAs in serum and thus cause false positive signals. That is, we examined if the ssDNA probes used in the diagnosis of NSCLC and GC (Supplementary Table 3 of the revised manuscript) capture non-target miRNA markers through cross-reactivity using 6 different miRNA markers (miR-196a, miR-1290, and miR-182-5p for NSCLC; miR-223-3p, miR-222-3p, and miR-106a-5p for GC). As shown in the figure below (Supplementary Figure 18 of the revised manuscript), each ssDNA probe emits the highest readout signal (FRET ratio) for its complementary target miRNA, while the readout signals for the other non-target miRNAs and negative control (healthy serum only) are negligible, indicating that highly selective, target-specific detection of miRNA is enabled by FRET platform.

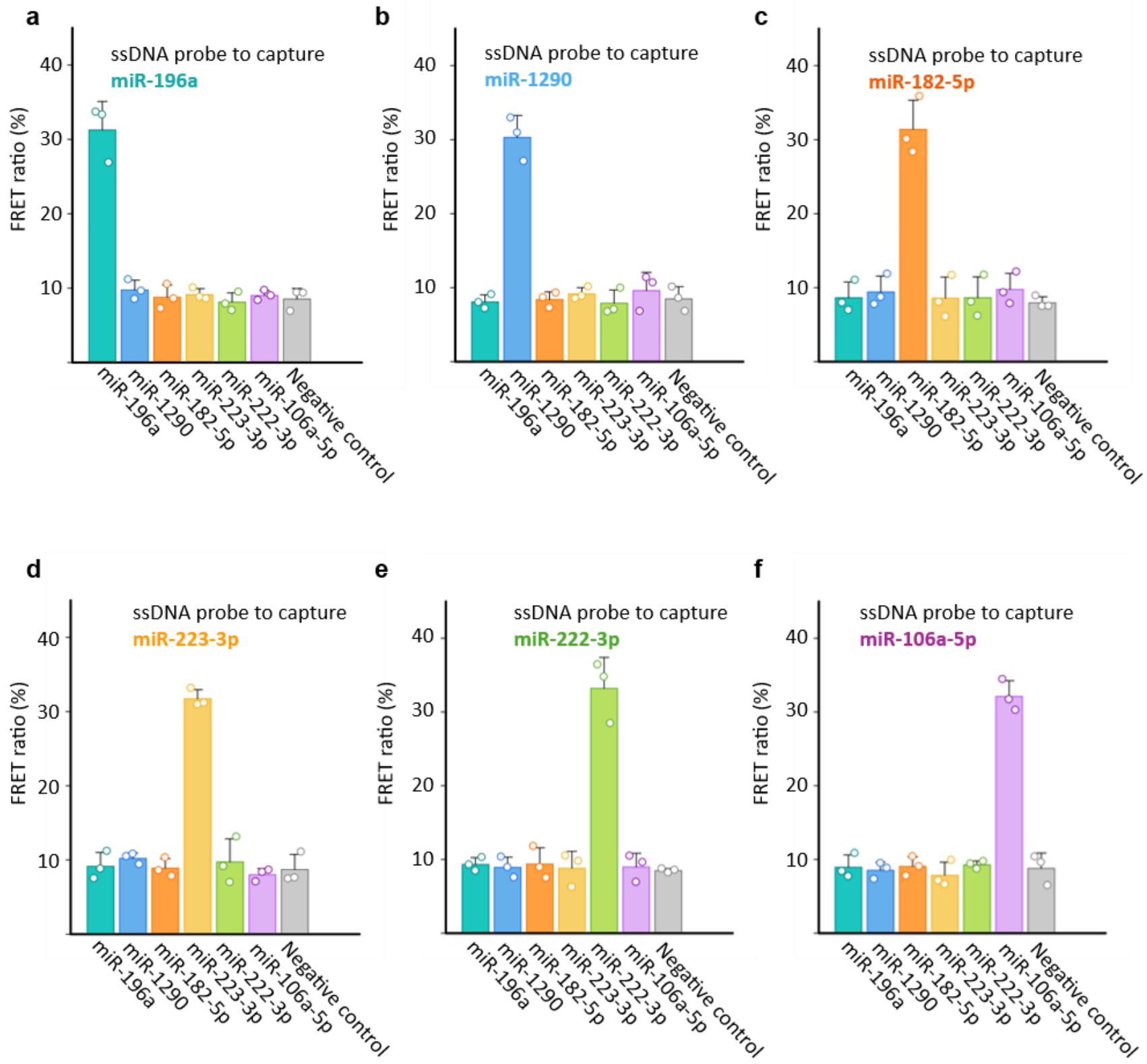

The above results were discussed in the Results of the revised manuscript (p. 12,13).

[Comment 3]

In Figure 3, the authors demonstrate discrimination of single and double base mismatches; however, the sensitivity of the HBD is known to be sensitive to the structural perturbation of the helix. The authors should investigate and discuss whether the position of the mismatch affects the HBD binding affinity and the resulting FRET signal, as this is critical for the general applicability of the assay.

<u>Revision</u>:

According to the comment of reviewer, we further examined whether the location of mismatch(es) within the hybrid helix (ssDNA-miRNA) affects the assay performance of FRET platform through additional experiments.

First, we mutated the sequence of ssDNA probe (5'-AAC TAT ACA ACC TAC TAC CTC A-3') that is complementary to let-7a miRNA (a cancer-associated miRNA marker, 3'-UUG AUA UGU UGG AUG AUG GAG U-5'), resulting in the preparation of 4 different ssDNA probes as follows: 5'-AAC CAT ACA ACC TAC TAC CTC A-3' (for a single mismatch proximal to 5' end), 5'-AAC TAT ACA ACC TAC TAC CCC A-3' (for a single

mismatch proximal to 3' end), 5'-AAC TAT ACA ACC CAC TAC CTC A-3' (for a single mismatch in middle region), and 5'-AAC CAT ACA ACC TAC TAC CCC A-3' (for double mismatch). Then, we spiked let-7a into human healthy serum at 10, 10^2, 10^3, and 10^4 fM and measured readout signals (FRET ratios) by FRET platform using each of let-7a-complementary- and 4 mutated ssDNA probes above. As shown in the figure below (Supplementary Figure 14 of the revised manuscript), the let-7a-complementary ssDNA probe emits distinctly the highest and concentration-dependent signals compared to the other mutated ssDNA probes, which lead to single or double base mismatch at various positions upon the capture of let-7a and emit notably lower, negligible signals, indicating that the discrimination of mismatched hybrids by HBD of FRET acceptor is not influenced by mismatch position. This result is explainable with the native mechanism of recognizing DNA-RNA hybrids by HBD, which is that HBD senses overall A-form minor-groove geometry of hybrid helix regardless of mismatch positions.

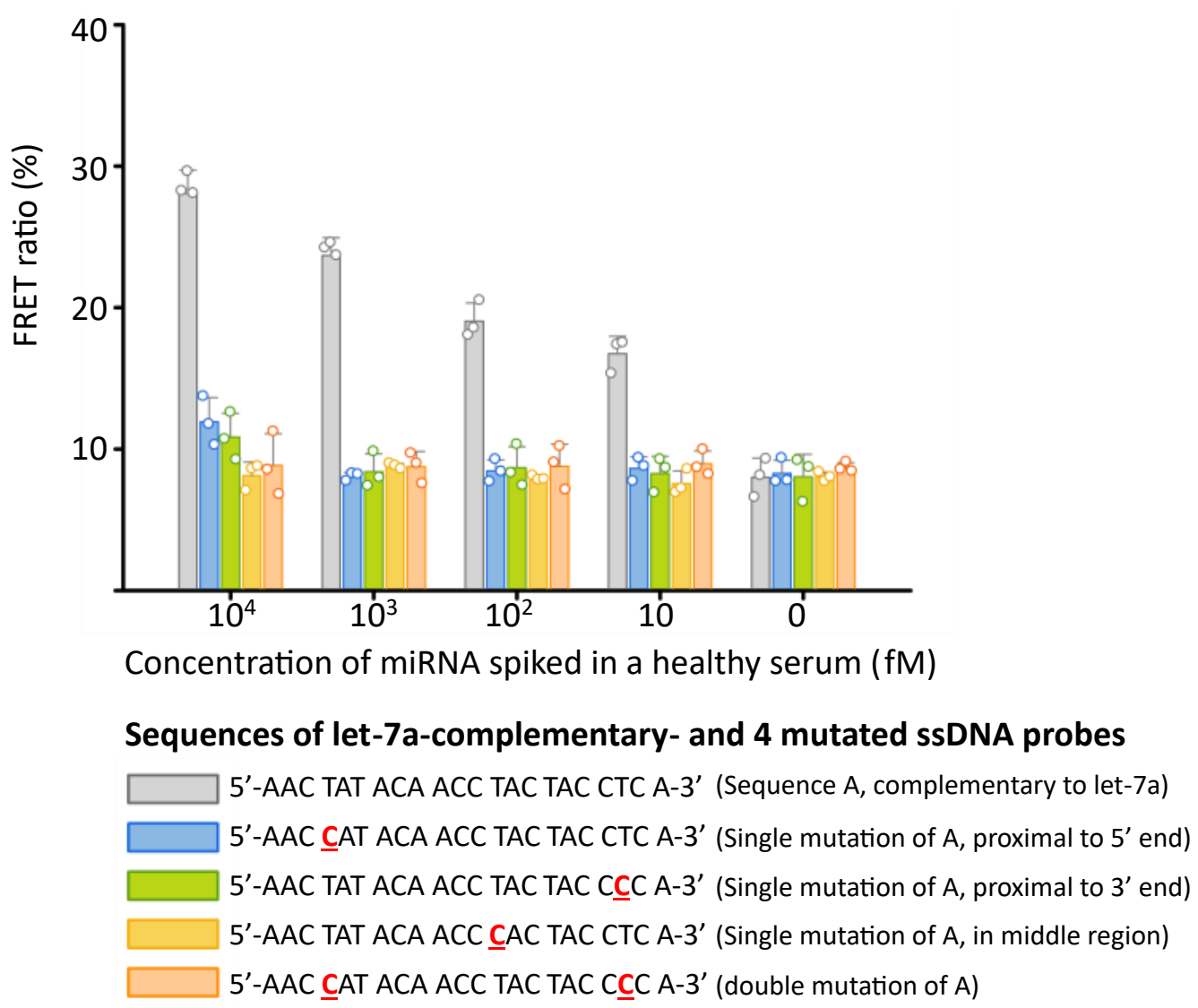

The above results were discussed in detail in the text of revised manuscript (p. 9,10).

[Comment 4]

The rationale for selecting miR-196a needs stronger justification; while upregulated in NSCLC, it is not a unique marker for this malignancy. To demonstrate the versatility and robustness of the platform, the authors should provide data showing the detection of other low-abundance miRNAs or targets relevant to other conditions, proving the platform is not limited to this specific sequence.

Revision:

To diagnose NSCLC, we tested two additional NSCLC-associated miRNA markers – miR-1290 and miR-182-5p – alongside miR-196a. Reportedly, serum miR-1290 is significantly overexpressed in NSCLC patients (*J. Thorac. Dis.* **7**, 1570–1579 (2015); *Onco Targets Ther*. **13**, 7809–7818 (2020)), and miR-182-5p is also upregulated in NSCLC tissues and sera, because it functions as an oncogene that promotes cancer cell proliferation, migration, and invasion (*PLoS ONE* **11**, e0153046 (2016); *BMC Med. Genomics* **13**, 3 (2020), *J. Cancer* **12**, 7120–7129 (2021)). The full sequences of the three NSCLC-associated miRNA markers and their complementary ssDNA probes are summarized in Supplementary Table 3 of the revised manuscript.

Target miRNA	Length (nt)	Sequence (5′ → 3')	ssDNA probe (5′ → 3')
let-7a	22	UGA GGU AGU AGG UUG UAU AGU U	AAC TAT ACA ACC TAC TAC CTC A-spacer (C6)-mal
let-7f	22	UGA GGU AGU AGA UUG UAU AGU U	-
let-7g	22	UGA GGU AGU AGU UUG UAC AGU U	-
miR-196a	22	UAG GUA GUU UCA UGU UGU UGG G	CCC AAC AAC ATG AAA CTA CCT A-spacer (C6)-mal
miR-1290	19	UGG AUU UUU GGA UCA GGG A	TCC CTG ATC CAA AAA TCC A-spacer (C6)-mal
miR-182-5p	24	UUU GGC AAU GGU AGA ACU CAC ACU	AGT GTG AGT TCT ACC ATT GCC AAA-spacer (C6)-mal
miR-223-3p	21	AGC UAC AUC UGG CUA CUG GGU	ACC CAG TAG CCA GAT GTA GCT-spacer (C6)-mal
miR-222-3p	22	UGU CAG UUU GUC AAA UAC CCC A	TGG GGT ATT TGA CAA ACT GAC A-spacer (C6)-mal
miR-106a-5p	23	AAA AGU GCU UAC AGU GCA GGU AG	CTA CCT GCA CTG TAA GCA CTT TT-spacer (C6)-mal

The correlation between FRET ratio (%) and miRNA concentration shows excellent linearity for every miRNA marker (Supplementary Fig. 19 of revised manuscript). Based on these linear correlations, limit of detection (LOD) was estimated to be ~ 5 fM for all markers (Supplementary Fig. 20 of the revised manuscript), which is below the range of circulating miRNA concentration in the blood of cancer patients (*Nat. Commun.* **15**, 2901 (2024), *Nat. Commun.* **15**, 8342 (2024), *Anal. Chem.* **94**, 14659 (2022)).

In addition, we increased the cohort size by adding 40 more patients (15, 15, 5, and 5 patients at stage I, II, III, and IV, respectively) and 42 more healthy controls, meaning that we tested totally 80 NSCLC patients (25, 25, 15, and 15 patients at stage I, II, III, and IV, respectively) and 80 healthy donors. The box plots of the miRNA levels in patient sera, measured by both RT-qPCR and FRET platform, are presented in the figures below (Figs. 5a-f of the revised manuscript). Notably, in the detection of all three miRNA markers (miR-196a, miR-1290, and miR-182-5p) in each serum of 80 NSCLC patients, FRET platform gave such readout signals (FRET ratios) that are clearly distinguished from those

for healthy group. Notably, compared to RT-qPCR, the FRET ratios for stage I- and II patients are more clearly higher than those for healthy group, suggesting that FRET platform is more effective for early diagnosis of NSCLC.

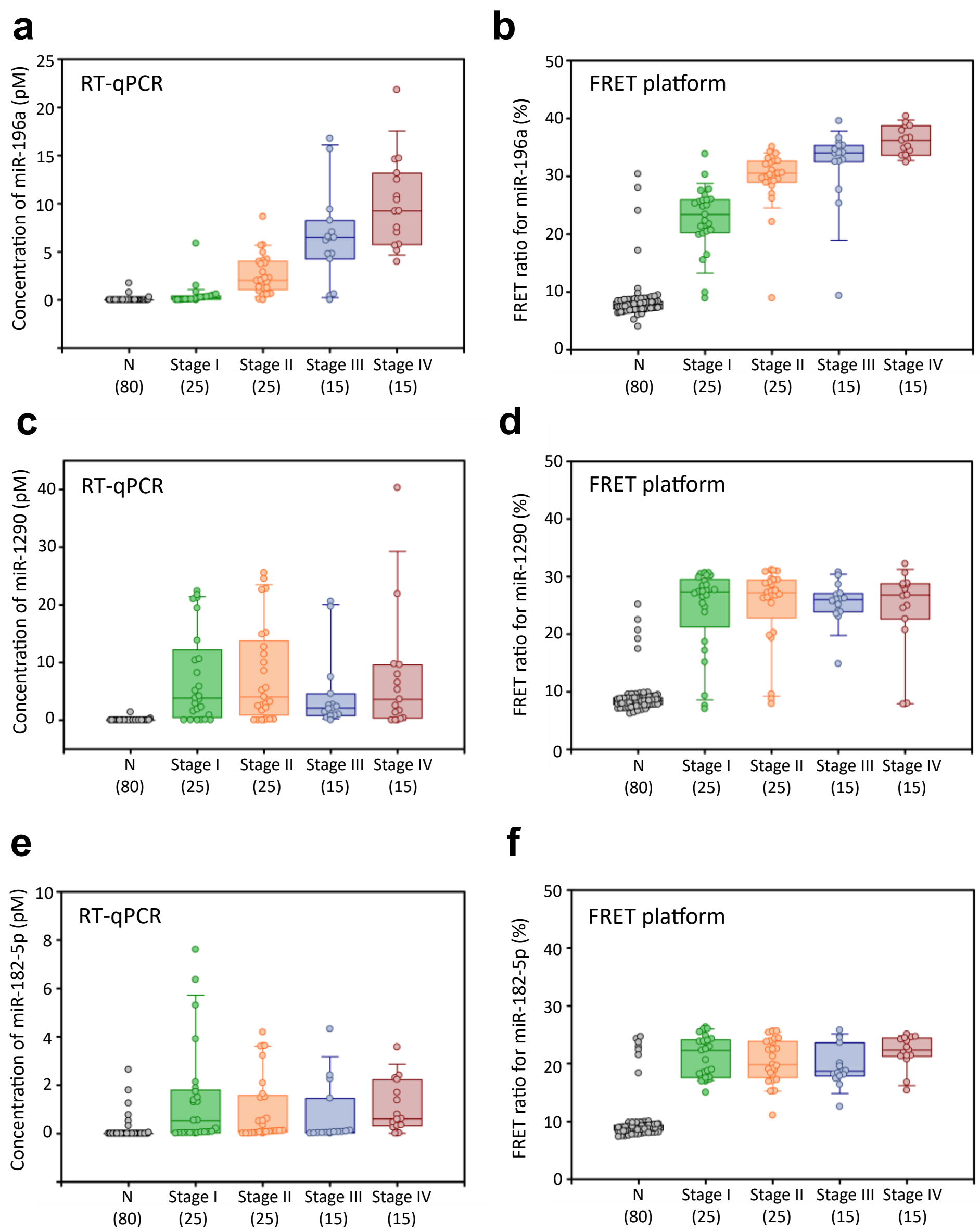

As seen in the heat maps below (Fig. 5g of the revised manuscript), which are prepared using the same readout signals as the box plots above, the readout signal pattern from FRET platform and qRT-PCR is very similar.

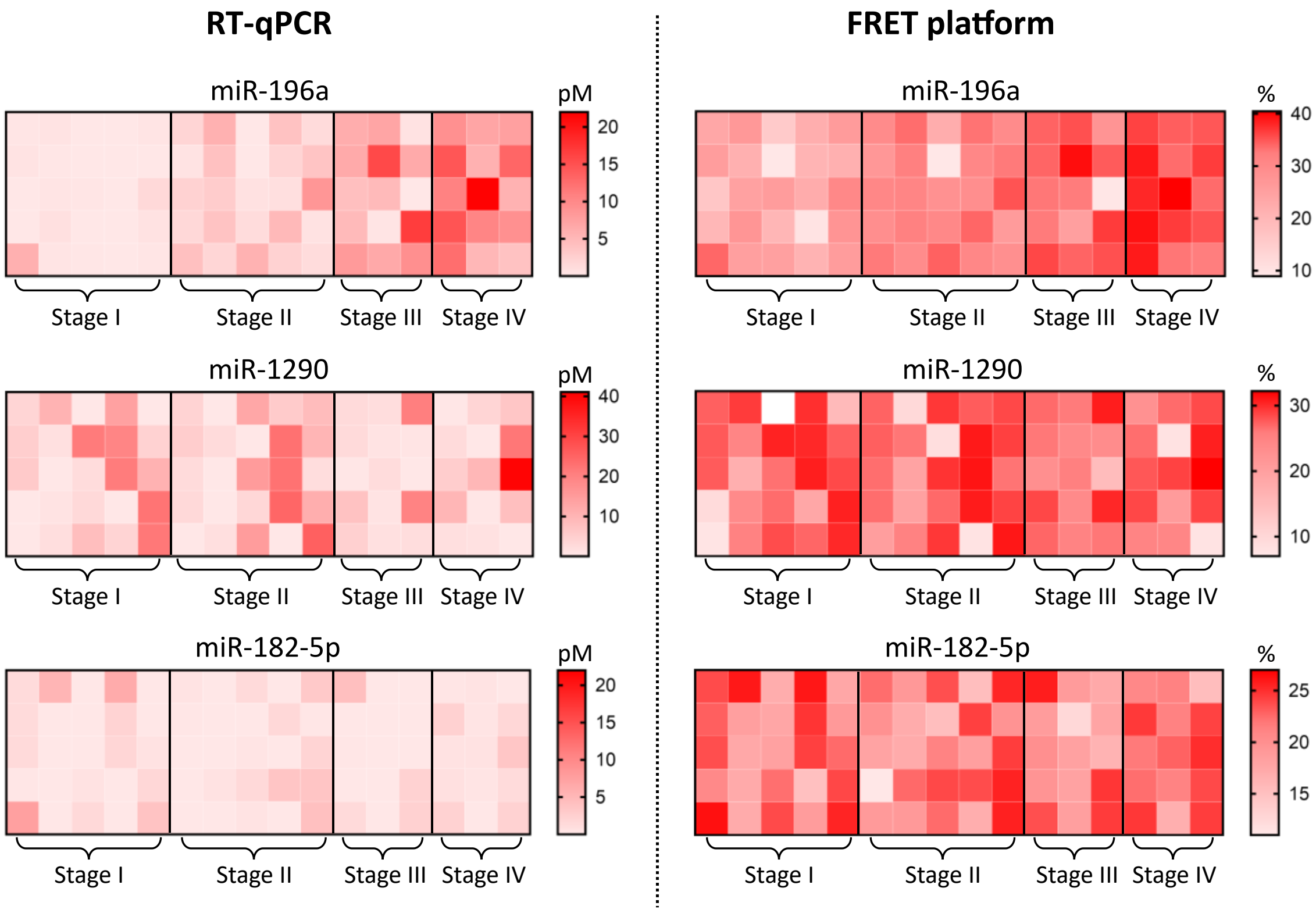

Further, to quantitatively evaluate the diagnostic performance (sensitivity and specificity) of FRET platform, ROC curves were plotted and analyzed for all miRNA markers as presented below (Fig. 5h of the revised manuscript).

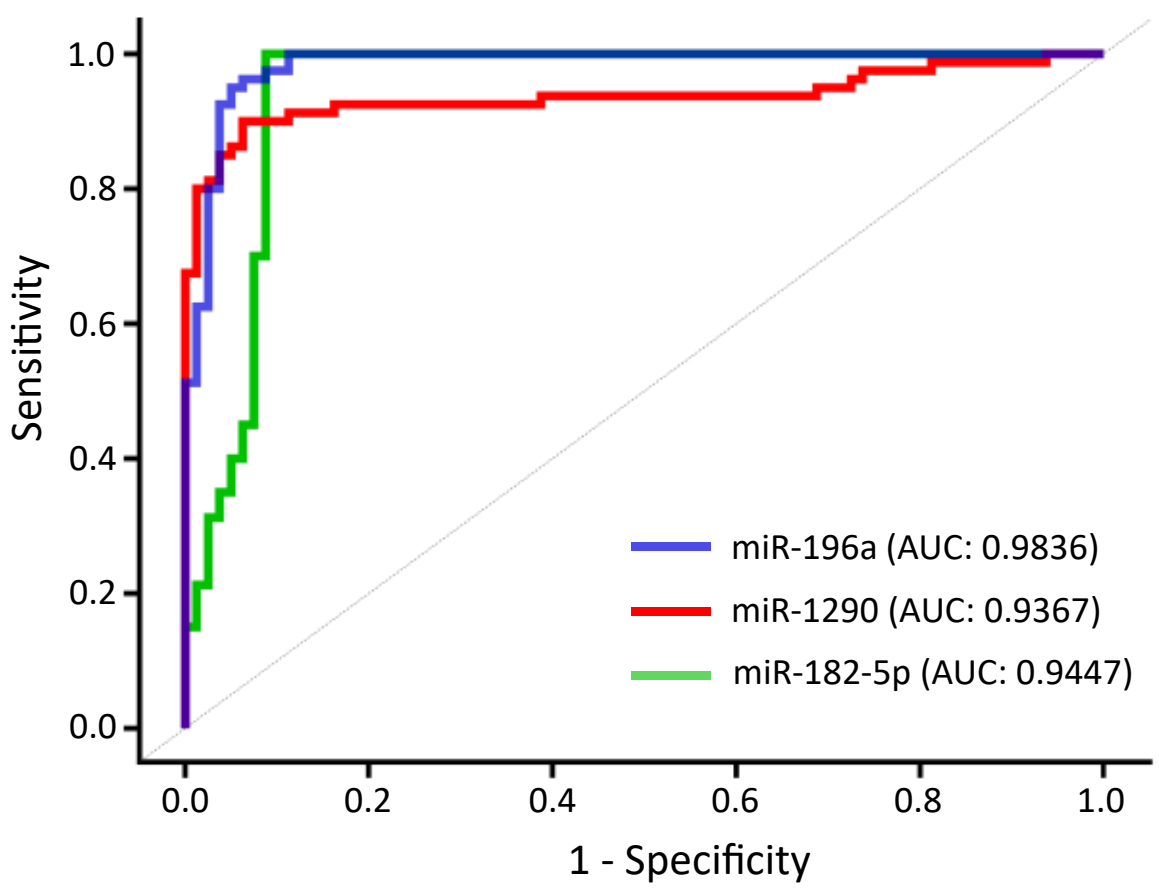

The AUC values were 0.9836 (95% confidence interval (CI): 0.9669 ~ 1.000), 0.9367 (95% CI: 0.8931 ~ 0.9804), and 0.9447 (95% CI: 0.9033 ~ 0.9860) for miR-196a, miR-1290, and miR-182-5p, respectively, all of which are high enough to claim that FRET platform is such a reliable diagnostic method that well distinguishes between patient- and healthy

groups. Also, using Youden index (J = sensitivity + specificity − 1), which is a widely used criterion for selecting a cutoff point that maximizes overall classification performance in ROC analysis (*Nat. Commun.* **15**, 10213 (2024); *BMC Med. Res. Methodol.* **24**, 84 (2024)), we estimated the optimal cutoff of FRET ratios to be 13.05%, 12.35%, and 11.06% for miR-196a, miR-1290, and miR-182-5p, respectively, through calculating J at every possible threshold along the ROC curve to find an optimal cutoff that yields maximum J. Based on the cutoff values above, clinical sensitivity and specificity in the diagnosis of 80 NSCLC patients and 80 healthy donors were estimated for each miRNA marker: 95.0 and 95.0 %, respectively, for miR-196a; 90.0 and 93.8%, respectively, for miR-1290; and 98.8 and 91.3 %, respectively, for miR-182-5p, demonstrating that FRET platform enables significantly better, improved diagnostic performance as compared to the other recent reports on cancer diagnosis [sensitivity and specificity: 86.7, 93.3 % for breast cancer using CRISPR-Cas13a Gemini System (*Nat. Commun*. **15**, 2901 (2024)); 81.3, 90.7 % for lung cancer using RT-qPCR (*Proc. Natl. Acad. Sci. U.S.A.* **117**, 25036 (2020)); 89.0, 91.0 % (PDAC/pancreatic ductal adenocarcinoma using RT-qPCR (*Gastroenterology* **163**, 1252 (2022))].

The above results were discussed in the Results (p. 12-14) and Conclusion (p. 16) of revised manuscript.

[Comment 5]

The current data relies on a single miRNA length and sequence model. To validate the methodological advantage and structural compatibility of the nanocage-HBD system, the authors should test miRNAs of varying lengths and sequences to ensure that the steric constraints of the nanocage do not hinder the detection of different targets.

Revision:

According to the reviewer's comment, we tested 6 miRNA markers with different sequences and lengths (19 to 24 nt) in the diagnosis of NSCLC and GC as summarized in Supplementary Table 3: three markers (miR-196a (22 nt), miR-1290 (19 nt), miR-182-5p (24 nt)) for NSCLC (80 patients) and another three markers (miR-223-3p (21 nt), miR-222-3p (22 nt), and miR-106a-5p (23 nt)) for GC (40 patients). The calibration fit and LOD were estimated for each miRNA marker as summarized in the table below (Supplementary Table 5 of the revised manuscript). Since this comment is the same as the Comments 1 and 5 of Reviewer #1, please see our responses to the comments, where the diagnostic performances of FRET platform using miRNA markers for each cancer are

described in detail.

Cancer type	Target miRNA	Length (nt)	Calibration fit	R^2	LOD
NSCLC	miR-196a	22	$y = 4.61\log x + 11.65$	0.969	~5 fM
NSCLC	miR-1290	19	$y = 6.45\log x + 10.96$	0.996	~5 fM
NSCLC	miR-182-5p	24	$y = 3.72\log x + 12.99$	0.988	~5 fM
GC	miR-223-3p	21	$y = 5.09\log x + 10.86$	0.985	~5 fM
GC	miR-222-3p	22	$y = 4.46\log x + 11.22$	0.974	~5 fM
GC	miR-106a-5p	23	$y = 3.38\log x + 14.04$	0.989	~5 fM

[Comment 6]

While the study focuses on NSCLC, the discussion would benefit from a broader evaluation of the method's generalizability to other diseases or sample types to substantiate the claimed universality of the platform.

Revision:

As stated in our response to Comment 5 of Reviewer #1, FRET platform was also applied to the diagnosis of another type of cancer, or GC through detecting three GC-associated miRNA markers (miR-223-3p, miR-222-3p, and miR-106a-5p) using 40 GC patients and 40 healthy donors. As seen in the figures below (Supplementary Figs. 24 (box plots) and 25 (heat maps and ROC curves) of the revised manuscript), FRET platform clearly distinguishes GC patients from healthy controls in the detection of each GC marker (box plots), and the comparison of heat maps between RT-qPCR and FRET platform also confirms that the two different diagnostic methods give a similar pattern of readout signals for all 40 GC patients.

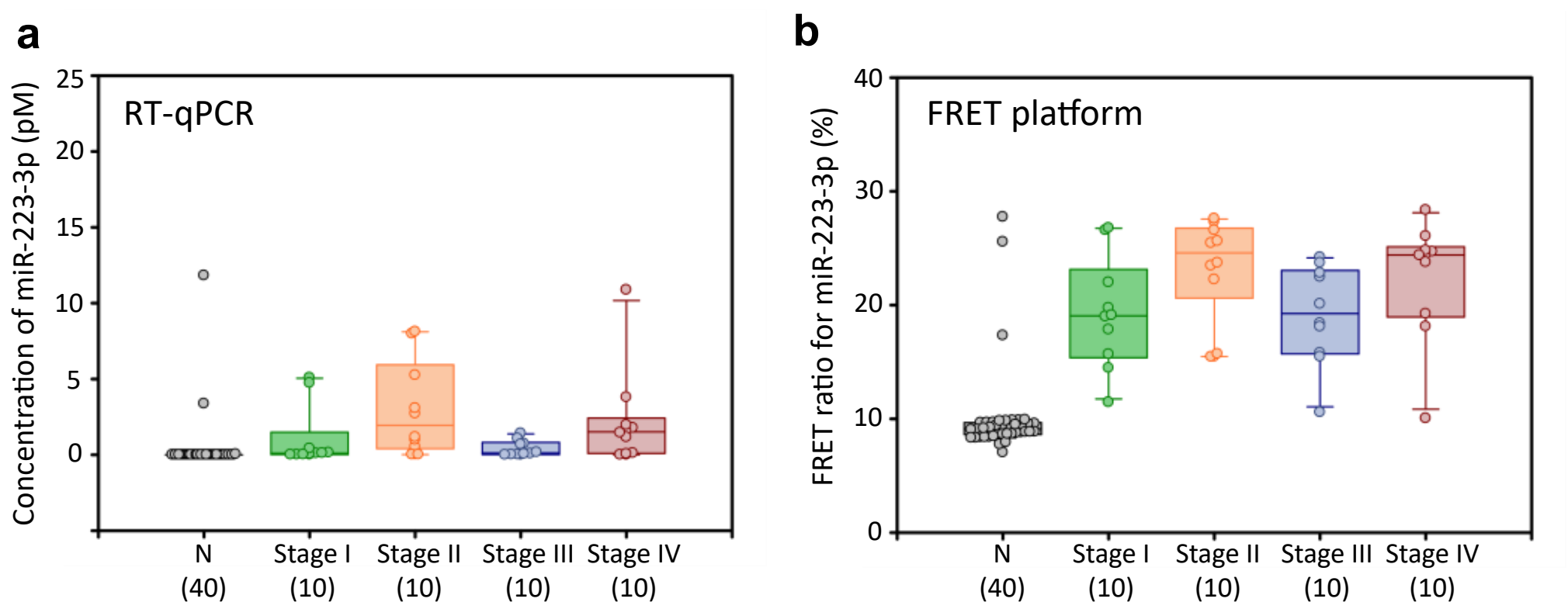

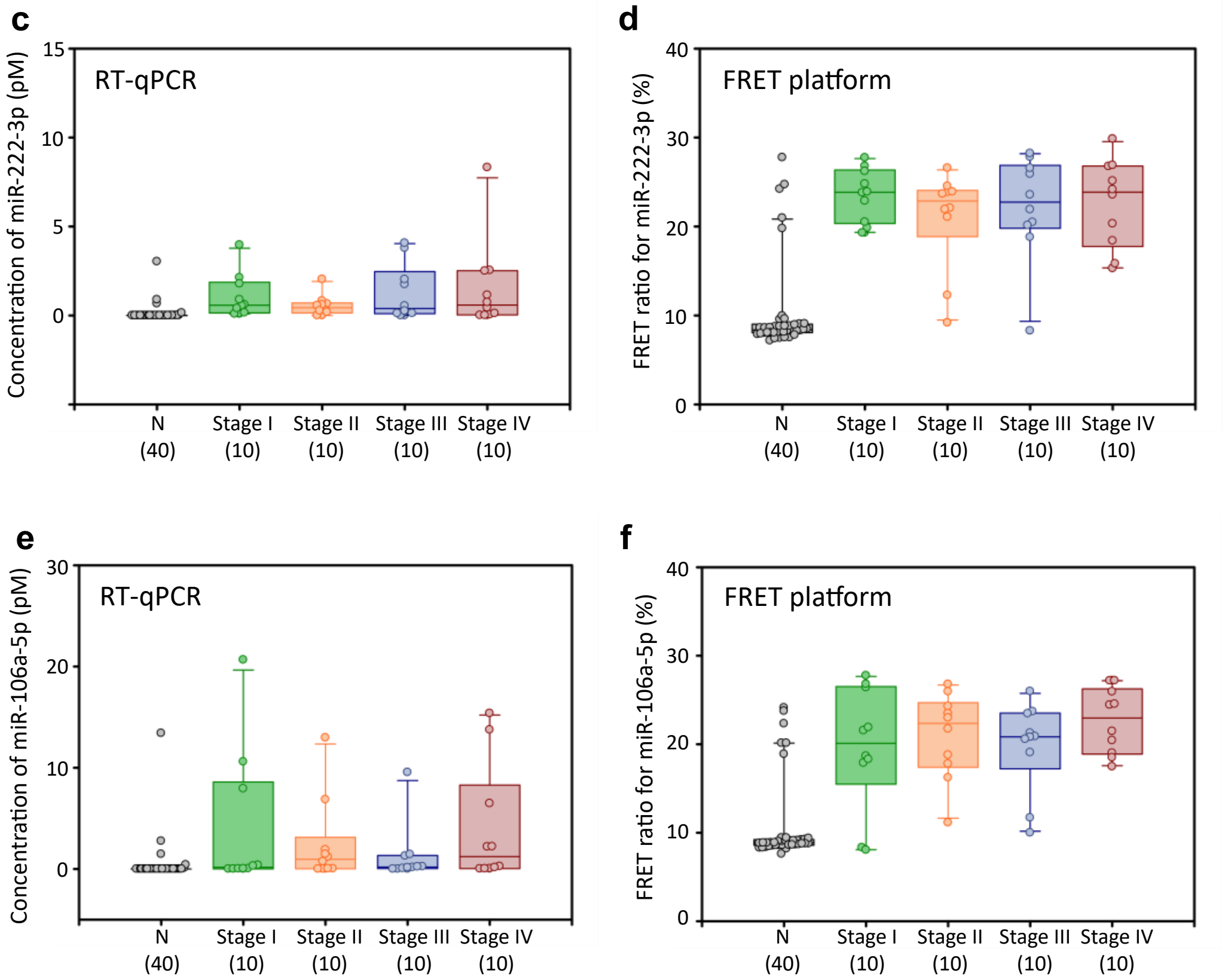
c
RT-qPCR
Concentration of miR-222-3p (pM)
N (40)
Stage I (10)
Stage II (10)
Stage III (10)
Stage IV (10)
d
FRET platform
FRET ratio for miR-222-3p (%)
e
RT-qPCR
Concentration of miR-106a-5p (pM)
f
FRET platform
FRET ratio for miR-106a-5p (%)

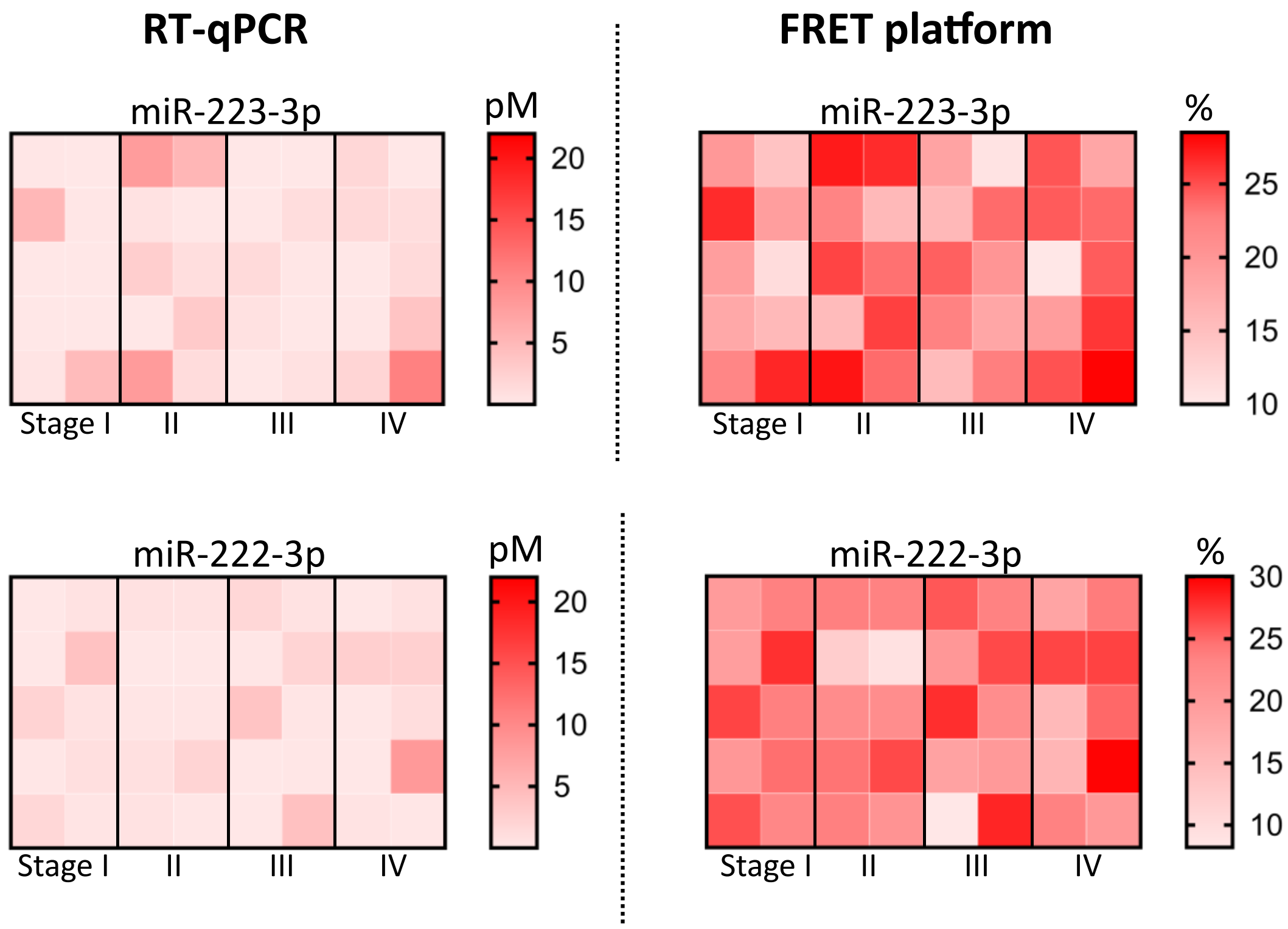
RT-qPCR
FRET platform
miR-223-3p
miR-222-3p
pM
%
Stage I
II
III
IV

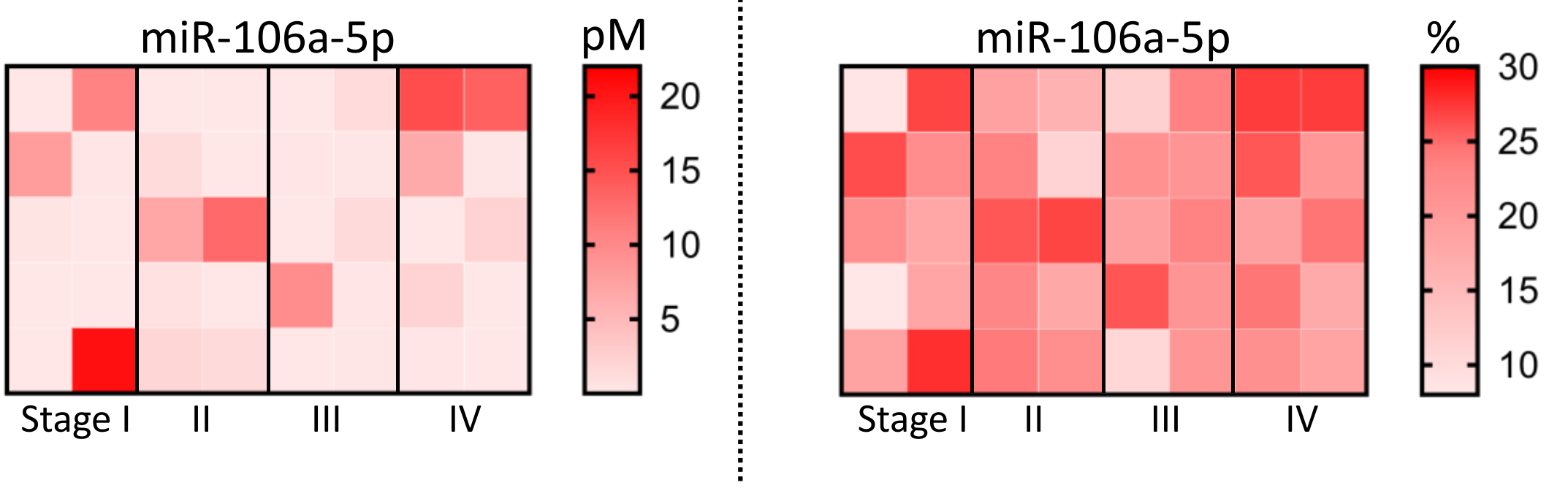

ROC curve analysis was also performed, giving the AUC values of 0.9494, 0.9188, and 0.8838 for each of GC markers detected by FRET platform (Supplementary Fig. 25b of the revised manuscript), which are sufficiently high values to claim that FRET platform is a reliable diagnostic method that well distinguishes between patient- and healthy groups. As described in the response to Comment 5 of Reviewer #1, we estimated the sensitivity and specificity in the GC diagnosis through ROC-based Youden index analysis as follows: (sensitivity, specificity) 95.0, 92.5% for miR-223-3p; 95.0, 87.5% for miR-222-3p; and 95.0, 85.0% for miR-106a-5p, which is likely comparable or better performance as compared to the cancer diagnosis of recent literatures [sensitivity and specificity: 86.7 and 93.3 % for breast cancer using CRISPR-Cas13a Gemini System (*Nat. Commun*. **15**, 2901 (2024)); 81.3 and 90.7 % for lung cancer using RT-qPCR (*Proc Natl Acad Sci USA* **117**, 25036 (2020)); 89.0 and 91.0 % (PDAC/pancreatic ductal adenocarcinoma using RT-qPCR (*Gastroenterology* **163**, 1252 (2022))].

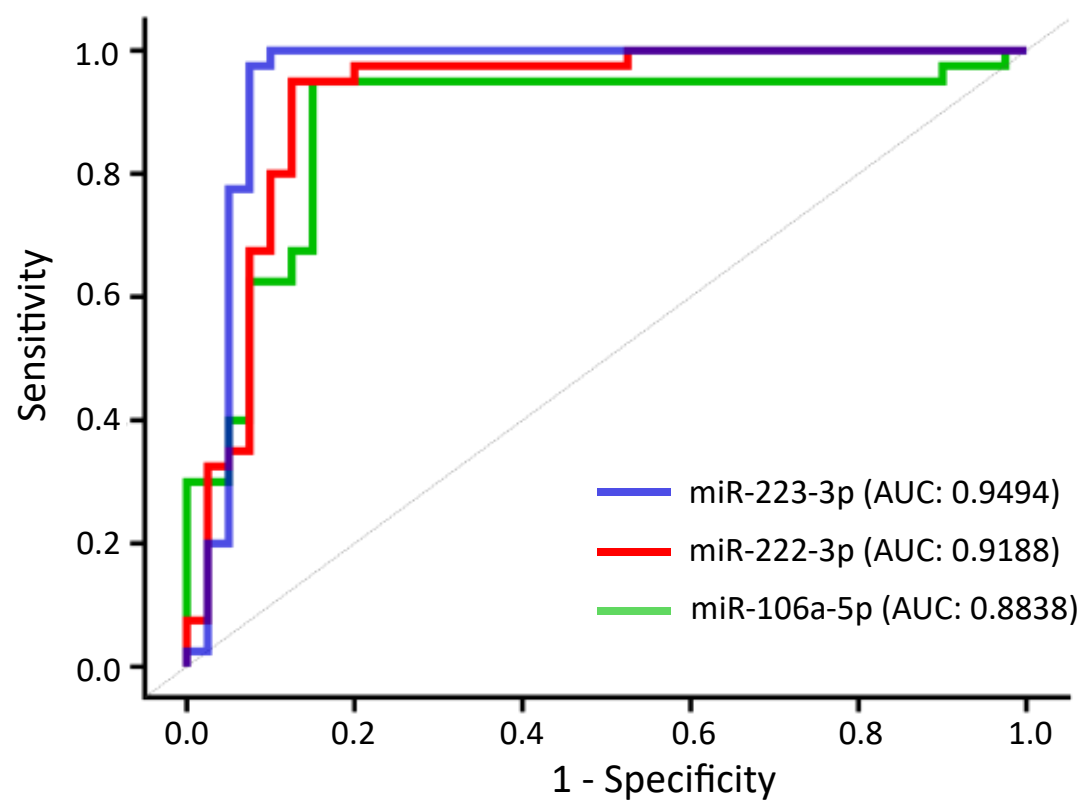

Together with the results of NSCLC diagnosis (Fig. 5), these results of GC diagnosis demonstrate that FRET platform holds a high potential for clinical diagnosis of various types of cancer, which is readily implemented by simply switching the ssDNA probes while keeping the other components unchanged.

We discussed the above results in the text of revised manuscript (p. 12-15).

[Comment 7]

There is a visual discrepancy in Figures 5a and 5b between the described sample size (10 patients per group) and the plotted data, which appears to show a density of points exceeding the stated N number. The authors must clarify this inconsistency, ensure the plots accurately reflect the individual data points, or explain the data visualization method used.

Revision:

In the original manuscript, each serum sample from patients and healthy donors was analyzed in triplicate, and all three data points per sample were marked in the box plots, which is the reason why the marked data points exceed the number of samples tested. In the revised manuscript, only an average value per sample was marked in the box plots (Figs. 5a-f).

Minor comments:

[Comment 1]

The title "RNase-dependent microRNA detection" is misleading because the assay utilizes the Hybrid Binding Domain (HBD) of RNase H1 for structural recognition, not the enzymatic cleavage activity of the RNase. The title and corresponding text should be revised to accurately reflect that this is an HBD-binding-based assay to avoid confusion regarding the mechanism.

Revision:

According to the reviewer's comment, we changed the paper title to "An RNase domain-dependent microRNA detection with DNA-spiked nanocage for accurate cancer diagnosis".

Reviewer #3:

Summary

This manuscript reports an RNaseH1-dependent, amplification-free FRET platform for detecting circulating microRNAs directly in serum. The system combines a DNA probe–decorated fluorescent HBV capsid nanocage (donor) with an RNaseH1 HBD–mOrange fusion protein (acceptor), with the goal of enabling highly specific, mismatch-sensitive miRNA detection. The authors report a limit of detection of ~5 fM and claim 100%

sensitivity and 100% specificity in distinguishing 40 NSCLC patients from 38 healthy controls based on serum miR-196a levels. The conceptual framework is interesting and technically sophisticated. However, the clinical and diagnostic claims are overstated, and key validation steps are missing. In its current form, the manuscript does not provide sufficient evidence to support the strength of its conclusions.

Major comments:

[Comment 1]

Diagnostic Performance Is Overstated

The claim of 100% sensitivity and 100% specificity is not adequately supported. The cohort size is modest (40 patients and 38 controls), yet no ROC curves, AUC values, confidence intervals, or validation procedures are provided. Without proper diagnostic statistics and independent validation, the reported performance cannot be considered robust. As presented, the data suggest separation between groups, but this is not equivalent to establishing clinical diagnostic accuracy.

Revision:

According the reviewer's comment, we increased the cohort size to 80 NSCLC patients (25 stage I, 25 stage II, 15 stage III, and 15 stage IV) and 80 healthy donors and attempted to detect three NSCLC-associated miRNA markers (miR-196a, miR-1290, and miR-182-5p), as listed in Supplementary Table 3 of the revised manuscript. To estimate diagnostic performance, ROC curves were plotted and analyzed for all miRNA markers. The AUC values were 0.9836 (95% confidence interval (CI): 0.9669-1.000) for miR-196a, 0.9367 (95% CI: 0.8931 to 0.9804) for miR-1290, and 0.9447 (95% CI: 0.9033 to 0.9860) for miR-182-5p as presented below (Fig. 5h of the revised manuscript). Then, the cutoff values were also calculated through ROC-based Youden index analysis, which are 13.05%, 12.35%, and 11.06% for miR-196a, miR-1290, and miR-182-5p, respectively, based on which sensitivity and specificity in the diagnosis of 80 NSCLC patients and 80 healthy donors were estimated for each marker as follows: (sensitivity, specificity) 95.0, 95.0 %, for miR-196a; 90.0, 93.8% for miR-1290; and 98.8 and 91.3 % for miR-182-5p, demonstrating that FRET platform enables significantly better, improved diagnostic performance as compared to the other recent reports on cancer diagnosis [sensitivity and specificity: 86.7, 93.3 % for breast cancer using CRISPR-Cas13a Gemini System (*Nat. Commun*. **15**, 2901 (2024)); 81.3, 90.7 % for lung cancer using RT-qPCR (*Proc. Natl. Acad. Sci. U.S.A.* **117**, 25036 (2020)); 89.0, 91.0 % (PDAC/pancreatic ductal

adenocarcinoma using RT-qPCR (*Gastroenterology* **163**, 1252 (2022))].

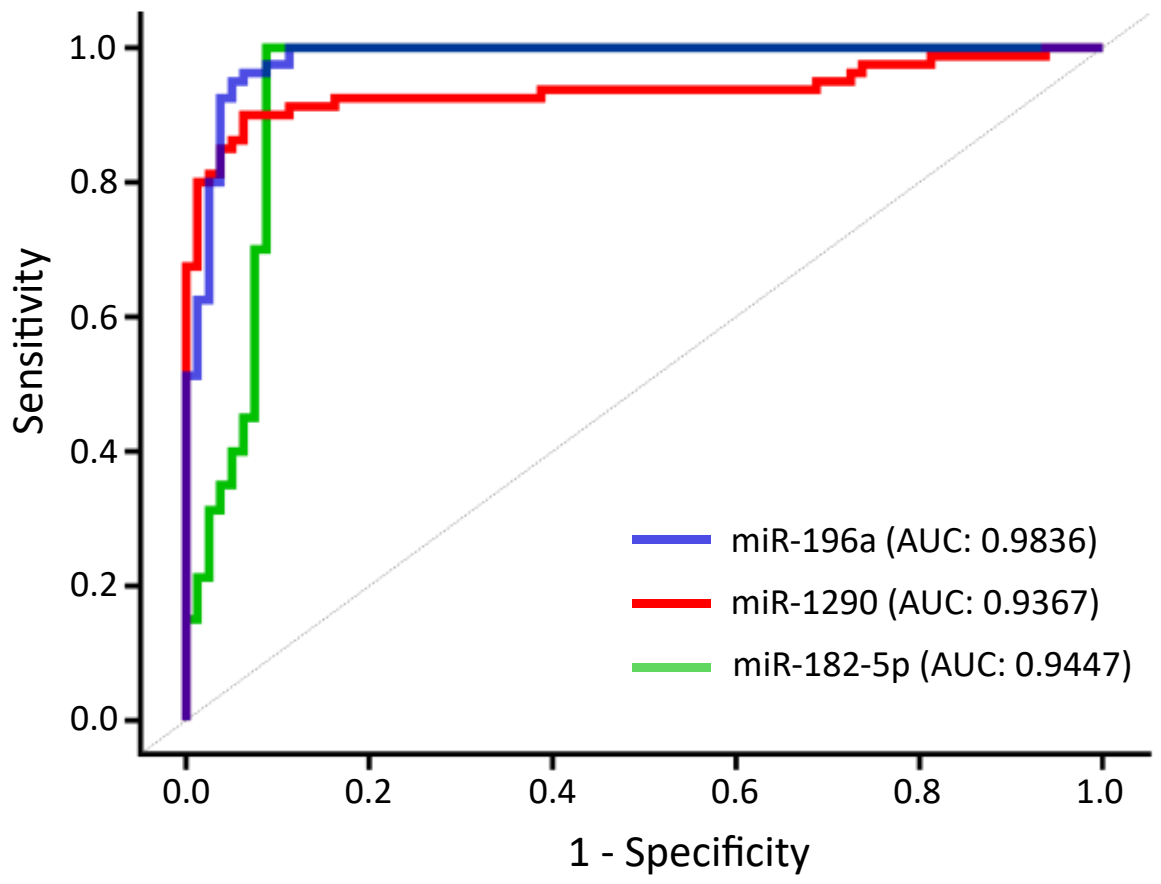

The details of diagnosis results (calibration fits, LOD, box plots, heat maps, and Spearman's rank correlation coefficients) are presented in our response to Comment 1 of Reviewer #1. Please see the response. These results were discussed in detail in the text of revised manuscript (p. 12-14, 16).

[Comment 2]

Comparison to RT-qPCR Is Not Convincing

The manuscript suggests that this platform outperforms RT-qPCR, particularly in early-stage disease. However, the RT-qPCR methodology is insufficiently detailed. There is no clear description of normalization strategy, technical replicates, recovery controls, or assay variability. Without a rigorous and well-controlled comparison, the claim of improved sensitivity over RT-qPCR is not persuasive.

<u>Revision</u>:

To address the reviewer's concern, we describe the details of RT-qPCR methodology, which follows the protocol from a commercial kit, or Mir-X miRNA First-Strand Synthesis and TB Green® qRT-PCR kit (Cat. No. 638314; Takara Bio, U.S.A.). All RT-qPCR measurements were performed in triplicate, as clearly stated in the revised Methods section. The normalization of the measured RT-qPCR data was done by standard curve-based quantification using synthetic miRNA marker standards, which were reverse-transcribed and amplified in parallel with the serum samples. As seen in the figures below (Supplementary Fig. 28 of the revised manuscript), standard curves between cycle threshold (Ct) and miRNA concentration show excellent linearity ($R^2 = 0.99$ for all; slopes ranging from −3.29 to −3.58) for each of all miRNA markers (miR-196a, miR-1290, and miR-182-5p for NSCLC; miR-223-3p, miR-222-3p, and miR-106a-5p for gastric cancer (GC)) used in this study, demonstrating analytical validity of RT-qPCR used.

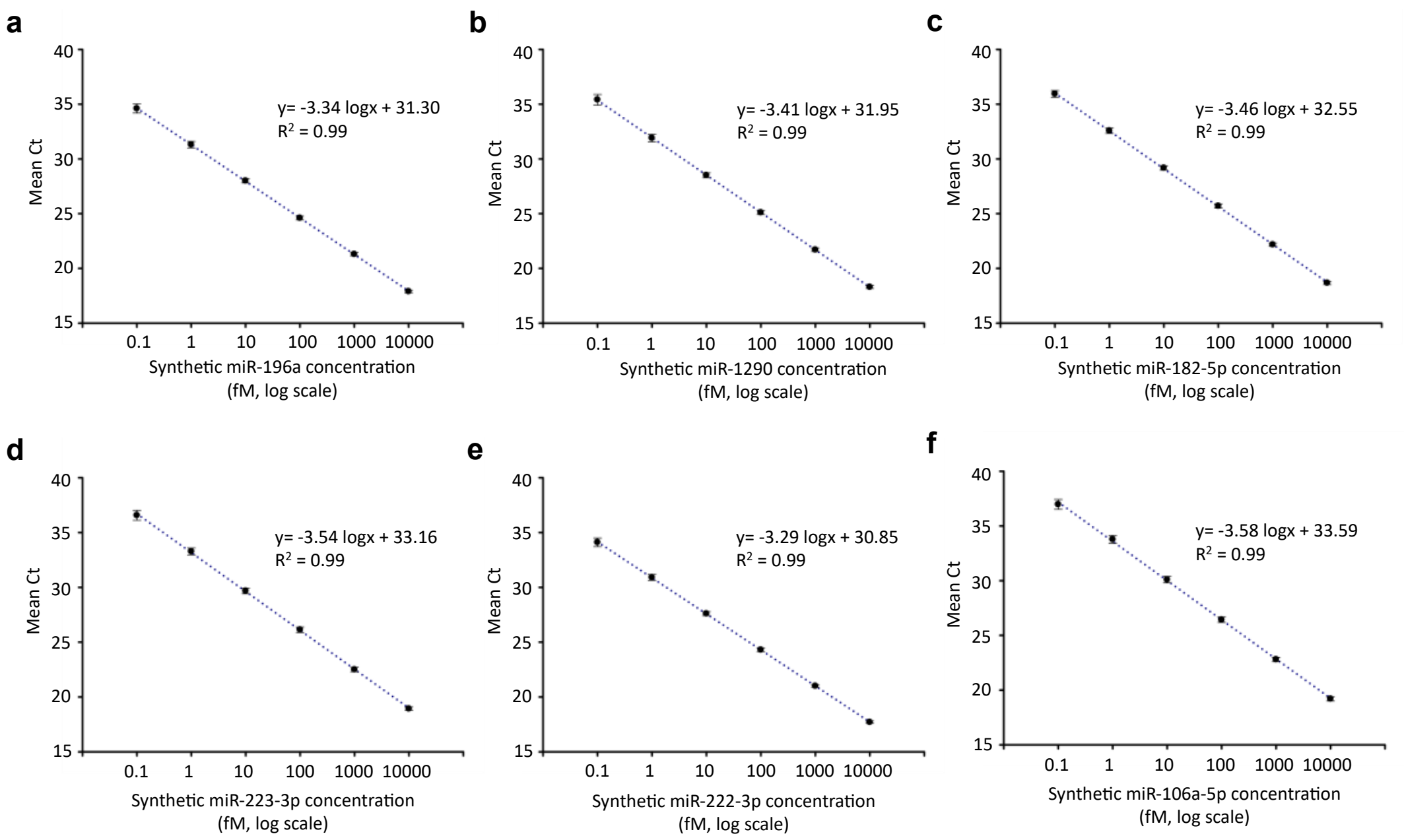

Matrix recovery and assay variability were evaluated through a matrix-matched spike-in experiment by spiking synthetic miR-196a to human healthy serum at 0 (background), 1.0, 2.5, 5.0, 7.5, and 10.0 fM, followed by processing these samples through RNA isolation, reverse-transcription, and qPCR, which is the same workflow as used for the clinical diagnosis of NSCLC and GC. As summarized in the table below (Supplementary Table 7 of the revised manuscript), the background-corrected recovery was 84.5, 85.6, 80.3, 79.3, and 79.0% for 1.0, 2.5, 5.0, 7.5, and 10.0 fM (concentrations spiked into the healthy serum), respectively, and qPCR variability, defined as coefficient of variation (CV (%) = 100 × standard deviation / mean), ranged from 8.28 to 12.97 %, indicating that the qPCR data is clearly within an acceptable range of variability according to the criterion (CV≦15%) recommended for qPCR-based quantitative assays in regulatory bioanalytical studies (*Mol. Ther. Methods Clin. Dev*. **20**, 152−168 (2021)).

Added miR-196a	Ct-1	Ct-2	Ct-3	Mean Ct ± s.d.	qPCR measured concentration (fM, mean ± s.d.)	Background-corrected concentration (fM)	Recovery (%)	qPCR CV (%)
0 fM	33.49	33.86	33.57	33.64±0.19	0.20±0.03	NA	NA	9.88
1.0 fM	31.36	31.1	31.26	31.24±0.13	1.05±0.10	0.85	84.45	9.84
2.5 fM	30.02	30.21	29.98	30.07±0.12	2.34±0.19	2.14	85.6	11.70
5.0 fM	29.41	29.07	29.18	29.22±0.17	4.22±0.49	4.02	80.29	8.28
7.5 fM	28.58	28.84	28.59	28.67±0.15	6.15±0.61	5.96	79.33	9.12
10.0 fM	28.11	28.33	28.37	28.27±0.14	8.10±0.80	7.9	79.01	12.97

Standard curve: Ct = -3.42 log10[C] + 22.35; R^2 = 0.998.

The above methodology of RT-qPCR was described in the Methods section of revised manuscript (p. 22,23).

[Comment 3]

Lack of Interference and Matrix Controls

The assay is described as working directly in serum without extraction. However, no data are provided to address common confounders such as hemolysis, RNase activity, sample storage conditions, freeze-thaw cycles, or nonspecific serum effects. Given the complexity of serum, the absence of interference testing is a significant weakness, especially for a study that emphasizes clinical applicability.

The manuscript does not adequately acknowledge or discuss prior reports demonstrating direct detection of miRNAs in raw plasma or serum samples. There are multiple published platforms that claim amplification-free or minimal-processing detection of circulating miRNAs. The current manuscript does not provide a meaningful comparison of performance metrics such as limit of detection, dynamic range, assay time, specificity, or clinical validation scale. Without a clear and balanced discussion positioning this platform relative to existing direct-detection technologies, it is difficult to assess the true novelty and advantage of the proposed system. The manuscript would benefit substantially from a critical discussion of how this approach differs mechanistically and practically from prior amplification-free miRNA detection strategies. At present, the claim of conceptual and clinical superiority is not sufficiently justified in the context of the existing literature.

Revision:

To address the reviewer's concern, we performed additional experiments and also discussed prior reports on direct detection of miRNAs as follows.

1) Interference and matrix controls

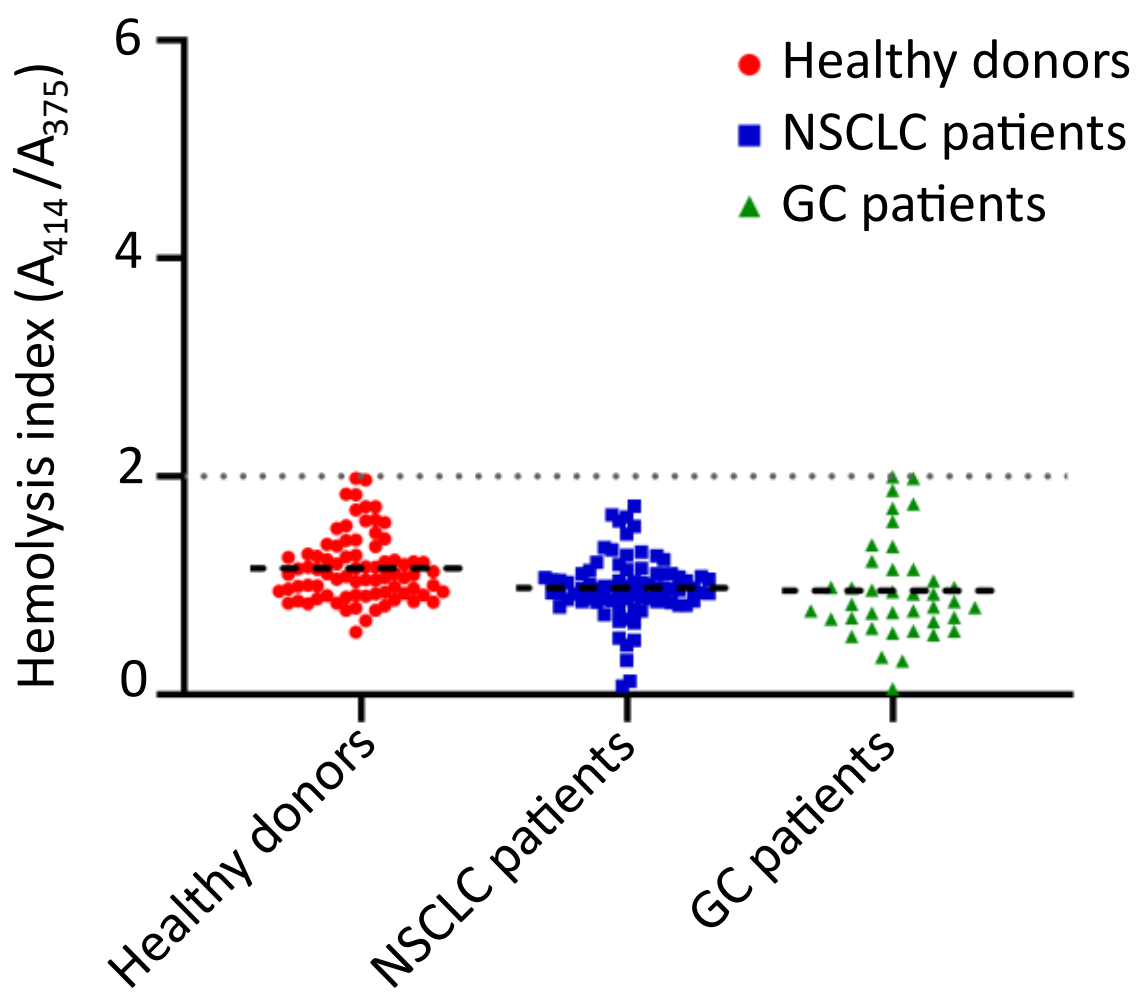

• Regarding hemolysis: We measured hemolysis index (absorbance ratio, $A_{414\ nm}/A_{375\ nm}$) for all serum samples used in this study (80 NSCLC patients, 40 GC patients, and 80 healthy donors). Reportedly, the hemolysis index below 2.0 represents hemolysis-free state of samples (*Clin. Biochem.* **47**, 218–220 (2014)). As shown in the figure above (Supplementary Figure 27 of the revised manuscript), no serum samples exhibited the index ($A_{414\ nm}/A_{375}$) above 2.0, indicating that all serum samples are hemolysis-free specimens.

• Regarding RNase activity: We performed an RNase-interference assay by spiking synthetic let-7a into healthy serum (at 0 ~10 fM) in the presence or absence of Protector RNase Inhibitor (0.5 U/µL; Roche) (*Nat. Commun.* **11**, 6075 (2020)). As shown in Supplementary Fig. 17 of the revised manuscript, RNase inhibition does not significantly change the assay performance, that is, LOD still remains at femtomolar level although LOD slightly decreased to ~ 2.5 fM from ~ 5 fM, indicating that serum RNase does not critically affect the assay performance under the assay conditions, which is presumably because each serum (50 µL) is mixed, or diluted with 150-µL assay solution and further because once miRNA-ssDNA hybrids are formed and subsequently bound by HBD of FRET acceptor, it is resistant to RNase degradation. Furthermore, it seems that in clinical detection of miRNA in patient serum, miRNA that survived against serum RNase is detected in reality. Consequently, the effect of serum RNase on miRNA detection by FRET platform seems to be not significant.

• Regarding sample storage and freeze-thaw stability: All serum specimens were aliquoted into a single-use volume upon receipt from the supplier (KIRAMS Radiation Biobank, Seoul, Republic of Korea) and immediately stored at − 80 °C, and each aliquot was thawed once before the assay. It should be noted that no specimen underwent repeated freeze-thaw cycles. Reportedly, circulating miRNAs in serum remain stable under prolonged storage at − 80 °C and even through multiple freeze-thaw cycles (*Proc. Natl. Acad. Sci. U.S.A.* **105**, 10513–10518 (2008)). The sample handling conditions above were added to the Methods section (p. 20,21) of revised manuscript.

• Regarding nonspecific serum effects: As discussed in detail in the response to Comment 2 of Reviewer #2, we tested the cross-reactivity between ssDNA probes of FRET donor and various miRNAs with different sequences. As presented in the figure below (Supplementary Fig. 18 of the revised manuscript), each ssDNA probe emits the highest readout signal (FRET ratio) for its complementary target miRNA, whereas the readout

signals for the other non-target miRNAs and negative control (healthy serum only) are negligible, indicating that FRET platform enables highly selective, target-specific detection of miRNA.

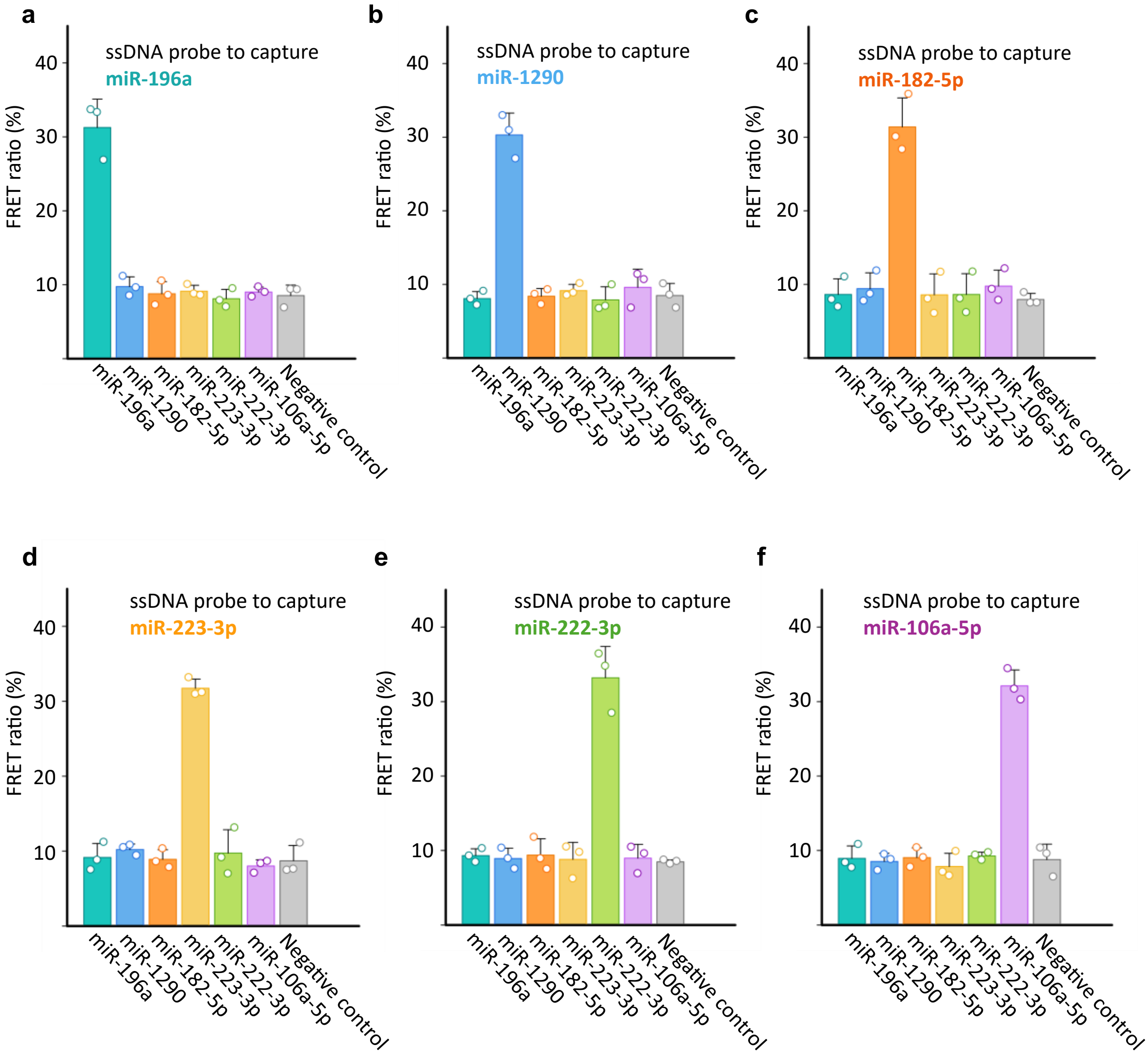

2) Comparison between FRET platform and prior direct-detection technologies

According to the reviewer's comment, we introduced recent literatures reporting "direct-detection" methods for circulating miRNAs in serum/plasma in a newly added table below (Supplementary Table 6 of revised manuscript), which includes single-molecule electro-optical nanopore (*Nat. Commun.* **12**, 3515 (2021)), nanophotonic BiMW biosensor (*Anal. Chem.* **94**, 14659–14665 (2022)), high-throughput nanoplasmonic LSPR assay (*ACS Sens.* **8**, 1085–1100 (2023)), CRISPR-Cas13a Gemini system (*Nat. Commun.* **15**, 2901 (2024)), and split-crRNA CRISPR/Cas12a (SCas12a) assay (*Nat. Commun.* **15**, 8342 (2024)).

Reference	Workflow	Platform technology / Analytical instrument	LOD & Assay conditions	Assay selectivity	Clinical cohort
Nat. Commun. **12**, 3515 (2021)	- Unprocessed human serum<br>- Direct serum analysis	Single-molecule electro-optical nanopore	- LOD: 5–8 fM<br>(Test range of 0.1 to 10 pM for ~80 min)	- Single-/double-nucleotide mismatch discrimination	- PCa serum (n = 10) with 2-miRNA markers<br>- Localized (n = 3) metastatic (n = 3) remission (n = 7) with 3 miRNA markers
Anal. Chem. **94**, 14659 (2022)	- Human plasma<br>- Direct one-step<br>- Real-time, no extraction or amplification	Nanophotonic BiMW biosensor	- LOD: 25 pM<br>(0.5 to 100 nM tested for < 45 min)	- Non-complementary miR-210-3p shows no detectable response	- NSCLC plasma (n = 20) Healthy (n = 20) with 1 miRNA marker
ACS Sens. **8**, 1085 (2023)	- Unprocessed plasma<br>- Microsamples	High-throughput nanoplasmonic LSPR assay	- LOD: ~130 aM<br>(10 to 10^{11} aM tested for ~17 h)	- High specificity against non-complementary miRNA in plasma<br>- No single-base mismatch metric reported	- PDAC plasma (n = 75) Chronic Pancreatitis (n = 35) Healthy (n = 60) with 2 miRNA markers
Nat. Commun. **15**, 2901 (2024)	- Serum<br>- RNA extraction<br>- No target amplification	CRISPR-Cas13a Gemini System (fluorescence + lateral flow)	- LOD: 733 fM<br>(1 to 100 pM tested for ~60 min)	- Dual-target simultaneous detection	- Breast cancer serum (n = 30) healthy (n = 15) with 2 miRNA markers<br>- EBV detection (n = 75 positive, n = 52 negative) with 2 small RNA markers
Nat. Commun. **15**, 8342 (2024)	- Plasma/cells<br>- RNA extraction<br>- No reverse transcription or amplification	Split crRNA CRISPR/Cas12a (SCas12a) fluorescence + lateral flow	- LOD: ~100 fM<br>(10 to 10^6 fM tested for ~50 min)	- Single-point mutation discrimination in both DNA and miRNA<br>- Distinguishes mature miRNA from pre-miRNA	- Cervical cancer plasma (n = 7) Healthy (n = 3) with 1 miRNA marker<br>- HPV16 detection in vaginal secretion (n = 25)
**Present study**	- Serum<br>- No extraction/ purification/ amplification	RNaseH1-dependent FRET platform using off-the-shelf instruments (well plate, microplate reader)	- LOD: 5 fM<br>(10 to 10^9 fM tested for ~60 min, for 7 miRNA markers)	- Single- and double-mismatch discrimination<br>- Mismatch position-independent discrimination<br>- No cross-reactivity for non-target miRNAs	- NSCLC serum (n = 80) Healthy (n = 80) with 3 miRNA markers<br>- GC serum (n = 40) Healthy (n = 40) with 3 miRNA markers

This table provides a broad spectrum of comparisons with FRET platform in terms of workflow, platform technology/analytical instruments, LOD with dynamic test range and assay time, assay selectivity, and clinical cohort size with miRNA markers tested. Several important, distinct advantages of FRET platform are summarized as follows: 1) FRET platform was tested with the largest cohort size (NSCLC: 80 patients + 80 healthy; GC: 40 patients + 40 healthy) through detecting 3 different miRNA markers per each type of cancer and thus is likely to deliver more reliable diagnostic performance with minimizing inter-patient variability; 2) FRET platform enables clinical diagnosis using

undistinguished, off-the-shelf instruments such as well-plates and microplate reader, without using elaborately designed, sophisticated instruments such as electro-optical nanopore, nanophotonic BiMW biosensor, nano-plasmonic LSPR instrument, and CRISPR+LFA system (requiring RNA extraction prior to diagnosis); and 3) except for the case of nano-plasmonic LSPR assay claiming ~ 130-aM LOD, FRET platform's LOD is significantly better or comparable.

We added the above discussion to the text of revised manuscript (p. 16,17).

[Comment 4]

Mechanistic Claims Rely Heavily on Simulations

The mismatch discrimination mechanism is supported primarily by molecular dynamics simulations and docking analyses. While these are well executed, computational modeling cannot replace experimental validation of binding geometry and kinetics. Direct biophysical measurements (e.g., SPR or ITC) would substantially strengthen the mechanistic claims.

Revision:

In the present study, the mechanistic interpretation is performed not only through MD and docking simulations but also through experimental analyses such as ELISA-like plate assay (Fig. 4i of the revised manuscript) and mismatch discrimination analysis (Fig. 4a, Supplementary Fig. 13 of the revised manuscript). Through ELISA-like plate assay, we assessed the effect of single- and double base mismatch on the binding affinity between ssDNA-miRNA hybrid formed on FRET donor and HBD of FRET acceptor with estimating dissociation constants, demonstrating that the binding affinity becomes progressively weaker as the number of mismatched base pair increases, which exactly corresponds to the results of simulation studies. Further, through another experimental analysis, we attempted to figure out whether base-mismatched binding between ssDNA probe and non-complementary miRNA can be distinguished by the diagnostic signals (FRET ratios) from FRET platform and finally confirmed that perfectly matched hybrid helix emitted a significantly higher FRET ratio with clearly distinguishing mismatched hybrids.

One key information that remains veiled yet in mismatch discrimination is whether the location of mismatch(es) within hybrid helix (ssDNA-miRNA) affects the assay performance of FRET platform. It seems reasonable that this needs to be unveiled using

the same FRET platform instead of using other biophysical assay tools like SPR. In the manuscript revision, we attempted to reveal effect of mismatch position on the diagnostic performance of FRET platform through additional experiments as described below.

We mutated the sequence of ssDNA probe (5'-AAC TAT ACA ACC TAC TAC CTC A-3') complementary to let-7a miRNA (a cancer-associated miRNA marker, 3'-UUG AUA UGU UGG AUG AUG GAG U-5'), resulting in the preparation of 4 different ssDNA probes as follows: 5'-AAC **C**AT ACA ACC TAC TAC CTC A-3' (for a single mismatch proximal to 5' end), 5'-AAC TAT ACA ACC TAC TAC C**C**C A-3' (for a single mismatch proximal to 3' end), 5'-AAC TAT ACA ACC **C**AC TAC CTC A-3' (for a single mismatch in middle region), and 5'-AAC **C**AT ACA ACC TAC TAC C**C**C A-3' (for double mismatch). Then, we spiked let-7a into human healthy serum at 10, 10^2, 10^3, and 10^4 fM and measured readout signals (FRET ratios) from the detection of let-7a by FRET platform using each of let-7a-complementary- and 4 mutated ssDNA probes above.

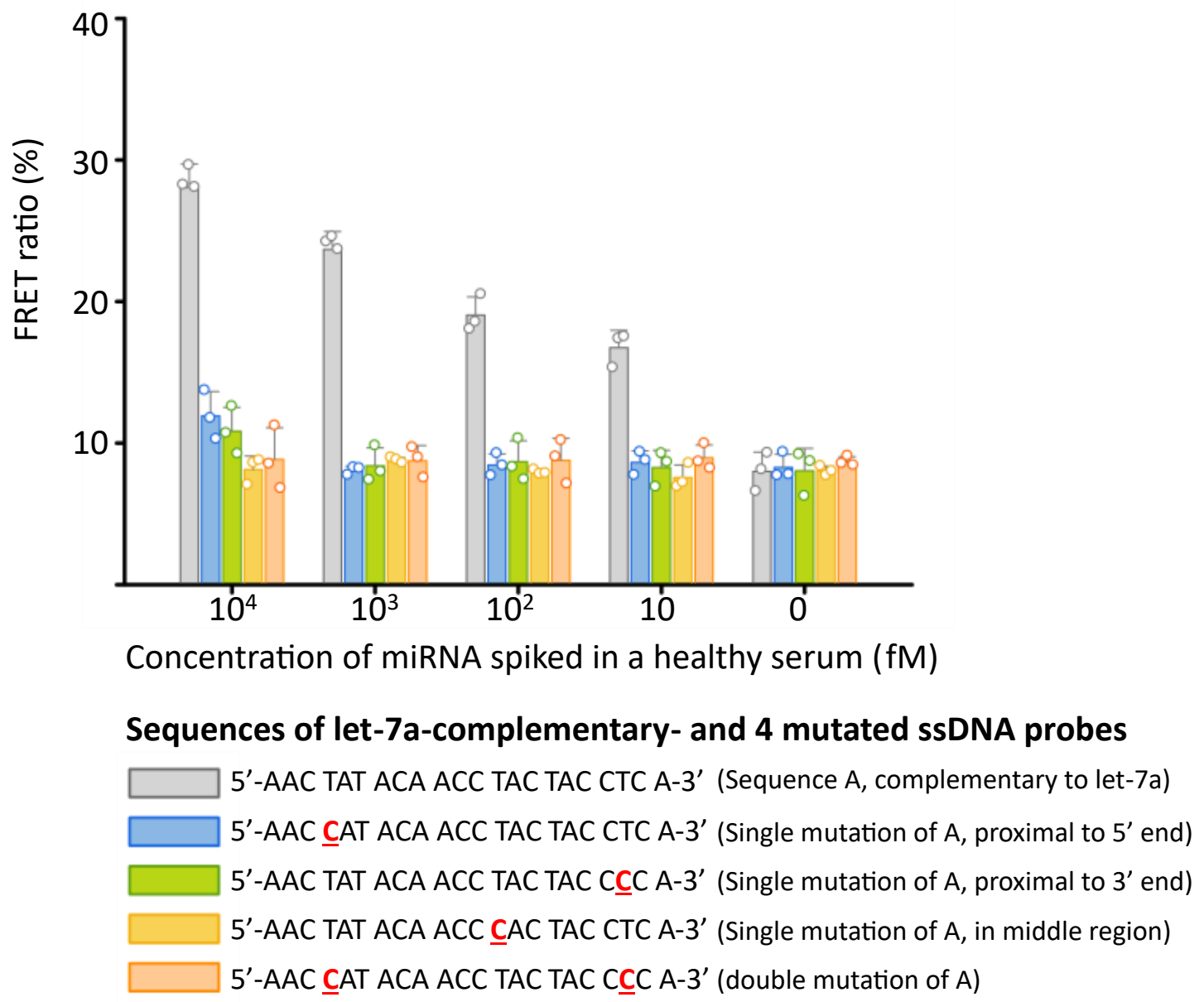

As shown in the figure above (Supplementary Figure 14 of the revised manuscript), only let-7a-complementary ssDNA probe emits distinctly the highest and concentration-dependent signals, while the other mutated ssDNA probes, which lead to single- or double mismatch at various positions upon the capture of let-7a, emit notably lower, negligible signals, indicating that the discrimination of mismatched hybrids by HBD of FRET acceptor is not influenced by mismatch position. This result is well explainable with the native mechanism of recognizing DNA-RNA hybrids by HBD, which is that HBD senses overall A-form minor-groove geometry of hybrid helix regardless of particular mismatch positions.

We discussed the additional results above in the text of revised manuscript (p. 9,10).

[Comment 5]

Reproducibility and Practical Considerations

There is no information on batch-to-batch reproducibility, inter-assay variability, stability of the nanocage constructs, or scalability of production. These issues are critical for any diagnostic platform but are not addressed.

<u>Revision</u>:

According to the reviewer's comment, we performed the following additional experiments in the manuscript revision. To evaluate batch-to-batch reproducibility and inter-assay variability, we produced three independent batches of both the EGF_{mut1}-linked nanocage (variant 20) and the HBD-linked FRET acceptor from three separate *E. coli* cultures (400 mL each). Then immediately, FRET platform was prepared through constructing FRET donor by attaching ssDNA probes onto the EGF_{mut1}-linked nanocage produced from each batch and subsequently was used for detecting miRNA (let-7a) spiked into healthy serum at 0 (blank), 10, 10^2, and 10^3 fM, while the remaining FRET platform were aliquoted into a single-use volume and stored at − 80 °C for 6 weeks. Afterwards, the frozen FRET platform was thawed at 2-week intervals and used to detect miRNA (let-7a). As shown in the figure below (Supplementary Figs. 12a-d of the revised manuscript), the detection signals (FRET ratios) from the three separately prepared FRET platforms – each of which was prepared from each batch − were not significantly distinguished from each other at each concentration of let-7a, which was consistently observed every 2 week for 6 weeks, thus indicating that batch-to-batch- and inter-assay variabilities are negligible.

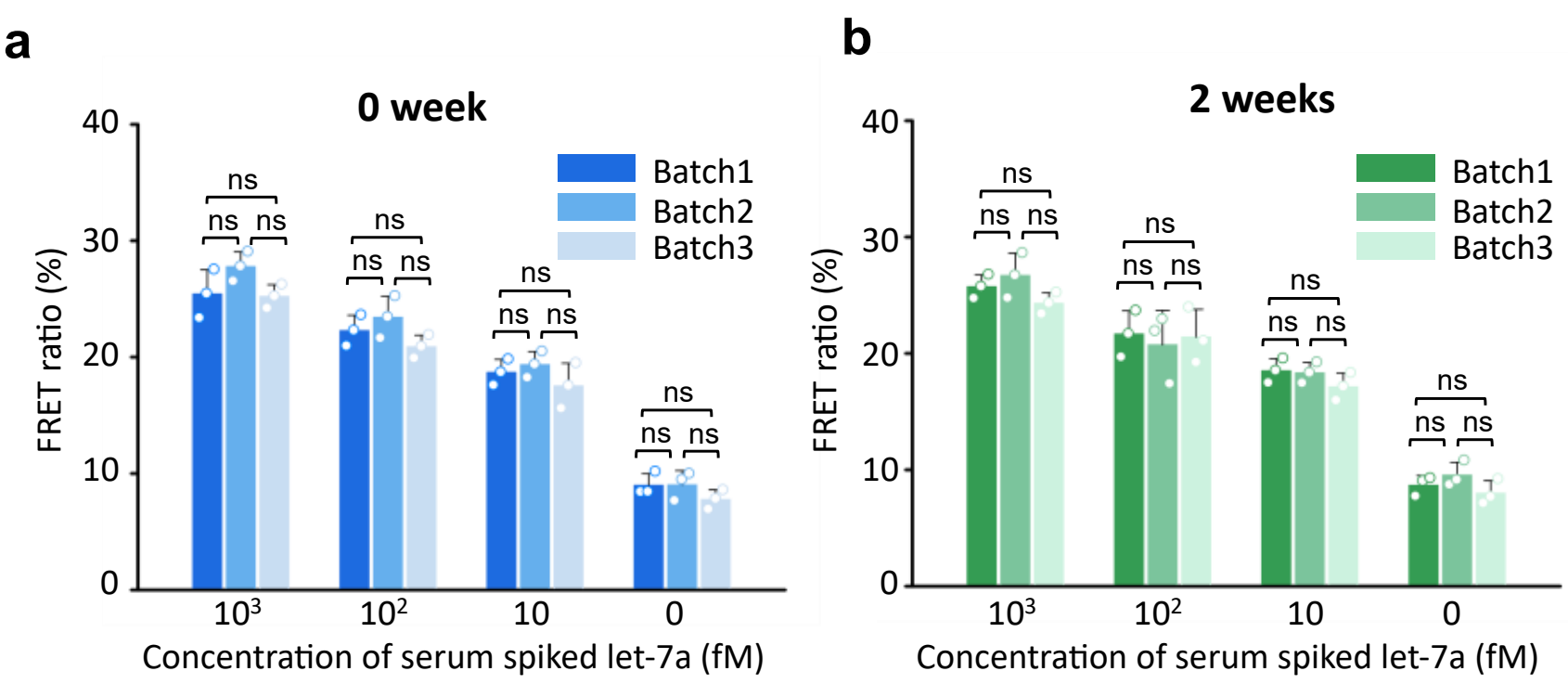

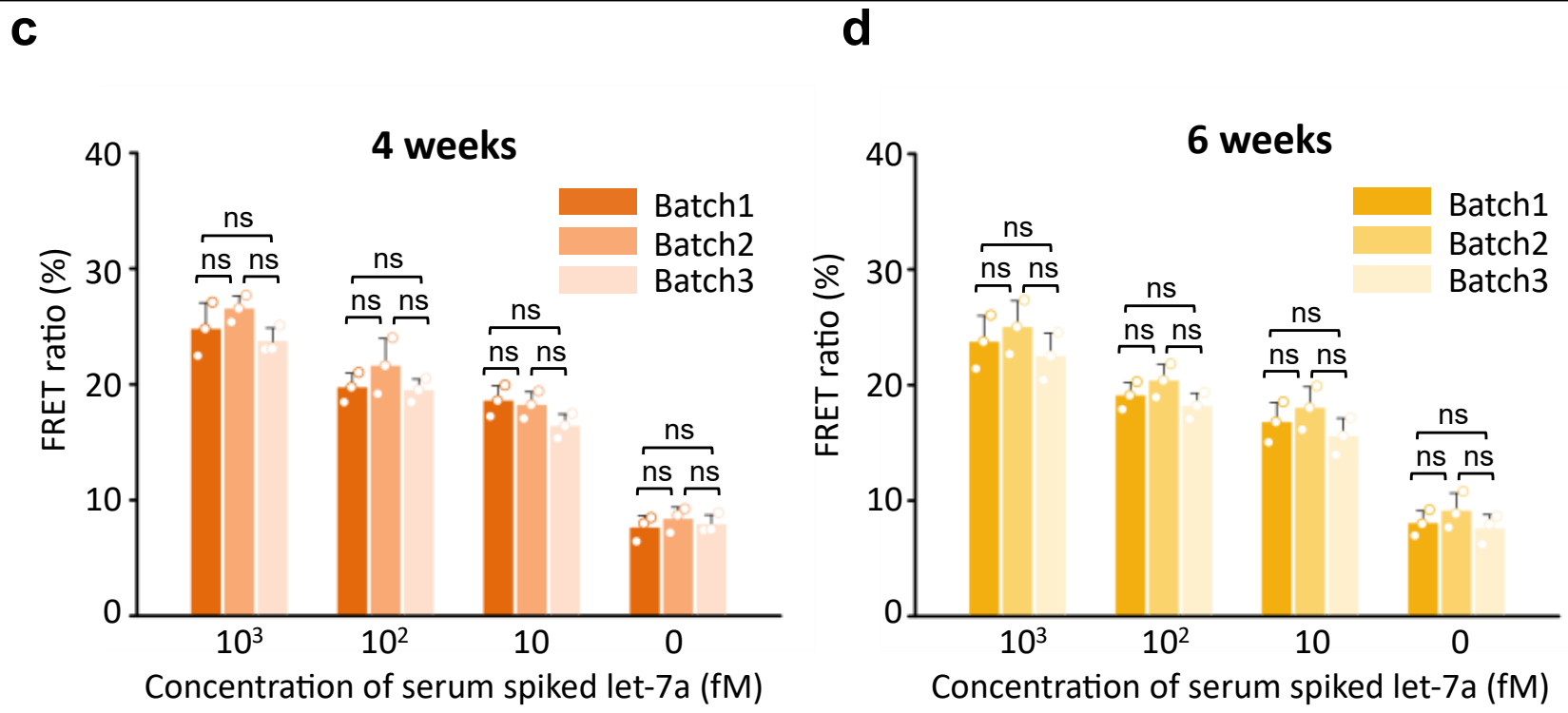

Next, to evaluate whether the stability of EGF_{mut1}-linked nanocage – which is critical to stable construction of FRET donor – lasts during the 6-week period of storage at − 80 °C, the frozen nanocage was thawed at 2-week intervals and analyzed through DLS and TEM. As shown in the figure below (Supplementary Figure 7 of the revised manuscript), DLS and TEM analyses show that its spherical shape and mean diameter (approximately 38 to 40 nm with PDI of 0.002~0.003) is nearly constant with neither inter-nanoparticle aggregation nor structural collapse at all time points, demonstrating the stable nature of FRET platform.

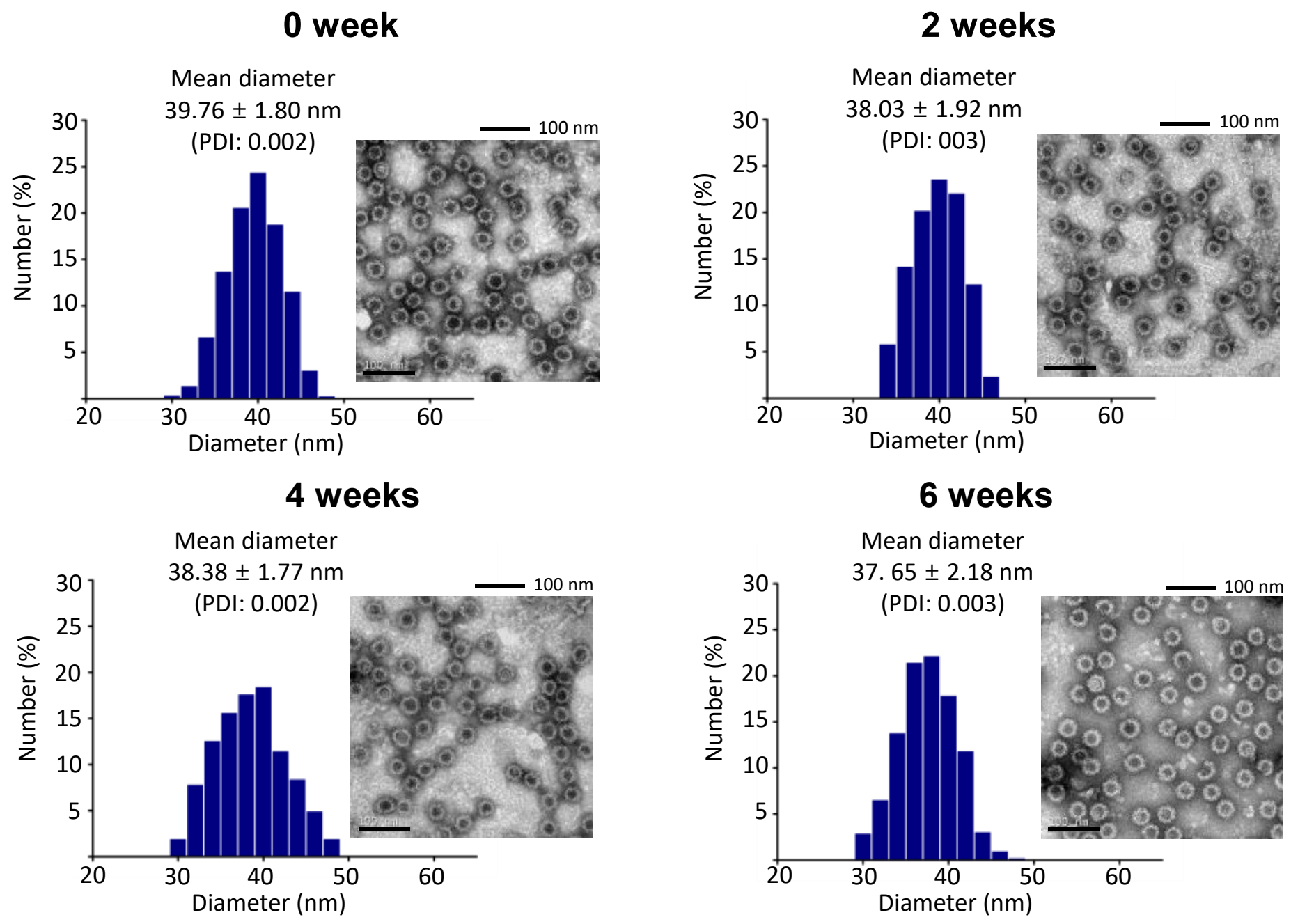

The above results were discussed in the text of revised manuscript (p. 6, 8, 9).

Minor comments:

[Comment 1]

Statistical methods are not described in sufficient detail.

Revision:

According to the reviewer's comment, we added a new "Statistical analysis" subsection to the Methods (p. 26, 27) of revised manuscript, which reads as follows: "ROC curves were plotted, and AUC (Area Under the Curve) values with 95% asymptotic confidence intervals were calculated using GraphPad Prism (version 10.6.1; GraphPad Software, San Diego, CA, USA) to evaluate the diagnostic performance of FRET platform for each miRNA marker. Also, using Youden index (J = sensitivity + specificity − 1), which is a widely used criterion for selecting a cutoff point that maximizes overall classification performance in ROC analysis[58,63], we estimated the optimal cutoff of FRET ratio for each of six miRNA markers for NSCLC and GC through calculating J at every possible threshold along the ROC curve to find a threshold (optimal cutoff) that yields maximum J. Spearman's rank correlation coefficients (ρ) were calculated using GraphPad Prism to assess the monotonic agreement between the readout signals from FRET platform and RT-qPCR, resulting in nonlinear (logarithmic) relationship between the two readout signals. FRET ratios for perfectly matched and mismatched miRNA targets (Fig. 4a) were analyzed by ordinary two-way ANOVA with miRNA identity (let-7a, let-7f, and let-7g) and miRNA concentration as fixed factors using GraphPad Prism. miRNA concentration was treated as a categorical factor, and the full model including the miRNA identity × concentration interaction was fitted. For post hoc analysis, pairwise comparisons among miRNAs were performed at each concentration by comparing column means within each row using Tukey's multiple comparisons test. Each concentration was treated as a separate family of comparisons, and multiplicity-adjusted P values were reported. $P < 0.05$ was considered statistically significant and is denoted as *$p < 0.05$, **$p < 0.01$, ***$p < 0.001$; ns, not significant. All FRET measurements were performed in technical triplicate. Box plots were generated using SigmaPlot (version 10.0; Systat Software, San Jose, CA, USA) and show median (center line), interquartile range (box), 1.5× interquartile range (whiskers), and individual data points. Data are presented as mean ± s.d. unless otherwise stated. All statistical tests were two-tailed unless otherwise stated."

[Comment 2]

The language throughout the manuscript is overly definitive relative to the data presented.

Revision:

In the revised manuscript, we corrected the text with avoiding over-definitive languages.

[Comment 3]

The discussion does not sufficiently position this work relative to other amplification-free miRNA detection technologies, including CRISPR-based systems.

Revision:

As described in the response to the major Comment 3 of reviewer #3, we discussed other amplification-free, direct miRNA detection technologies including CRISPR-based systems (p. 16,17 in the text and Supplementary Table 6 of the revised manuscript).

Revision Details

Reviewer #3:

The revised manuscript is improved and addresses many of the major concerns raised previously. The added mechanistic studies, mismatch analyses, reproducibility experiments, and expanded methodological details strengthen the work significantly. The amplification-free FRET platform for direct serum miRNA detection is innovative and potentially important for cancer diagnostics. However, minor issues should still be addressed before publication:

[Comment 1]

More clinical information should be provided for the patient cohorts, including smoking status, histology, treatment status, and possible confounding factors.

Revision:

According to the reviewer's comment, we added histologic type and smoking status of all NSCLC and GC patients to Supplementary Tables 1 and 4, respectively, with the following statement: "All serum specimens of patients were collected before receiving anticancer treatment." (p. 20) and "The demographic, clinical, and histopathological characteristics and smoking status of the NSCLC- and GC patients and the demographic information of healthy donors are summarized in Supplementary Tables 1, 4, and 2, respectively." (p. 20-21).

[Comment 2]

The manuscript should acknowledge the lack of an independent external validation cohort as a limitation.

Revision:

According to the reviewer's comment, we have added the following statement to the second revised manuscript (p. 20): "Although several independent external cohorts are necessary for complete clinical validation, here we performed a clinical diagnosis using the cohort samples from a single biobank (the KIRAMS Radiation Biobank)."

[Comment 3]

Claims that the platform outperforms RT-qPCR and other methods should be moderated

unless directly validated under comparable conditions.

Revision:

According to the reviewer's comment, we revised the text of manuscript as follows: 1) p.13: we removed the following phrase, ", suggesting that FRET platform has an advantage over RT-qPCR in early diagnosis of NSCLC." and 2) p.17: we changed the following sentence, "Notably, compared to RT-qPCR, the much larger difference in readout signals (FRET ratios) between stage I- and II patients and healthy group was observed in the diagnosis by FRET platform for both NSCLC and GC, and thus FRET platform is likely more effective for early cancer diagnosis." to "Notably, a significantly large difference in readout signals (FRET ratios) between stage I- and II patients and healthy group was observed in the diagnosis by FRET platform for both NSCLC and GC, and thus FRET platform is likely effective for early cancer diagnosis."

[Comment 4]

The distinction between technical and biological replicates should be clarified throughout the manuscript.

Revision:

According to the reviewer's comment, we clarified technical and biological replicates in the revised manuscript (p. 28) as follows: "In this study, *in vitro* analyses or clinical diagnoses were performed properly through the measurement of both technical and biological replicates. Technical-replicate measurement denotes that the measurement was repeated three times for every single specimen, and the results were presented as mean ± s.d. For biological-replicate measurements in clinical diagnoses, we used multiple serum samples for a single type of cancer (80 sera for NSCLC and 40 sera for GC) as well as for healthy control (80 sera) to minimize inter-patient variability. (For example, in Figure 5 and Supplementary Figure 24, multiple data points at each clinical stage as well as at healthy control represent biological replicates, and each data point is the mean of measured values from three technical replicates.)"

[Comment 5]

Additional details are needed for statistical analyses, including testing for normality, handling of outliers, and multiple-comparison corrections.

Revision:

In all analyses, no data points were excluded as outliers, that is, all measured values were used for data analysis.

Through the residual normality test upon using two-way ANOVA to statistically compare two different data groups (Fig. 4a, Supplementary Fig. 12), we confirmed that the residuals were not significantly deviated from a normal distribution, which was validated through multiple protocols including D'Agostino–Pearson, Anderson–Darling, Shapiro–Wilk, and Kolmogorov–Smirnov.

For multiple-comparison corrections, we used Tukey's multiple comparisons test, and accordingly multiplicity-adjusted P values were reported in this manuscript, which was already stated in detail in the first revised manuscript (p. 27): "For post hoc analysis, pairwise comparisons among miRNAs were performed at each concentration by comparing column means within each row using Tukey's multiple comparisons test. Each concentration was treated as a separate family of comparisons, and multiplicity-adjusted P values were reported. $P < 0.05$ was considered statistically significant and is denoted as *$p < 0.05$, **$p < 0.01$, ***$p < 0.001$; ns, not significant."

The above statements about statistical analyses were added to the Methods (p. 26,27).

[Comment 6]
The Discussion should better address potential serum matrix effects and assay variability in real clinical samples.

<u>Revision</u>:
Prior to the clinical diagnosis using healthy- and patient sera, we investigated if serum matrix effects are caused by hemolysis and serum RNase. It was confirmed that hemolysis (a major matrix confounder) is negligible in every serum specimen, because hemolysis index is less than 2.0 for all serum samples (Supplementary Fig. 27). Further, we demonstrated that the effect of endogenous serum RNase activity on assay performance is not significant (Supplementary Fig. 17).

We added the following statement to the Conclusion (p. 16): "Prior to the clinical diagnosis, we confirmed that serum matrix effects by hemolysis and serum RNase are negligible (Supplementary Figs. 17, 27), but matrix heterogeneity and assay variability across clinical serum specimens remain to be characterized with other independent

clinical cohorts."

[Comment 7]

Specificity testing against additional closely related miRNAs would strengthen the conclusions, or this limitation should be acknowledged.

Revision:

In this study, the specificity of FRET platform is supported by the following two evidences: 1) even single-nucleotide mismatches are accurately discriminated irrespective of mismatched position, which was demonstrated using the let-7 family miRNAs (Fig. 4a, Supplementary Fig. 14) and 2) testing the six clinical markers exhibits that FRET platform shows no cross-reactivity (Supplementary Fig. 18). According to the reviewer's comment, we added the following statement to the Conclusion (p. 16): "…. which were selected based on the previous findings that they are significantly upregulated in each type of cancer tissues[29-33, 34-36, 66-69], although testing additional closely related miRNAs would strengthen diagnostic specificity."

[Comment 8]

Some statements regarding clinical applicability appear overstated and should be toned down.

Revision:

We carefully revised the text of manuscript with avoiding overstatement about clinical applicability.

[Comment 9]

The ROC cutoff analysis should clarify whether cutoff determination and testing were performed using the same cohort.

Revision:

By adding the following statement to the revised manuscript (p. 27), we clarified that cutoff determination and testing were performed using the same cohort in ROC cutoff analysis: "The above statistical analyses for ROC, AUC, and optimal cutoff values were performed using the same cohort samples from a single biobank (the KIRAMS Radiation Biobank)."

[Comment 10]

Minor language editing and simplification of several long sentences would improve readability.

Revision:

We corrected carefully minor grammatical and typographical errors and simplified several long sentences to improve readability of manuscript text.
